# Global, regional, and national burden of mental disorders and substance use disorders in older adults from 1990 to 2021: a systematic analysis and future trend prediction study

**DOI:** 10.3389/fpsyt.2025.1638646

**Published:** 2025-09-24

**Authors:** Yitian Gao, Wanqiong Zhou, Qiuyi Wang, Qianyi Zhai, Lanshu Zhou

**Affiliations:** ^1^ College of Nursing, Shanghai University of Traditional Chinese Medicine, Shanghai, China; ^2^ Key Laboratory of Geriatric Long-term Care, Naval Medical University, Shanghai, China; ^3^ Department of Nursing, Shuguang Hospital Affiliated with Shanghai University of Traditional Chinese Medicine, Shanghai, China

**Keywords:** mental disorder, substance use disorder, prevalence, years lived with disability, SUDs

## Abstract

**Introduction:**

As the global population ages, mental health has become a major public health issue affecting healthy aging. To estimate the burden, trends, and inequalities of mental disorders and substance use disorders (SUDs) among older adults aged ≥60 years at the global, regional, and national levels from 1990 to 2021.

**Methods:**

Data from the Global Burden of Disease Study 2021. The study focused on the burden of mental health conditions, analyzing mental disorders and SUDs prevalence, years lived with disability (YLDs), and average annual percentage change (AAPC), encompassing eight age groups (60–64 years, 65–69 years, 70–74 years, 75–79 years, 80–84 years, 85–89 years, 90–94 years, ≥95 years) for both men and women.

**Results:**

Globally in 2021, 1.09 billion individuals aged ≥60 years included 161.3 million (14.8%) with mental disorders and 19.2 million (1.8%) with SUDs. Mental disorders accounted for 22.8 million YLDs versus 2.10 million from SUDs in this population. From 1990 to 2021, age standardized prevalence of mental disorders increased by 3.7%, while SUDs declined by 8.7%, with lower SDI levels correlating with higher YLDs burdens overall. Over the next fifteen years, projections indicating stagnant trends in SUDs alongside slight declines in mental disorders highlight persistent unmet needs.

**Discussion:**

Global population aging demands heightened prioritization of mental healthcare for older adults. Today, mental and substance use disorders persist as major contributors to disease burden in this population. Our findings call for equitable mental resource allocation, age-adapted diagnostic tools, and policies targeting late-life psychosocial vulnerabilities.

## 1 Introduction

Mental health conditions—encompassing mental disorders and substance use disorders—have been emphasized by the Lancet Commission on global mental health and sustainable development as both a fundamental human right and a prerequisite for sustainable development in all countries (1). Since the publication of the first Global Burden of Disease (GBD) study in the early 1990s, evidence has grown that mental disorders constitute a major contributor to the global disease burden, with particularly high prevalence in the 15–19 and 60–64 age groups (2, 3). The GBD 2021 report quantifies this impact through disability metrics: depression ranks as the second-leading cause of years lived with disability worldwide (56.3 million), followed by anxiety disorders in sixth place (42.5 million YLDs) (3, 4). However, mental health service provision lags critically behind needs, particularly for the elderly (5, 6).

According to the GBD 2021 diseases hierarchy, mental disorders include schizophrenia, depressive disorders, bipolar disorder, anxiety disorders, conduct disorder, eating disorder, autism spectrum disorders, attention-deficit/hyperactivity disorder, idiopathic developmental intellectual disability, and other mental disorders. Although the GBD categorizes substance use disorders (SUDs) separately from mental disorders, to provide a comprehensive overview of mental health according to the International Classification of Diseases (ICD), we also included the SUDs in this study (7). The definition and cataloging system codes (ICD-10, an DSM-IV-TR) (8) to which each disorder is mapped appear in **eTable 1** in **Supplementary 1**.

Despite ongoing global demographic shifts from younger to older age groups and increased longevity, with most populations now surviving into their 60s and beyond, geriatric psychiatry remains underprioritized (3, 9). Existing studies have mostly focused on the analysis of characteristics related to mental disorders in the overall or adolescent population, and there is a lack of targeted analyses for the elderly population (7, 10). We found no publications dedicated to reporting global or local findings from GBD 2021 on mental disorders specifically in older age groups.

To address this disparity, we analyzed age-specific and sex-stratified trends in prevalence, YLDs, and annual burden changes associated with mental disorders and SUDs among adults aged 60 years and older at global, regional, and national levels from 1990 to 2021.

## 2 Methods

### 2.1 Overview

We utilized the data from the GBD 2021 to conduct this research and analysis. The study focused on the burden of mental health conditions, analyzing mental disorders and SUDs prevalence and YLDs at global, regional, and national levels, encompassing eight age groups (60–64 years, 65–69 years, 70–74 years, 75–79 years, 80–84 years, 85–89 years, 90–94 years, ≥95 years) for both men and women.

### 2.2 Data source

All data in this study are extracted from the results of Global Burden of Disease Study 2021, obtaining datasets from the Global Health Data Exchange covering the period from 1990 to 2021. GBD 2021 contains information on 371 diseases and injuries from 204 countries and territories in 21 regions, including data on all types of mental disorders and SUDs (4). The 371 diseases and injuries included in GBD 2021 were organized within a cause hierarchy with four levels plus the Level 0 aggregate of all causes.

In this study, we exclusively analyzed two level 2 mental health causes (mental disorders and SUDs) and ten level 3 mental health causes (schizophrenia, depressive disorders, bipolar disorder, anxiety disorders, autism spectrum disorders, attention-deficit/hyperactivity disorder, idiopathic developmental intellectual disability, other mental disorders, alcohol use disorders, and drug use disorders) for people aged 60 and older years (**eFigure 1** in **Supplementary 1**). Conduct disorder and eating disorder were excluded because: (1) conduct disorder is strictly defined for individuals under 18 years of age, and (2) epidemiological data on eating disorders are unavailable for populations aged ≥60 years.

In GBD 2021, prevalence and incidence were modelled using DisMod-MR 2.1 (Disease Modelling Meta-Regression, version 2.1). DisMod-MR 2.1 is a Bayesian disease modelling meta-regression tool that generates internally consistent estimates of prevalence, incidence, remission, and mortality by sex, location, year, and age group (11). YLDs were calculated with a microsimulation process that used estimated age-sex-location-year-specific prevalent counts of non-fatal disease sequelae (consequences of a disease or injury) for each cause and disability weights for each sequela as the inputs. Because most mental disorders are not considered direct causes of death, years of life lost (YLL) cannot be estimated, and years of life lost can only be used to approximate disability-adjusted life-years (DALYs), so only the YLDs indicator was analyzed in this study (10). The 95% uncertainty intervals (UIs) for all metrics were computed using the mean estimate across 500 draws and reported as the 2.5th and 97.5th percentiles of that distribution.

### 2.3 Statistical analysis

Descriptive analyses were used to characterize the global burden of mental disorders and SUDs among adults aged ≥60 years. Rates in our estimates are shown per 100–000 population. Based on the world standard population reported in the Global Burden of Disease Study 2021, the age standardized rates were calculated through standardization (**Supplementary 1**).

We compared the age standardized prevalence and YLDs of mental disorders and SUDs across different age groups, sexes, regions, and countries. We estimated average annual percentage changes (AAPC) by joinpoint regression to measure the temporal trend (12). Joinpoint regression is used to detect significant changes in time trends by dividing longitudinal data into segments via piecewise regression. It identifies statistically significant inflection points and estimates trend changes by fitting linear segments. In our study, we used a model with seven segments (six joinpoints), implemented using the Joinpoint Regression Program (version 5.3.0; National Cancer Institute, Rockville, MD, USA). We then analyzed the relationship between the age standardized prevalence and YLDs rate per 100–000 population in 2021 and country development status using the GBD Socio-demographic Index (SDI). In addition, a hierarchy cluster analysis was conducted to classify 21 regions into four categories related to the estimated annual percentage changes (EAPC). Finally, the auto regressive integrated moving average model (ARIMA) was used to predicted the mental disorders and SUDs burden from 2021 to 2036. ARIMA comprises three components: autoregressive (p), integrated (d), and moving average (q), denoted as ARIMA(p, d, q) (13). Through automatic fitting, the optimal parameters for our study were ARIMA(2,0,0). All statistical analyses were conducted using R (version 4.3.2).

### 2.4 Patient and public involvement

No patients or members of the public were directly involved in this study. The study adhered to the Guidelines for Accurate and Transparent Health Estimates Reporting (GATHER) guidelines (14) (**eTable 9**, **Supplementary 1**).

## 3 Results

### 3.1 Global trends

**Figure 1:**
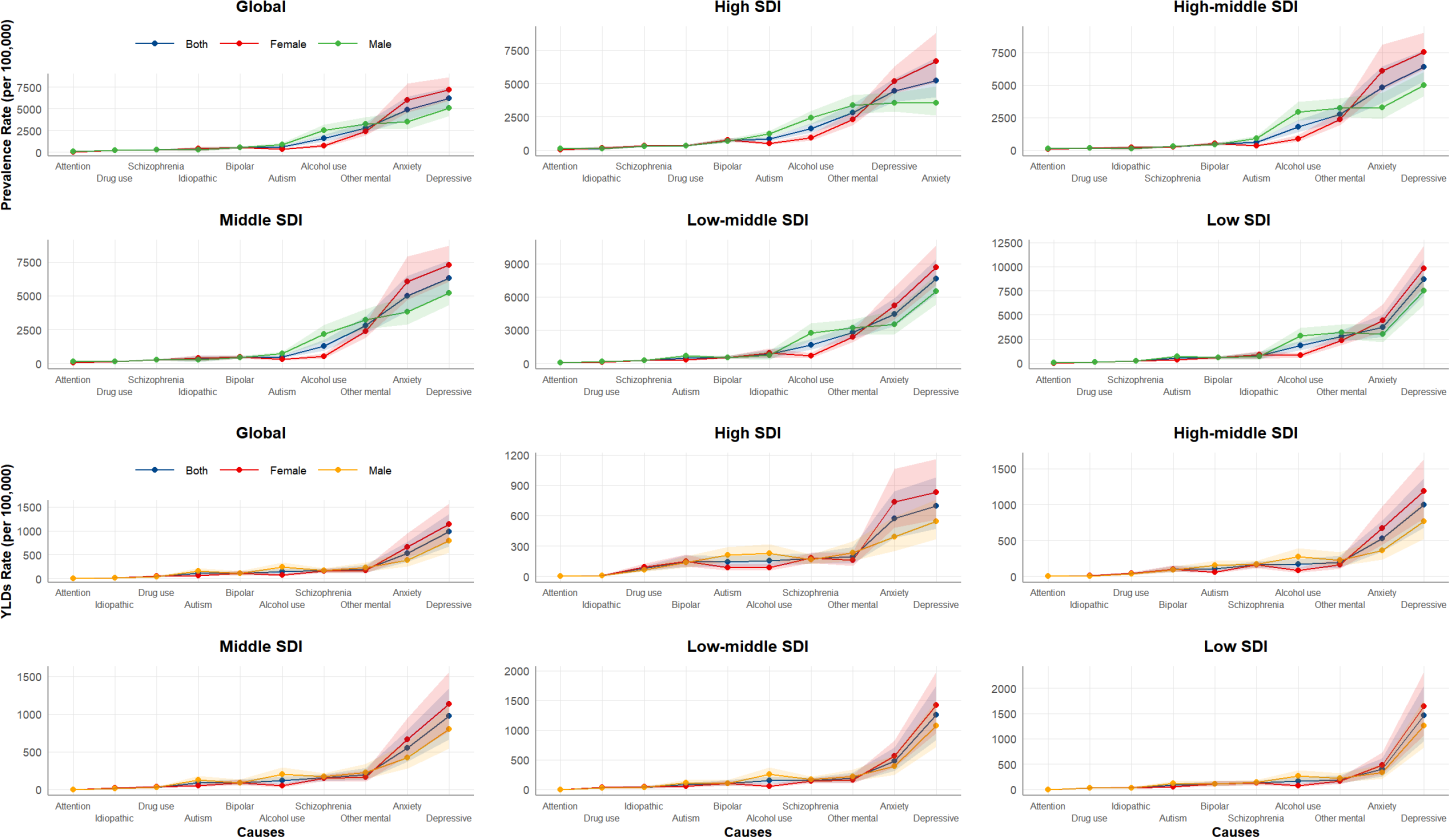
Prevalence and YLDs rate of level 3 causes among adults aged ≥60 years, 2021.

Globally in 2021, the population aged ≥60 years reached 1.09 billion, with 161.3 million (14.8%) experiencing at least one mental disorder and 19.2 million (1.8%) affected by substance use disorders. Between 1990 and 2021, the age standardized prevalence of mental disorders in this age group increased by 3.7% (from 14 274.0 to 14 800.9 per 100–000 population), with an average annual trend of 0.13%. In contrast, SUDs prevalence showed an 8.7% decline (from 1 924.7 to 1 757.2 per 100–000 population), corresponding to an average annual decrease of 0.3%. For level 3 causes, the highest prevalence was observed for depressive disorders (7 162.9 [95% UI:5 905.5-8 601.7]), while the lowest was recorded for attention-deficit/hyperactivity disorder (75.0 [95% UI: 39.0-122.1]) (**Table 1**, **Figure 1**).

**Table 1:** Age standardized prevalence and AAPC of mental disorders and substance use disorders in adults aged ≥60 years at global and regional level, from 1990 to 2021.

Cause	Rate (95% UI/95%CI)																	
	Global			High SDI			High-middle SDI			Middle SDI			Low-middle SDI			Low SDI		
	1990	2021	AAPC,%	1990	2021	AAPC,%	1990	2021	AAPC,%	1990	2021	AAPC,%	1990	2021	AAPC,%	1990	2021	AAPC,%
Mental disorders																		
Male	12783.2 (11473 ,14261)	13217.9 (11856.1 ,14771.2)	0.11 (0.11,0.12)	11828.4 (10625.3 ,13296.2)	12179.9 (10928.4 ,13675.9)	0.11 (0.09,0.12)	12329 (11056.9 ,13769.1)	12719.4 (11357.9 ,14205.3)	0.09 (0.08,0.10)	12730.6 (11416.8 ,14190.8)	13398.5 (12022.4 ,14961.6)	0.17 (0.16,0.17)	14488.6 (12931 ,16250.2)	14667.7 (13056.6 ,16499.7)	0.05 (0.04,0.08)	14919.4 (13193.8 ,16969.8)	15082.2 (13312.9 ,17118.1)	0.06 (0.04,0.07)
Female	15462.3 (13754.9 ,17536.6)	16168.4 (14343.5 ,18365.7)	0.16 (0.14,0.18)	14358.6 (12698.8 ,16423.5)	15048.7 (13306.1 ,17311.6)	0.17 (0.15,0.20)	15908.7 (14126.7 ,18028.1)	16365.9 (14487.2 ,18627.8)	0.09 (0.08,0.11)	15295 (13609.7 ,17305.7)	16229.7 (14426.5 ,18341.3)	0.20 (0.17,0.22)	16825 (14913.5 ,19091.5)	17220 (15236.5 ,19533)	0.07 (0.04,0.10)	17316.2 (15124.8 ,19856.9)	17524.2 (15242.7 ,20194.2)	0.04 (0.02,0.06)
Both	14274 (12758.3 ,16039.5)	14800.9 (13202.3 ,16645.1)	0.13 (0.11,0.15)	13300.6 (11870.8 ,15016)	13712.6 (12221.2 ,15593)	0.12 (0.10,0.14)	14449.4 (12896.9 ,16277.7)	14738.9 (13110.9 ,16599.6)	0.06 (0.06,0.07)	14093.6 (12597.2 ,15809)	14904.1 (13323.4 ,16722.2)	0.18 (0.16,0.20)	15646.8 (13909 ,17654.9)	16011 (14217.2 ,18087.6)	0.09 (0.07,0.12)	16103.1 (14142.2 ,18385.6)	16342.1 (14333.1 ,18691.8)	0.06 (0.04,0.09)
Schizophrenia																		
Male	268.1 (225.9 ,315.8)	280.1 (236.5 ,329.8)	0.14 (0.14,0.15)	282.1 (237.9 ,333.5)	279.7 (236.6 ,330.1)	-0.03 (-0.04,-0.03)	260.4 (222.9 ,303.1)	283.4 (243.6 ,327.7)	0.28 (0.27,0.29)	272.8 (229.9 ,322.3)	284.3 (239.1 ,336)	0.14 (0.13,0.14)	267 (219 ,321.8)	284.8 (235.2 ,342.2)	0.21 (0.21,0.21)	221.2 (179.7 ,269.5)	236.4 (192.8 ,287.3)	0.22 (0.21,0.22)
Female	260.4 (220 ,305.7)	268 (226.1 ,315.3)	0.09 (0.09,0.10)	306.3 (257.3 ,360)	309.8 (261.2 ,364.2)	0.03 (0.02,0.04)	244.8 (210.5 ,284.4)	266.1 (229.1 ,307.6)	0.28 (0.27,0.28)	247.9 (209.4 ,291.1)	256.4 (216.1 ,302.8)	0.11 (0.10,0.11)	229.2 (189.3 ,275.3)	246.2 (203.9 ,294.8)	0.23 (0.23,0.24)	212 (172.7 ,259.7)	223.9 (182.9 ,273.1)	0.18 (0.17,0.18)
Both	264.4 (223.2 ,311.2)	274 (231.3 ,322.2)	0.12 (0.11,0.12)	296.4 (249.6 ,348.9)	295.9 (250.4 ,348.2)	-0.004 (-0.01,0.002)	251.7 (216.1 ,292.5)	274.3 (236.1 ,317.1)	0.28 (0.28,0.29)	260.1 (219.5 ,306.2)	269.9 (226.9 ,318.9)	0.12 (0.12,0.12)	248.4 (204.7 ,299.1)	264.7 (219 ,317.3)	0.21 (0.20,0.21)	216.8 (176.5 ,264.7)	230 (188.1 ,280)	0.19 (0.19,0.20)
Depressive disorders																		
Male	4785.1 (3952.5 ,5773.3)	5071 (4180.6 ,6123.2)	0.18 (0.17,0.19)	3463.9 (2880.3 ,4156.4)	3544.5 (2906.5 ,4323.2)	0.09 (0.07,0.11)	4722.9 (3913.3 ,5677.6)	4991.1 (4142 ,6008.5)	0.15 (0.12,0.18)	4747.1 (3918.7 ,5705.7)	5219.6 (4318.4 ,6294.7)	0.30 (0.29,0.31)	6301.3 (5151.1 ,7687.3)	6517.9 (5300.2 ,8026.1)	0.13 (0.10,0.16)	7490.1 (6013.2 ,9292.5)	7496.5 (6008.3 ,9309.9)	0.02 (-0.01,0.04)
Female	6731.9 (5589.1 ,8071.3)	7162.9 (5905.5 ,8601.7)	0.21 (0.17,0.25)	4990.8 (4180.9 ,5936.9)	5192.2 (4264.9 ,6295.1)	0.15 (0.11,0.19)	7307.7 (6070.4 ,8789.7)	7522.2 (6213 ,9035.1)	0.08 (0.05,0.11)	6766.7 (5619.1 ,8120.6)	7286.8 (6054.2 ,8703.6)	0.25 (0.22,0.29)	8482.9 (6933.7 ,10348.9)	8686.7 (7094.2 ,10637.6)	0.09 (0.05,0.14)	9907.6 (7917.1 ,12242.1)	9826.8 (7840.6 ,12175.8)	-0.02 (-0.05,0.02)
Both	5859.1 (4848.3 ,7046.1)	6189.2 (5112.2 ,7451.9)	0.19 (0.17,0.22)	4349.2 (3633.5 ,5182.4)	4424.9 (3632.2 ,5372.3)	0.08 (0.04,0.11)	6247.7 (5195.8 ,7518.9)	6387.9 (5287.6 ,7667.7)	0.07 (0.06,0.09)	5812.3 (4821.5 ,6993.3)	6315.1 (5244.7 ,7594.8)	0.28 (0.26,0.30)	7380 (6039.1 ,8985.4)	7655.7 (6254.7 ,9386)	0.14 (0.10,0.17)	8680.8 (6954.2 ,10723.2)	8697.2 (6953.9 ,10771.8)	0.004 (-0.02,0.03)
Bipolar disorder																		
Male	548.5 (428.2 ,693.5)	523.2 (408.7 ,660.4)	-0.15 (-0.16,-0.15)	699 (559.8 ,863.4)	671.7 (541 ,826.2)	-0.13 (-0.13,-0.13)	513.4 (394 ,660.2)	450.7 (346.2 ,579.3)	-0.42 (-0.42,-0.41)	433 (334.7 ,551.4)	448.8 (347.3 ,571.3)	0.12 (0.11,0.12)	523 (401.5 ,667.3)	528.8 (405.4 ,676.1)	0.04 (0.04,0.04)	566.2 (428.8 ,729.9)	558.4 (423 ,719.1)	-0.04 (-0.05,-0.04)
Female	604 (472.9 ,759.4)	559.9 (439 ,703.9)	-0.25 (-0.25,-0.24)	788.9 (633 ,972.1)	749 (605.3 ,917.3)	-0.17 (-0.17,-0.16)	583.7 (449.4 ,742.1)	510.4 (392.7 ,649.9)	-0.43 (-0.44,-0.43)	440.4 (341.5 ,559.5)	462.7 (359.2 ,587.5)	0.16 (0.15,0.16)	521.8 (400 ,666.3)	527.5 (404.4 ,673.8)	0.04 (0.03,0.04)	572.3 (431.1 ,742.9)	563.5 (424.4 ,731.3)	-0.05 (-0.05,-0.05)
Both	578.9 (453 ,729.8)	542.6 (425.3 ,683.6)	-0.21 (-0.21,-0.21)	750 (600.8 ,923.8)	712.5 (575.2 ,874.1)	-0.17 (-0.17,-0.16)	553.5 (425.2 ,706.7)	482.9 (370.6 ,617.3)	-0.44 (-0.44,-0.43)	436.2 (337.9 ,554.7)	455.8 (353.6 ,579.2)	0.14 (0.14,0.15)	522.4 (400.2 ,666.8)	528.2 (404.4 ,674.2)	0.04 (0.04,0.04)	569.2 (430 ,736.8)	561 (424.2 ,725.8)	-0.05 (-0.05,-0.05)
Anxiety disorders																		
Male	3257.8 (2415.5 ,4387.7)	3524.8 (2624 ,4703.8)	0.27 (0.24,0.30)	3269.9 (2408.3 ,4431.7)	3538.3 (2595.2 ,4766.4)	0.29 (0.25,0.33)	3061.7 (2262.1 ,4152.5)	3258.7 (2397.9 ,4404.1)	0.21 (0.19,0.23)	3526.1 (2658.7 ,4631.1)	3809.5 (2868.6 ,5000.9)	0.26 (0.24,0.29)	3260.4 (2400.9 ,4406.6)	3538.5 (2619 ,4754.9)	0.30 (0.26,0.35)	2824.9 (2048.1 ,3883.2)	2999.8 (2176.2 ,4066.5)	0.22 (0.18,0.26)
Female	5556.9 (4236.5 ,7365.1)	5992.1 (4573 ,7922.2)	0.26 (0.23,0.29)	6085.9 (4618.6 ,8040.8)	6694.8 (5087.8 ,8835.3)	0.34 (0.29,0.39)	5667.2 (4319.3 ,7525)	6070.8 (4583.6 ,8101.4)	0.23 (0.21,0.24)	5489.4 (4221.5 ,7228.2)	6068.9 (4700 ,7896.9)	0.33 (0.30,0.36)	4713.3 (3538.8 ,6300)	5210 (3968.1 ,6927.1)	0.36 (0.31,0.43)	4159.7 (3045 ,5754.1)	4407.6 (3227 ,6080.9)	0.22 (0.18,0.26)
Both	4541.9 (3467.1 ,6014.5)	4852.4 (3707.2 ,6415)	0.23 (0.21,0.26)	4901.4 (3734.3 ,6489.6)	5224.9 (3952.4 ,6924.3)	0.24 (0.19,0.28)	4602.3 (3509.1 ,6094.1)	4817.1 (3630.7 ,6436.7)	0.15 (0.13,0.16)	4574.5 (3521.8 ,5985.9)	5013.8 (3872.9 ,6491.4)	0.31 (0.28,0.33)	3983.8 (3002.4 ,5317.1)	4422.8 (3359.7 ,5895)	0.38 (0.33,0.43)	3485.7 (2565.2 ,4761.1)	3727.7 (2741.9 ,5048)	0.25 (0.21,0.28)
Autism spectrum disorders																		
Male	804.9 (660.5 ,956)	873.4 (716.8 ,1041.6)	0.26 (0.26,0.27)	1087.1 (896.5 ,1293.4)	1205.9 (991.4 ,1438.5)	0.34 (0.33,0.34)	833.7 (683.6 ,996.2)	901.5 (740.5 ,1079.4)	0.25 (0.25,0.26)	640.6 (521.4 ,765)	732.8 (599.1 ,874.5)	0.43 (0.43,0.44)	598.4 (486 ,715.7)	649.5 (530.4 ,772.9)	0.26 (0.26,0.27)	652.8 (528.3 ,782.2)	696.9 (566.5 ,830.7)	0.21 (0.21,0.21)
Female	353.3 (287.2 ,422.9)	361.3 (293.9 ,432.6)	0.07 (0.07,0.08)	445.3 (364.6 ,533.3)	489.1 (400.9 ,585.5)	0.30 (0.30,0.31)	358.2 (291.8 ,430.6)	347.6 (282.7 ,418)	-0.10 (-0.10,-0.09)	269 (216.7 ,325.3)	297 (240 ,357.1)	0.32 (0.32,0.33)	294.6 (237.6 ,355.2)	312.9 (253 ,376.1)	0.19 (0.19,0.20)	323.7 (260.6 ,391.3)	340.5 (276.1 ,411.1)	0.16 (0.16,0.16)
Both	557.1 (455.9 ,662.1)	599.6 (491.3 ,714)	0.24 (0.23,0.24)	720.2 (593.4 ,857.9)	823.4 (676.9 ,983)	0.43 (0.43,0.44)	557.2 (456.1 ,664.3)	597.2 (490 ,714.2)	0.22 (0.22,0.23)	445.7 (361.9 ,531.8)	502.5 (410.2 ,599.5)	0.39 (0.39,0.39)	448.4 (363.8 ,536.3)	472.9 (386 ,563.5)	0.17 (0.17,0.17)	492 (398.1 ,590.3)	514.1 (417.5 ,613.5)	0.14 (0.14,0.15)
Attention-deficit/hyperactivity disorder																		
Male	105.5 (56 ,171.5)	104.3 (54.3 ,170)	-0.04 (-0.05,-0.03)	85.5 (43.9 ,141.6)	90.8 (45.3 ,151.8)	0.20 (0.19,0.21)	130.6 (70.7 ,211.1)	133.3 (71.4 ,216.6)	0.07 (0.06,0.08)	144.7 (80.1 ,230.9)	127 (67.2 ,207.1)	-0.42 (-0.43,-0.41)	58.9 (29.1 ,98.3)	64.1 (32.9 ,105.5)	0.28 (0.27,0.29)	40.7 (19.2 ,70.8)	39 (18.1 ,69.1)	-0.14 (-0.14,-0.13)
Female	47.8 (23.5 ,80.6)	47.7 (23.7 ,81.1)	-0.01 (-0.03,0.00)	38 (17.4 ,67.2)	41.3 (19.2 ,72.3)	0.27 (0.24,0.29)	57.2 (29.2 ,93.9)	60.4 (30.7 ,100.5)	0.18 (0.16,0.20)	64.5 (32.7 ,106.3)	57.3 (29 ,96.4)	-0.39 (-0.40,-0.38)	29.9 (14 ,51.9)	30.7 (14.7 ,53.1)	0.08 (0.07,0.09)	19.3 (8.2 ,35.4)	18.7 (7.8 ,34.3)	-0.12 (-0.13,-0.10)
Both	75.4 (39.3 ,123)	75 (39 ,122.1)	-0.02 (-0.03,-0.01)	59.8 (30.2 ,100.5)	65.3 (32.4 ,110.1)	0.28 (0.26,0.30)	90.2 (47.5 ,146.3)	94.8 (50.3 ,153.9)	0.15 (0.14,0.17)	104.2 (55.9 ,168.4)	91.1 (48 ,148.4)	-0.44 (-0.45,-0.43)	44.8 (22 ,75.5)	46.8 (23.5 ,78)	0.14 (0.13,0.14)	30.5 (14 ,53.9)	28.7 (12.9 ,51.2)	-0.19 (-0.20,-0.18)
Idiopathic developmental intellectual disability																		
Male	423.4 (172 ,725.3)	296.3 (109.5 ,526.3)	-1.12 (-1.14,-1.10)	154.1 (25.6 ,336.7)	97.5 (13.8 ,241.2)	-1.47 (-1.50,-1.46)	199.8 (49.1 ,401.7)	127 (23.7 ,282.3)	-1.45 (-1.47,-1.44)	403.6 (159.7 ,694.9)	275.3 (87.2 ,493.5)	-1.21 (-1.22,-1.19)	1114.4 (537.1 ,1724)	726.3 (330.6 ,1150)	-1.35 (-1.37,-1.33)	763.8 (365.3 ,1202)	715 (347.1 ,1126.5)	-0.19 (-0.22,-0.16)
Female	459.3 (215.3 ,719)	391.1 (180.7 ,608.5)	-0.52 (-0.53,-0.50)	206.1 (53.6 ,365.7)	143.1 (29.2 ,264.7)	-1.18 (-1.20,-1.17)	256.7 (87.1 ,433.7)	197.8 (52.4 ,340.4)	-0.84 (-0.85,-0.83)	503.6 (253.3 ,770.1)	386.2 (173.7 ,600.1)	-0.86 (-0.87,-0.85)	1226.1 (664 ,1817)	912.3 (512.1 ,1341)	-0.95 (-0.96,-0.94)	790.1 (413.1 ,1201.4)	854.6 (471.7 ,1263.9)	0.26 (0.24,0.27)
Both	442.7 (198 ,721.2)	346.5 (147.5 ,570.6)	-0.78 (-0.79,-0.77)	184.5 (43.8 ,354.9)	122 (23.6 ,250.2)	-1.34 (-1.36,-1.32)	233.1 (70.3 ,423.5)	166.1 (38.9 ,314.8)	-1.09 (-1.10,-1.08)	455.7 (209.6 ,734.1)	333.5 (132.4 ,548.3)	-1.00 (-1.02,-0.99)	1169.3 (601.1 ,1773.2)	823.9 (428.2 ,1249.8)	-1.12 (-1.14,-1.11)	776.4 (390.1 ,1197.3)	786.3 (412.5 ,1197.7)	0.05 (0.03,0.07)
Other mental disorders																		
Male	3253.3 (2623.1 ,4027.9)	3249.7 (2615.8 ,4029.4)	-0.0035 (-0.0037,-0.0033)	3366.9 (2744.9 ,4129.7)	3370.1 (2747 ,4136.6)	0.0033 (0.0031,0.0036)	3214.4 (2578.7 ,3986.8)	3217.8 (2577.7 ,3998.9)	0.0034 (0.0032,0.0035)	3217.8 (2565.9 ,4031)	3220.2 (2568.5 ,4032.8)	0.0024 (0.0023,0.0025)	3209.3 (2566.5 ,4009.9)	3208.1 (2567 ,4005.5)	-0.0013 (-0.0014,-0.0012)	3193.2 (2555.4 ,3988)	3197.5 (2558.2 ,3991.6)	0.0042 (0.0041,0.0043)
Female	2355.4 (1896.8 ,2917.2)	2366.9 (1903.8 ,2931.7)	0.0157 (0.0156,0.0158)	2302.2 (1859 ,2842.9)	2309.2 (1867.2 ,2849.7)	0.0097 (0.0096,0.0098)	2374 (1905.1 ,2945.6)	2379.5 (1908.6 ,2949.4)	0.0074 (0.0073,0.0076)	2399.7 (1919.3 ,2975.7)	2401.9 (1921.3 ,2979.5)	0.0030 (0.0029,0.0031)	2387.5 (1915.7 ,2965.3)	2388.4 (1917.4 ,2966.5)	0.0012 (0.0011,0.0014)	2366.4 (1903.1 ,2950.9)	2368.1 (1904.2 ,2952.7)	0.0022 (0.0020,0023)
Both	2752.9 (2221.7 ,3402.9)	2774.5 (2238.6 ,3434)	0.0252 (0.0249,0.0254)	2749.5 (2228.6 ,3379.8)	2801.3 (2274.6 ,3443.3)	0.0603 (0.0600,0.0606)	2718.6 (2186.6 ,3370.4)	2753.4 (2213.2 ,3416.5)	0.0411 (0.0408,0.0413)	2782.3 (2232.1 ,3469.1)	2784 (2232.7 ,3471.8)	0.0020 (0.0017,0.0022)	2800.7 (2250.5 ,3489.4)	2774.8 (2231.1 ,3454.7)	-0.0299 (-0.0304,-0.0294)	2784.7 (2239.7 ,3468.4)	2769 (2228 ,3448)	-0.018 (-0.019,-0.017)
Substance use disorders																		
Male	2869 (2248.7 ,3629.2)	2702.3 (2132.1 ,3395.8)	-0.21 (-0.23,-0.18)	2521.3 (2059.3 ,3039.4)	2735.8 (2265.5 ,3257.4)	0.28 (0.25,0.30)	3783.3 (3001.7 ,4700.9)	3083.1 (2434.4 ,3868.2)	-0.70 (-0.73,-0.66)	2225 (1670.4 ,2923.5)	2301.4 (1741.2 ,3002)	0.09 (0.04,0.13)	3114.1 (2306.1 ,4110.8)	2887.4 (2191.4 ,3749.6)	-0.29 (-0.33,-0.26)	3022.3 (2278.7 ,3935.4)	2969.9 (2295 ,3775.6)	-0.10 (-0.18,-0.05)
Female	1127.9 (869.3 ,1444.2)	924.4 (724.5 ,1171.9)	-0.62 (-0.65,-0.58)	1106.6 (866.9 ,1396.2)	1275.3 (1047.9 ,1548.9)	0.50 (0.44,0.54)	1679.1 (1297.9 ,2156.6)	1035.2 (800.9 ,1325.2)	-1.54 (-1.63,-1.46)	765.6 (584.9 ,987.4)	666 (493.6 ,877.5)	-0.48 (-0.51,-0.45)	806.7 (593.6 ,1069.7)	789.8 (589.7 ,1039.8)	-0.07 (-0.08,-0.07)	946.4 (705.6 ,1232.3)	922.1 (704.6 ,1177.9)	-0.11 (-0.16,-0.06)
Both	1924.7 (1509.1 ,2443.1)	1757.2 (1391.3 ,2209.9)	-0.30 (-0.31,-0.29)	1725.9 (1400.8 ,2117)	1964.2 (1635.1 ,2347.7)	0.45 (0.41,0.48)	2574.8 (2035.3 ,3235)	1965.2 (1549.5 ,2479.3)	-0.88 (-0.91,-0.85)	1458.6 (1099.5 ,1904.5)	1435.1 (1084.7 ,1874)	-0.07 (-0.11,-0.03)	1974.7 (1464.3 ,2609.7)	1784.2 (1353.9 ,2309.1)	-0.37 (-0.39,-0.34)	2021.4 (1528.2 ,2612.7)	1926.1 (1492.7 ,2441.8)	-0.15 (-0.20,-0.09)
Alcohol use disorders																		
Male	2677.3 (2052.6 ,3432.1)	2514.2 (1938.6 ,3197.8)	-0.21 (-0.23,-0.18)	2285.8 (1830.2 ,2805)	2414.8 (1956.2 ,2925.2)	0.19 (0.17,0.22)	3581.2 (2798.3 ,4498.3)	2927.3 (2264 ,3702.7)	-0.69 (-0.73,-0.66)	2035.9 (1481.3 ,2731.5)	2151.7 (1584.4 ,2855.9)	0.15 (0.11,0.20)	2981.9 (2174.8 ,3978.4)	2749.4 (2052.6 ,3609.9)	-0.31 (-0.34,-0.27)	2897.8 (2151.6 ,3816)	2838.9 (2162.7 ,3642.6)	-0.12 (-0.19,-0.06)
Female	938 (684.6 ,1249.7)	737 (538.8 ,981.4)	-0.77 (-0.80,-0.73)	908.7 (667.5 ,1190.6)	935.5 (715.5 ,1205)	0.15 (0.10,0.20)	1473.8 (1093.9 ,1946.3)	873.7 (639.6 ,1160.5)	-1.67 (-1.78,-1.57)	550 (378.1 ,768.5)	524.5 (358.6 ,731.5)	-0.19 (-0.23,-0.16)	676.5 (467.9 ,943)	662 (465.3 ,906.7)	-0.07 (-0.09,-0.06)	847.7 (610.9 ,1134.9)	825.9 (609.9 ,1081.6)	-0.11 (-0.17,-0.06)
Both	1733.1 (1314.8 ,2249.5)	1569 (1199.6 ,2017.2)	-0.33 (-0.34, -0.32)	1511 (1182.3 ,1892.7)	1633.2 (1308.7 ,2004)	0.28 (0.24,0.32)	2370 (1819.1 ,3026)	1805.7 (1385 ,2309.9)	-0.87 (-0.90,-0.85)	1254.5 (903.6 ,1691.6)	1289.3 (939.3 ,1727.5)	0.06 (0.02,0.11)	1843.2 (1332.5 ,2477.1)	1651.3 (1224.7 ,2176.9)	-0.40 (-0.43,-0.37)	1909.4 (1418.8 ,2504.7)	1813 (1379.9 ,2328.4)	-0.16 (-0.22,-0.10)
Drug use disorders																		
Male	197.9 (160.7 ,241.2)	193.7 (160.1 ,232.4)	-0.08 (-0.09,-0.07)	241.5 (196.1 ,294.8)	329.9 (279.2 ,390.2)	1.01 (0.99,1.03)	211.8 (173 ,255.6)	162 (131.8 ,195.3)	-0.87 (-0.89,-0.85)	193.4 (156.4 ,236.9)	153.7 (123.9 ,188.4)	-0.74 (-0.75,-0.72)	137 (106.7 ,176)	142.4 (113.1 ,177.6)	0.12 (0.08,0.14)	128.7 (101.2 ,163.4)	135.1 (107.9 ,167.9)	0.15 (0.12,0.16)
Female	191.7 (155.7 ,234.8)	189.1 (157 ,225.4)	-0.03 (-0.05,-0.01)	199.7 (163 ,241.7)	343.6 (293.5 ,404.6)	1.77 (1.70,1.83)	208.5 (168 ,257.2)	163.1 (131.2 ,199.9)	-0.79 (-0.80,-0.78)	216.7 (172.7 ,270.1)	142.2 (113.3 ,175.5)	-1.36 (-1.38,-1.34)	131.1 (100.7 ,167.9)	128.8 (100.6 ,161.8)	-0.06 (-0.07,-0.06)	99.4 (74.8 ,129)	96.9 (74.2 ,123.9)	-0.08 (-0.09,-0.08)
Both	195.4 (159.6 ,237.6)	191.7 (159.7 ,228.8)	-0.06 (-0.07,-0.05)	218.6 (179 ,265.4)	337.3 (288 ,394.5)	1.43 (1.40,1.45)	210.9 (171.1 ,256.7)	163.3 (132.3 ,196.9)	-0.82 (-0.84,-0.81)	206.8 (166.8 ,253.9)	148.1 (119.2 ,182.1)	-1.07 (-1.08,-1.06)	134.4 (104.7 ,170.4)	135.5 (107.7 ,168.3)	0.02 (-0.00,0.03)	114.5 (88.8 ,145.3)	115.5 (91 ,144)	0.03 (0.01,0.04)
Self-harm																		
Male	544.7 (472.2 ,619.2)	478.1 (412.6 ,545.5)	-0.43 (-0.47,-0.41)	692.7 (593.7 ,792.4)	704.4 (601.6 ,811.6)	0.05 (0.02,0.06)	754.6 (653.8 ,860.4)	557.1 (480.5 ,636.1)	-1.00 (-1.03,-0.97)	370.9 (322.6 ,421)	333.7 (289.3 ,379.6)	-0.36 (-0.41,-0.33)	337.2 (293.1 ,383.8)	335.7 (292.5 ,379.7)	-0.03 (-0.08,-0.01)	265.4 (231.2 ,300.2)	303.7 (263.6 ,343.4)	0.42 (0.37,0.45)
Female	653.2 (560.1 ,751.6)	491.1 (421.8 ,563.1)	-0.96 (-1.03,-0.92)	568.3 (488 ,652.3)	613.5 (524.5 ,706.8)	0.22 (0.15,0.27)	726.2 (622.4 ,833)	462 (396.3 ,530.2)	-1.51 (-1.58,-1.46)	725.1 (619.7 ,837.8)	400.3 (344.6 ,458.2)	-1.95 (-2.02,-1.89)	656.1 (546.2 ,797.3)	548.4 (466.8 ,636.9)	-0.61 (-0.65,-0.58)	373.4 (320.1 ,430.5)	376.3 (323.7 ,430.9)	-0.02 (-0.17,0.09)
Both	604.2 (520.6 ,689.9)	485.1 (418 ,554.2)	-0.74 (-0.79,-0.71)	619.5 (531.7 ,709.9)	655.1 (560.4 ,753.3)	0.17 (0.12,0.20)	738.3 (636.2 ,844.3)	505.2 (434.4 ,578.4)	-1.24 (-1.29,-1.21)	559.4 (480.9 ,642.6)	369.9 (319.5 ,422.4)	-1.37 (-1.43,-1.32)	495.3 (419.7 ,583.5)	448.3 (385.3 ,514.5)	-0.35 (-0.38,-0.32)	318.7 (275.8 ,363)	341.3 (295.4 ,388.7)	0.21 (0.11,0.28)

SDI, socio demographic index; AAPC, average annual percentage changes; 95% CI, 95% confidence intervals(AAPC); 95% UI, 95% uncertainty interval.

The age standardized YLDs of mental disorders increased by 4.5% (from 2 003.4 to 2 095.2 per 100–000 population) between 1990 and 2021, with an annualized increase of 0.15%, whereas SUDs decreased by 8.2% (from 206.1 to 189.2 per 100–000 population), showing an annualized decline of 0.28%. For level 3 causes, depressive disorders were the leading contributors (980.4 [95% UI: 667.2-1 352.4]) and the lowest was observed for idiopathic developmental intellectual disability disorder (15.9 [95% UI: 6.8-28.0]) (**Figure 1**, **eTable 2**, **Supplementary 1**).

### 3.2 Global trends by sociodemographic index

Between 1990 and 2021, the age standardized prevalence of mental disorders among people aged ≥60 years increased across all SDI subgroups, particularly in middle SDI region where rates rose from 14,093.6 to 14 904.1 per 100–000 population (AAPC 0.18%). The prevalence of SUDs showed divergent trends, increasing in high SDI regions (AAPC 0.45%) while declining in all other SDI regions, most markedly in high-middle SDI regions (AAPC -0.88%). For level 3 causes in 2021, the prevalence rankings were largely similar, with depression predominating in most SDI regions. Collectively, the prevalence rates of all level 3 causes were significantly higher in low SDI regions compared to other SDI subgroups (**Table 1**, **Figure 1**).

Age standardized YLDs for mental disorders among persons aged ≥60 years increased across all SDI regions between 1990 and 2021. Similar to prevalence patterns, the fastest rate of increase occurred in the middle SDI region (AAPC 0.27%). For substance use disorders, all SDI regions exhibited declining trends, except high SDI region (AAPC 0.81%) and the most rapid decline occurred in high-middle SDI region (AAPC -0.93%). In 2021, the distribution of level 3 causes showed notable consistency across SDI regions, with depression consistently ranking first in YLDs, followed by anxiety. Overall, lower SDI levels correlated with higher YLDs burdens (**Figure 1**, **eTable 2**, **Supplementary 1**).

### 3.3 Global trends by sex

From 1990 to 2021, the age standardized prevalence rate of mental disorders among people aged 60 and older globally increased in both males (12 783.2 to 13 217.9 per 100–000 population) and females (15 462.3 to 16 168.4 per 100–000 population), with females showing a faster rate of increase (AAPC 0.16% vs.0.11%). Conversely, the prevalence of SUDs decreased in both males (2 869.0 to 2 702.3 per 100 000) and females (1 127.9 to 924.4 per 100 000), with a greater reduction observed in females (AAPC -0.62% vs.-0.21%). Among level 3 causes, depressive disorders had the highest prevalence in both sexes, whereas attention-deficit/hyperactivity disorder showed the lowest prevalence (**Table 1**, **Figure 1**).

The age standardized YLDs for mental disorders increased in both males and females from 1990 to 2021, with nearly identical rates of increase (AAPC 0.17%). YLDs for SUDs declined in both sexes, with a faster decline in females (AAPC -0.49% vs.-0.21%). Regarding level 3 causes, depressive disorders were the leading contributor to YLDs in both sexes, whereas attention-deficit/hyperactivity disorder accounted for the smallest proportion (**Figure 1**, **eTable 2**, **Supplementary 1**).

### 3.4 Global trends by age subgroup

From 1990 to 2021, the prevalence of mental disorders increased per 100–000 population in the following age subgroups: 60–64 years (from 14 894.6 to 15 707.3), 65–69 years (from 14 591.1 to 15 186.8), 70–74 years (from 14 147.8 to 14 640.4), 75–79 years (from 13 763.7 to 14 138.0), and 80–84 years (from 13 398.8 to 13 620.9). In contrast, prevalence declined in older subgroups: 85–89 years (from 13 120.0 to 13 097.9), 90–94 years (from 12 864.2 to 12 636.4), and ≥95 years (from 12 758.2 to 12 259.6). For substance use disorders, prevalence decreased across all age subgroups except the ≥95 years group. Level 3 causes generally showed lower prevalence in older subgroups, except for other mental disorders, alcohol use disorders, and drug use disorders (**eFigure 2**, **eTable 3**, **Supplementary 1**).

**Figure 2:**
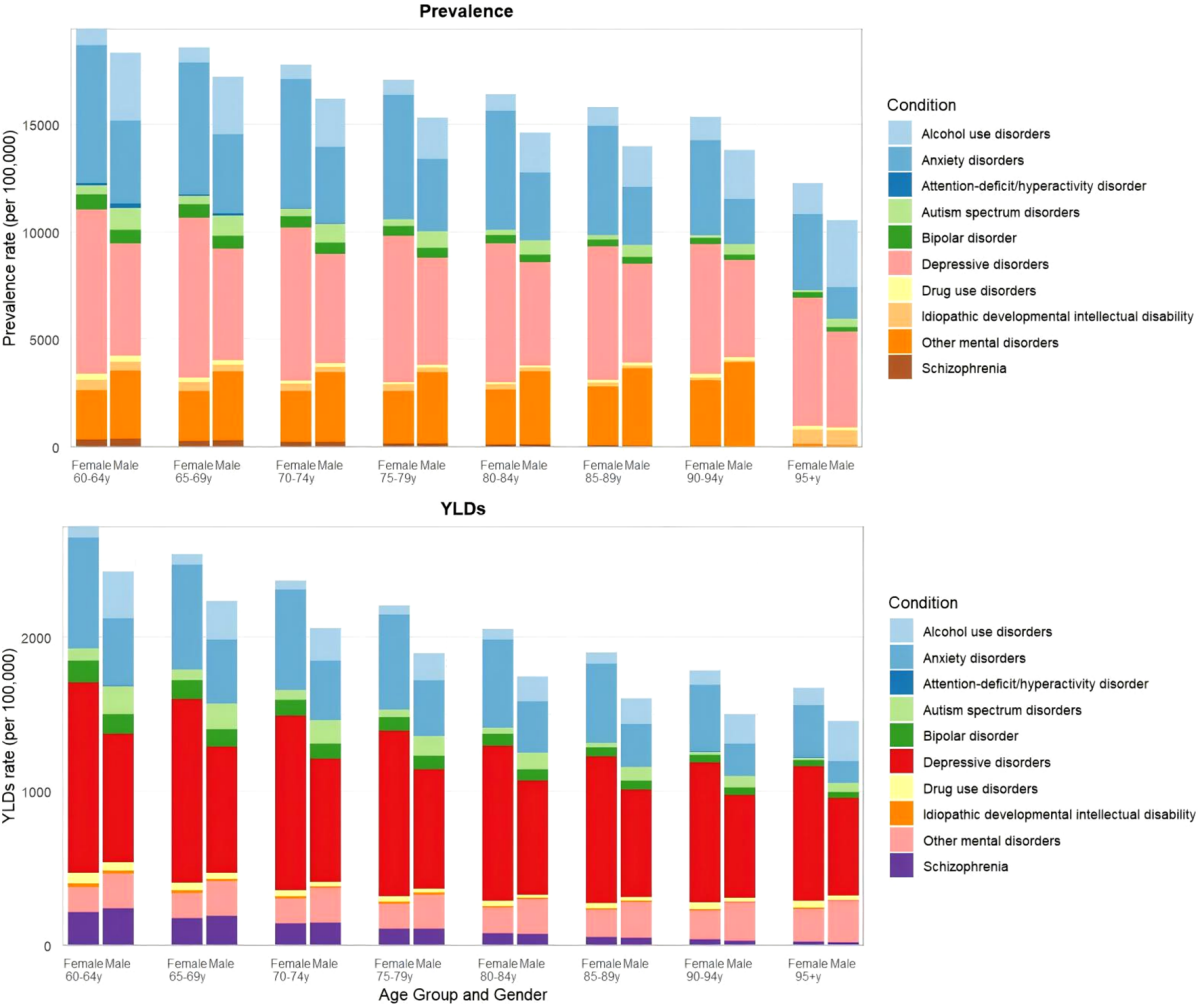
Prevalence and YLDs of Level 3 Causes by Age Group and Gender, 2021.

Globally in 2021, mental disorders were one of the leading causes of YLDs among individuals aged ≥60 years, accounting for 22.8 million YLDs, compared with 2.10 million YLDs from SUDs. The 60–64 age group exhibited higher YLDs than the ≥95 years group across level 3 causes. For depression—the leading level 3 cause of YLDs—the YLDs rate was 1.2-fold higher in the 60–64 group than in the ≥95 group (6 454.5 vs.5 547.5). Conversely, certain causes exhibited reversed patterns; notably, alcohol use disorders showed higher YLDs rates in the ≥95 group compared with the 60–64 group (2 625.3 vs.1 992.7) (**Figure 2**, **eTable 4**, **Supplementary 1**).

### 3.5 Regional trends

Between 1990 and 2021, Central Asia was the only region among 21 global regions showing declining age standardized YLDs (AAPC -0.03%) for mental disorders in populations aged ≥60 years, whereas all other regions demonstrated increasing prevalence and YLDs. For substance use disorders, nearly half of regions exhibited rising trends (**eTables 5**, **6**, **Supplementary 1**).

**Figure 3:**
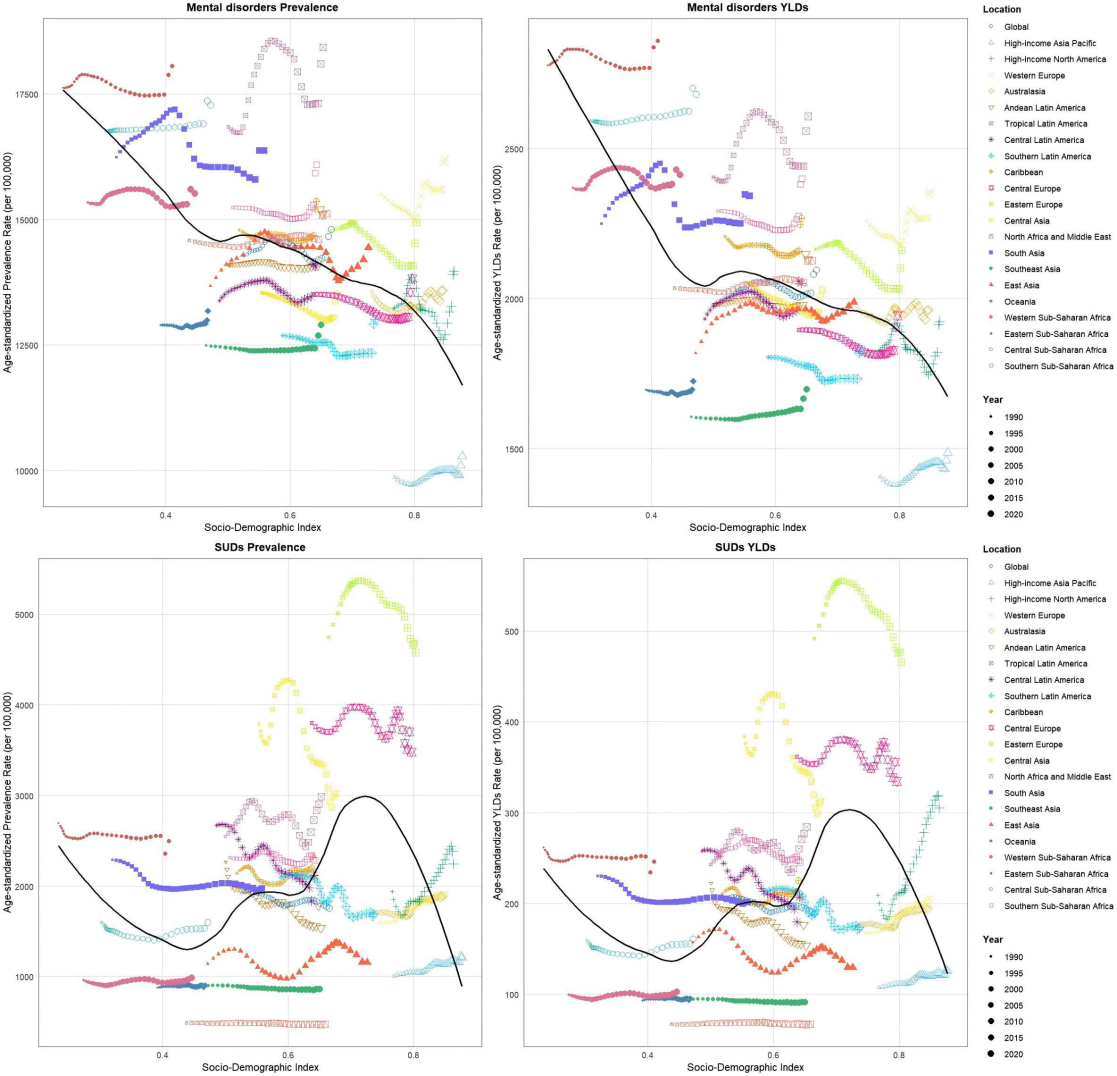
SDI correlations with: average annual percentage changes in prevalence and YLDs for mental disorders and SUDs among adults aged 60+ Years, 1990–2021.

In 2021, the prevalence and YLDs for mental disorders among adults aged ≥60 years showed an inverse association with SDI, indicating lower burden in higher-SDI regions. Tropical Latin America had the highest mental disorders prevalence (16 848.5-18 429.8 per 100 000) with sustained growth (AAPC 0.32%), while Eastern Sub-Saharan Africa showed the highest YLDs (2 777.2-2 895.8 per 100 000, AAPC 0.11%). SUDs displayed nonlinear epidemiological patterns, with prevalence and YLDs initially decreasing, then increasing, before declining again as SDI rose. Eastern Sub-Saharan Africa maintained the highest substance use disorders burden, though progressive declines were observed (**Figure 3**, **eTables 5**, **6**, **Supplementary 1**).

**Figure 4:**
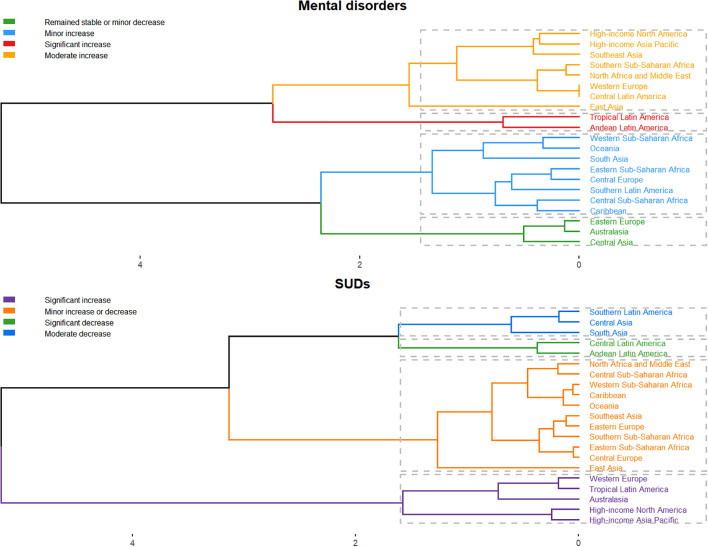
Hierarchical clustering analysis of prevalence and YLDs in 21 Regions, 2021.

To furtherly analyze regions with similar variation in disease burden, a hierarchical clustering analysis was conducted in this study. For mental disorders, significant increases in prevalence and YLD rates were observed in Western Europe, Tropical Latin America, Australasia, High-income North America, and High-income Asia Pacific. Regarding SUDs, significant growth was noted in Tropical Latin America and Andean Latin America (**Figure 4**).

### 3.6 National trends

**Figure 5:**
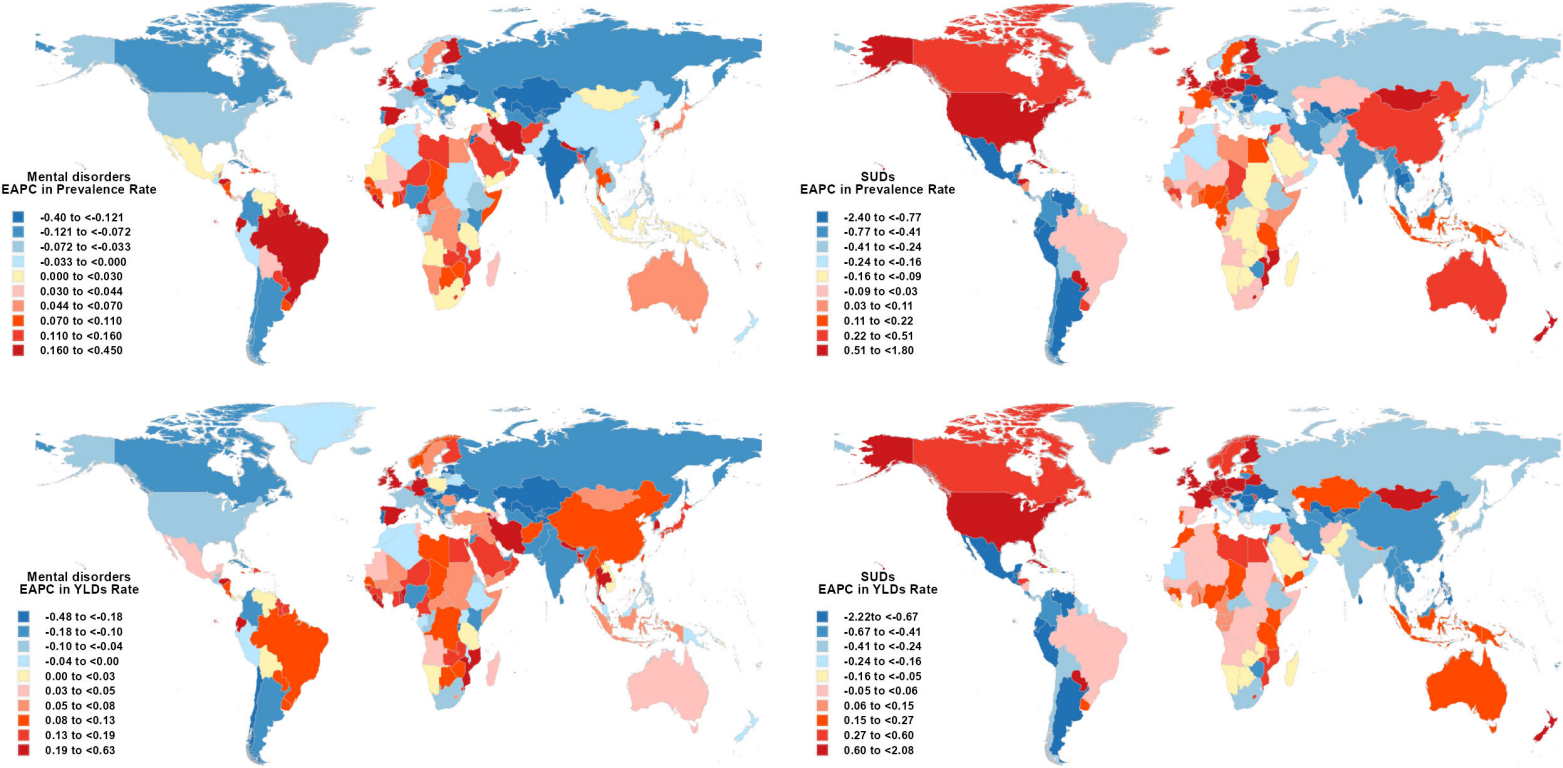
Map showing estimated percentage change in prevalence and YLDs for mental disorders and SUDs among adults aged 60+ Years, 1990–2021.

At the national level, from 1990 to 2021, Spain experienced the highest increase in age standardized prevalence of mental disorders among those aged ≥60 years, with an estimated annual trend of 0.44%, followed by Germany (EAPC 0.30%) and United Kingdom (0.25%). For substance use disorders, United Kingdom had the highest increase (EAPC 1.78%), followed by Austria (EAPC 1.76%) and Belarus (EAPC 1.52%). Over this period, Spain showed the most significant increase in age standardized YLDs for older people with mental disorders (EAPC 0.62%), while United States Virgin Islands experienced the largest increase for SUDs (**Figure 5**, **eTables 7**, **8**, **Supplementary 1**).

In 2021, Uganda had the highest age standardized prevalence of mental disorders among people aged 60 and older (22 374.1 per 100–000 population), and Belarus had the highest prevalence for SUDs (6 187.0 per 100–000 population). Uganda had the highest age standardized YLDs of mental disorders (3 762.4 per 100–000 population), and Belarus had the highest YLDs for SUDs (620.9 per 100–000 population) (**eTables 7**, **8**, **Supplementary 1**).

### 3.7 Prediction of prevalence and YLDs for mental disorders and SUDs over the next fifteen years

The ARIMA model was applied to analyze trends in prevalence and YLDs for mental disorders and SUDs over a 15-year projection period. For mental disorders, the prevalence rate is projected to decline from 14 800.9 per 100–000 population in 2021 to 14 448.4 in 2036, with corresponding YLDs decreasing from 2 091.0 to 2 038.2 per 100–000 during the same period. For SUDs, both prevalence (1 475.1 vs. 1 476.8 per 100 000) and YLDs (188.2 vs. 189.8 per 100 000) show minimal variation between 2021 and 2036 (**eFigure 3**, **eTable 9**, **Supplementary 1**).

## 4 Discussion

Our study advanced understanding of the global burden of mental and substance use disorders among older adults (≥60 years) by analyzing prevalence and years lived with disability trends. Mental health condition remained a significant global concern among older adults in 2021, affecting 161.3 million individuals with mental disorders and 19.2 million with SUDs. From 1990 to 2021, age standardized prevalence of mental disorders increased by 3.7%, while SUDs declined by 8.7%, revealing divergent trajectories shaped by aging-related vulnerabilities—chronic comorbidities, social isolation, and fragmented psychiatric care—alongside socioeconomic disparities across countries. These findings underscore mental health in older age as an urgent public health priority, demanding targeted guidelines, equitable resource allocation, and age-specific interventions.

Gender disparities in mental health trajectories revealed distinct risk profiles: faster progression of mental disorders in women (AAPC 0.16% vs. 0.11% in men) may stem from biological (e.g., postmenopausal hormonal shifts) and social roles (e.g., caregiving burdens), while sharper SUDs declines in females likely reflect gendered help-seeking behaviors (15, 16). Age stratification identified critical transitions: rising mental disorders prevalence in younger elderly (60–84 years) aligned with retirement-related stressors and multimorbidity accumulation, whereas lower rates in ≥85-year-olds may indicate survivorship bias or diagnostic gaps. Notably, elevated alcohol-related YLDs in the ≥95 age group implicated aging-specific risks like metabolic alterations and polypharmacy interactions (17). These findings highlighted the need for sex-specific interventions and improved SUD surveillance in advanced aging populations (18, 19).

The rising prevalence of mental disorders globally, particularly in middle-SDI regions (AAPC 0.18%), underscored age-associated susceptibilities exacerbated by fragmented mental health systems and socioeconomic transitions. Conversely, increasing SUDs in high-SDI regions (AAPC 0.45%) alongside declines elsewhere may reflect polypharmacy risks and delayed substance misuse detection in affluent aging populations. For level 3 causes, the low-SDI regions exhibited the highest burden, with depression driving both prevalence and YLDs, highlighting systemic care-access inequities. These disparities demand tailored strategies: scaling community-based mental health interventions in middle-SDI areas, enhancing geriatric SUD surveillance in high-SDI settings, and integrating mental health into primary care frameworks for low-SDI regions (20, 21).

The regional divergence of mental health burdens across 21 regions underscored complex socioeconomic and healthcare dynamics. Central Asia’s unique decline in mental disorders YLDs (AAPC -0.03%) may reflect targeted geriatric care initiatives or cultural resilience factors warranting further study. The nonlinear SDI-SUDs relationship—peaking in middle-SDI settings—suggested transitional risks (22), such as alcohol liberalization preceding robust regulation.

Nationally, Spain’s steep mental disorder increased (EAPC 0.44%) and the UK’s SUDs surge (EAPC 1.78%) highlighted vulnerabilities in high-income aging societies, potentially linked to workforce transitions and prescription drug accessibility. Our findings on extreme health burdens in Uganda and Belarus align with Baingana et al.’s (23)projection that low- and middle-income countries will experience the steepest rise in mental disease and SUDs burden due to increasing life expectancy, population growth, and under-resourced healthcare systems. Projections of stagnant SUDs trends versus slight mental disorders declines signal unmet needs. Prioritizing the integration of geriatric mental health services into primary care—particularly in low- and middle-SDI regions—combined with enhanced pharmaceutical regulation in high-income countries, may help counter these trajectories (24, 25). Given the multifactorial links between mental health, SUDs, and biological-social vulnerabilities, interventions must integrate local contexts and global patterns of adversity impacts and multimorbidity (26). Policy-driven, cross-sectoral strategies are essential to address pressing mental challenges and advance health equity.

### 4.1 Strengths and limitations of this study

We present a comprehensive assessment of global, regional, and national trends in mental and substance use disorders and their associated years lived with disability using data from the Global Burden of Disease Study 2021, enhancing understanding of late-life mental health and informing resource allocation priorities. However, we must recognize certain limitations of this study. First, as mentioned earlier, the fact that the DALYs for most mental disorders are nearly equivalent to the YLDs (12), combined with the lack of reliable data on mortality associated with these disorders, may lead to an underestimation of the global burden of mental illnesses. Second, data on conduct disorder and eating disorder are unavailable. Conduct disorder is strictly defined as conditions diagnosed in individuals under 18 years of age (4), while epidemiological gaps in data on eating disorder among individuals aged ≥60 years may limit a systematic assessment of their lifelong impact. Finally, our findings are limited by the methodological framework of the Global Burden of Disease 2021 study, and given the significant differences in health information systems and data reporting mechanisms in different countries and regions, the risk of potential bias cannot be completely ruled out although every effort was made to control for bias, and thus the findings need to be interpreted with caution. While DSM and ICD classifications ensure consistent case definitions across studies, they may lack sensitivity to all cultural contexts (27). Future research should evaluate the cross-cultural applicability of our definitions and data collection methods.

## 5 Conclusions

Global aging necessitates rethinking mental healthcare for older adults. For mental disorders, both were higher in women, those living in countries or regions with a low sociodemographic index, and those younger than 84 years. And for SUDs, both were higher in men, those living in countries or regions with a high sociodemographic index, and those older than 84 years. Our findings call for equitable resource allocation, age-adapted diagnostic tools, and policies addressing the unique psychosocial challenges of later life.

## Data Availability

All data in this study are publicly available through the GBD 2021 portal, please visit the Global Health Data Exchange at http://ghdx.healthdata.org/gbd-results-tool.
