# Supplementary material for: Global, regional, and national burden of mental disorders and substance use disorders in older adults from 1990 to 2021: a systematic analysis and future trend prediction study
Source: Front Psychiatry. 2025 Sep 24;16:1638646. doi: 10.3389/fpsyt.2025.1638646 (PMC12504471; doi:10.3389/fpsyt.2025.1638646)
Supplement: Supplementary file 1 [file DataSheet1.pdf]

Supplement1: Supplementary materials to "Global, regional, and national burden of mental disorders and substance use disorders in older adults from 1990 to 2021: A systematic analysis and future trend prediction study"

Table of content

Supplementary Methods **1**

1.Data sources 1

2.Equations used to calculate age standardised rate 2

Supplementary Figures **3**

eFigure 1. GBD 2021 Cause Hierarchy for Mental Disorders and Substance Use Disorders ... 3

eFigure 2. Prevalence of Level 2 and 3 causes by Age Group and Gender, 2021 4

eFigure 3. Prediction of Prevalence and YLDs for Mental Disorders and SUDs Over the Next Fifteen Years 5

Supplementary Tables **6**

eTable 1. GBD 2021 Definitions and Classification Codes for Mental and Substance Use Disorders 6

eTable 2. Age standardised YLDs rate and AAPC of Mental disorders, Substance use disorders in people aged ≥60 years at global and regional level, from 1990 to 2021 8

eTable 3. Global Prevalence of Mental Disorders and Substance Use Disorders per 100 000 People, by Sexes, in 1990,2021 11

eTable 4. Global YLDs of Mental Disorders and Substance Use Disorders per 100 000 People, by Sexes, in 1990,2021 16

eTable 5. Age Standardised Prevalence and AAPC of Mental disorders, Substance use disorders in people aged ≥60 years at 21 GBD regions, from 1990 to 2021 20

eTable 6. Age Standardised YLDs and AAPC of Mental disorders, Substance use disorders in people aged ≥60 years at 21 GBD regions, from 1990 to 2021 26

eTable 7. Age Standardised Prevalence and EAPC of Mental disorders, Substance use disorders in people aged ≥60 years in 204 countries and territories, from 1990 to 2021 31

eTable 8. Age Standardised YLDs and EAPC of Mental disorders, Substance use disorders in people aged ≥60 years in 204 countries and territories, from 1990 to 2021 47

eTable 9. GATHER checklist of information that should be included in reports of global health estimates 63

Supplementary Methods

1.Data sources

All data in this study were extracted from the Global Burden of Disease Study 2021, with datasets obtained from the Global Health Data Exchange (http://ghdx.healthdata.org/gbd-results-tool). GBD 2021 estimates cover 371 diseases and injuries (including 95 communicable, maternal, neonatal, and nutritional [CMNN] diseases; 234 non-communicable diseases; and 40 injuries); 204 countries and territories; 21 countries with subnational locations; 25 age groups; data for females, males, and both sexes combined; and years spanning 1990–2021[1].

The study population consisted of patients aged 60 years and older with mental disorders or substance use disorders (SUDs), including those diagnosed before age 60. As few psychiatric disorders are recorded as causes of death, the main burden of mental illness is measured through years lived with disability (YLDs) and disability-adjusted life years (DALYs)[2]. Therefore, we integrated the following data for analysis:

(1) GBD world standard population in 2021; (2)Global age- and sex-specific prevalence and YLD rates (per 100 000 population) from 1990 to 2021; (3) Regional age- and sex-specific prevalence and YLD rates (per 100,000 population) from 1990 to 2021, stratified by Socio-demographic Index (SDI) categories; (4) Regional age- and sex-specific prevalence and YLD rates (per 100,000 population) from 1990 to 2021 across 21 GBD regions; (5)National age- and sex-specific prevalence and YLD rates (per 100,000 population) from 1990 to 2021.

2.Equations used to calculate age standardised rate

$$\textbf{Age standardised rate} = \frac{\sum_{i=1}^{A} a_i w_i}{\sum_{i=1}^{A} w_i}$$

where

- a_i is the age-specific rate in the ith age class,
- w_i is the weight assigned to the same age subgroup within the reference standard population,
- i indexes the age class ($i = 1, 2, ..., A$),
- A represents the upper age limit of the population.

References:

1. Global incidence, prevalence, years lived with disability (YLDs), disability-adjusted life-years (DALYs), and healthy life expectancy (HALE) for 371 diseases and injuries in 204 countries and territories and 811 subnational locations, 1990-2021: a systematic analysis for the Global Burden of Disease Study 2021. *Lancet*. 2024; 403(10440): p. 2133-2161. 10.1016/s0140-6736(24)00757-8

2. The Lancet, P. Global Burden of Disease 2021: mental health messages. *Lancet Psychiatry*. 2024; 11(8): p. 573. 10.1016/s2215-0366(24)00222-0

Supplementary Figures

eFigure 1. GBD 2021 Cause Hierarchy for Mental Disorders and Substance Use Disorders

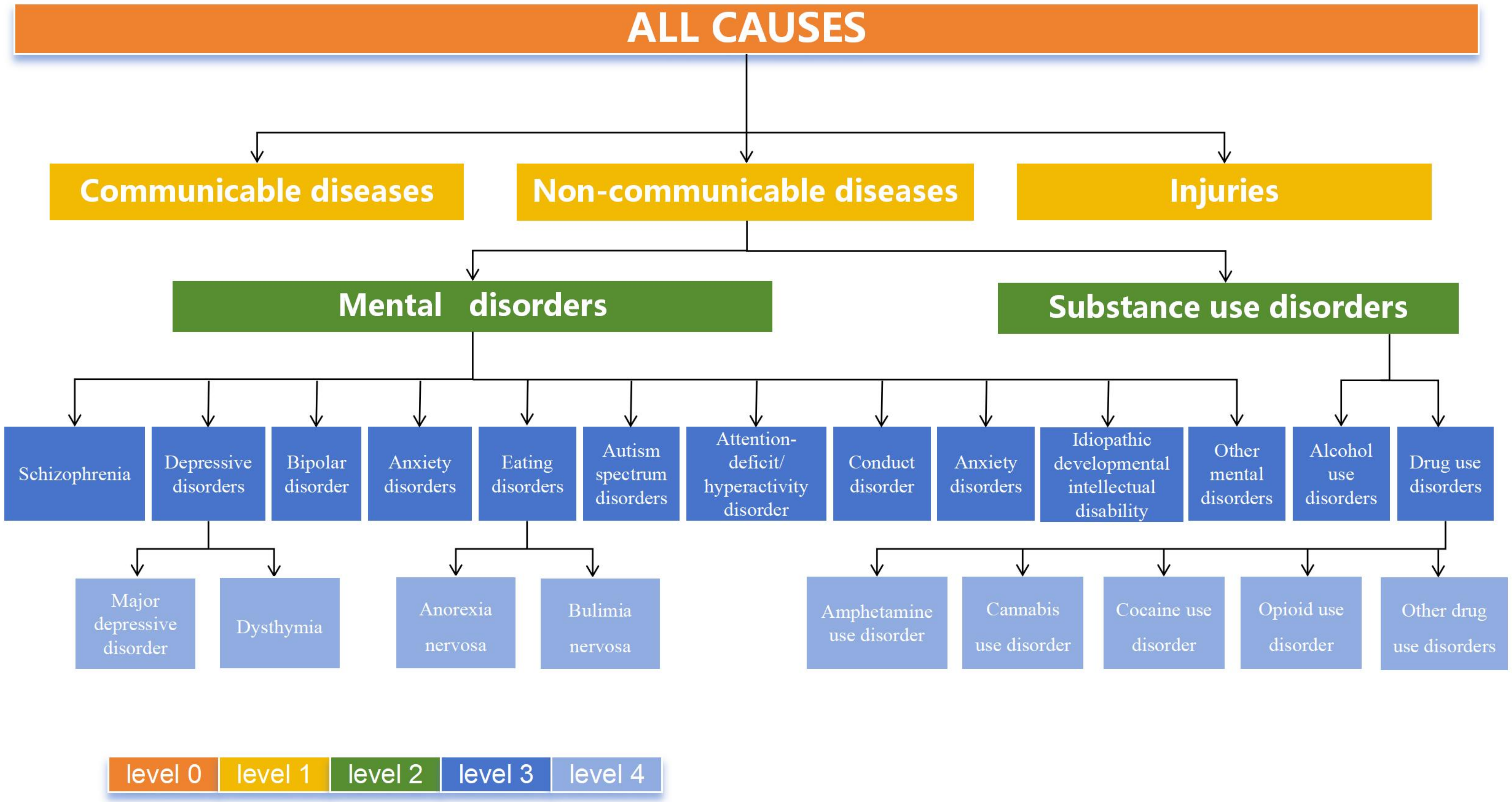

eFigure 2. Prevalence of Level 2 and 3 causes by Age Group and Gender, 2021

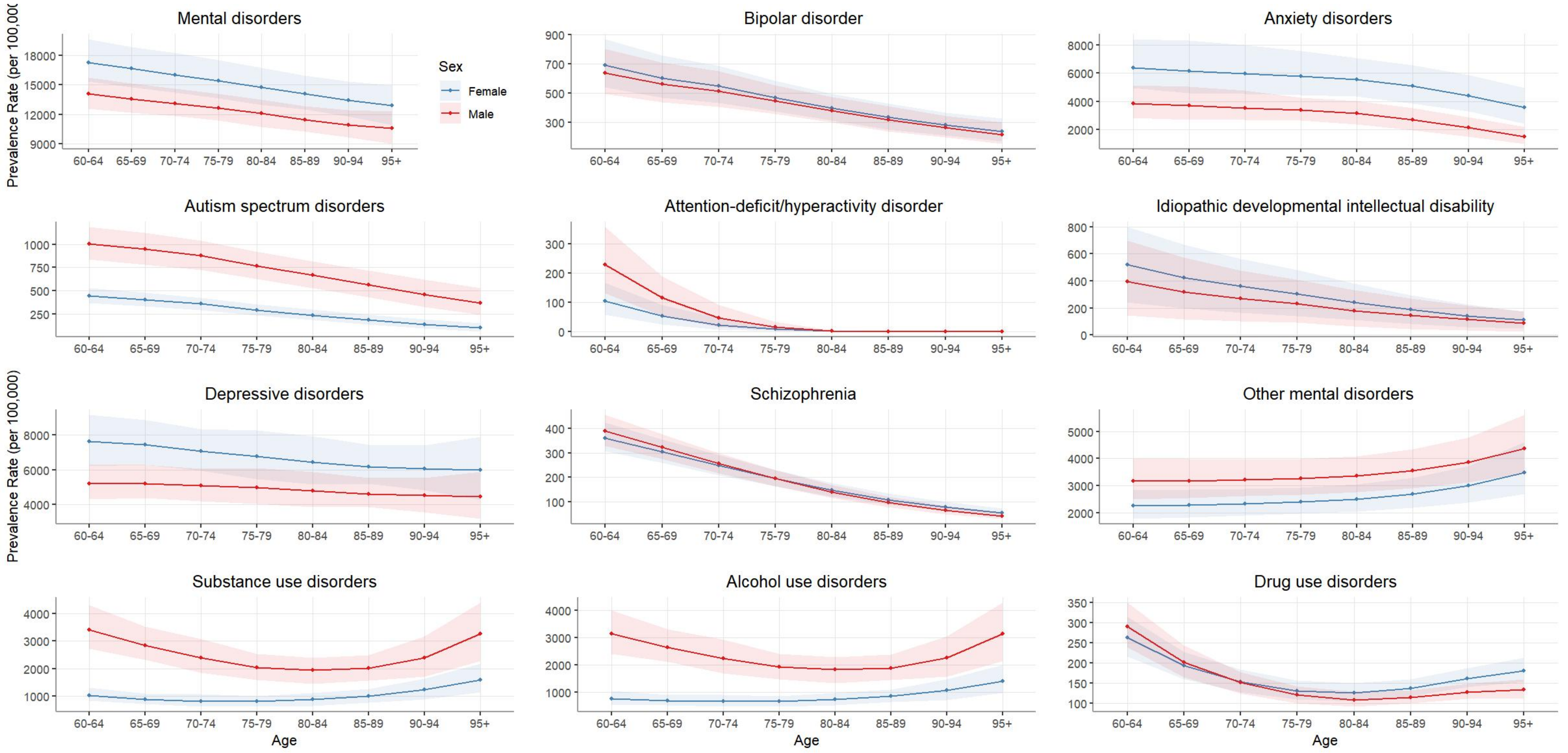

eFigure 3. Prediction of Prevalence and YLDs for Mental Disorders and SUDs Over the Next Fifteen Years

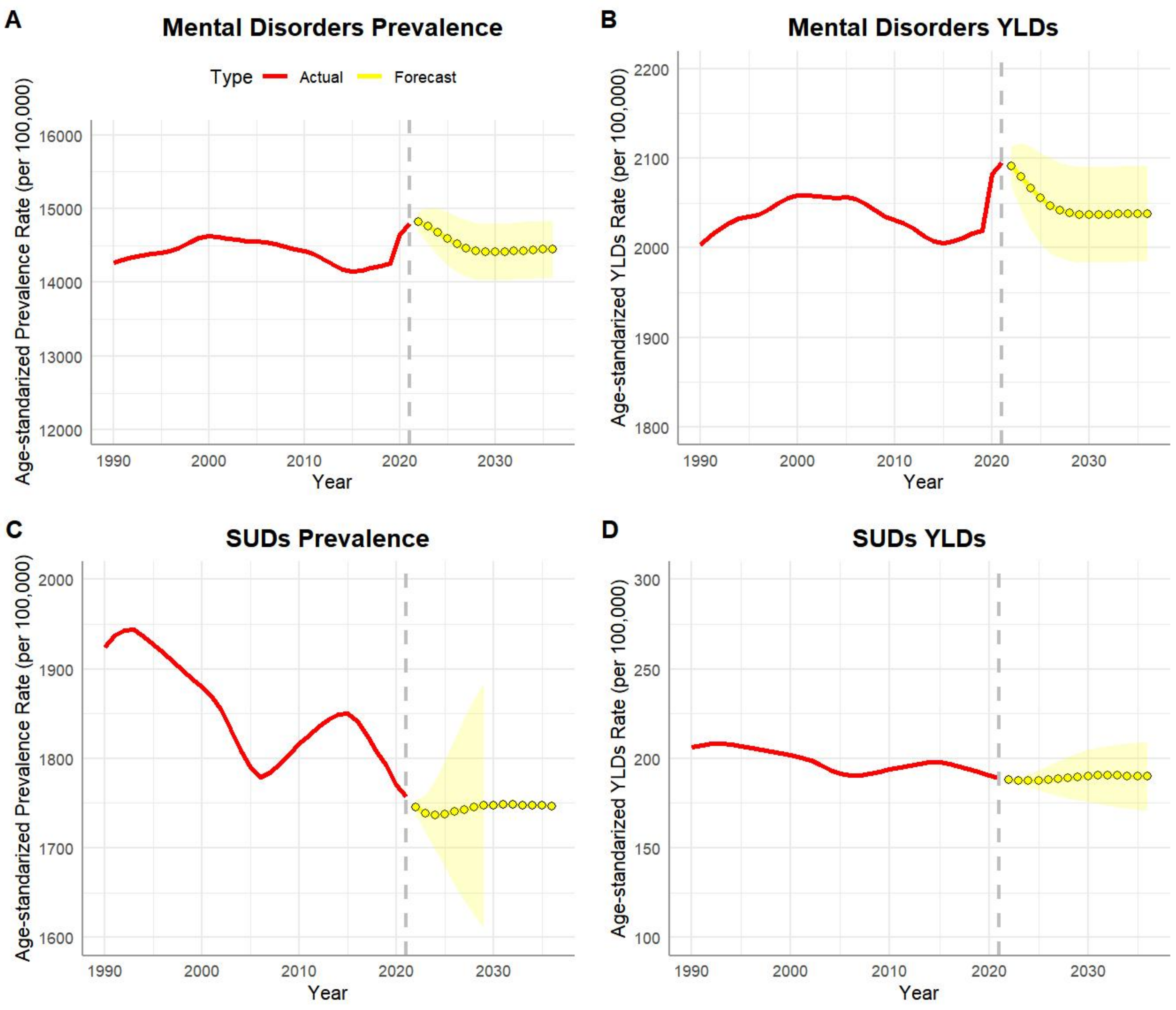

Supplementary Tables

eTable 1. GBD 2021 Definitions and Classification Codes for Mental and Substance Use Disorders

Cause	Definition	Corresponding DSM-IV-TR and ICD-10 codes
**Mental disorders**		
**Schizophrenia**	A chronic psychotic disorder is characterized by the presence of positive symptoms (e.g., delusions, hallucinations, thought disorder) and negative symptoms (e.g., flat affect, loss of interest, and emotional withdrawal).	DSM-IV-TR: 295.10-295.30, 295.60, 295.90; ICD 10: F20
**Depressive disorders**		
Major depressive disorder	Major depressive disorder (MDD) is an episodic mood disorder involving the occurrence of one or more major depressive episodes.	DSM-IV-TR: 296.21–24, 296.31–34; ICD-10: F32.0–9, F33.0–9
Dysthymia	Dysthymia is a mood disorder characterized by chronic depression, with less severe but longer-lasting symptoms than major depressive disorder.	DSM-IV-TR: 300.4; ICD-10: F34.1
**Bipolar disorder**	Bipolar disorder is a serious mood disorder with infrequent or no complete remission periods.	DSM-IV-TR: 296.0–296.7, 296.89, 301.13; ICD-10: F30.0-F30.9, F31.0–F31.6, F31.8–F31.9, F34.0
**Anxiety disorders**	Anxiety disorders are characterized by the experience of intense fear and distress, often accompanied by physiological symptoms.	DSM-IV-TR: 300.0-300.3, 208.3, 309.21, 309.81; ICD-10: F40-42, F43.0, F43.1, F93.0-93.2, F93.8
**Eating disorders**		
Anorexia nervosa	Anorexia nervosa is characterized by refusal to maintain body weight at or above a minimally normal level for age and height, intense fear of gaining weight, and a distorted perception of one's body weight or shape.	DSM-IV-TR: 307.1; ICD-10: F50.0-50.1
Bulimia nervosa	Bulimia nervosa is characterized by recurrent episodes of binge eating followed by inappropriate compensatory behaviors to prevent weight gain. These behaviors must occur, on average, at least twice weekly for a minimum of three months.	DSM-IV-TR: 307.51; ICD-10: F50.2
**Autism spectrum disorders**	Autism spectrum disorders (ASD) – also known as pervasive developmental disorders – are a group of neurodevelopmental disorders with onset in early childhood.	DSM-IV-TR: 299.00, 299.80, 299.8, 299.8, 299.10; ICD-10: F84.0, F84.1, F84.2, F84.3, F84.4, F84.5, F84.8, F84.9
**Attention-deficit/hyperactivity disorder**	Attention-deficit/hyperactivity disorder (ADHD) is an externalizing disorder characterized by persistent inattention and/or hyperactivity-impulsivity.	DSM-IV-TR: 314.0, 314.01; ICD-10: F90

**Conduct disorder**	Conduct disorder (CD) is an externalizing behavior disorder characterized by a persistent pattern of antisocial behavior that violates the basic rights of others or societal norms appropriate to the individual's age.	DSM-IV-TR: 312.81-312.89; ICD-10: F91
**Idiopathic developmental intellectual disability**	Developmental intellectual disability (ID) is a condition characterized by significant limitations in both intellectual functioning and adaptive behavior.	Profound (IQ score 0-19), severe (IQ score 20-34), moderate (IQ Score 35-49), mild (IQ score 50 to 69), and Involves borderline (IQ Score 70-85)
**Other mental disorders**	"Other mental disorders" is a residual category in the GBD framework, encompassing disabilities from a group of personality disorders. Personality disorders are characterized by pervasive, inflexible, and maladaptive patterns of behavior and inner experience that deviate markedly from cultural expectations.	DSM-IV-TR: 300.3, 301.0; 301.2, 301.22, 301.5–301.9; ICD-10: F60
**Substance use disorders**		
**Alcohol use disorders**	Alcohol dependence is a substance-related disorder defined by a dysfunctional pattern of alcohol use.	DSM-IV-TR: 303.90; ICD 10: F10.1 and F10.2
**Drug use disorders**		
Amphetamine use disorder	Amphetamine use disorder is characterized by a substance-related disorder involving a dysfunctional pattern of amphetamine use.	DSM-IV-TR: 304.40; ICD-10: F15-F15.9
Cannabis use disorder	Cannabis dependence is a substance-related disorder defined by a dysfunctional pattern of cannabis use.	DSM-IV-TR:304.30; ICD-10: F12.2
Cocaine use disorder	Cocaine dependence is a substance-related disorder defined by a dysfunctional pattern of cocaine use.	DSM-IV-TR: 304.20; ICD-10: F14.2
Opioid use disorder	Opioid use disorders are defined as a maladaptive pattern of opioid abuse leading to clinically significant impairment or distress, manifested through symptoms such as withdrawal or tolerance.	DSM-IV-TR:304.00; ICD-10: F11.2
Other drug use disorders	In addition to the four drug use disorders specifically included in non-fatal burden estimates (opioid, cocaine, amphetamine, and cannabis dependence), the burden attributable to a residual category of "other drug use disorders" is also estimated.	DSM-IV-TR: 304.00; ICD-10: F11-F11.99, R78.1

Note: Refer to eFigure 1 for information on the GBD disease hierarchy.

eTable 2. Age standardised YLDs rate and AAPC of Mental disorders, Substance use disorders in people aged ≥60 years at global and regional level, from 1990 to 2021

Cause	Rate (95% UI/95%CI)																	
	Global			High SDI			High-middle SDI			Middle SDI			Low-middle SDI			Low SDI		
	1990	2021	AAPC,%	1990	2021	AAPC,%	1990	2021	AAPC,%	1990	2021	AAPC,%	1990	2021	AAPC,%	1990	2021	AAPC,%
**Mental disorders**																		
Male	1760.1 (1305.9 ,2255)	1850.3 (1373 ,2373.3)	0.17 (0.16,0.18)	1636.3 (1225.6 ,2075.8)	1687.7 (1267.9 ,2138.6)	0.11 (0.10,0.13)	1700.3 (1263.6 ,2181.4)	1775.3 (1323.8 ,2275.2)	0.13 (0.12,0.14)	1705.2 (1266 ,2187.6)	1850.4 (1371.4 ,2377.3)	0.27 (0.26,0.27)	2017.4 (1472.8 ,2607.9)	2102.7 (1540.8 ,2717.8)	0.15 (0.13,0.18)	2170.6 (1560.7 ,2822.1)	2218.4 (1589.1 ,2883.7)	0.08 (0.06,0.10)
Female	2198.1 (1593.2 ,2870.9)	2307.5 (1680.5 ,3015.9)	0.17 (0.13,0.20)	2058.7 (1502.2 ,2667.5)	2149 (1570.6 ,2795.8)	0.16 (0.13,0.20)	2295.2 (1655 ,3009.8)	2349.7 (1704.6 ,3083.6)	0.07 (0.06,0.09)	2092.1 (1516.9 ,2747.9)	2270.3 (1652.1 ,2979.5)	0.28 (0.25,0.31)	2386.7 (1711.5 ,3110.9)	2485.9 (1798.6 ,3242)	0.16 (0.11,0.20)	2586.3 (1831.6 ,3414.1)	2616.5 (1843.7 ,3452.7)	0.05 (0.02,0.08)
Both	2003.4 (1464 ,2598.8)	2095.2 (1537.3 ,2718.8)	0.15 (0.12,0.17)	1881.7 (1386.9 ,2426.9)	1934 (1429.2 ,2502.7)	0.11 (0.08,0.14)	2051.8 (1495.7 ,2670.4)	2092.8 (1535 ,2717.6)	0.07 (0.06,0.08)	1910.3 (1399.6 ,2481.6)	2073.3 (1517.4 ,2696.8)	0.27 (0.26,0.29)	2200.3 (1592.1 ,2853.8)	2304.1 (1685.5 ,2996.5)	0.16 (0.13,0.19)	2375.7 (1696.4 ,3105.5)	2423.7 (1726.3 ,3172.9)	0.07 (0.05,0.09)
**Schizophrenia**																		
Male	160.3 (116.7 ,205.6)	167.3 (122.5 ,214.5)	0.14 (0.14,0.14)	169 (122.2 ,217.7)	166.7 (122.5 ,215.5)	-0.05 (-0.06,-0.04)	156.4 (112.4 ,198.9)	170.6 (124.5 ,217.1)	0.29 (0.28,0.30)	164 (118.9 ,212.9)	170.3 (123.4 ,220.7)	0.12 (0.11,0.12)	157.6 (112.3 ,206.2)	168.5 (120.5 ,220.8)	0.22 (0.21,0.22)	130.1 (92.1 ,172.1)	140.2 (97.9 ,186.5)	0.24 (0.23,0.25)
Female	153.5 (112.7 ,196)	157.2 (114.9 ,201.4)	0.08 (0.07,0.08)	181.9 (132.2 ,234.9)	182.5 (132.7 ,233.3)	0.02 (0.01,0.03)	144.7 (105.6 ,184.6)	157.3 (115.9 ,198.8)	0.28 (0.27,0.28)	146.3 (106.1 ,187.5)	150.6 (108.8 ,194.1)	0.09 (0.08,0.10)	132.4 (94.4 ,172.9)	142 (102.2 ,185.2)	0.23 (0.23,0.24)	122.6 (87.3 ,162.6)	129.5 (91.8 ,171.5)	0.18 (0.17,0.18)
Both	157 (115.2 ,200.4)	162.2 (118.9 ,207.8)	0.10 (0.10,0.11)	176.7 (128.2 ,226.8)	175.2 (128 ,224.6)	-0.03 (-0.03,-0.02)	149.9 (108.8 ,190.5)	163.5 (120.5 ,206.6)	0.29 (0.28,0.30)	155 (113.2 ,199)	160.1 (115.6 ,206.2)	0.10 (0.10,0.11)	145.2 (104 ,189.3)	154.7 (111.4 ,201.9)	0.20 (0.20,0.21)	126.5 (90.6 ,166.6)	134.7 (95.4 ,177.7)	0.20 (0.19,0.21)
**Depressive disorders**																		
Male	743.2 (503.9 ,1023.7)	795.2 (540.4 ,1102.6)	0.22 (0.21,0.24)	531.8 (362.5 ,729.4)	546.2 (370.1 ,758.8)	0.11 (0.08,0.13)	720.7 (490.4 ,992.3)	762 (520.7 ,1052.3)	0.15 (0.13,0.18)	706.7 (482.8 ,972.3)	801.2 (545.9 ,1111.7)	0.40 (0.39,0.41)	1025.7 (687.7 ,1426.7)	1070.5 (714.2 ,1492.5)	0.16 (0.13,0.21)	1253.7 (829.8 ,1760.4)	1261.8 (826.8 ,1767.2)	0.03 (-0.00,0.06)
Female	1071.7 (727.2 ,1479.1)	1142.1 (776.2 ,1568.2)	0.23 (0.19,0.27)	799.6 (547.8 ,1102.2)	831.7 (560.6 ,1160.8)	0.16 (0.11,0.21)	1172.4 (796 ,1614.3)	1190 (808.3 ,1634.3)	0.06 (0.02,0.09)	1024.7 (695.8 ,1407.3)	1131.9 (770.2 ,1557.2)	0.35 (0.29,0.40)	1381.8 (923.9 ,1923)	1421.9 (948.1 ,1974.5)	0.11 (0.07,0.16)	1655.5 (1094.4 ,2325.4)	1644.6 (1067.2 ,2315)	-0.01 (-0.04,0.03)
Both	924.3 (626.9 ,1275.2)	980.4 (667.2 ,1352.4)	0.21 (0.18,0.24)	687 (470.1 ,943.3)	698.7 (472.3 ,976.2)	0.08 (0.05,0.12)	986.9 (670.3 ,1357.1)	997.9 (679.7 ,1372)	0.03 (0.02,0.05)	874.2 (595.5 ,1202.4)	976.1 (664.1 ,1347.2)	0.37 (0.34,0.40)	1201.6 (803.3 ,1668)	1254.6 (837.2 ,1742.5)	0.16 (0.14,0.20)	1451.4 (960.9 ,2034.3)	1458.9 (948.8 ,2047.8)	0.01 (-0.01,0.04)
**Bipolar disorder**																		
Male	110 (68.7 ,162.8)	104.9 (65.6 ,156.5)	-0.15 (-0.16,-0.15)	140.8 (89.5 ,206.9)	134.8 (85.5 ,198)	-0.14 (-0.15,-0.14)	103 (63.8 ,154.1)	90.7 (55.8 ,136.5)	-0.41 (-0.41,-0.40)	87 (53.9 ,129.2)	90.2 (56 ,134)	0.12 (0.11,0.13)	104.1 (64.4 ,155.3)	105.6 (64.7 ,158.9)	0.05 (0.04,0.05)	112.7 (69.1 ,170)	111.9 (68.2 ,167.7)	-0.02 (-0.02,-0.01)

Female	120.2 (75.3 ,177.6)	110.9 (69.9 ,164)	-0.26 (-0.26,-0.25)	158.2 (100.5 ,230.6)	149.4 (95.6 ,217.4)	-0.18 (-0.19,-0.18)	116.3 (72.1 ,173.3)	101.6 (63.2 ,151.6)	-0.44 (-0.45,-0.43)	87.5 (54.3 ,130.3)	91.6 (57.2 ,135.6)	0.16 (0.15,0.16)	102.2 (63.6 ,151.9)	103.3 (63.6 ,154.9)	0.03 (0.03,0.04)	112.2 (67.9 ,168.3)	110.6 (67.6 ,167.7)	-0.05 (-0.06,-0.04)
Both	115.6 (72.3 ,170.9)	108.1 (67.8 ,160.7)	-0.22 (-0.22,-0.21)	150.7 (95.9 ,219.3)	142.5 (91.2 ,208.5)	-0.18 (-0.18,-0.18)	110.6 (68.6 ,165.1)	96.6 (59.8 ,144.2)	-0.43 (-0.44,-0.43)	87.1 (54.1 ,129.6)	90.9 (56.6 ,135.3)	0.14 (0.13,0.15)	103.1 (64.4 ,153.6)	104.4 (64.4 ,156.8)	0.04 (0.04,0.04)	112.4 (68.8 ,169.9)	111.2 (68 ,167.6)	-0.04 (-0.04,-0.03)
**Anxiety disorders**																		
Male	360.4 (231.6 ,532)	390 (250.4 ,572.3)	0.27 (0.24,0.30)	362.1 (230.1 ,541.2)	390.9 (249.5 ,588.5)	0.28 (0.25,0.32)	339.7 (218 ,502.8)	362.4 (230.3 ,538.5)	0.21 (0.20,0.23)	391.7 (255.2 ,577.1)	422.1 (275.1 ,617.9)	0.25 (0.23,0.28)	357 (230.3 ,527.1)	389.2 (244.1 ,578.4)	0.32 (0.27,0.36)	308.6 (195.2 ,465.2)	330.1 (207.4 ,497.5)	0.27 (0.22,0.31)
Female	608.7 (404.2 ,884.9)	654.1 (434.3 ,946.7)	0.25 (0.22,0.28)	670.7 (447.1 ,976.7)	733.8 (483.3 ,1064.9)	0.32 (0.28,0.37)	622.6 (412.1 ,907.9)	666.3 (438.9 ,971.6)	0.22 (0.20,0.23)	600.5 (400.5 ,870.5)	662 (444.3 ,944.8)	0.32 (0.29,0.35)	508 (333.2 ,750)	561.9 (367.7 ,822.6)	0.37 (0.31,0.43)	447.5 (284.5 ,681.5)	475.9 (304.3 ,729.7)	0.23 (0.19,0.27)
Both	498.6 (329.4 ,726.2)	531.8 (351.8 ,769.4)	0.23 (0.20,0.25)	540.3 (358.4 ,786.9)	573.7 (380.3 ,841.8)	0.22 (0.18,0.27)	506.5 (333.4 ,739.9)	530.4 (346.7 ,775.9)	0.15 (0.14,0.16)	502.9 (332.8 ,728.6)	549.6 (367 ,788.8)	0.30 (0.27,0.32)	432.2 (281.4 ,638.3)	480.4 (311.6 ,705.1)	0.38 (0.33,0.43)	377.3 (242.1 ,569.6)	405.4 (259.4 ,612.8)	0.26 (0.23,0.30)
**Autism spectrum disorders**																		
Male	140 (96.5 ,194.6)	151.7 (104 ,212.2)	0.26 (0.26,0.26)	189 (130.2 ,261.9)	209 (144.7 ,291.8)	0.32 (0.32,0.33)	145.1 (99.1 ,203)	157.1 (107.6 ,220.7)	0.26 (0.25,0.26)	112.1 (77.8 ,157)	127.7 (87 ,178.9)	0.42 (0.42,0.42)	103.5 (71.4 ,143.3)	112.4 (77 ,156.4)	0.27 (0.27,0.28)	112.9 (77.7 ,157.3)	121 (83 ,167.6)	0.23 (0.22,0.23)
Female	61.3 (42.4 ,85)	62.4 (42.8 ,86.6)	0.06 (0.06,0.07)	77.7 (53.4 ,106.9)	84.8 (58 ,117.6)	0.28 (0.28,0.29)	62.2 (42.5 ,85.7)	60.2 (41.4 ,84)	-0.10 (-0.11,-0.10)	46.7 (32.2 ,65.3)	51.4 (35.2 ,72)	0.31 (0.31,0.32)	50.5 (34.9 ,70)	53.6 (36.8 ,74.9)	0.19 (0.18,0.19)	55.5 (38 ,77.7)	58.5 (40.1 ,81.2)	0.17 (0.17,0.18)
Both	97 (66.6 ,134.7)	104 (71.3 ,145.5)	0.23 (0.22,0.23)	125.5 (86.4 ,173.8)	142.8 (98.3 ,198.1)	0.42 (0.41,0.42)	97 (66.3 ,134.9)	104 (71.3 ,146)	0.23 (0.22,0.23)	77.9 (53.8 ,108.9)	87.4 (59.4 ,122.5)	0.37 (0.37,0.38)	77.4 (53.6 ,107.3)	81.6 (55.9 ,113.6)	0.17 (0.17,0.18)	84.9 (58.6 ,118.1)	89 (60.9 ,123.4)	0.15 (0.14,0.15)
**Attention-deficit/hyperactivity disorder**																		
Male	1.2 (0.6 ,2.2)	1.2 (0.6 ,2.2)	-0.04 (-0.05,-0.03)	1 (0.4 ,1.8)	1 (0.5 ,1.9)	-0.08 (-0.20,-0.01)	1.5 (0.7 ,2.7)	1.6 (0.7 ,2.8)	0.05 (-0.01,0.11)	1.7 (0.8 ,2.9)	1.5 (0.7 ,2.7)	0.42 (0.42,0.42)	0.7 (0.3 ,1.3)	0.7 (0.3 ,1.4)	0.27 (0.25,0.28)	0.5 (0.2 ,0.9)	0.5 (0.2 ,0.9)	-0.13 (-0.16,-0.11)
Female	0.6 (0.2 ,1)	0.6 (0.2 ,1)	-0.02 (-0.03,-0.0012)	0.4 (0.2 ,0.9)	0.5 (0.2 ,0.9)	0.87 (0.74,1.13)	0.6 (0.3 ,1.2)	0.7 (0.3 ,1.3)	0.01 (-0.10,0.13)	0.7 (0.3 ,1.4)	0.6 (0.3 ,1.2)	0.31 (0.31,0.32)	0.4 (0.1 ,0.7)	0.4 (0.1 ,0.7)	0.06 (0.05,0.08)	0.2 (0.1 ,0.5)	0.2 (0.1 ,0.4)	-0.10 (-0.12,-0.08)
Both	0.9 (0.4 ,1.6)	0.9 (0.4 ,1.6)	-0.03 (-0.04,-0.01)	0.7 (0.3 ,1.3)	0.8 (0.3 ,1.4)	0.33 (0.06,0.49)	1 (0.5 ,1.9)	1.1 (0.5 ,2)	0.30 (0.25,0.35)	1.2 (0.6 ,2.1)	1 (0.5 ,1.9)	0.37 (0.37,0.38)	0.5 (0.2 ,1)	0.5 (0.2 ,1)	0.13 (0.12,0.15)	0.4 (0.1 ,0.7)	0.3 (0.1 ,0.7)	-0.18 (-0.19,-0.16)
**Idiopathic developmental intellectual disability**																		

Male	18.8 (7.4 ,34.7)	13.9 (5.5 ,25.9)	-0.95 (-0.97,-0.93)	8 (2 ,17)	5 (0.9 ,11.7)	-1.55 (-1.58,-1.52)	9.7 (3 ,19.9)	6 (1.3 ,13.7)	-1.54 (-1.58,-1.51)	17.1 (6.1 ,32.8)	13 (4.5 ,24.6)	-0.90 (-0.92,-0.87)	48.1 (21.3 ,83.3)	34.3 (15.4 ,58.8)	-1.04 (-1.07,-1.00)	33 (15.2 ,57.8)	32.1 (14.4 ,55.9)	-0.04 (-0.08,-0.00)
Female	19.9 (8.8 ,34.4)	17.6 (8.1 ,30)	-0.39 (-0.40,-0.37)	10.6 (4.2 ,19)	7.1 (2.4 ,13.1)	-1.31 (-1.33,-1.29)	12.3 (4.9 ,22.1)	9.2 (3.2 ,17)	-0.93 (-0.95,-0.91)	20.4 (8.7 ,35.9)	17.4 (7.6 ,30.1)	-0.52 (-0.54,-0.49)	49.9 (23.8 ,83.8)	41.1 (21.4 ,66.7)	-0.63 (-0.65,-0.61)	32.6 (15.8 ,55.7)	36.4 (18.3 ,60)	0.37 (0.35,0.39)
Both	19.4 (8.1 ,34.5)	15.9 (6.8 ,28)	-0.63 (-0.65,-0.61)	9.6 (3.5 ,18.1)	6.1 (1.8 ,12.4)	-1.45 (-1.47,-1.43)	11.2 (4.1 ,21.1)	7.8 (2.4 ,15.5)	-1.17 (-1.19,-1.15)	18.9 (7.5 ,34.4)	15.3 (6.2 ,27.5)	-0.68 (-0.70,-0.65)	49 (22.5 ,83.6)	37.9 (18.4 ,62.9)	-0.81 (-0.86,-0.79)	32.8 (15.4 ,56.6)	34.3 (16.4 ,58.4)	0.16 (0.14,0.18)
**Other mental disorders**																		
Male	226.2 (147.9 ,337.6)	226 (148 ,336.6)	-0.0028 (-0.0045,-0.0011)	234.5 (153.5 ,344.3)	234 (152.7 ,344.9)	-0.01 (-0.01,-0.0047)	224.1 (145.2 ,336)	225 (146.4 ,336.3)	0.01 (0.01,0.02)	224.8 (145.6 ,336.5)	224.5 (145.5 ,335.6)	-0.01 (-0.01,-0.0035)	220.7 (144 ,329.6)	221.5 (144.1 ,329.4)	0.01 (0.0068,0.012)	219.2 (143.2 ,325.9)	220.8 (143.2 ,328.3)	0.02 (0.02,0.03)
Female	162.3 (106.1 ,241.4)	162.6 (105.9 ,242.4)	0.0052 (0.0036,0.0067)	159.7 (104.5 ,237.1)	159.4 (104.4 ,235.7)	-0.01 (-0.01,-0.0036)	164 (106.5 ,245)	164.3 (105.8 ,245.7)	0.01 (0.0033,0.01)	165.3 (107.3 ,248.6)	164.9 (106.8 ,246.8)	-0.01 (-0.01,-0.0042)	161.7 (104.5 ,241.3)	161.8 (106.1 ,244.1)	0.0041 (0.0009,0.0066)	160.2 (103.3 ,238.9)	160.8 (105.3 ,238.3)	0.01 (0.01,0.02)
Both	190.7 (124.5 ,283.4)	191.9 (125.3 ,285.4)	0.020 (0.018,0.021)	191.3 (125.5 ,282.6)	194.1 (127.5 ,286.7)	0.05 (0.04,0.05)	188.8 (122.3 ,281.8)	191.4 (124.1 ,286.2)	0.05 (0.04,0.05)	193.2 (125 ,289.1)	192.7 (124.8 ,288)	-0.01 (-0.01,-0.01)	191.4 (124.7 ,283.6)	190 (124 ,283.4)	-0.03 (-0.028,-0.023)	190.1 (124 ,282.4)	189.9 (123.8 ,281.7)	-0.01 (-0.01,-0.0042)
**Substance use disorders**																		
Male	288.6 (190.6 ,401.4)	271.3 (181.2 ,377.8)	-0.21 (-0.24,-0.18)	252 (171.1 ,341.6)	289.5 (200 ,389)	0.46 (0.42,0.50)	382.5 (253.4 ,527.6)	305.8 (202.4 ,428.2)	-0.76 (-0.79,-0.73)	234 (152.4 ,331.3)	229 (147.7 ,327.1)	-0.09 (-0.13,-0.05)	300.4 (191.4 ,432.7)	280.9 (182.4 ,400.4)	-0.26 (-0.30,-0.23)	293.1 (187.3 ,419.5)	290.2 (187.6 ,412.9)	-0.07 (-0.14,-0.03)
Female	135.9 (92 ,187)	116.6 (80.5 ,158.5)	-0.49 (-0.53,-0.46)	127.3 (86.2 ,174)	178.6 (125.3 ,235.1)	1.13 (1.06,1.18)	194.5 (129.8 ,268)	122.5 (82 ,169.4)	-1.48 (-1.56,-1.42)	110.8 (74.5 ,153.2)	82.5 (54.9 ,114.4)	-0.98 (-1.01,-0.96)	96.6 (64.2 ,135)	94.2 (63.5 ,131.6)	-0.08 (-0.09,-0.07)	102.3 (68 ,143.3)	99.7 (67.2 ,138.6)	-0.10 (-0.13,-0.07)
Both	206.1 (137.6 ,285.5)	189.2 (128.4 ,260.3)	-0.28 (-0.29,-0.27)	182.3 (123.2 ,248.3)	231 (161.4 ,306.2)	0.81 (0.77,0.85)	275 (184 ,377.6)	206 (137.1 ,284.9)	-0.93 (-0.95,-0.90)	169.5 (112.2 ,236)	151.5 (99.2 ,214.2)	-0.39 (-0.42,-0.36)	199.8 (129.3 ,284.3)	182.8 (120.4 ,257.6)	-0.31 (-0.34,-0.29)	201.1 (130.1 ,285.9)	193.1 (127 ,272)	-0.17 (-0.24,-0.13)
**Alcohol use disorders**																		
Male	249 (160 ,355.9)	234.1 (151.6 ,334.1)	-0.20 (-0.23,-0.18)	213.4 (140.8 ,298.5)	224.9 (150.9 ,315)	0.20 (0.17,0.22)	333.4 (216.5 ,471)	273.2 (176.2 ,391)	-0.68 (-0.71,-0.65)	189.8 (118 ,279.7)	200.7 (124.7 ,294.2)	0.15 (0.11,0.20)	275 (170.6 ,405.9)	253.7 (160.9 ,369.2)	-0.31 (-0.35,-0.27)	268.1 (169.3 ,392.4)	263.6 (167.3 ,382.9)	-0.10 (-0.17,-0.05)
Female	86.1 (53.8 ,126.6)	67.3 (42.3 ,98.5)	-0.78 (-0.82,-0.74)	83.7 (52.8 ,123)	85.8 (55.7 ,123.6)	0.14 (0.09,0.18)	136 (85 ,198.8)	80.5 (50.4 ,118.2)	-1.67 (-1.78,-1.57)	50.4 (30.5 ,76.3)	47.8 (28.8 ,72.5)	-0.20 (-0.23,-0.16)	60.9 (37.4 ,92.8)	59.6 (36.6 ,89.7)	-0.07 (-0.08,-0.06)	75.4 (47.7 ,111.6)	73.9 (48 ,108.2)	-0.09 (-0.13,-0.05)
Both	160.8 (102.5 ,231.8)	145.5 (94 ,208.4)	-0.33 (-0.35,-0.31)	140.6 (91.6 ,198.8)	151.5 (101.2 ,213.5)	0.27 (0.23,0.31)	220.2 (141.5 ,314.6)	168.1 (108.3 ,240.9)	-0.86 (-0.89,-0.84)	116.6 (72 ,172.9)	119.8 (74.2 ,176.2)	0.05 (0.01,0.10)	169.3 (104.9 ,250.6)	151.7 (96 ,221.5)	-0.40 (-0.43,-0.37)	175.2 (110.9 ,256.6)	167 (106.9 ,241)	-0.14 (-0.20,-0.08)
**Drug use disorders**																		

Male	39.6 (26.2 ,55.1)	37.2 (25.6 ,50)	-0.21 (-0.23,-0.18)	38.6 (25.7 ,52.9)	64.6 (44.8 ,85)	1.67 (1.63,1.71)	49.2 (32.2 ,69.5)	32.5 (21.7 ,45.2)	-1.33 (-1.35,-1.31)	44.1 (28.5 ,62.9)	28.3 (18.7 ,39.6)	-1.43 (-1.46,-1.39)	25.4 (16.4 ,36.8)	27.2 (18 ,38.7)	0.21 (0.20,0.22)	25 (16 ,36.1)	26.6 (17.5 ,37.8)	0.20 (0.19,0.21)
Female	49.7 (32.6 ,70.1)	49.3 (33.3 ,67.5)	-0.05 (-0.07,-0.03)	43.6 (28.9 ,60.9)	92.7 (63.7 ,124.4)	2.47 (2.38,2.56)	58.4 (38 ,82.8)	42 (27.4 ,59.4)	-1.06 (-1.08,-1.04)	60.5 (39.2 ,86.9)	34.6 (22.3 ,50.1)	-1.78 (-1.80,-1.77)	35.6 (22.2 ,52.4)	34.6 (22.3 ,50.1)	-0.09 (-0.10,-0.08)	26.9 (16.5 ,40.6)	25.8 (15.8 ,38.4)	-0.14 (-0.16,-0.12)
Both	45.4 (29.9 ,63.4)	43.7 (29.8 ,59.4)	-0.12 (-0.13,-0.10)	41.7 (27.7 ,57.6)	79.5 (54.9 ,105.5)	2.13 (2.08,2.17)	54.8 (35.9 ,77.4)	37.9 (25.1 ,53)	-1.19 (-1.21,-1.17)	52.9 (34.3 ,75.2)	31.7 (20.8 ,45.1)	-1.64 (-1.66,-1.62)	30.5 (19.4 ,44.4)	31.1 (20.6 ,44)	0.06 (0.05,0.07)	26 (16.5 ,38)	26.2 (17 ,37.4)	0.05 (0.03,0.06)

Note: YLDS-years lived with disability; AAPC-average annual percentage changes; 95% CI-95% confidence intervals(AAPC); 95% UI-95% uncertainty interval

eTable 3. Global Prevalence of Mental Disorders and Substance Use Disorders per 100 000 People, by Sexes, in 1990,2021

Cause	Rate (95% UI)															
	Age 60-64y		Age 65-69y		Age 70-74y		Age 75-79y		Age 80-84y		Age 85-89y		Age 90-94y		Age 95+y	
	1990	2021	1990	2021	1990	2021	1990	2021	1990	2021	1990	2021	1990	2021	1990	2021
**Mental disorders**																
Male	13474.2 (12061.3 ,15044.9)	14062.8 (12563.3 ,15750.1)	13124.7 (11752.6 ,14640.5)	13593.4 (12196.5 ,15162.4)	12713 (11529.6 ,14175.4)	13101.2 (11867.2 ,14633)	12226.3 (11064.1 ,13555.1)	12645.5 (11411.1 ,14064.2)	11789.7 (10479.5 ,13121.6)	12092.5 (10753.1 ,13522.3)	11385 (10182.2 ,12764)	11487.1 (10271.5 ,12816.9)	11055.2 (9751.4 ,12573.9)	10956.5 (9683.7 ,12438.9)	10908.4 (9249 ,12725.8)	10606.5 (9023.8 ,12295.8)
Female	16254.2 (14522.6 ,18439)	17262 (15367.3 ,19591.7)	15859.5 (14126.2 ,17899.3)	16645.5 (14759.4 ,18836)	15295.2 (13595.7 ,17430.5)	15996 (14202.7 ,18241)	14831.7 (13158.5 ,16850.9)	15375.6 (13634.9 ,17537.2)	14366.3 (12678.2 ,16283.1)	14720.7 (12941.9 ,16723.6)	13994.5 (12461.4 ,15789.7)	14074 (12516 ,15909.3)	13616.8 (11903.4 ,15596.5)	13448.3 (11767.4 ,15334.4)	13393.3 (11297.6 ,15589.4)	12894.3 (10906 ,14970.9)
Both	14894.6 (13347.8 ,16685.9)	15707.3 (14016.7 ,17656.7)	14591.1 (13054.1 ,16373.5)	15186.8 (13544.8 ,16985.6)	14147.8 (12636.2 ,15962.9)	14640.4 (13101.2 ,16539)	13763.7 (12343.3 ,15465.3)	14138 (12664.8 ,15911.4)	13398.8 (11855.8 ,15085.3)	13620.9 (12051.8 ,15356.7)	13120 (11754.3 ,14703.1)	13097.9 (11678 ,14714.1)	12864.2 (11306.2 ,14700)	12636.4 (11093.6 ,14416.5)	12758.2 (10825.6 ,14807)	12259.6 (10398.9 ,14225.4)
**Schizophrenia**																

Male	378.2 (319.7 ,444.4)	389.6 (329.8 ,457.5)	311.4 (264 ,364)	323.1 (274.4 ,378.2)	244.1 (205.7 ,285.3)	255.8 (216.2 ,299.3)	180 (151.2 ,214.8)	194.4 (163.6 ,230.8)	126.1 (103 ,154.7)	138.7 (114.5 ,168.8)	4728.6 (3977.5 ,5706.3)	95.8 (76.5 ,119)	53.5 (39.5 ,70.1)	63.6 (48.1 ,82.3)	34.2 (22.9 ,48.5)	40.8 (28.1 ,56.2)
Female	354 (299.6 ,414.5)	362.2 (306.5 ,425.3)	298 (253.3 ,347.3)	304.9 (258.3 ,355.9)	240.6 (203.5 ,280.8)	248.2 (209 ,290.7)	186.5 (157 ,220.8)	194.6 (163.7 ,230.2)	139.3 (115.6 ,168.4)	145.9 (121.5 ,176.9)	6279.7 (5277.9 ,7602)	108.2 (88.7 ,133.8)	70.9 (54.2 ,91.1)	77.6 (60.5 ,99)	48.5 (33.4 ,66)	53.8 (38.5 ,71.8)
Both	365.8 (309.5 ,430)	375.5 (317.7 ,440.6)	304.2 (258.6 ,355.1)	313.6 (266.1 ,366.4)	242.1 (204.1 ,283.2)	251.8 (212.8 ,294)	183.8 (155.1 ,218.3)	194.5 (163.6 ,230.6)	134.4 (110.7 ,163.1)	142.9 (118.8 ,173.5)	5759.9 (4853.1 ,6987.4)	103.5 (84.2 ,128.1)	65.8 (49.9 ,85.1)	73 (56.3 ,93.2)	44.8 (30.7 ,61.1)	50.2 (35.5 ,67.4)
**Depressive disorders**																
Male	4785 (3931 ,5823.8)	5211.2 (4297.9 ,6289.8)	4810.4 (4020.5 ,5782)	5207.8 (4378 ,6268)	4831.2 (4046.4 ,5696.4)	5077.6 (4181.9 ,6042.9)	4743.6 (3880.2 ,5742.4)	4981.2 (4040.2 ,6088.2)	4702.6 (3796.5 ,5735)	4778.2 (3837.9 ,5871.4)	331.7 (249.4 ,426.6)	4597.7 (3866.3 ,5518.4)	4800.3 (3841.6 ,5921)	4508 (3566.9 ,5531.8)	4880.3 (3492.1 ,6485.2)	4448.3 (3159.5 ,5879.5)
Female	7047 (5781.1 ,8478.9)	7629.9 (6220.6 ,9173.1)	6874.6 (5851.2 ,8226.7)	7449.5 (6279.5 ,8887.5)	6661.6 (5602.5 ,7790)	7087.2 (5934.7 ,8333.7)	6415.6 (5242 ,7756.4)	6775 (5475 ,8262.6)	6237.8 (5053.8 ,7595.4)	6419.5 (5159.8 ,7920.7)	372.6 (283.9 ,476.4)	6176.7 (5190.6 ,7436.7)	6371.7 (5115.5 ,7824.4)	6048.5 (4807.6 ,7399.3)	6536.1 (4694.3 ,8631.9)	5969.5 (4262.2 ,7887.1)
Both	5940.8 (4875.3 ,7196)	6454.5 (5302.8 ,7765.1)	5917.2 (4992.5 ,7098.1)	6378.1 (5391.9 ,7652.6)	5848.3 (4897 ,6845.7)	6146.1 (5115.2 ,7273.9)	5730.2 (4665.6 ,6932.8)	5961.8 (4821.5 ,7276.9)	5661.4 (4584.8 ,6892.4)	5732.6 (4599.6 ,7054.3)	358.9 (272.3 ,461)	5580.9 (4703.6 ,6699.2)	5910 (4749.3 ,7258.4)	5546.6 (4402.2 ,6781.3)	6112.9 (4382.7 ,8056)	5547.5 (3950.8 ,7300.6)
**Bipolar disorder**																
Male	645.3 (501.9 ,814.7)	634.7 (494.4 ,800.7)	604.6 (468.2 ,763.8)	561.9 (435.9 ,708.6)	536.4 (423.9 ,680.3)	512.9 (403.5 ,650.6)	479.8 (381.9 ,603.9)	443.7 (354.3 ,553.5)	403 (319.6 ,507)	376.1 (298.3 ,472.1)	2541.1 (1831.9 ,3329.6)	316.1 (238 ,407.1)	268.8 (197.6 ,350.8)	263 (194.6 ,342.1)	213.8 (152.9 ,298.3)	213.8 (152 ,297.7)
Female	716.7 (559.3 ,901.4)	686.9 (537.9 ,864.8)	666.4 (517.4 ,836.1)	601.3 (467.2 ,755.1)	580.7 (459.8 ,731.5)	545.5 (433.3 ,686.4)	521.7 (416.2 ,651.3)	468.6 (374 ,582.7)	443.5 (351.8 ,555.6)	395.4 (312.4 ,495.8)	4914.5 (3720.1 ,6312.3)	332.3 (250.2 ,424.9)	311.5 (232.3 ,404.8)	282.6 (209.3 ,365.5)	250.9 (181.5 ,344)	237.1 (170 ,326.2)

Both	681.8 (532.3 ,859.3)	661.6 (518 ,834.6)	637.7 (494.4 ,801.6)	582.5 (452.6 ,731.9)	561 (444.7 ,709.9)	530.3 (420 ,670.6)	504.6 (401.1 ,631.8)	457.3 (364.6 ,569.4)	428.3 (339.2 ,537.7)	387.3 (306.8 ,485.5)	4119.1 (3089.8 ,5282.2)	326.2 (246.1 ,417.9)	298.9 (222.1 ,388.6)	276.2 (204.6 ,357.9)	241.4 (174.4 ,332.3)	230.7 (165.1 ,318.3)
**Anxiety disorders**																
Male	3476.4 (2551.1 ,4727.4)	3843.6 (2808.3 ,5140.3)	3430.3 (2499.1 ,4731.9)	3685.6 (2702.3 ,5049.2)	3304.1 (2503 ,4462.2)	3528.5 (2708.7 ,4773.1)	3148.4 (2428.1 ,4030.3)	3371.2 (2627.3 ,4266.9)	2924.7 (2184.7 ,3790.7)	3150.4 (2353 ,4045.7)	0 (0,0)	2710.4 (1954.4 ,3520.7)	2019.3 (1427.5 ,2763)	2114.5 (1506.6 ,2863.8)	1459 (958.4 ,2150.4)	1477.9 (986.7 ,2162.2)
Female	5723.4 (4424.7 ,7534.8)	6388.8 (4933.5 ,8388.1)	5728.1 (4272 ,7795.2)	6149.5 (4599.4 ,8320.2)	5573.5 (4224.8 ,7462.8)	5985.4 (4555.7 ,7998.1)	5523.6 (4255.5 ,7188.3)	5793.8 (4458.9 ,7567.5)	5332.3 (4166.4 ,6812.2)	5559.6 (4336.7 ,7072.7)	0 (0,0)	5106.7 (3860.5 ,6556.1)	4289.1 (3158.4 ,5700.6)	4413.1 (3275.8 ,5857.1)	3556.6 (2413.8 ,4950.6)	3545.9 (2424.7 ,4932.6)
Both	4624.5 (3556.6 ,6141.5)	5151.9 (3964.8 ,6810.4)	4662.4 (3481.3 ,6331.4)	4971.9 (3720.5 ,6735.7)	4565.1 (3500.8 ,6093.6)	4834.9 (3694.9 ,6463.7)	4550 (3519.6 ,5846.3)	4695.6 (3649.7 ,6046)	4428.3 (3473.1 ,5612.3)	4551.4 (3557.5 ,5747.5)	0 (0,0)	4202.5 (3174.1 ,5350.6)	3622.3 (2663.7 ,4820.1)	3664.2 (2706.6 ,4901.5)	3020.4 (2063.3 ,4200.4)	2972.2 (2040.4 ,4130.1)
**Autism spectrum disorders**																
Male	976.1 (812.9 ,1144.7)	1006.8 (838.3 ,1186)	901.9 (746 ,1061.3)	946.5 (783.5 ,1122.3)	790.5 (650.5 ,934.2)	880.4 (723.8 ,1043.6)	678.2 (550.1 ,814.8)	768.8 (624.2 ,922.6)	546.2 (428.4 ,674.3)	666.6 (529.8 ,817.2)	0 (0,0.2)	562.5 (428.9 ,718.7)	312.6 (215.5 ,439)	459.2 (326.7 ,618.1)	222.2 (135.7 ,340.2)	371.1 (239.2 ,532.3)
Female	448.7 (369.7 ,529.6)	446.8 (367.9 ,529.2)	404.9 (332.2 ,480.4)	402.1 (330.3 ,477.5)	341.6 (279 ,408.6)	357.7 (292.7 ,424.6)	279 (223.2 ,338.9)	294 (235.5 ,355.6)	211.7 (162.5 ,265.4)	237.1 (184.1 ,295.7)	0 (0,0.2)	182.8 (134 ,239.5)	106.4 (70 ,152.5)	137.7 (93.1 ,195)	69.7 (41 ,109)	101.1 (62.2 ,151.8)
Both	706.6 (585.1 ,830.6)	719 (597 ,846.4)	635.4 (524 ,747.5)	662.3 (546.3 ,781.8)	541 (444.5 ,640.7)	602.5 (495.1 ,715)	442.6 (357.9 ,532.6)	509.2 (412.7 ,612.6)	337.3 (262.9 ,419.1)	416.8 (328.8 ,512.2)	0 (0,0.2)	326.1 (245.6 ,420.5)	166.9 (113.8 ,237.2)	242.4 (170.4 ,329.8)	108.7 (65.8 ,165.2)	176 (112.1 ,258.1)
**Attention-deficit/hyperactivity disorder**																
Male	233.5 (137.2 ,363.5)	228.6 (131.4 ,356.5)	112.7 (53 ,186.6)	113.7 (53.7 ,188.5)	47.5 (16.1 ,91.2)	46.6 (14.9 ,90.8)	13.1 (2.6 ,30.8)	13.9 (2.7 ,31.9)	0.9 (0.1 ,3.4)	1.1 (0.1 ,3.6)	0 (0,0)	0 (0,0.2)	0 (0,0)	0 (0,0)	0 (0,0)	0 (0,0)
Female	104.8 (57.3 ,167.9)	103.3 (57.5 ,166.8)	51.1 (22.9 ,87.9)	52.4 (23.2 ,90.7)	22.4 (6.5 ,45.5)	22.1 (6.3 ,45.1)	6.8 (1.1 ,16.4)	7.3 (1.2 ,17.8)	0.7 (0.1 ,2.5)	0.8 (0.1 ,2.8)	0 (0,0)	0 (0,0.2)	0 (0,0)	0 (0,0)	0 (0,0)	0 (0,0)
Both	167.7 (96.4 ,261.5)	164.2 (94.5 ,254.3)	79.7 (37.4 ,132.6)	81.7 (38.4 ,136.6)	33.6 (10.6 ,64.8)	33.6 (10.6 ,65.1)	9.4 (1.7 ,23.1)	10.3 (1.9 ,24.7)	0.8 (0.1 ,2.8)	0.9 (0.1 ,3.1)	0 (0,0)	0 (0,0.2)	0 (0,0)	0 (0,0)	0 (0,0)	0 (0,0)
**Idiopathic developmental intellectual disability**																

Male	569.9 (241.3 ,953.5)	396.8 (146.7 ,697.4)	468.1 (192.2 ,801.5)	319.8 (116.3 ,571.4)	388.1 (153.8 ,672)	270.9 (101.9 ,478.7)	301.2 (115.8 ,531.6)	232.4 (90.8 ,411.3)	256.2 (93.5 ,461.2)	180.7 (66 ,333.1)	3555.9 (2896.2 ,4318.7)	143.8 (46.8 ,268.4)	173.5 (61.4 ,318.9)	114.5 (36.6 ,216.3)	146.3 (56 ,265.3)	649.7 (564.2 ,741.3)
Female	624 (299.7 ,960.6)	519 (243.3 ,798)	505.8 (236.8 ,794.3)	426.1 (196.1 ,668.3)	423.2 (196.2 ,669.4)	361 (163.1 ,563.8)	324.4 (143.8 ,520.3)	306.1 (142.4 ,481.6)	270.7 (122 ,433.3)	241.6 (111.8 ,378.7)	2673.9 (2189.1 ,3243.7)	187.1 (83.9 ,293.4)	178.1 (82.8 ,282.8)	142.3 (60 ,225.7)	150.3 (72.5 ,237.5)	638.9 (551.3 ,738)
Both	597.5 (273.6 ,955.1)	459.6 (196.5 ,751.2)	488.3 (220.4 ,797.6)	375.3 (159.2 ,622.8)	407.6 (180.6 ,672.4)	318.8 (134.4 ,526.2)	314.9 (131.1 ,527.4)	272.7 (118.9 ,447.4)	265.3 (114 ,442.8)	216.1 (91.7 ,360.1)	2969.5 (2417.8 ,3596.7)	170.7 (70.3 ,283.2)	176.7 (75.7 ,294.4)	133.3 (50.6 ,222.7)	149.3 (68.4 ,244.8)	641.9 (555.7 ,738.7)
**Other mental disorders**																
Male	3170.1 (2497.7 ,3982.4)	3168.4 (2496.1 ,3982.5)	3191.1 (2576.8 ,3972.8)	3182.3 (2561.6 ,3977.5)	3218.6 (2628.8 ,3967.8)	3212.1 (2614 ,3962.7)	3268.7 (2670.6 ,3978.3)	3266.4 (2669.7 ,3982.4)	3364.3 (2764 ,4080.1)	3365.5 (2756.6 ,4079.5)	83.8 (65.6 ,105)	3561.8 (2903.9 ,4334.4)	3868.6 (3117.1 ,4791.2)	3875.8 (3118.1 ,4787.4)	4361.8 (3480.2 ,5589.1)	89.2 (27 ,172.9)
Female	2257.6 (1773.4 ,2822.7)	2260.1 (1777.1 ,2827.7)	2286.9 (1833.6 ,2862.3)	2295.8 (1841.6 ,2862.8)	2327.6 (1907.2 ,2870.7)	2336.6 (1909.5 ,2887.5)	2388 (1958.4 ,2906.1)	2403.4 (1969.2 ,2932)	2488.3 (2043.9 ,3029)	2513.1 (2058.4 ,3064.9)	100.4 (81.3 ,124.1)	2707.6 (2199.1 ,3283.8)	2964.8 (2381.7 ,3662.9)	3012.4 (2396.2 ,3722.9)	3426.1 (2675.2 ,4533.7)	111.4 (47.2 ,175.1)
Both	2703.8 (2137.4 ,3384.2)	2701.5 (2134.6 ,3383.4)	2706.3 (2182.2 ,3373.5)	2719.5 (2193.6 ,3397.4)	2723.5 (2230.1 ,3342.6)	2746.6 (2247.7 ,3370.7)	2749 (2252.4 ,3342.1)	2794.7 (2288.9 ,3410.2)	2817.2 (2309 ,3417.1)	2869.8 (2352.6 ,3485.6)	94.8 (76.1 ,117.5)	3029.9 (2463.7 ,3682.8)	3230.3 (2593.9 ,3987.9)	3293.7 (2644.5 ,4054.2)	3665.2 (2884.7 ,4815.4)	105.3 (41.7 ,175)
**Substance use disorders**																
Male	3811.3 (2976.7 ,4825.6)	3418.9 (2718.1 ,4301.8)	3006 (2446.1 ,3762.6)	2844.5 (2317 ,3527.9)	2418 (1867.8 ,3135.9)	2389.2 (1842.7 ,3072)	2134.9 (1667.1 ,2648.5)	2041.5 (1591.5 ,2540.7)	1935.3 (1445.6 ,2436)	1933.7 (1442.8 ,2391.6)	2002.8 (1547.6 ,2486.7)	2010.3 (1577.2 ,2486.9)	2274.8 (1603.7 ,2989.6)	2390.6 (1702.1 ,3165.2)	3077.1 (2146.7 ,4199.7)	3260.2 (2267.2 ,4409.8)
Female	1266 (984 ,1641.9)	1033.5 (827.9 ,1317.7)	1106.7 (882 ,1380.7)	891.3 (718.5 ,1104.7)	982.8 (746.3 ,1274.9)	820 (628 ,1063.2)	1033.6 (783.6 ,1292.3)	806.1 (618.1 ,1000.8)	1051.3 (777.4 ,1360.4)	877 (655.1 ,1116.7)	1160.2 (889.9 ,1467.9)	999 (772.1 ,1252.9)	1346.5 (940.8 ,1825.4)	1234.9 (888.7 ,1640.6)	1693.3 (1205.4 ,2341.4)	1594.4 (1143.9 ,2170)

Both	2510.8 (1969 ,3202.5)	2192.8 (1752.3 ,2778.1)	1987.6 (1605.5 ,2480.9)	1824.8 (1497.4 ,2257.2)	1620.5 (1253.4 ,2102.2)	1554.8 (1195.2 ,1992.9)	1485.1 (1166.9 ,1843.6)	1366.1 (1065.9 ,1682.3)	1383.3 (1037.4 ,1761.5)	1319.2 (990.7 ,1644.6)	1442.6 (1115.5 ,1801.6)	1380.6 (1081.5 ,1712.5)	1619.2 (1134.8 ,2167.3)	1611.4 (1156.7 ,2126.7)	2047 (1450.9 ,2811.8)	2056.5 (1459.4 ,2782.5)
**Alcohol use disorders**																
Male	3543.1 (2691.4 ,4545)	3138.9 (2409.5 ,4006)	2802.6 (2242.8 ,3553)	2648.9 (2130.3 ,3313.9)	2256.2 (1714.1 ,2969.2)	2241.9 (1696.7 ,2923.1)	2001.3 (1530.8 ,2516.7)	1923.7 (1480.6 ,2411)	1819.3 (1322.8 ,2325.5)	1827.2 (1330.7 ,2293.5)	1889.8 (1435.9 ,2370.2)	1897.9 (1467.9 ,2373.4)	2158.4 (1486.6 ,2876)	2265.5 (1581 ,3050)	2957.9 (2029.4 ,4083.8)	3131.3 (2142.1 ,4288.1)
Female	1029.2 (752.7 ,1404.4)	772.9 (562.1 ,1055)	908.8 (684.5 ,1177.8)	699.8 (529.9 ,903.4)	807.5 (581.8 ,1097.9)	668.2 (483.6 ,909.9)	880.1 (642.7 ,1130.9)	676.3 (492.4 ,869.1)	912.3 (637 ,1207.2)	752.6 (533.6 ,995.5)	1026.2 (757.4 ,1319.2)	863.9 (643.5 ,1114.9)	1208 (803.1 ,1689.2)	1075.7 (729.1 ,1491)	1542.2 (1054.1 ,2194.9)	1416.9 (983.3 ,1998.6)
Both	2258.6 (1707.5 ,2942.2)	1922.7 (1466.7 ,2490.1)	1787.2 (1404.2 ,2279.1)	1631.4 (1304.2 ,2057.9)	1451.2 (1090.1 ,1930.8)	1405.1 (1050.6 ,1848.5)	1339.7 (1017.8 ,1700)	1241.8 (939.1 ,1559.7)	1252.8 (901.4 ,1638.1)	1202.3 (873.7 ,1535.8)	1315.6 (990 ,1675.5)	1254.1 (962.7 ,1585.6)	1487.2 (1003.7 ,2038.4)	1463.4 (1007.3 ,1988.6)	1904.1 (1320.1 ,2684)	1892.5 (1304.2 ,2625.3)
**Drug use disorders**																
Male	279.5 (225.8 ,345.4)	290.1 (239.5 ,350.6)	209.7 (168.9 ,255.3)	201.4 (164.3 ,243.9)	165.5 (134.4 ,199.5)	150.8 (124.2 ,177.7)	136.2 (112 ,161)	120.2 (100.6 ,141.7)	118.1 (98.1 ,143.6)	108.5 (91.6 ,128.7)	115 (97.8 ,136.1)	114.6 (99.2 ,133.1)	118.8 (99.7 ,141.3)	127.9 (110.7 ,148.8)	122.6 (98.4 ,153.4)	133.2 (111 ,159.6)
Female	239.5 (191.8 ,296.6)	263.3 (216.6 ,315.2)	199.7 (162.4 ,239.2)	193.1 (159.6 ,227.2)	176.6 (144.7 ,218.7)	153 (127.9 ,184.5)	154.8 (127.6 ,191)	130.7 (109.7 ,156.8)	140.2 (113.9 ,171.6)	125.3 (104.6 ,150.2)	135.4 (113.7 ,162)	136.4 (117.6 ,158.9)	140.2 (118.1 ,167)	161.1 (140.2 ,186.5)	153.4 (124.9 ,185.2)	180.3 (153 ,212.3)
Both	259.1 (209.3 ,320.7)	276.3 (229.3 ,334.4)	204.3 (166.3 ,243.1)	197 (162.7 ,232.3)	171.6 (141.2 ,208)	152 (126 ,180.1)	147.2 (121.8 ,178.1)	125.9 (106.1 ,149.5)	131.9 (109 ,161.1)	118.3 (100.1 ,141)	128.5 (108.7 ,152.8)	128.2 (110.7 ,148.7)	133.9 (113.6 ,158.7)	150.3 (131.4 ,172.9)	145.6 (119.4 ,176.5)	167.2 (143.2 ,196.3)

Note: AAPC-average annual percentage changes; 95% UI-95% uncertainty interval

eTable 4. Global YLDs of Mental Disorders and Substance Use Disorders per 100 000 People, by Sexes, in 1990,2021

Cause	Rate (95% UI)															
	Age 60-64y		Age 65-69y		Age 70-74y		Age 75-79y		Age 80-84y		Age 85-89y		Age 90-94y		Age 95+y	
	1990	2021	1990	2021	1990	2021	1990	2021	1990	2021	1990	2021	1990	2021	1990	2021
**Mental disorders**																
Male	1940.1 (1444.1 ,2508.3)	2067.7 (1531.3 ,2700.6)	1848 (1388.6 ,2356.5)	1946.7 (1455.9 ,2473.1)	1740 (1297.3 ,2195.4)	1821.1 (1361.7 ,2296)	1620.8 (1171.2 ,2068.9)	1697.5 (1241.5 ,2159.1)	1506.2 (1097.4 ,1942.3)	1560 (1144.8 ,2000.3)	1395.9 (1028 ,1798.8)	1413.1 (1050 ,1816.4)	1298.2 (952.8 ,1706.5)	1282 (948.1 ,1668.5)	1215.5 (845.5 ,1652)	1170.5 (824.3 ,1565.5)
Female	2406.2 (1731.4 ,3168.6)	2577.2 (1859.6 ,3400.8)	2298.3 (1673.3 ,3000.5)	2421.6 (1762.7 ,3154.5)	2156.7 (1588.1 ,2787.5)	2266.4 (1686.8 ,2923.2)	2039.1 (1460.2 ,2647)	2111.1 (1524.1 ,2766.4)	1915.3 (1376.3 ,2497.8)	1953.5 (1416.4 ,2544.1)	1807.1 (1332.9 ,2353.5)	1793.8 (1333.9 ,2329.8)	1700.5 (1231.4 ,2230.4)	1647.4 (1198.1 ,2161.2)	1608.2 (1114.7 ,2186)	1509.7 (1050.1 ,2055.5)
Both	2178.3 (1591.4 ,2839.5)	2329.6 (1699.3 ,3052.1)	2089.4 (1531.8 ,2701.5)	2194.6 (1619.1 ,2840.3)	1971.5 (1455.7 ,2540.2)	2057.9 (1531.9 ,2638.5)	1867.6 (1344.3 ,2416.6)	1923.6 (1390.1 ,2487.7)	1761.7 (1275.7 ,2292.8)	1788.8 (1303.3 ,2320.3)	1669.3 (1228.1 ,2167.3)	1650.2 (1227.6 ,2128.9)	1582.3 (1144.5 ,2085)	1528.3 (1116.7 ,1991.7)	1507.8 (1049.6 ,2045.2)	1415.6 (987.2 ,1915.7)
**Schizophrenia**																
Male	232.7 (169.5 ,296.9)	239.5 (175.9 ,305.8)	187.1 (135.9 ,240.8)	194.1 (143.5 ,248.7)	142.6 (105.7 ,182)	149.7 (109.6 ,192.7)	102.5 (73.9 ,131.4)	110.4 (79.3 ,141.6)	69.7 (48.9 ,91.9)	76.6 (54 ,99.5)	45 (31.5 ,59.9)	51.4 (36.5 ,67.6)	28.1 (19.4 ,38.8)	33.2 (23.3 ,45.6)	17.8 (11 ,26.2)	21 (13.7 ,30.6)
Female	214.1 (158.4 ,272.1)	218.5 (158.8 ,280.2)	176.9 (128.5 ,224.9)	180 (131.4 ,231.5)	139.6 (103.2 ,179.7)	143.2 (106 ,181.9)	105.6 (77.1 ,134.9)	109.2 (80.8 ,138.5)	76.6 (55.2 ,98.8)	79.5 (57.8 ,101.8)	53.4 (38.2 ,70.4)	57.1 (41.4 ,74.9)	36.6 (26.3 ,48.9)	39.7 (28.5 ,52.9)	24.4 (16.1 ,34.6)	26.7 (17.9 ,37.5)
Both	223.2 (164.8 ,284.2)	228.7 (167.1 ,292.6)	181.6 (132.9 ,230.9)	186.8 (137.8 ,239.5)	141 (104.1 ,180.7)	146.3 (107.7 ,187.3)	104.3 (76 ,132.7)	109.8 (81 ,140.6)	74 (52.5 ,96.2)	78.3 (56 ,100.7)	50.5 (35.9 ,66.6)	55 (39.5 ,72.4)	34.1 (24.4 ,45.8)	37.6 (26.9 ,50.3)	22.7 (14.9 ,32.1)	25.1 (16.6 ,35.5)
**Depressive disorders**																
Male	747.6 (505 ,1035.8)	829.9 (559.8 ,1161.7)	748 (510.1 ,1013.5)	818.2 (559.2 ,1111)	751.4 (504.9 ,1017.4)	13.1 (5.4 ,24.1)	737.5 (498 ,1025.3)	11.7 (5 ,21.2)	728 (496.1 ,1033.5)	739.1 (502.6 ,1050.8)	721.1 (510 ,997.3)	696 (492.4 ,963.3)	715.6 (485 ,1019.8)	664.6 (455.1 ,957.3)	705.2 (425 ,1068.3)	634.2 (382.4 ,956.1)
Female	1131.8 (767.3 ,1572.2)	1236.7 (830.8 ,1695.7)	1097.7 (748.3 ,1490)	1190.3 (814.3 ,1609.4)	1057.6 (717 ,1436.5)	16.7 (7.8 ,28)	1019.6 (679.8 ,1426.5)	14.8 (7.1 ,24.3)	986.3 (670.7 ,1395.8)	1006.3 (680.6 ,1432.5)	978.6 (688.5 ,1346.5)	947.6 (670 ,1318.8)	971.6 (664.4 ,1391.2)	905.7 (621.6 ,1294.7)	969.3 (593.3 ,1459.4)	868.4 (521.8 ,1308)
Both	943.9 (640.4 ,1309.9)	1039 (700.9 ,1439.5)	935.5 (637.1 ,1272.2)	1012.4 (693.3 ,1372.6)	921.5 (621.8 ,1247.1)	15 (6.7 ,26)	904 (604 ,1263.8)	13.4 (6.2 ,22.8)	889.3 (605.2 ,1259.7)	894.5 (607 ,1271.1)	892.3 (628.9 ,1230.9)	852.7 (602.2 ,1181.7)	896.4 (612.1 ,1278.5)	827.1 (567.6 ,1182.5)	901.8 (550.1 ,1357.1)	803.4 (482.9 ,1210.4)
**Bipolar disorder**																

Male	133.3 (82.6 ,198.8)	131.2 (80.5 ,198.3)	122.5 (76.5 ,181.3)	113.8 (71.8 ,169)	106.3 (68.1 ,156.1)	101.6 (65.2 ,151.9)	92.9 (58.5 ,137.5)	85.9 (54.5 ,126)	76.1 (46.8 ,110.9)	71.1 (43.7 ,102.3)	61.1 (37.1 ,90.5)	58.3 (35.1 ,86.5)	48.4 (28.8 ,71.9)	47.3 (28.3 ,69.5)	37.5 (22.4 ,56.5)	37.6 (22.4 ,55.9)
Female	146.5 (91.4 ,219)	139.8 (87.4 ,208.4)	133.9 (84.3 ,198.7)	120.3 (76.7 ,177.2)	114.4 (72.7 ,168.3)	107 (68.6 ,158.4)	100.7 (64.1 ,146.2)	89.9 (56.9 ,131.5)	83.5 (50.6 ,119.3)	74.1 (45.2 ,107.2)	68.1 (41.2 ,100.1)	60.6 (37 ,89.4)	55.4 (32.8 ,82.4)	50.2 (30.1 ,74.5)	43.4 (26.3 ,63.9)	40.9 (24.4 ,60.4)
Both	140.1 (87.2 ,209)	135.6 (84.1 ,204.3)	128.6 (80.7 ,190.8)	117.2 (74.3 ,173.2)	110.8 (70.3 ,162.6)	104.5 (66.9 ,155.6)	97.5 (62.1 ,142.5)	88.1 (55.7 ,128.9)	80.7 (48.9 ,116.3)	72.9 (44.5 ,105.1)	65.8 (39.9 ,97.2)	59.7 (36.4 ,88.1)	53.4 (31.8 ,79.2)	49.3 (29.3 ,72.8)	41.9 (25.4 ,62.2)	40 (23.9 ,59.2)
**Anxiety disorders**																
Male	397.2 (251.8 ,597.9)	439 (278.7 ,658.5)	384.7 (241.9 ,573.7)	413.4 (261.1 ,609.7)	362.6 (239.7 ,526.5)	387.2 (254.1 ,556)	337.7 (227 ,472.2)	361.1 (242.1 ,510.2)	306.3 (197.8 ,451.7)	330.1 (210.1 ,481.4)	259.4 (164.1 ,387.8)	277.3 (175.9 ,408.8)	201.5 (127 ,296)	211.3 (133.4 ,303.9)	141.8 (84.1 ,217.9)	143.9 (85.9 ,222.6)
Female	646.8 (428.1 ,940)	719.4 (476.3 ,1038.9)	636.9 (414.1 ,949.3)	681.2 (444.7 ,1007.8)	607.6 (402.5 ,879.9)	649.8 (432.9 ,942.3)	589.8 (406.4 ,842)	615.2 (421.5 ,875.2)	555.9 (382.1 ,788.8)	576.8 (390.2 ,813.9)	498.2 (329.8 ,707.2)	515.1 (339.1 ,730.4)	423.3 (272 ,600.9)	433.3 (280.6 ,606.1)	341.3 (215.2 ,492.4)	338.6 (215.3 ,485.7)
Both	524.7 (343.6 ,772.3)	583.1 (383.3 ,855.6)	519.9 (334.9 ,771.7)	553.2 (359.3 ,810.8)	498.7 (331.1 ,719.8)	526.8 (351.6 ,756.2)	486.4 (335.7 ,696.5)	500 (343.1 ,704.9)	462.2 (319 ,650.2)	473.6 (321.4 ,667)	418.2 (272.7 ,593.5)	425.4 (274.4 ,602.2)	358.1 (229.3 ,502.5)	361 (232.4 ,506.6)	290.3 (182.9 ,423.3)	284.6 (180.5 ,409.6)
**Autism spectrum disorders**																
Male	173.9 (120.4 ,240.8)	179.3 (123.3 ,250.8)	158 (109 ,219.5)	165.8 (113.7 ,231.4)	135.9 (93.5 ,188.1)	151.5 (103.7 ,211.3)	114.4 (78.9 ,159.6)	129.9 (89.2 ,182)	90.5 (61.7 ,127)	110.4 (75.6 ,155.4)	68.2 (45.9 ,97.2)	91.4 (62.1 ,129.3)	49.9 (32 ,75)	73.1 (48 ,106.1)	34.8 (20.5 ,54.8)	58 (36.4 ,88.7)
Female	79.3 (55.4 ,109.4)	78.8 (54.4 ,109.2)	70.6 (49.1 ,97.5)	69.9 (48 ,96.1)	58.6 (40.2 ,81.3)	61.2 (41.5 ,84.6)	47.1 (32.4 ,65.9)	49.4 (33.6 ,69.2)	35.2 (23.8 ,49.4)	39.2 (26.9 ,55.2)	25.1 (16.5 ,35.9)	29.8 (20.3 ,43.2)	17.4 (10.9 ,26)	22.1 (14.4 ,32.9)	11.6 (6.5 ,18.7)	16.3 (9.7 ,25.8)
Both	125.6 (87 ,174.1)	127.6 (87.8 ,178.2)	111.2 (76.1 ,153.9)	115.7 (79.5 ,161.7)	93 (63.6 ,129.2)	103.5 (70.5 ,143.9)	74.7 (51.5 ,104.2)	85.9 (58.8 ,120.1)	56 (38.1 ,78.4)	69 (47.1 ,97.5)	39.5 (26.3 ,56.1)	53.1 (36.2 ,75.6)	26.9 (17.1 ,40.3)	38.7 (25.6 ,57)	17.5 (10.2 ,28.1)	27.9 (17.1 ,42.8)
**Attention-deficit/hyperactivity disorder**																
Male	2.7 (1.4,4.6)	2.6 (1.3 ,4.6)	1.3 (0.6,2.4)	1.3 (0.6,2.4)	0.5 (0.2,1.2)	0.5 (0.2,1.2)	0.2 (0,0.4)	0.2 (0,0.4)	0 (0,0)	0 (0,0.1)	0 (0,0)	0 (0,0)	0 (0,0)	0 (0,0)	0 (0,0)	0 (0,0)

Female	1.2 (0.6,2.2)	1.2 (0.6 ,2.1)	0.6 (0.2,1.1)	0.6 (0.2,1.2)	0.3 (0.1,0.6)	0.3 (0.1,0.6)	0.1 (0,0.2)	0.1 (0,0.2)	0 (0,0)	0 (0,0)	0 (0,0)	0 (0,0)	0 (0,0)	0 (0,0)	0 (0,0)	0 (0,0)
Both	1.9 (1,3.4)	1.9 (0.9,3.3)	0.9 (0.4,1.7)	0.9 (0.4,1.8)	0.4 (0.1,0.9)	0.4 (0.1,0.9)	0.1 (0,0.3)	0.1 (0,0.3)	0 (0,0)	0 (0,0)	0 (0,0)	0 (0,0)	0 (0,0)	0 (0,0)	0 (0,0)	0 (0,0)
**Idiopathic developmental intellectual disability**																
Male	24.5 (9.6 ,45.3)	17.9 (6.6 ,33.6)	20.5 (7.8 ,37.6)	14.8 (5.6 ,27.7)	17.4 (6.9 ,32)	794.9 (539.7 ,1083.1)	14 (5.6 ,26)	776.8 (527.1 ,1096.3)	12.3 (5.1 ,22.9)	9.2 (4.1 ,16.8)	10.5 (4.6 ,19.6)	7.5 (3.3 ,13.8)	9.2 (4.1 ,17.1)	6.1 (2.5 ,11.2)	8.2 (3.9 ,14.9)	4.9 (2 ,8.9)
Female	25.7 (11.2 ,45.1)	22.3 (9.7 ,38.5)	21.4 (9.3 ,37.5)	18.8 (8.4 ,32.3)	18.5 (8.1 ,31.7)	1128 (771.6 ,1527)	15.1 (6.8 ,25.8)	1071 (728.7 ,1499)	13.5 (6.7 ,22.3)	12.3 (6.3 ,20.2)	11.5 (6 ,18.4)	10 (5.3 ,16.2)	9.8 (5.6 ,15.6)	7.8 (4.2 ,12.6)	8.5 (4.8 ,13.1)	6.3 (3.4 ,9.9)
Both	25.1 (10.4 ,45.2)	20.2 (8 ,36.1)	21 (8.5 ,37.6)	16.9 (7 ,30.1)	18 (7.4 ,31.7)	972 (662.4 ,1316.7)	14.7 (6.2 ,25.9)	937.6 (638.1 ,1318.2)	13 (6 ,22.6)	11 (5.4 ,18.8)	11.2 (5.7 ,18.7)	9 (4.5 ,15.4)	9.7 (5.1 ,16)	7.3 (3.7 ,12.2)	8.4 (4.6 ,13.5)	5.9 (3.1 ,9.6)
**Other mental disorders**																
Male	228.3 (147.8 ,343.7)	228.3 (148.3 ,343)	225.7 (147.6 ,338.1)	225.1 (146.7 ,335.4)	223.1 (145.5 ,329.6)	222.6 (145.1 ,329.3)	221.7 (145 ,331.4)	221.5 (146.1 ,331.3)	223.3 (148.3 ,330.9)	223.5 (148.7 ,330.6)	230.6 (156.1 ,339.6)	231.3 (155.8 ,339.2)	245.5 (164.8 ,358.5)	246.3 (166 ,358.1)	270.2 (173.6 ,397.6)	270.9 (174.2 ,398.3)
Female	160.8 (103.6 ,241.9)	160.6 (103 ,242.3)	160.4 (105.4 ,237.5)	160.6 (104.3 ,238.5)	160.1 (104.3 ,238.5)	160.3 (104.5 ,239.8)	161.2 (106.1 ,239.4)	161.5 (106.2 ,241)	164.2 (108.7 ,241.4)	165.2 (109.5 ,243.1)	172.3 (114.8 ,252.5)	173.5 (116 ,253.2)	186.3 (124.6 ,272.3)	188.5 (126.3 ,273.4)	209.6 (134 ,308.6)	212.6 (137.5 ,314.7)
Both	193.8 (125.2 ,290)	193.5 (125 ,290.4)	190.7 (124.6 ,283.6)	191.4 (124.3 ,284.1)	188.1 (122.3 ,279)	189.5 (123.9 ,281.6)	186 (121.7 ,276.6)	188.7 (123.6 ,281.8)	186.4 (123.7 ,274.5)	189.6 (125.8 ,277.9)	191.8 (129.1 ,281)	195.3 (131.5 ,286.1)	203.7 (136.5 ,298)	207.3 (139.3 ,300.8)	225.1 (144.6 ,331.3)	228.8 (148.1 ,336.5)
**Substance use disorders**																
Male	392.4 (250.3 ,548.6)	355.9 (232.6 ,498.6)	304 (204.9 ,418.7)	287.1 (194.2 ,394.4)	242 (163.8 ,338.9)	235.1 (159 ,331)	209.4 (144.1 ,286.3)	197.1 (134.9 ,270)	186 (122.7 ,257.5)	183.7 (123.4 ,256.8)	187.7 (128 ,260.2)	188.1 (129.6 ,260.5)	206.9 (136.9 ,301.7)	218.7 (145.7 ,315.7)	268 (166.6 ,403.5)	285.5 (181.9 ,419.9)
Female	157.6 (104.4 ,218.7)	141.5 (97.6 ,192.8)	136.3 (94.1 ,184.7)	114.9 (80 ,154.6)	121.6 (82.4 ,165)	100.7 (68.8 ,136.4)	121 (83.5 ,165.8)	94.6 (65.8 ,128)	117.8 (78.7 ,165.7)	99.1 (66.3 ,137.9)	123.4 (86.4 ,169.7)	109.9 (78.7 ,149.4)	137 (91.5 ,198.8)	132.5 (92.8 ,184.4)	164.9 (107.8 ,243.3)	162.5 (111.9 ,232.9)

Both	272.4 (176.7 ,382.4)	245.7 (164.6 ,341.5)	214.1 (144.4 ,290.6)	197.2 (135.2 ,266.1)	175.1 (120 ,243)	163.6 (111.9 ,225.9)	157.2 (108.7 ,213.6)	141.1 (96.8 ,191.4)	143.4 (95.3 ,199.7)	134.5 (89.2 ,187.4)	145 (100.4 ,200.1)	139.4 (98.2 ,191.3)	157.6 (105.3 ,228.7)	160.6 (110 ,227.9)	191.3 (123.8 ,284.7)	196.6 (129.7 ,283.2)
**Alcohol use disorders**																
Male	338.2 (210.3 ,487.3)	300.3 (188.4 ,430.4)	262.8 (171.8 ,371.2)	249.1 (164.1 ,352.5)	207.4 (137.6 ,299.6)	206.6 (137 ,298.5)	180.2 (120.3 ,252.4)	173.6 (116.1 ,242.9)	160.8 (102.3 ,226.5)	161.6 (104.1 ,230.5)	163.8 (109.7 ,231.1)	164.5 (111.8 ,232.5)	183.3 (115.8 ,275.6)	192.5 (122.2 ,287)	245.1 (147.1 ,376.3)	259.8 (158.6 ,391.5)
Female	97.3 (57.5 ,144.5)	72.8 (43.6 ,107.4)	84.7 (54.4 ,121.2)	65 (41.7 ,92.8)	74 (47.5 ,110.7)	61 (39.6 ,91.4)	79.1 (51.4 ,114)	60.6 (39.5 ,86.6)	80.3 (50.5 ,118.5)	66 (41.2 ,96.3)	88.3 (57.2 ,129.2)	73.9 (48.2 ,107.8)	101.4 (62.6 ,157.2)	89.9 (56.1 ,135.7)	126.4 (76.3 ,197.9)	115.5 (69.3 ,179.8)
Both	215.1 (132.9 ,312.4)	183.4 (114.7 ,263.6)	167.3 (108.5 ,238)	153 (100.4 ,216.3)	133.3 (87.1 ,195.2)	129.2 (85.8 ,188.2)	120.5 (79.8 ,169.5)	111.8 (73.9 ,157)	110.5 (70.5 ,157.7)	106 (68.5 ,150.8)	113.6 (74.9 ,162)	108.1 (72.6 ,155.8)	125.5 (77.4 ,192)	123.3 (77.4 ,186.2)	156.7 (94.4 ,244.7)	155.5 (93.9 ,236)
**Drug use disorders**																
Male	54.1 (35.4 ,75.6)	55.6 (37.8 ,74.5)	41.2 (27.1 ,56.5)	38 (26.1 ,51.2)	34.6 (23 ,49.5)	28.6 (19.7 ,39.1)	29.2 (19.8 ,40)	23.5 (16.5 ,31.3)	25.3 (17.2 ,35.6)	22.1 (15.7 ,30)	23.9 (16.5 ,31.9)	23.6 (16.7 ,30.8)	23.6 (16.3 ,31.8)	26.2 (18.7 ,34.3)	22.9 (15.8 ,30.9)	25.7 (18.2 ,33.9)
Female	60.3 (39.5 ,85.1)	68.7 (46.3 ,94.3)	51.6 (33.4 ,71.8)	49.9 (33.9 ,68.1)	47.5 (30.6 ,69.2)	39.7 (26.2 ,55.9)	41.9 (28.3 ,58.1)	34.1 (23.4 ,46.2)	37.5 (25.2 ,52.9)	33.1 (22.5 ,45.6)	35.1 (23.9 ,47.6)	36 (25.8 ,47.2)	35.6 (24.5 ,47.7)	42.6 (30.2 ,55.7)	38.5 (27 ,52.4)	47 (33.7 ,61.5)
Both	57.3 (37.5 ,80.3)	62.3 (42.2 ,84.6)	46.8 (30.5 ,64.2)	44.2 (30.3 ,59.8)	41.8 (27.5 ,60.7)	34.5 (23.3 ,48.2)	36.7 (24.6 ,50.4)	29.3 (20.2 ,39.3)	32.9 (22.2 ,46.3)	28.5 (19.7 ,38.8)	31.4 (21.4 ,42.4)	31.3 (22.1 ,40.9)	32.1 (22.1 ,42.7)	37.2 (26.4 ,48.4)	34.5 (24.2 ,46.5)	41.1 (29.6 ,53.6)

Note: YLDS-years lived with disability; AAPC-average annual percentage changes; 95% UI-95% uncertainty interval

eTable 5. Age Standardised Prevalence and AAPC of Mental disorders, Substance use disorders in people aged ≥60 years at 21 GBD regions, from 1990 to 2021

Regions \ Causes	Rate (95% UI/95%CI)																							
	Mental disorders			Substance use disorders			Schizophrenia		Depressive disorders		Bipolar disorder		Anxiety disorders		Autism spectrum disorders		Attention-defic/ Hyperactivity disorder		Idiopathic developmental intellectual disability		Other mental disorders		Alcohol use disorders		Drug use disorders
	1990	2021	AAPC,%	1990	2021	AAPC,%	1990	2021	1990	2021	1990	2021	1990	2021	1990	2021	1990	2021	1990	2021	1990	2021	1990	2021	1990	2021
**Andean Latin America**																									
Male	12678.5 (11008.4 ,14707.3)	13307.6 (11391.1 ,15685.4)	0.18 (0.14 ,0.25)	3558.3 (2609.6 ,4713.2)	2299.3 (1697.8 ,3028.7)	-1.04 (-1.23 ,-0.86)	252.8 (197.2 ,321.5)	262.5 (206.7 ,329.5)	4009.9 (3178.4 ,5041.5)	4157.7 (3208.3 ,5293.7)	815.2 (599.7 ,1085.5)	815.1 (600.2 ,1085.5)	4088.6 (2736.2 ,5944.3)	4623.6 (3023 ,6887.9)	591.9 (481.9 ,711.2)	664.8 (541 ,796.3)	216.8 (119.3 ,353)	216.8 (119.3 ,353)	207.9 (35.6 ,394.7)	137.3 (18.1 ,287.3)	3153.2 (2516.2 ,3930.7)	3153.2 (2516.2 ,3930.7)	3423.6 (2477.3 ,4586.2)	2154.7 (1556 ,2888.4)	139.8 (111.4 ,176.2)	148 (118.3 ,185.7)
Female	15419 (13023.5 ,18334.3)	16745.9 (13894.3 ,20216.2)	0.29 (0.21 ,0.35)	1047.5 (754.9 ,1426.5)	849.1 (617.4 ,1148.7)	-0.66 (-0.68 ,-0.64)	214.1 (167.3 ,270.4)	222.1 (174.3 ,280.7)	5596.2 (4402.7 ,7137.1)	5934.9 (4558.9 ,7602.5)	833.1 (610.3 ,1112.5)	833.1 (610.1 ,1112)	6739.6 (4640.3 ,9635.9)	7901.8 (5312.5 ,11577.4)	292.4 (234.4 ,352.6)	320 (258.7 ,390)	99.3 (50.1 ,161.2)	99.3 (50.1 ,161.2)	226.3 (67.7 ,384.3)	165.2 (32.9 ,291.9)	2318.2 (1855.3 ,2904.7)	2318.2 (1855.3 ,2904.7)	958.6 (664.5 ,1342.7)	758.2 (527.4 ,1055.2)	89.8 (69.8 ,112.8)	91.6 (71.6 ,114.7)
Both	14096 (12155 ,16568.7)	15112.4 (12772.8 ,17975.6)	0.27 (0.21 ,0.35)	2267.1 (1674.1 ,2989)	1539.3 (1152.2 ,2001.8)	-1.00 (-1.14 ,-0.86)	233 (183 ,294.1)	241.5 (189.6 ,304.6)	4828.4 (3837.3 ,6116.7)	5089.4 (3935.9 ,6448.5)	824.4 (606.2 ,1096.9)	824.5 (606 ,1097.1)	5459.5 (3823.6 ,7784.2)	6344.7 (4331 ,9320.8)	437.9 (356.3 ,527.6)	484.3 (395.2 ,583.9)	157.2 (86.2 ,253.3)	156 (85.6 ,251.5)	217.4 (50.4 ,388.9)	152 (26.3 ,289.6)	2721.3 (2174.1 ,3389.7)	2714 (2168.4 ,3380)	2155.8 (1561.7 ,2878.3)	1422.7 (1037.3 ,1888.5)	114.2 (90.4 ,143.1)	118.7 (94.9 ,147.4)
**Australasia**																									
Male	12614.8 (11205.2 ,14283.9)	12724.6 (11150.2 ,14693)	0.03 (0.02 ,0.04)	2472.8 (1902 ,3259.8)	2640.6 (2021.9 ,3354.4)	0.22 (0.19 ,0.24)	332 (287 ,385.2)	335.1 (290.4 ,387.2)	3859 (2990.7 ,4884.1)	3840.8 (2861.4 ,5078.1)	950.7 (736.6 ,1213.1)	948.2 (730.2 ,1221.6)	3272.3 (2239 ,4698.1)	3299.8 (2086.4 ,4988)	1300.7 (1057 ,1562.9)	1406.9 (1150.3 ,1706.4)	233.7 (128.7 ,380.4)	234.5 (125.6 ,378.6)	21.4 (0 ,125)	29 (0 ,139.5)	3321.1 (2787.3 ,3985.7)	3321.5 (2788.2 ,3985.2)	2202.5 (1636.1 ,2990.1)	2370.4 (1748.8 ,3102.8)	276.7 (219.5 ,342.6)	277.1 (222.1 ,334.3)
Female	14358.7 (12466.3 ,16783.7)	14375.3 (12187.9 ,17228.8)	0.02 (-0.0036 ,0.04)	1072.4 (769.2 ,1451.1)	1208.9 (900.9 ,1635.1)	0.38 (0.38 ,0.39)	362 (297.3 ,425.8)	367.1 (301.8 ,430.6)	4658.4 (3766.8 ,5773.7)	4379.9 (3297.4 ,5799.1)	1068.4 (845.5 ,1339.3)	1075.7 (848.9 ,1349.3)	5614.1 (3941.2 ,7974.8)	5779.1 (3775.1 ,8435.5)	528.6 (430.2 ,643.6)	556.7 (451.1 ,679.5)	102.6 (50.2 ,171.9)	103 (49.4 ,173.2)	106.3 (16.5 ,221.4)	199.6 (33.6 ,364.1)	2747.1 (2278.7 ,3316.4)	2747.8 (2280.1 ,3317.9)	876.5 (579 ,1260.3)	998.4 (693.5 ,1420.6)	197.6 (159.5 ,241)	212.6 (174.8 ,256.1)
Both	13567.7 (12008.1 ,15636.8)	13590.3 (11755.4 ,15965.2)	0.02 (0.0007 ,0.03)	1713.4 (1330.7 ,2198.4)	1894.1 (1471.4 ,2395.8)	0.33 (0.31 ,0.34)	348.8 (295.9 ,403.8)	351.9 (301.3 ,405.5)	4290.2 (3465.7 ,5315.3)	4125.5 (3122.9 ,5379.1)	1014.8 (798.3 ,1285.5)	1014.9 (791.9 ,1281.1)	4557.4 (3270.5 ,6406.7)	4596.8 (3046.6 ,6727.6)	877.7 (717 ,1052)	961.7 (789.1 ,1157)	166.4 (91.6 ,270.9)	166.7 (89.7 ,268.7)	68.1 (9.8 ,172.4)	118.3 (21.2 ,250.1)	3003.2 (2527 ,3601.5)	3020.3 (2540.8 ,3621.3)	1482.9 (1098.9 ,1969.9)	1655.1 (1236 ,2159.1)	234.5 (190.2 ,285.5)	243.4 (198.8 ,290.6)
**Caribbean**																									
Male	13279.3 (11602 ,15320.7)	13763.2 (11970.9 ,15982.8)	0.12 (0.09 ,0.14)	3464.4 (2563.4 ,4432.6)	3506.5 (2815.1 ,4306.1)	0.23 (-0.01 ,0.48)	250.5 (197.4 ,315.1)	256.9 (202.4 ,322.1)	5229.4 (4087.5 ,6646.7)	5402.6 (4184.1 ,6942.5)	810.3 (595.3 ,1081)	809.6 (595.1 ,1078.5)	3440.6 (2317.6 ,5005.9)	3770.7 (2481.4 ,5485.3)	649.9 (528.4 ,777.9)	682.7 (562.8 ,816.9)	337.6 (192.7 ,526.9)	337.5 (191.7 ,528.3)	118.7 (18.3 ,293.2)	117.9 (15.8 ,271.6)	3153.2 (2516.2 ,3930.7)	3153.2 (2516.2 ,3930.7)	3282.3 (2384.9 ,4249.9)	3325.4 (2639.7 ,4113.3)	188.9 (141.6 ,246.7)	187.8 (142.2 ,244.4)
Female	16205.4 (13886.1 ,19083)	16758.6 (14313.8 ,19899.2)	0.13 (0.10 ,0.15)	909.8 (646 ,1239.2)	892.4 (654.1 ,1198.3)	-0.06 (-0.07 ,-0.05)	210 (164.3 ,265.4)	214.2 (167.6 ,269.7)	7623 (5967.2 ,9801.6)	7620.2 (5899.2 ,9875.1)	836.3 (615.7 ,1114)	835.6 (614.7 ,1114)	5605.7 (3872.2 ,8039)	6220.2 (4303.6 ,9131.4)	318.7 (257.2 ,387.5)	327.7 (264.5 ,396)	127.3 (69.1 ,204.9)	127.3 (67.4 ,208.5)	147.2 (24.5 ,301.1)	148.3 (22.7 ,286.7)	2318.2 (1855.3 ,2904.7)	2318.2 (1855.3 ,2904.7)	798.4 (534.3 ,1128.5)	785 (549.8 ,1090.6)	112.2 (86.5 ,143.7)	108.2 (84 ,137.6)
Both	14796.3 (12862 ,17184.8)	15364.2 (13305.1 ,17978.5)	0.14 (0.12 ,0.16)	2143.9 (1607.4 ,2742)	2118.5 (1693.8 ,2622.6)	0.10 (-0.07 ,0.28)	229.7 (180.9 ,288.8)	234.4 (184 ,294)	6466.3 (5089.3 ,8252.4)	6584.1 (5129.6 ,8436.1)	823.7 (605.3 ,1096.5)	823.4 (605 ,1097.2)	4566.2 (3239.2 ,6494.4)	5081 (3548.8 ,7385.8)	478.6 (388.9 ,575.2)	493.8 (405.1 ,590.9)	230.2 (132.8 ,357.7)	227.5 (129.2 ,354.5)	133.5 (21.8 ,296.5)	134.1 (19.5 ,280)	2719 (2172.4 ,3386.6)	2706.2 (2162.1 ,3370)	1998.1 (1470.2 ,2593.3)	1976.3 (1554.9 ,2481)	149.5 (115 ,193.2)	145.8 (113.3 ,187.4)
**Central Asia**																									

	10971.8	11215.9	0.08	5905.1	5288.2	-0.34	199.8	206.2	4559.7	4656.3	535.8	535.7	1877.6	2023.6	823	873.7	87.5	87.5	204	171.4	3153.2	3153.2	5763.7	5127.9	150.8	170.6
Male	(9658.8	(9857.7	(0.07	(4288.3	(4022.8	(-0.40	(157.8	(163.3	(3585	(3648.9	(386.9	(386	(1289.4	(1366.1	(673.2	(712.3	(42.7	(42.7	(34.5	(26	(2516.2	(2516.2	(4129.3	(3863.9	(119.8	(141.8
	,12481.4)	,12787.7)	,0.08)	,7556.2)	,6740.3)	,-0.28)	,250.6)	,257.7)	,5742.7)	,5933.5)	,719.8)	,719.7)	,2684.5)	,2874.2)	,995)	,1048.8)	,145.1)	,145.1)	,435.6)	,371.9)	,3930.7)	,3930.7)	,7416.8)	,6580.3)	,187.2)	,202.6)
	15004.4	15134.9	0.03	2515	1451.5	-1.66	195.5	201	8844.7	8711.7	560.6	560.3	3146.4	3449.9	417.9	431.7	41.1	41.1	233.9	202.4	2318.2	2318.2	2357.6	1281.2	161.3	172.7
Female	(12956.7	(13012.7	(0.02	(1870.5	(1134.9	(-1.80	(155.4	(160.3	(7036.3	(6842	(407.6	(406.2	(2146.8	(2337.2	(339.4	(353	(18.4	(18.4	(53.6	(39	(1855.3	(1855.3	(1706.8	(964.2	(123	(138.9
	,17421.3)	,17794.6)	,0.05)	,3311.7)	,1816.1)	,-1.52)	,245)	,252.5)	,11034.2)	,11078.1)	,752.3)	,751.7)	,4599.5)	,5073.8)	,504)	,517.7)	,70.2)	,70.2)	,432.8)	,365.7)	,2904.7)	,2904.7)	,3154.4)	,1641.5)	,210.6)	,214.6)
	13546.4	13515.8	0.00	3790.5	3017.3	-0.69	197.3	203.4	7283.3	7027.5	551	549.7	2686.8	2862.1	570	618	60.3	61.9	222.8	189.5	2625.5	2665	3638.2	2849.8	158.4	173.3
Both	(11805.9	(11744.4	(-0.01	(2805.1	(2328.6	(-0.75	(156.9	(162.2	(5801.9	(5555	(399	(397	(1859.8	(1958.1	(465.5	(506	(29.4	(30	(46.3	(35.2	(2097.7	(2128.9	(2652.9	(2160.2	(123.7	(142.8
	,15603.5)	,15692.5)	,0.01)	,4822.3)	,3793.2)	,-0.63)	,247.3)	,253.3)	,9074)	,8899.7)	,737.9)	,736.2)	,3863.1)	,4115.6)	,679.8)	,739.5)	,100)	,102.7)	,433.8)	,368.7)	,3268.1)	,3317.7)	,4669.3)	,3615)	,200.5)	,208)
**Central Europe**																										
	10857.9	11202.6	0.11	4533.3	4756.4	0.17	211.4	223.3	3360.6	3427.2	540	540.7	2863.8	3139.3	905.4	1014.5	84	83.3	194.3	105.7	3177.5	3179.8	4411.9	4627.3	127.6	136.1
Male	(9667.2	(9924.4	(0.10	(3598	(3811.7	(0.14	(170.9	(181.7	(2704	(2751.1	(407	(409	(2027.9	(2208.5	(743.9	(830.1	(40.8	(40.3	(29.4	(12.4	(2541.3	(2544.5	(3478.2	(3677.7	(99.8	(107.9
	,12341.5)	,12739.2)	,0.12)	,5551.6)	,5807.9)	,0.19)	,260.2)	,273.6)	,4176.1)	,4252.6)	,706.6)	,705.3)	,4035.9)	,4388)	,1082.2)	,1215.7)	,139.8)	,138.7)	,402.9)	,264.2)	,3956.2)	,3967.7)	,5432.7)	,5671.6)	,161)	,168.8)
	15365.3	15793.6	0.10	3228.5	2466.3	-0.73	207.7	217.6	7006	6823.3	581.9	582.7	5362.3	6064.3	460.3	490.5	38.8	38.4	236.9	158.2	2352	2353.5	3127	2358.8	104.9	110.2
Female	(13399.4	(13732.6	(0.09	(2365.1	(1832.3	(-0.94	(168.8	(176.3	(5675.7	(5457.4	(440.1	(441.7	(3892.9	(4388.5	(376.3	(400.3	(17.4	(17.1	(50.8	(22.8	(1892.4	(1893.7	(2260.3	(1728.4	(81.3	(87.2
	,17691.9)	,18324.3)	,0.12)	,4278.5)	,3237.9)	,-0.52)	,254.5)	,265.1)	,8632.3)	,8439.1)	,757.2)	,756.1)	,7414)	,8387.6)	,551.9)	,589.1)	,66.9)	,66.5)	,419.5)	,298.1)	,2938.6)	,2938.2)	,4172)	,3130.6)	,131.2)	,135.6)
	13517.7	13820.6	0.08	3801.1	3480.6	-0.22	209.4	220.2	5503.2	5357.3	564	564	4340.9	4812.1	646.1	716.8	58.9	59.2	219.5	135.8	2691.1	2706.9	3690.4	3362.7	115.4	122.6
Both	(11926.9	(12123.8	(0.07	(2946.5	(2742.2	(-0.32	(169.7	(179.5	(4462.5	(4310.7	(426.3	(427.1	(3155.7	(3473	(530	(586.6	(28.6	(28.7	(42.9	(19.8	(2164.2	(2177.9	(2843.1	(2620.2	(89.9	(97.5
	,15441.3)	,15903.2)	,0.10)	,4810.2)	,4345.8)	,-0.12)	,256.5)	,268.7)	,6775.5)	,6621)	,734.5)	,732.1)	,6025.5)	,6633.3)	,767.6)	,855.8)	,98.5)	,99.3)	,412.9)	,283.1)	,3347.5)	,3369)	,4699.1)	,4227.7)	,144.2)	,151)
**Central Latin America**																										
	12538.5	13099.6	0.14	4616.5	3802	-1.11	258.1	265.2	4917.5	5200.2	770.5	770.1	3102.4	3396.4	682.7	750.2	117.7	119.1	116.6	85.5	3196.7	3195.4	4446.6	3626.6	178	182.2
Male	(11211.5	(11607.3	(0.10	(3572.1	(2922.3	(-1.38	(210.3	(217.2	(4008.9	(4167.1	(598	(597.3	(2258.5	(2413.6	(557.2	(615.2	(61.1	(61	(15.3	(11.1	(2557.5	(2556.6	(3398.1	(2749.5	(140.7	(144.8
	,14126.2)	,14841.6)	,0.16)	,5883)	,4849.3)	,-0.83)	,313)	,323.6)	,6055.6)	,6394.3)	,980.7)	,980.7)	,4245.7)	,4697.3)	,810.7)	,896.7)	,191)	,193.6)	,266.5)	,214.2)	,3991.8)	,3990.5)	,5722.1)	,4682.8)	,224.8)	,228.3)
	14169.6	14889.2	0.17	868.6	711	-0.62	222.5	226.8	6154.6	6390.2	783.4	780.5	4847.8	5414.1	333.1	353.2	55.6	56.4	159.1	131.9	2370.5	2369.6	737.7	594.7	131.8	116.9
Female	(12379.2	(12922.4	(0.13	(647.3	(532	(-0.65	(181.4	(184.6	(4875.6	(5072.6	(610.2	(607.9	(3589.4	(3933.8	(270.9	(287	(27.2	(27.5	(27.1	(17.3	(1906.2	(1905.2	(518.3	(417	(106.4	(94.2
	,16275.7)	,17240.4)	,0.21)	,1143.1)	,934.7)	,-0.59)	,269.8)	,275.2)	,7683.2)	,7984.1)	,992.8)	,989.9)	,6589.5)	,7481)	,401.7)	,428.1)	,93.6)	,95)	,293.6)	,250.8)	,2953.7)	,2953.1)	,1006.7)	,817.5)	,161.2)	,143)
	13386.6	14071.4	0.17	2671.3	2124	-1.14	239.7	244.5	5558.5	5843.3	777.2	775.8	4012.2	4495.7	501	534.9	85.7	85.5	138.7	110.7	2766.6	2745.6	2521.5	1980.4	154.2	147.1
Both	(11851	(12371.1	(0.13	(2063	(1639.2	(-1.36	(195.7	(199.4	(4491.5	(4687.4	(604.3	(603	(2986.4	(3276.6	(408.4	(437.5	(43.5	(44	(21.9	(15.4	(2226.4	(2210.1	(1910.6	(1494.4	(123.6	(118.4
	,15199.6)	,16120.1)	,0.20)	,3405.1)	,2707.3)	,-0.94)	,290.4)	,298.5)	,6816.2)	,7204)	,985.5)	,984.3)	,5422.2)	,6174.1)	,596.6)	,640.3)	,139.7)	,139.4)	,281.9)	,232.8)	,3444.3)	,3417.5)	,3259.4)	,2566)	,191.8)	,181.6)
**Central Sub-Saharan Africa**																										
	16036.9	16341.8	0.07	2567.2	2691.6	0.14	183	181.4	9425.7	9546	598.6	598.6	2710.8	2814.1	733	785	30.3	30.3	73.1	147.7	3153.2	3153.2	2449.8	2562.4	120.5	132.8
Male	(13751.5	(13837.2	(0.05	(1802.6	(1965.5	(0.07	(141.9	(140	(7229.9	(7133.3	(435.5	(435.4	(1835.9	(1812.4	(592	(633.2	(12	(12	(21	(27.3	(2516.2	(2516.2	(1679.5	(1833.4	(94.1	(106.7
	,18810.5)	,19481.3)	,0.08)	,3487.8)	,3526.1)	,0.18)	,233.3)	,230.2)	,12219.8)	,12639.3)	,804.1)	,803.9)	,3911.9)	,4125.6)	,884)	,942.1)	,56)	,56)	,242.2)	,374.1)	,3930.7)	,3930.7)	,3374.7)	,3398.4)	,152.4)	,164)
	17405.5	17971.1	0.13	767.6	695	-0.33	194	191.8	10440.7	10710.8	604.7	604.8	4396.3	4672.8	362.1	384.9	15.7	15.7	72.5	148.5	2318.2	2318.2	699.9	623	68.2	72.4
Female	(14755.5	(14965.8	(0.10	(536.2	(498.7	(-0.34	(150.5	(148.2	(7992.5	(8038.1	(434.8	(435.3	(3009.2	(3045.6	(289.5	(308.3	(6.1	(6.1	(23.8	(28.6	(1855.3	(1855.3	(466.8	(424.5	(50.1	(54.3
	,20522.3)	,21753.7)	,0.17)	,1043.5)	,943.2)	,-0.31)	,247.8)	,242.4)	,13335.8)	,14121.5)	,817.7)	,818.2)	,6361.4)	,6998.4)	,442.8)	,468)	,30.1)	,30.1)	,213.2)	,334)	,2904.7)	,2904.7)	,976.5)	,869.1)	,89.4)	,94.5)

	16765.6	17274.4	0.11	1601.5	1595.3	-0.03	188.9	187.2	9967.9	10203.6	601.9	602	3605.8	3884.8	535.6	559.5	22.5	22.5	72.7	148.2	2709.1	2674.5	1510.5	1499.2	92.7	98
Both	(14353	(14589.9	(0.09	(1148.7	(1191.4	(-0.08	(146.9	(145.6	(7685.5	(7670.3	(436.9	(437.2	(2517.8	(2566	(430.8	(450.9	(9.1	(9.1	(23.8	(28.8	(2164.8	(2136.2	(1058.7	(1097	(70.9	(77
	,19708.5)	,20788.7)	,0.14)	,2151.8)	,2073.2)	,0.01)	,239.5)	,236.7)	,12753.7)	,13452.7)	,807.9)	,807.4)	,5102.6)	,5712)	,644.7)	,669.8)	,41.4)	,41.4)	,226.6)	,351.9)	,3372.3)	,3330.3)	,2067.8)	,1973.2)	,117.6)	,122.3)
**East Asia**																										
	12162.2	12822.1	0.15	1628.9	1808.9	0.29	298.5	308.6	4553.2	5164.7	226.1	225.8	3436.6	3483.5	666.7	798.6	188.2	166.8	142	83.3	3233.9	3234.2	1419.1	1685.6	213	125.6
Male	(10890.2	(11513.1	(0.13	(1227.4	(1341.8	(0.21	(257	(266.1	(3773.8	(4299.1	(172.9	(172.7	(2601.4	(2627.2	(541.9	(653.1	(104.2	(90.2	(26.7	(12.7	(2573.1	(2572.9	(1018	(1216.2	(169.2	(98.5
	,13624.5)	,14252.1)	,0.18)	,2135.4)	,2392.3)	,0.36)	,345.4)	,357.5)	,5477.6)	,6212.1)	,288.5)	,288.2)	,4511.3)	,4590.9)	,798.9)	,961.6)	,298.3)	,272.1)	,315.2)	,207.5)	,4070.6)	,4071.5)	,1924.5)	,2264.8)	,263.8)	,156.3)
	14971.6	15889	0.18	687.4	556.6	-0.76	280.5	290.9	6845.3	7761.9	223.9	223.7	5519.2	5631.1	222.5	250.1	86.1	74.5	193	142.6	2419.5	2419.7	400.3	401.7	288.3	155.5
Female	(13303.9	(14134.2	(0.15	(541.2	(417.2	(-0.84	(242.6	(251.5	(5684.8	(6478.8	(172.8	(172.5	(4238	(4330.7	(177.9	(201.5	(44.1	(37.4	(62.8	(34.3	(1926.3	(1926.4	(272.8	(270.6	(227.5	(121.2
	,16982.1)	,17982.1)	,0.20)	,867.3)	,727.9)	,-0.69)	,323.4)	,336.7)	,8204.2)	,9225.8)	,285.1)	,284.5)	,7261)	,7377.3)	,268.6)	,305.7)	,140.8)	,124.8)	,342.1)	,249)	,3002.7)	,3003)	,564.4)	,564.3)	,362.4)	,194.7)
	13679.8	14440.4	0.16	1141.6	1157.4	0.01	289.7	299.8	5778.8	6527.9	224	224.2	4562.7	4618.7	432.9	512.3	137.4	120.3	169	114.3	2795.6	2804.9	889.5	1017.2	254.3	141.6
Both	(12242.4	(12931.2	(0.13	(880.7	(866.9	(-0.06	(250.3	(258.9	(4809.9	(5466.4	(172.4	(172.6	(3522.1	(3547.5	(350.5	(417.9	(75.2	(64.5	(45.8	(24.6	(2236.8	(2244.3	(632.5	(729	(202.6	(111.6
	,15399.4)	,16149.1)	,0.17)	,1471.9)	,1522.8)	,0.08)	,334.1)	,346.7)	,6934.5)	,7794.5)	,285)	,285.2)	,5947.4)	,6044.6)	,519.3)	,618.1)	,219.4)	,196.6)	,332.1)	,228.2)	,3495.4)	,3506.9)	,1213)	,1382.6)	,316.4)	,176.1)
**Eastern Europe**																										
	12956.5	13217	0.06	8738.3	8719	-0.06	187.9	198.3	5901.2	5776.8	554.6	554.7	2657.8	2998.4	881.9	963.5	76.4	76.3	112.5	99.9	3226.4	3228.6	8487.4	8504.9	278.2	236.7
Male	(11521.6	(11726.3	(0.06	(6807.3	(6754.6	(-0.11	(156.3	(165.6	(4764.8	(4648.1	(431.6	(431.8	(1992.7	(2246.2	(723.3	(789	(36.1	(36.1	(16.5	(12.6	(2571.3	(2572.1	(6554.5	(6525.6	(225.1	(195.4
	,14506.2)	,14771.7)	,0.07)	,11032.5)	,11029.9)	,-0.01)	,224.9)	,236)	,7272.8)	,7105.8)	,700.2)	,699.1)	,3512.1)	,3933.8)	,1059.3)	,1159.4)	,130.7)	,130.6)	,289.7)	,256.3)	,4051.3)	,4056.1)	,10787.9)	,10823.4)	,340.6)	,281.9)
	15607.7	15939.2	0.07	2767.2	2074.8	-0.85	190.8	198.3	7728.6	7358.7	579.9	579.8	4937.7	5672.3	443.1	463.5	34.1	34.1	166.3	158.5	2413.7	2413.2	2537.7	1874.9	236.1	204.2
Female	(13766.7	(14023.9	(0.06	(2143	(1619	(-0.99	(159.8	(166.3	(6233.3	(5902.6	(452	(452	(3822.3	(4367.3	(360.7	(377.5	(14.7	(14.7	(25.1	(20.8	(1924.2	(1924.3	(1909.3	(1418.8	(185.5	(162.7
	,17729)	,18165.3)	,0.08)	,3552.6)	,2633.4)	,-0.73)	,225.6)	,234.7)	,9470.8)	,8990.6)	,731.3)	,730.8)	,6431.2)	,7397.8)	,532.8)	,558.9)	,60.2)	,60.2)	,326.1)	,304.3)	,2995.4)	,2994.7)	,3316.6)	,2439.4)	,295.3)	,251.2)
	14776.6	14937.4	0.03	4748.6	4581.3	-0.11	189.9	198.4	7141.7	6767	571.3	570.1	4230.3	4697.3	585.1	649	49.9	51.5	149.1	136.9	2669.5	2711	4509.8	4374.2	253	218.8
Both	(13078.8	(13195.3	(0.03	(3713.8	(3570.6	(-0.13	(158.8	(166.3	(5770.4	(5433	(445.4	(444.6	(3279.4	(3611.3	(480.6	(531.5	(23.6	(24.4	(23.7	(19.9	(2139.3	(2173.1	(3470.7	(3357.1	(201.1	(178.1
	,16668.1)	,16918.5)	,0.04)	,5982.9)	,5797.8)	,-0.08)	,225.3)	,235.1)	,8762.7)	,8289.1)	,719.8)	,717.8)	,5485)	,6098.5)	,696.2)	,776.6)	,85.3)	,88.3)	,314.5)	,287.6)	,3323.8)	,3379.1)	,5739.8)	,5590.1)	,313.8)	,264.3)
**Eastern Sub-Saharan Africa**																										
	15880.8	16241.3	0.08	3917.1	3815.4	0.0039	176.4	182.8	9247.4	9408.4	649.9	650.3	2601.7	2765.9	739.6	799.2	27.9	27.9	116.5	125.5	3183.6	3191.4	3795.2	3676.8	127.1	144
Male	(13935.2	(14219.8	(0.07	(2956.7	(2966.3	(-0.09	(140.9	(146.5	(7425.4	(7573.7	(491.8	(493	(1843.3	(1980.6	(598.3	(647.4	(11.2	(11.2	(39.4	(28.6	(2548.6	(2554.2	(2834.8	(2823.2	(100	(117.5
	,18238.6)	,18589.6)	,0.09)	,5006.9)	,4763.4)	,0.10)	,219.9)	,226.6)	,11425.8)	,11647.6)	,845.3)	,843.2)	,3590.1)	,3818.2)	,890.2)	,952.8)	,52.4)	,52.3)	,302.3)	,331.5)	,3973.4)	,3982.6)	,4888.6)	,4622)	,159.5)	,175.4)
	19361.3	19658.3	0.06	1384.7	1268.6	-0.32	188.1	194.1	12336.9	12257.2	664.1	664.5	4504.7	4893.3	365	389.8	14.2	14.2	121.3	139.2	2354.4	2359.8	1309.4	1195.8	76	73.5
Female	(16768.6	(17003.5	(0.04	(1026.9	(977	(-0.41	(151.2	(156.9	(9872.2	(9802.1	(498.1	(499.8	(3267.1	(3540.2	(293	(315.4	(5.3	(5.3	(39.1	(29.4	(1895	(1898.3	(956.2	(905.8	(56.1	(54.5
	,22405.4)	,22820.9)	,0.07)	,1779.4)	,1611)	,-0.26)	,233.6)	,239.4)	,15272.2)	,15217.2)	,863.7)	,862.2)	,6183.4)	,6720.3)	,441)	,472.9)	,27.4)	,27.3)	,284.6)	,315.7)	,2939.3)	,2943.5)	,1705.8)	,1536.2)	,99.3)	,95.9)
	17626.4	18064.4	0.09	2696.9	2496.6	-0.22	182.2	188.8	10794.5	10924.5	657	657.8	3558	3905.2	553.3	582.2	21.1	20.8	118.8	132.7	2766.8	2745.9	2598.5	2393.4	101.4	106.1
Both	(15356.6	(15709.7	(0.08	(2048.7	(1957.2	(-0.33	(146.4	(151.7	(8652.4	(8779.2	(494.9	(496.7	(2602.6	(2882.1	(447.2	(472.4	(8.3	(8.2	(39.5	(29.2	(2226	(2209.7	(1952.9	(1855	(78.1	(84.2
	,20330.7)	,20790.3)	,0.10)	,3407.8)	,3101.9)	,-0.05)	,226.8)	,233.2)	,13318.5)	,13546.5)	,853.3)	,852.8)	,4835.3)	,5349.4)	,665.6)	,693.9)	,39.9)	,39.1)	,293.1)	,324.8)	,3443.9)	,3417.8)	,3314.5)	,2997.3)	,128.3)	,131.8)
**High-income Asia Pacific**																										
	9654.2	9965.6	0.11	1475.7	1865.7	0.80	242.9	234.6	2305.3	2436.9	574.8	565.4	1943.7	2037.2	1594.2	1778.5	68.6	70.5	75.5	16	3234	3237.1	1300.6	1697.7	177.7	171.5
Male	(8730.6	(9006.1	(0.10	(1112.8	(1413.6	(0.74	(204.1	(194.9	(1903.7	(1987.7	(446.9	(435.3	(1470.2	(1510.3	(1319	(1471.5	(31.5	(33.1	(4.9	(0	(2612.6	(2614.8	(942.2	(1249.1	(140.6	(136.5
	,10672.9)	,11017.7)	,0.11)	,1890.6)	,2388.3)	,0.86)	,289.6)	,282.2)	,2763.5)	,2942)	,728.2)	,726.3)	,2568.7)	,2708.2)	,1892.3)	,2117.5)	,121.8)	,122.6)	,249.8)	,98.1)	,3995.4)	,3996.9)	,1713.8)	,2227.2)	,221.5)	,213.7)

Female	10001.7 (8952.6 ,11169.5)	10549.2 (9340.4 ,11944.4)	0.17 (0.17 ,0.18)	651.7 (494.6 ,843.7)	615.3 (454.9 ,819.7)	-0.18 (-0.21 ,-0.17)	277.2 (232.4 ,327.5)	283.7 (236.3 ,336.7)	3219.1 (2650.6 ,3890.4)	3454.7 (2813.5 ,4212.2)	655.2 (513.1 ,828.5)	651.4 (507.9 ,825.8)	3155.2 (2375.5 ,4173.1)	3528.1 (2610.8 ,4746.7)	636.9 (521.5 ,761.6)	717.2 (589.1 ,854.3)	23.5 (8.8 ,44.3)	24.6 (9.5 ,46.4)	108.7 (18.5 ,232.6)	10.4 (0.1 ,56.3)	2325.3 (1872.3 ,2907.7)	2320.8 (1876.7 ,2898.6)	508.2 (355.5 ,697.8)	478.4 (319.3 ,683.7)	144.3 (112 ,182.9)	137.6 (107.3 ,173.5)
Both	9872.3 (8894 ,10943.9)	10287.4 (9248.2 ,11477.1)	0.14 (0.13 ,0.14)	1010.1 (782.6 ,1276.8)	1213.1 (940.2 ,1530.1)	0.62 (0.58 ,0.67)	263.1 (220.9 ,311.1)	260.8 (217.2 ,310.8)	2845.2 (2355.7 ,3410.5)	2989.9 (2445.2 ,3620.1)	620.7 (485.4 ,786.2)	610.8 (473.7 ,778.3)	2646.9 (2014.3 ,3465.9)	2832.2 (2116.3 ,3786.5)	1044.5 (862.9 ,1240.8)	1214.4 (1003.1 ,1445.3)	44.2 (19.7 ,78.6)	47.1 (21.6 ,83.1)	95.1 (14.8 ,237.6)	13.2 (0.1 ,76.4)	2707.3 (2183.7 ,3336.8)	2747.2 (2213.3 ,3383.3)	852.9 (622.2 ,1119.8)	1061.5 (787.5 ,1376.5)	158.9 (125.2 ,199)	153.6 (122.1 ,193)
**High-income North America**																										
Male	11769.5 (10622.9 ,13125)	12273.5 (11039.5 ,13706)	0.07 (0.05 ,0.09)	2699.6 (2130.4 ,3404.6)	2789.9 (2263.6 ,3438.6)	0.13 (0.08 ,0.17)	329.6 (278 ,385.5)	317.4 (269.5 ,371.2)	2785.7 (2307.5 ,3366.7)	3018 (2477.2 ,3658.9)	598.7 (525.2 ,681.1)	599.9 (528.1 ,678.2)	3561 (2685.4 ,4671.6)	3868.7 (2900.5 ,5097.6)	1107.3 (912.8 ,1321.6)	1173.7 (960.2 ,1403.6)	98.6 (48.5 ,167)	101.8 (49.2 ,174.8)	203.3 (34.6 ,427.8)	164.6 (25.7 ,366.9)	3659.6 (2942.7 ,4577.3)	3658.2 (2942.7 ,4574.3)	2371.7 (1798.2 ,3086.4)	2228.2 (1710.8 ,2857.4)	336.9 (270.4 ,412.5)	576.2 (490.3 ,676.4)
Female	14337.9 (12698.9 ,16333.6)	15453.3 (13689.3 ,17664.1)	0.17 (0.15 ,0.20)	1327.8 (1050.2 ,1667.1)	1773.9 (1452.2 ,2134.6)	0.98 (0.91 ,1.05)	340.4 (287.8 ,398.8)	346.2 (293.8 ,404.8)	4324.8 (3587.8 ,5152.4)	4841.4 (4007 ,5812.8)	622 (548.6 ,705)	630.9 (559.2 ,713.1)	6799.7 (5271.6 ,8729.1)	7521.4 (5858 ,9716.6)	500.5 (409.8 ,601.3)	518.6 (423.1 ,622.6)	50.7 (22.6 ,89.8)	51.7 (23.5 ,89.8)	224.1 (48.1 ,399)	194.2 (35.9 ,353.8)	2254.7 (1819 ,2818.3)	2253.7 (1818.2 ,2817)	1037.2 (763.1 ,1368.4)	1127.6 (838.1 ,1475.2)	293.8 (239.3 ,355.8)	654.4 (553.8 ,778.1)
Both	13228.9 (11828.8 ,14881.9)	13974.3 (12514.8 ,15795.9)	0.13 (0.12 ,0.16)	1939.1 (1534.1 ,2435.9)	2247.1 (1837.7 ,2739.9)	0.50 (0.45 ,0.55)	336.2 (283.7 ,392.2)	333 (282.7 ,388.4)	3655.7 (3037.6 ,4376.2)	3991.8 (3303.3 ,4794.7)	611.7 (538 ,693.5)	616.4 (545.1 ,697.3)	5410.1 (4216.9 ,6929.5)	5828.5 (4540.3 ,7552.5)	762.8 (628.9 ,912.2)	822.3 (673.6 ,981.9)	72.8 (35.3 ,122.7)	75.7 (36.2 ,129.5)	215.8 (44.8 ,413.1)	180.9 (32.8 ,358.5)	2853.9 (2304 ,3554)	2901.7 (2343.1 ,3613.8)	1631.8 (1230.8 ,2128)	1640.8 (1246.2 ,2118.2)	313.1 (254.7 ,380.9)	617.5 (527.9 ,727.1)
**North Africa and Middle East**																										
Male	13024.3 (11511.6 ,14785.4)	13265.8 (11687 ,15148.1)	0.16 (0.14 ,0.19)	659.7 (483.2 ,877.5)	634.1 (473.6 ,833.5)	-0.14 (-0.15 ,-0.12)	217.1 (176.5 ,266.1)	226.9 (184.3 ,277.5)	4716.8 (3728.5 ,5948.7)	4888.1 (3826.1 ,6254)	717.6 (537.4 ,939.1)	718 (537.6 ,938.8)	3225.3 (2304.7 ,4487.5)	3485.4 (2441.2 ,4907.7)	679.1 (555.6 ,809.7)	764.3 (627.1 ,914.5)	177.8 (103.5 ,277)	172.9 (98.6 ,273.3)	837 (370.5 ,1282.9)	572.7 (200.1 ,922.7)	3165 (2534.4 ,3939)	3169.5 (2537.2 ,3943)	549.7 (376.5 ,762)	509.8 (349.5 ,710.1)	110.7 (86.4 ,138.8)	125 (101.4 ,151.6)
Female	16172.5 (14049.6 ,18777)	16975.5 (14563.5 ,20027.2)	0.24 (0.19 ,0.28)	320.9 (235.2 ,428.3)	314.4 (233.7 ,410.4)	-0.06 (-0.08 ,-0.05)	206.5 (167.7 ,253.5)	213.8 (174.5 ,261.9)	6996.7 (5536.3 ,8784.1)	7391.2 (5759.6 ,9445.6)	776.3 (577.7 ,1019.7)	779.5 (582 ,1020.5)	5683.1 (4105.2 ,7968.3)	6356.8 (4510.1 ,8960.7)	341 (276.4 ,414.2)	371.5 (302.9 ,446.5)	97.6 (55.3 ,152.3)	99.5 (55.4 ,156.7)	761.3 (379.7 ,1127.8)	542.6 (245.2 ,841.7)	2332.8 (1871.6 ,2918.9)	2337.6 (1877.2 ,2924.6)	210.8 (132.1 ,313.6)	200.7 (128.4 ,292.8)	110.3 (83.5 ,145.5)	113.8 (88.3 ,144.9)
Both	14586.9 (12828 ,16752)	15114.6 (13199.6 ,17526)	0.20 (0.17 ,0.23)	491.7 (363 ,650.3)	474.6 (357.2 ,619.5)	-0.12 (-0.13 ,-0.11)	211.9 (172.5 ,260)	220.4 (180 ,269.9)	5844.6 (4631.2 ,7322.5)	6131.8 (4771.6 ,7834.2)	746.5 (557.2 ,980)	748.6 (559.7 ,980.4)	4447.6 (3250.7 ,6134.5)	4920.4 (3511.8 ,6881.8)	512.5 (417.7 ,613)	568.4 (465.2 ,679.2)	138.8 (80.2 ,214.7)	136.9 (78.6 ,213.6)	799.4 (374.8 ,1206.6)	557.8 (222.7 ,883.1)	2751.2 (2204.6 ,3421.6)	2753.8 (2207 ,3422.6)	381.4 (256.2 ,534.5)	355.5 (240.3 ,498.2)	110.7 (85.3 ,142.5)	119.5 (95.3 ,148)
**Oceania**																										
Male	11723.8 (10176.7 ,13659.8)	11971.9 (10217.9 ,14017)	0.06 (0.05 ,0.07)	1268 (923 ,1720.6)	1314.2 (979.4 ,1706.6)	0.06 (0.02 ,0.10)	276.3 (217.6 ,345.1)	274.3 (217.4 ,342.6)	4125.7 (3221.6 ,5275.6)	4160.8 (3175 ,5379.6)	358.3 (255 ,487.9)	358.3 (254.8 ,488)	3453.1 (2351.7 ,4928.4)	3665.5 (2300.3 ,5511.5)	636.5 (515.9 ,767.5)	652.9 (530.4 ,788.7)	93.5 (46.3 ,155.9)	93.5 (46.3 ,155.9)	172.4 (36.6 ,393)	182.9 (32.6 ,382.2)	3153.2 (2516.2 ,3930.7)	3153.2 (2516.2 ,3930.7)	1136.1 (799.4 ,1592.2)	1180.5 (849.8 ,1575.4)	133.3 (97.3 ,177.1)	135.2 (100.7 ,177.9)
Female	14121.4 (11949.5 ,16896.8)	14475.8 (12029.5 ,17589.7)	0.07 (0.05 ,0.08)	466.3 (335.2 ,632.8)	463.5 (333.8 ,624.5)	-0.02 (-0.03 ,-0.02)	233.7 (184.2 ,289.9)	232.9 (184.1 ,289.5)	5583.7 (4344.6 ,7175)	5620.1 (4311.5 ,7231.1)	349.7 (248.7 ,476.4)	349.7 (248.7 ,476.6)	5815.7 (4033.5 ,8397)	6163.1 (4051.6 ,9127.9)	284.3 (228.4 ,345.9)	288.5 (231.4 ,350.3)	44.8 (20.7 ,77.6)	44.8 (20.7 ,77.6)	227.2 (67 ,423.4)	230 (64.1 ,402.3)	2318.2 (1855.3 ,2904.7)	2318.2 (1855.3 ,2904.7)	340.7 (214.8 ,505.1)	340.7 (217.4 ,500.6)	126 (93.7 ,164.4)	123.1 (92.2 ,160.9)
Both	12904.5 (11129.8 ,15195.1)	13184.2 (11151 ,15686.4)	0.06 (0.05 ,0.07)	877.1 (644.5 ,1169.1)	904.9 (684.3 ,1170.2)	0.05 (0.02 ,0.08)	255.8 (202.8 ,317.5)	254.7 (202.2 ,317.3)	4840.9 (3804.5 ,6206.5)	4864.7 (3737 ,6226.5)	354 (252.5 ,480.4)	354.1 (252.6 ,480.5)	4616 (3239.8 ,6590.3)	4873.9 (3207.2 ,7067.1)	465.2 (377.1 ,559)	478.3 (389.5 ,578.8)	70.1 (34.3 ,117.3)	70.6 (34.6 ,118)	199.1 (52.3 ,407.9)	205.6 (48 ,391.9)	2742.7 (2191.7 ,3417.1)	2749.7 (2197.3 ,3426.2)	747.8 (528.2 ,1044.5)	776 (559 ,1039.2)	130.2 (96.7 ,170)	129.9 (97.1 ,168)
**South Asia**																										

	15203.7	15235.2	0.02	3591.1	3143.5	-0.47	289.6	315.2	6659.9	6901.9	468.5	466.6	3317.5	3543.3	573.5	629.7	33.4	34.2	1571.5	1039.1	3226.5	3226.5	3450.3	2999.8	146.3	148.3
Male	(13560.3	(13575	(0.0007	(2604.4	(2337	(-0.50	(237.6	(261.1	(5455.7	(5631	(361.2	(359.5	(2474.1	(2654	(465.8	(512.5	(14.4	(14.8	(780.6	(493.5	(2570.9	(2571.4	(2460.3	(2189.3	(111.3	(115
	,17042.2)	,17117.1)	,0.05)	,4812.8)	,4112.2)	,-0.45)	,348)	,376.8)	,8119.1)	,8486.4)	,589.4)	,588)	,4416.3)	,4676.4)	,687.1)	,745.4)	,60.5)	,61.9)	,2372.1)	,1595.8)	,4051.1)	,4050.4)	,4676)	,3968.7)	,191.5)	,191.5)
	17368.8	17444.8	0.03	859.7	856.8	-0.02	240.7	265.1	8891.6	8999.9	454.8	453.2	4362.7	4721.9	281.8	303.3	15.7	16.2	1859.6	1416	2413.3	2412.9	715.4	719.5	145.3	138.3
Female	(15436.7	(15543.6	(-0.0002	(614.9	(620.2	(-0.03	(198.5	(220.1	(7318.2	(7436.6	(352.1	(350.3	(3338.1	(3631.3	(227.6	(245	(6	(6.1	(1024.2	(831.8	(1924.1	(1924.5	(475.1	(485.8	(108.8	(106.3
	,19592.7)	,19661.6)	,0.07)	,1169.4)	,1148.3)	,-0.01)	,288.9)	,316.6)	,10798.5)	,10841.6)	,576)	,575)	,5718.8)	,6143.1)	,339.7)	,365.2)	,30)	,30.5)	,2687.9)	,2025.2)	,2994.9)	,2993.9)	,1026)	,1010.2)	,189.2)	,176.8)
	16238.3	16371.3	0.03	2288.9	1969.2	-0.52	266.4	289.7	7725	7978.9	462	459.8	3819.1	4153.4	434.3	462.2	25.1	25.1	1709	1232.4	2836.6	2805.5	2146.1	1828.4	146.2	143.6
Both	(14498	(14610	(0.01	(1664.2	(1472.8	(-0.54	(219.1	(240.3	(6357.5	(6570.2	(356.8	(354.8	(2902.9	(3185.9	(352.1	(376.1	(10.7	(10.7	(899	(670.7	(2271.2	(2247.8	(1522.8	(1325.2	(112.3	(111
	,18228.2)	,18393.1)	,0.06)	,3071.1)	,2574.1)	,-0.50)	,319.6)	,346.1)	,9379.2)	,9674.8)	,583.6)	,581.9)	,5028.5)	,5396.7)	,521)	,549.4)	,45.7)	,46.1)	,2523.7)	,1816)	,3541.4)	,3502.7)	,2923.4)	,2433.6)	,187.9)	,181.3)
**Southeast Asia**																										
	11497.7	11830.3	0.09	1485	1420.7	-0.13	270.7	287.3	4025.6	4157.5	423.7	423.6	3015.9	3360.2	626.1	702	77.8	79.7	398.8	192.2	3194.8	3190.7	1390.5	1325	96.1	97.1
Male	(10225.4	(10515.7	(0.09	(1090.3	(1058.2	(-0.15	(221.8	(236.5	(3262.5	(3368.5	(317.8	(317.3	(2210.7	(2447	(512.4	(574.1	(39.1	(41.1	(122.9	(28.7	(2556.7	(2554.1	(992.8	(964.1	(72	(72.5
	,12957.7)	,13340.2)	,0.10)	,1980.5)	,1852)	,-0.11)	,328.6)	,347.6)	,4972.8)	,5130.9)	,548.6)	,549.5)	,4079.7)	,4576.5)	,747.8)	,839.1)	,130.3)	,132.7)	,701.6)	,380.3)	,3989.8)	,3985.2)	,1890.2)	,1756)	,124)	,124.9)
	13328.6	13782.6	0.11	409.2	398.7	-0.09	229.5	242.9	4902.6	5072.8	418.1	418.2	5081.8	5728.1	302.9	324.5	36.2	37.4	679.7	311.7	2365.9	2363.7	306.9	295.9	102.6	103.2
Female	(11675	(12015.4	(0.11	(303.4	(294.8	(-0.10	(188.5	(200.5	(3956.6	(4085.5	(313.3	(313.3	(3769.2	(4282	(245.9	(264.2	(16.4	(17	(338.1	(109.7	(1902.5	(1901.3	(203.2	(195.2	(77.7	(79.2
	,15325.8)	,15955.2)	,0.11)	,545.1)	,528.9)	,-0.08)	,279.8)	,293.9)	,6047.6)	,6243.6)	,540.3)	,541.2)	,6856.2)	,7810.2)	,367.1)	,390.7)	,63.5)	,64.6)	,1012.8)	,510.4)	,2949.7)	,2948.1)	,440.7)	,424.4)	,131.9)	,131.9)
	12490.5	12901.8	0.11	904.6	860.4	-0.16	248.9	263.5	4497.9	4655.4	420.5	420.5	4140.1	4664.2	452.1	495.6	56	57.4	549.9	257.3	2743.4	2735.1	805.2	760.2	100.4	101.1
Both	(11060.6	(11380	(0.10	(673.4	(651.9	(-0.17	(204.6	(217.7	(3651	(3769.9	(314.8	(314.5	(3103.6	(3482.2	(368.8	(404.6	(27.5	(28.6	(239.5	(70.9	(2208.1	(2201.6	(573.6	(558.9	(75.7	(77.1
	,14216.6)	,14778.3)	,0.11)	,1197.8)	,1115.8)	,-0.14)	,302.4)	,318.6)	,5532.7)	,5726.4)	,543.9)	,544.6)	,5571.5)	,6318.1)	,539.7)	,594.2)	,95.3)	,96.5)	,867.1)	,447.7)	,3416.6)	,3406.5)	,1102.9)	,1013.8)	,129.2)	,129.7)
**Southern Latin America**																										
	10937.6	11031.3	0.05	3300.4	2441.2	-0.96	250.1	253.3	3112.5	3017.6	746.1	708.8	2810	3015.7	1005.3	1099.7	67.4	67.3	211.4	142.9	3230.5	3230.5	3041.3	2152.6	267.5	295.2
Male	(9641.6	(9648.1	(0.03	(2419.8	(1999.2	(-1.04	(197.9	(201.5	(2452.1	(2332.6	(545.2	(518.8	(1904.7	(1933.7	(819.9	(900.2	(31.1	(31.3	(38.3	(21.6	(2595.4	(2595.4	(2158.6	(1712.5	(216.1	(245.2
	,12475.6)	,12814.5)	,0.08)	,4253.2)	,2968)	,-0.89)	,317.4)	,318)	,3947.3)	,3835.6)	,998.1)	,950.6)	,4041.1)	,4486.2)	,1208.8)	,1324.2)	,116.6)	,116.6)	,436)	,330.6)	,4037.4)	,4037.4)	,3995.2)	,2672.8)	,324)	,350.2)
	14064	14605.9	0.16	1150.5	1094.7	-0.17	276	279.9	4452.8	4216.5	833.9	845.3	6269.8	7106.3	434.2	462.8	33	32.8	256.7	200.8	2271.9	2271.9	988.9	928.4	163.2	167.8
Female	(12084	(12207.7	(0.12	(819.4	(793.2	(-0.19	(218.9	(221.5	(3498.5	(3252.4	(610.4	(618.4	(4467.3	(4777.2	(349.9	(375.1	(13.8	(13.5	(66.6	(40.8	(1831.2	(1831.2	(657.3	(624.1	(129.7	(135.2
	,16511.6)	,17751.5)	,0.21)	,1580.2)	,1491.8)	,-0.15)	,345.4)	,350.8)	,5679.6)	,5438.7)	,1115.3)	,1123.8)	,8651.8)	,10378.9)	,526.9)	,558.7)	,60.5)	,60.4)	,443.8)	,356)	,2840.6)	,2840.6)	,1417.3)	,1324.6)	,200.6)	,202.2)
	12695.2	13010.6	0.11	2101.3	1692.8	-0.75	264.8	268.2	3869.6	3683.6	795.1	783.9	4750.9	5282.5	686.3	746.8	48.9	48.9	237.2	175.5	2688.7	2694.4	1896.7	1471.8	209.3	224.9
Both	(11100.7	(11184.3	(0.08	(1633.8	(1395.7	(-0.81	(210.7	(214.4	(3042.8	(2858.8	(581.8	(576.5	(3477.6	(3557.8	(560.9	(612	(22.2	(22.4	(55.6	(36	(2167.7	(2172.5	(1425.1	(1177.8	(169.1	(185.8
	,14680.5)	,15428.5)	,0.15)	,2623.9)	,2062.8)	,-0.69)	,332.7)	,335.7)	,4884.4)	,4713)	,1053.4)	,1042.7)	,6546)	,7677.2)	,820.1)	,894.3)	,85.5)	,85.1)	,440.5)	,343.5)	,3317.8)	,3324.8)	,2423.2)	,1845.4)	,252.8)	,265.3)
**Southern Sub-Saharan Africa**																										
	13071.9	13799.7	0.18	4048.2	4200.3	0.12	198.3	202.6	6113.8	6612	612.4	613.8	2696.6	2957.5	804.8	846.7	25.8	25.3	46.6	37.8	3219.5	3223.6	3610.1	3758.8	455.3	459.4
Male	(11665.1	(12236	(0.17	(3112.2	(3248.2	(0.09	(162.7	(167.6	(5014.3	(5400.2	(471.4	(474.6	(1986.2	(2187.7	(654.5	(689.6	(10.1	(9.8	(11.6	(8.6	(2572.2	(2570.8	(2658	(2801.8	(380.7	(389.5
	,14668.8)	,15520.6)	,0.19)	,5219.9)	,5362.5)	,0.14)	,240.4)	,244.7)	,7455.4)	,8109.7)	,778.2)	,776)	,3573.8)	,3926.4)	,965.3)	,1015.5)	,48.9)	,48.4)	,159)	,139.3)	,4026.7)	,4038.1)	,4790.7)	,4930.5)	,535.5)	,536.6)
	16754.9	17563.8	0.16	1032.8	974.4	-0.21	209.1	213.8	9175.2	9416.3	621	621.7	4842.4	5494.9	395.4	412.3	12.6	12.5	78	76.2	2401.9	2405.8	821.5	756.5	213	219.6
Female	(14811.4	(15492.3	(0.13	(796.2	(765.9	(-0.23	(174.4	(178.1	(7529.8	(7664.7	(481.9	(483.6	(3711.3	(4189.4	(318.6	(333.7	(4.3	(4.2	(15.6	(14.6	(1922.1	(1923.5	(587.1	(551.3	(173.1	(181.7
	,19074.7)	,20009)	,0.20)	,1312.1)	,1226.4)	,-0.18)	,251.2)	,256.9)	,11180.7)	,11555.5)	,786.5)	,786.6)	,6379.7)	,7334.6)	,477.4)	,497.6)	,24.8)	,24.6)	,198.5)	,189)	,2976.6)	,2983.5)	,1094.9)	,999.1)	,260.2)	,262.6)

Both	15241.3 (13554 ,17254.6)	16094.8 (14260.6 ,18254.3)	0.19 (0.17 ,0.22)	2314.1 (1781.8 ,2959.4)	2283.4 (1775.4 ,2904.9)	-0.05 (-0.07 ,-0.03)	204.5 (169.6 ,246)	209.3 (174.2 ,251.3)	7915.7 (6489.9 ,9644.8)	8323.1 (6800.2 ,10208.9)	617.3 (477 ,783.1)	618.5 (479.7 ,781.2)	3953.4 (3018.3 ,5176.9)	4494.5 (3419.5 ,5983)	568.6 (459.6 ,681)	587.2 (478.5 ,703.5)	18.4 (6.9 ,35.2)	17.9 (6.6 ,34.4)	64.6 (14.5 ,180.4)	60.5 (12.7 ,169.8)	2741.6 (2200.8 ,3414.8)	2728.5 (2189.4 ,3401.5)	2007.1 (1472.4 ,2655.3)	1974.5 (1466.4 ,2597.5)	315.4 (262.8 ,372.5)	317.2 (268.4 ,372)
**Tropical Latin America**																										
Male	14432.3 (12849.4 ,16191.5)	15378.1 (13563 ,17369.7)	0.22 (0.20 ,0.24)	4180.2 (3129.3 ,5485.3)	5182.5 (3888.3 ,6776.4)	0.69 (0.63 ,0.74)	249.8 (207.3 ,296.7)	259.3 (215.8 ,308.4)	4594.9 (3773.3 ,5596.5)	4685.3 (3843.1 ,5708)	935.2 (743.8 ,1162)	935.6 (744.1 ,1162.4)	5381.5 (4102.2 ,6974.4)	6328.5 (4819.3 ,8415.2)	541.4 (439.5 ,649.6)	588.8 (479.4 ,706.9)	175.8 (92.7 ,285.6)	176.4 (93 ,286.8)	157 (34.7 ,323.6)	116.9 (17.8 ,249.5)	3234.8 (2573.2 ,4072.8)	3235.4 (2573.5 ,4073.9)	3948.3 (2884.5 ,5259.8)	4924.8 (3602.8 ,6529.5)	241.5 (193.7 ,301.1)	271.9 (220.6 ,332.5)
Female	18911.2 (16812.3 ,21471.8)	20882.9 (18436.9 ,23899.7)	0.34 (0.31 ,0.37)	1165.3 (880.3 ,1508.4)	1221.8 (934 ,1564.8)	0.16 (0.14 ,0.18)	215.9 (180.4 ,255.9)	222.5 (184.7 ,264.9)	7786.2 (6414.6 ,9465.7)	7629.4 (6198.3 ,9365.7)	982.5 (783.8 ,1217.2)	983.3 (784.4 ,1218.5)	8311.9 (6570 ,10598.4)	10727.3 (8323.5 ,13763.6)	268 (215.3 ,323.7)	278.9 (225.3 ,339.1)	81.1 (39.9 ,136.7)	81.2 (40.2 ,135.7)	190.2 (50.8 ,338)	148.5 (30.5 ,268.9)	2421 (1927.2 ,3004.6)	2421.7 (1927.6 ,3005.5)	987.6 (706 ,1331.8)	1043.3 (756.8 ,1384.1)	179.5 (144.7 ,219.9)	180.4 (147.2 ,218.6)
Both	16848.5 (15055.9 ,18983.1)	18429.8 (16302.4 ,20921)	0.32 (0.29 ,0.34)	2549.2 (1911.9 ,3331.1)	2986.9 (2267.7 ,3873.8)	0.56 (0.51 ,0.60)	231.8 (193.4 ,275.3)	239.2 (199.4 ,284.5)	6306.9 (5216 ,7663)	6312.1 (5140.5 ,7699)	960.4 (765.7 ,1191.8)	961.8 (766.8 ,1193)	6971.4 (5500.2 ,8895.6)	8771.9 (6814.7 ,11243.4)	394.8 (319.8 ,474)	417.6 (340.6 ,500.3)	125.9 (64.7 ,203.6)	125 (65.5 ,203.8)	174.9 (44.2 ,332.7)	134.4 (25.3 ,260.7)	2793.4 (2235.1 ,3492.9)	2781.1 (2225.4 ,3476.4)	2346.2 (1702.7 ,3127.3)	2772.7 (2037.2 ,3668.3)	208.5 (168 ,257.4)	221.7 (181 ,269.3)
**Western Europe**																										
Male	13271.3 (11806 ,15034.1)	13778.4 (12208.4 ,15794.7)	0.14 (0.12 ,0.15)	2581.3 (2114.9 ,3087.2)	2928.2 (2425.1 ,3485.3)	0.45 (0.42 ,0.48)	270.7 (227.1 ,322.7)	270.3 (225.6 ,322.8)	4566.2 (3792.4 ,5491.1)	4611.5 (3716.5 ,5706.3)	938.6 (720 ,1206.8)	951.9 (727.1 ,1228.2)	3768.3 (2700.7 ,5231.4)	4274.8 (3009.2 ,5961.5)	930.2 (767.2 ,1110.6)	1012.9 (828.6 ,1204.2)	70.2 (34.5 ,118.8)	71.2 (35.2 ,120.7)	218.6 (51.8 ,419.6)	135.2 (20.8 ,303.8)	3231.5 (2609 ,3985.6)	3231.6 (2608.9 ,3986.8)	2366.1 (1906.1 ,2864.4)	2687.4 (2177.2 ,3244.8)	220.5 (178.7 ,269.7)	247.8 (205.5 ,296.6)
Female	17093.5 (15035.9 ,19717.3)	18308.7 (15866.2 ,21421.4)	0.24 (0.20 ,0.27)	866.4 (672.5 ,1095.9)	994 (821.1 ,1193.1)	0.43 (0.41 ,0.46)	304.7 (252.5 ,363.2)	303.4 (250.5 ,363.9)	6464.2 (5436.1 ,7667.9)	6654.8 (5329.4 ,8294.2)	1100.5 (845.9 ,1410.7)	1126.1 (866.9 ,1447.5)	7374.6 (5504.1 ,9981.1)	8574.1 (6256.4 ,11456.8)	324.9 (265.3 ,391.4)	346.5 (283.9 ,417.4)	29.9 (13 ,53.6)	30.9 (13.7 ,55.2)	319.3 (138.3 ,504)	241.4 (81.3 ,399.6)	2291.5 (1851.6 ,2841.4)	2290.5 (1850 ,2840.8)	708 (509.1 ,936.7)	783.7 (612.1 ,979.1)	159.5 (131.8 ,192.1)	212 (180.6 ,245.4)
Both	15507.2 (13771.3 ,17685.9)	16206 (14210.9 ,18793)	0.17 (0.14 ,0.20)	1603.4 (1298.1 ,1939.3)	1906.7 (1593.9 ,2252.7)	0.58 (0.56 ,0.61)	290.6 (242.7 ,345.5)	288.1 (240 ,343.5)	5678.3 (4770.5 ,6749.3)	5707.3 (4590.8 ,7088.2)	1030.5 (790.6 ,1322.2)	1043.7 (800.2 ,1342.6)	5869 (4395.6 ,7955.4)	6575.7 (4794.6 ,8855.3)	583 (480.5 ,693.4)	658.6 (540.5 ,784.9)	48.3 (23.6 ,82.5)	50.4 (24.7 ,86.3)	277.1 (103 ,473)	192.1 (53.9 ,358.5)	2682.5 (2164.3 ,3292.2)	2726.5 (2197.6 ,3346.6)	1419.7 (1118.5 ,1753.6)	1681 (1366.5 ,2028.2)	186.7 (153.7 ,225.8)	230 (194.4 ,269.8)
**Western Sub-Saharan Africa**																										
Male	13957.4 (12279.5 ,15857.1)	14196.6 (12449.2 ,16203.4)	0.06 (0.04 ,0.07)	1001 (774.6 ,1276.2)	1023.9 (805.9 ,1278)	0.07 (0.06 ,0.08)	185.2 (150 ,228.3)	191.2 (154.8 ,236.1)	7582.5 (6074.4 ,9362.3)	7705.7 (6144.3 ,9567.1)	572.9 (435.5 ,737.9)	571.7 (432.3 ,739.7)	2257.4 (1646.2 ,3053.7)	2331.2 (1680.2 ,3164.3)	755 (614.1 ,902.7)	807.4 (658.1 ,965.7)	28.3 (10.7 ,53)	29 (11.2 ,54.3)	66.2 (24.6 ,198.5)	78.4 (18.8 ,233.3)	3195 (2556.8 ,3991.1)	3193.2 (2555.8 ,3986.2)	888.6 (659.7 ,1166.6)	888 (673.7 ,1141.1)	113.5 (91.9 ,139.5)	137.1 (115.9 ,161.6)
Female	16675.8 (14473.2 ,19228.3)	16692.7 (14466.2 ,19285)	0.02 (-0.00 ,0.04)	879.9 (648.3 ,1178.7)	953.2 (715 ,1260.1)	0.24 (0.21 ,0.26)	224.9 (184.1 ,274.2)	231.4 (189.1 ,281.7)	10514.2 (8373.1 ,13058.6)	10348.2 (8205.4 ,12849.7)	577.4 (438.1 ,745.3)	577.2 (436.4 ,746.8)	3407.7 (2520.5 ,4629.5)	3563.4 (2619 ,4924)	374.9 (302 ,451)	398.3 (322.4 ,478)	13.8 (5.1 ,26.5)	13.7 (5 ,26.5)	73.6 (25.8 ,196.8)	95.9 (20.4 ,237.6)	2374.7 (1909.5 ,2961)	2367.4 (1903.2 ,2951.7)	813.7 (582.3 ,1111.4)	884.7 (649 ,1191.6)	66.7 (49 ,87.7)	69 (51.9 ,90.2)
Both	15343.6 (13410.7 ,17546.4)	15519.4 (13547.5 ,17802.5)	0.04 (0.02 ,0.06)	958.1 (726.3 ,1249.9)	986.9 (765.9 ,1258.4)	0.09 (0.08 ,0.11)	204.6 (166.7 ,250.8)	212.5 (173.3 ,260.4)	9066.1 (7260.3 ,11223.1)	9106.1 (7230.9 ,11309.3)	575 (436.9 ,741.9)	574.6 (434.6 ,744.5)	2847.8 (2113.9 ,3827.8)	2984.4 (2192.2 ,4074.6)	566.3 (459.5 ,677.5)	590.7 (480.4 ,707.2)	21.5 (8.4 ,40.6)	20.8 (8.3 ,39.6)	69.8 (25.5 ,198.6)	87.6 (20.2 ,234.3)	2780.1 (2236.5 ,3464)	2755.1 (2217.6 ,3428.8)	869.2 (639 ,1160.1)	886.7 (667.9 ,1156.4)	89.7 (70.6 ,111.6)	101.1 (82.6 ,122.3)

Note: AAPC-average annual percentage changes; 95% CI-95% confidence intervals(AAPC); 95% UI-95% uncertainty interval

eTable 6. Age Standardised YLDs and AAPC of Mental disorders, Substance use disorders in people aged ≥60 years at 21 GBD regions, from 1990 to 2021

Causes / Regions	Rate (95% UI/95%CI) Mental disorders			Substance use disorders			Schizophrenia		Depressive disorders		Bipolar disorder		Anxiety disorders		Autism spectrum disorders		Attention-deficit/ Hyperactivity disorder		Idiopathic developmental intellectual disability		Other mental disorders		Alcohol use disorders		Drug use disorders	
	1990	2021	AAPC,%	1990	2021	AAPC,%	1990	2021	1990	2021	1990	2021	1990	2021	1990	2021	1990	2021	1990	2021	1990	2021	1990	2021	1990	2021
**Andean Latin America**																										
Male	1760.5 (1274.1,2313.4)	1856.2 (1341.5,2429.3)	0.17 (0.11,0.23)	342.9 (213.2,504.8)	223.8 (142.7,330.6)	-1.01 (-1.19,-0.83)	152.9 (98.9,216.2)	157.5 (105.3,218.4)	645.6 (428.5,949.5)	671.1 (430.2,967.4)	166.2 (96.8,260.3)	165.1 (96,256.1)	457.9 (269.3,737.6)	516.8 (291.1,857.7)	104.1 (69.7,147.5)	116.2 (79.5,165.8)	2.5 (1,4.6)	2.5 (1.1,4.8)	9.6 (2.1,20.1)	6.5 (1,14.5)	221.5 (140.8,331.5)	220.4 (142.8,326.8)	322.1 (198,479.8)	201.9 (124.5,305.7)	20.8 (12.5,31.3)	21.9 (13.5,32.5)
Female	2189.8 (1549.1,2915.1)	2381.3 (1674,3192.4)	0.29 (0.21,0.36)	109.1 (67.9,164.1)	90.4 (56.7,133.5)	-0.60 (-0.62,-0.58)	128.2 (84,181.8)	131.9 (84.6,186.6)	922.1 (597.8,1332.6)	983.7 (629.1,1438.1)	167.8 (98.5,263.8)	167.3 (98.1,260.5)	747.5 (449.1,1165.6)	873.1 (529.8,1412.8)	51.1 (33.5,73.9)	55.5 (36.9,80.1)	1.1 (0.4,2.3)	1.1 (0.4,2.3)	10.4 (3.4,19.8)	7.8 (2.2,14.9)	161.5 (103,242.9)	160.8 (101.3,241.8)	89.3 (52.8,139.1)	70 (40.7,108.8)	19.8 (10.3,33)	20.4 (10.9,33.2)
Both	1982.4 (1420.2,2622.7)	2131.6 (1527.9,2814.1)	0.25 (0.17,0.31)	222.7 (140.6,324.2)	153.9 (100.8,224.1)	-0.95 (-1.08,-0.82)	140.3 (92.8,194.2)	144.2 (96.2,197.3)	788.2 (518.8,1137.4)	834.9 (538.4,1210.2)	167.1 (99.6,259.2)	166.3 (98.3,256.8)	607.6 (369.9,947.8)	703.7 (416.4,1130.6)	76.8 (52.3,108.1)	84.5 (57.7,120)	1.8 (0.8,3.3)	1.8 (0.8,3.3)	10.1 (2.8,19.7)	7.2 (1.7,14.5)	190.5 (122.4,283.5)	189.1 (122,281.3)	202.4 (125,300.3)	132.8 (83.4,200)	20.3 (12,30.7)	21.1 (13.1,31.5)
**Australasia**																										
Male	1826.3 (1351.7,2330.3)	1854.1 (1364.4,2391.8)	0.05 (0.04,0.06)	241.5 (155.8,348.3)	260.1 (173,372.2)	0.24 (0.21,0.27)	197.7 (133.4,266.5)	200.8 (137,269.4)	617.2 (399.5,898.8)	615.6 (383.8,940.3)	190.7 (115.1,291.4)	190.9 (111.9,290.5)	361.4 (214.8,575.5)	366.6 (206.2,603.7)	224.9 (153.3,320.1)	243.9 (165.6,341.3)	2.7 (1.1,5.1)	2.7 (1.1,5.2)	1.5 (0,5.5)	1.9 (0,6.8)	230.2 (148.7,338.3)	231.7 (150.3,332)	204.5 (127.9,303.6)	220.6 (140,328.2)	37 (20.1,58.4)	39.5 (22,62.4)
Female	2087 (1499.7,2728)	2063 (1494.3,2729)	-0.02 (-0.04,0.0005)	119.1 (72.5,176.5)	136.1 (85.7,197.7)	0.43 (0.42,0.45)	214.8 (143.3,289.6)	217.6 (145.5,289.9)	750 (498.5,1071)	696.4 (436.8,1055.6)	214.7 (129.5,321.1)	215.2 (132.6,326.2)	618.7 (383,950.2)	635.9 (372.9,1008.9)	91.6 (60.6,131.5)	96.7 (63.7,138.8)	1.2 (0.4,2.5)	1.2 (0.4,2.6)	5.4 (1.2,11.6)	9.9 (2.9,19.3)	190.6 (122.2,278.6)	190.1 (121.3,278.2)	81 (44.3,130.9)	92.2 (52.2,144.9)	38.1 (21.4,60.2)	43.9 (23.9,68.3)
Both	1968.1 (1437.4,2547.4)	1963.6 (1439.7,2560.6)	0.0027 (-0.01,0.02)	175.4 (115.8,248.7)	195.5 (132.5,277.2)	0.34 (0.32,0.35)	207.3 (145.8,272)	209.6 (148.9,271.3)	688.8 (456,978)	658.3 (417.2,981.6)	203.7 (124.3,300.8)	203.6 (125.8,302.6)	502.2 (313,773.6)	507.3 (300.9,804.6)	152.1 (103.4,214.4)	166.9 (113.7,234.7)	1.9 (0.8,3.6)	1.9 (0.8,3.6)	3.7 (0.7,8.3)	6.1 (1.7,12.8)	208.4 (135.3,300.6)	209.8 (136.5,300.6)	137.7 (85.5,202.6)	153.7 (98.7,226.5)	37.8 (22.5,56.3)	41.8 (25.2,61.4)
**Caribbean**																										
Male	1932.5 (1391.8,2539.8)	1989.8 (1457.1,2621.7)	0.11 (0.09,0.13)	333.1 (213.6,484.6)	333.2 (222.5,471.8)	0.20 (0.02,0.41)	152.4 (101.2,211)	153.6 (103.9,210.1)	883 (571.6,1284.3)	907 (590.1,1327.1)	165.4 (96.9,258.4)	162.8 (96,251.6)	386.2 (229.6,610.4)	419.1 (245.3,666.8)	114.6 (77.3,160.8)	118.8 (81.5,166.6)	3.9 (1.9,6.8)	3.9 (1.9,6.7)	4.8 (0.6,12.7)	4.8 (0.5,12.2)	222.1 (142.1,329.7)	219.7 (142.1,326.8)	310 (195.2,456.5)	310.8 (204.2,445.3)	23.1 (14.2,34.5)	22.4 (14.3,32.6)
Female	2464.3 (1752.8,3308.9)	2511.2 (1800.4,3371.6)	0.09 (0.06,0.12)	96.8 (62,142.3)	93.5 (59.2,136.1)	-0.11 (-0.12,-0.10)	125.9 (83.6,173.5)	126.9 (84.8,173.8)	1319.9 (855.6,1911.4)	1306.6 (861.7,1909.9)	169.2 (101.2,261)	167.4 (100.4,255.6)	623.8 (379.1,980.1)	685.5 (412.6,1087.7)	55.9 (37.3,78.8)	57 (38.3,80.1)	1.5 (0.6,2.7)	1.4 (0.6,2.8)	6.1 (1,13.7)	6.1 (1.1,13)	162.1 (103.9,243)	160.4 (102,242.2)	74.2 (43.8,116.2)	72.3 (43,111.3)	22.5 (13.2,35.1)	21.2 (12.2,32.7)
Both	2207.7 (1577.6,2939.6)	2268 (1647.5,3013.1)	0.11 (0.08,0.14)	211 (137.9,302.7)	206 (138.5,289.1)	0.07 (-0.08,0.22)	138.8 (93.9,189.3)	139.5 (95.5,189)	1108.6 (720.7,1612.2)	1119.7 (739.5,1623.5)	167.4 (99.4,259.6)	165.2 (99.2,253.4)	509.6 (312.5,799.1)	561.5 (341.8,884.6)	84.2 (57.4,117.1)	85.9 (58.6,120.3)	2.7 (1.3,4.6)	2.6 (1.3,4.6)	5.5 (0.8,13)	5.5 (0.8,12.6)	190.9 (123.3,284.2)	188 (121.8,278.9)	188.2 (119.4,276.5)	184.2 (120,263.2)	22.8 (14.3,33.7)	21.8 (13.8,31.7)
**Central Asia**																										
Male	1549.4 (1145.5,2009.4)	1591.8 (1166.2,2071.1)	0.09 (0.09,0.10)	575.3 (361.5,824.8)	518.5 (339.5,734.5)	-0.32 (-0.38,-0.27)	120.6 (80.1,167.2)	124 (82.4,169.2)	733.6 (483.7,1043)	750.2 (491.7,1084.6)	108.8 (63,170.3)	108.8 (63.5,168.7)	209.9 (127.2,329)	226.1 (137.2,365.4)	144.1 (97.6,203.1)	152.8 (103.8,213.2)	1 (0.4,1.9)	1 (0.4,1.9)	9.8 (1.7,22)	8 (1.4,18.2)	221.6 (143.4,331.7)	221 (143.2,330)	540.2 (331,784.5)	479.6 (305.9,689.3)	35.1 (21.9,51.7)	38.9 (25.8,54.5)

	2342.3	2341.4	0.00	267.2	169.3	-1.38	115.9	118.4	1521.8	1487.7	112.6	112	347.5	378.7	73	74.9	0.5	0.5	9.9	8.9	161.2	160.4	218.8	117.8	48.3	51.4
Female	(1671.7	(1656.8	(-0.01	(175.3	(115.6	(-1.49	(78.1	(78.2	(1012.6	(977.9	(65.5	(66	(205.8	(227.8	(49.9	(51.2	(0.2	(0.2	(2.4	(2.3	(103.3	(102.2	(137	(75.4	(28.4	(32.2
	,3114.3)	,3149.9)	,0.02)	,378.8)	,234.2)	,-1.26)	,159.2)	,161.1)	,2148.7)	,2132.8)	,172.8)	,172.8)	,552.4)	,617.9)	,102.8)	,105.6)	,1)	,1)	,20)	,17.8)	,241.3)	,238.5)	,324.9)	,173.4)	,74.1)	,73.7)
	2054.1	2030.5	-0.03	383.9	312.8	-0.62	117.7	120.9	1233.9	1180.7	111.1	110.6	297.5	315.7	99.8	107.8	0.7	0.7	9.9	8.6	183.5	185.6	340.4	266.3	43.6	46.5
Both	(1484	(1458.6	(-0.04	(250.4	(209.5	(-0.69	(80.8	(82.1	(824.3	(778.9	(65.5	(65.7	(178.1	(193.2	(67.9	(74.4	(0.3	(0.3	(2.3	(2	(118.1	(120.4	(213.2	(171.6	(27.1	(30.6
	,2705.7)	,2695.7)	,-0.01)	,540)	,435)	,-0.57)	,160)	,162.9)	,1740.9)	,1691.6)	,171.3)	,169.7)	,470)	,510.8)	,139.6)	,150.9)	,1.3)	,1.4)	,20.5)	,18)	,274.1)	,274.4)	,491.1)	,382)	,64.8)	,65)
**Central Europe**																									
	1417.3	1491.7	0.17	428.5	451.9	0.18	124.2	132	490	506.2	107.2	108	312.7	345.3	155.4	174.6	1	1	8.9	4.8	218.1	219.7	408.1	429.7	20.4	22.1
Male	(1061.3	(1112.6	(0.16	(290.1	(302.9	(0.15	(85.2	(92.6	(334.1	(344.3	(64.9	(65.2	(192.9	(214.4	(106.6	(120.8	(0.4	(0.4	(1.6	(0.2	(140.9	(142.6	(272.7	(285.1	(13	(14.7
	,1811.9)	,1892.6)	,0.18)	,602.5)	,633.1)	,0.21)	,165.8)	,172.4)	,686.7)	,709.7)	,162)	,163)	,484.3)	,535.5)	,217.5)	,244.3)	,1.8)	,1.8)	,19.5)	,12.6)	,323.7)	,324.6)	,577.8)	,606.5)	,29.8)	,31.2)
	2232.5	2287.1	0.09	309.4	240.4	-0.67	122.3	127.6	1156.9	1125.1	115.6	115.9	585.2	664.1	79.6	84.8	0.4	0.4	11	7.2	161.5	161.9	288.3	217.6	21.1	22.8
Female	(1615.9	(1653.4	(0.07	(196.5	(152.5	(-0.87	(84.5	(89.1	(773.9	(749.2	(70.2	(71.5	(370.9	(420.5	(54.5	(58.1	(0.2	(0.2	(3.2	(1.6	(104.9	(104.4	(178.7	(134.9	(13.2	(14.7
	,2958.1)	,3026.8)	,0.12)	,455.8)	,347.9)	,-0.48)	,162.8)	,168.9)	,1639)	,1573.4)	,175.5)	,175.4)	,895.3)	,1015)	,110.7)	,118.4)	,0.9)	,0.9)	,21)	,14.6)	,240)	,242.2)	,431.7)	,321.9)	,31.2)	,33.3)
	1896.4	1943.7	0.09	361.9	334.5	-0.19	123.1	129.6	881	857.2	112	112.4	473.4	527.2	111.3	123.7	0.7	0.7	10.1	6.2	184.8	186.8	340.9	311.8	21	22.8
Both	(1393.4	(1421.8	(0.07	(238.9	(222.3	(-0.29	(86.2	(91.2	(594.9	(577.2	(68.3	(69.1	(303.1	(335.4	(76.2	(85.5	(0.3	(0.3	(2.6	(1.2	(120.4	(121.5	(221.4	(203.7	(13.4	(15.2
	,2477)	,2526.8)	,0.11)	,514.2)	,469.7)	,-0.09)	,163.5)	,170)	,1242.9)	,1194.5)	,169.3)	,169.2)	,722.1)	,804.4)	,154.6)	,172.9)	,1.3)	,1.3)	,20.3)	,13.7)	,273.8)	,277.6)	,490.2)	,443.9)	,30.7)	,32.4)
**Central Latin America**																									
	1795.4	1902.5	0.18	436.7	360.4	-1.10	152.7	157.3	801.5	859.5	154.1	154.4	341.8	375.1	118.2	129.9	1.4	1.4	5	3.6	220.6	221.4	412.9	336.9	23.8	23.5
Male	(1331.4	(1384.4	(0.14	(284.3	(234.1	(-1.38	(107.9	(110.9	(534.5	(563.5	(95.1	(94.9	(216.3	(233.1	(81.2	(89	(0.6	(0.6	(0.6	(0.3	(144.4	(143.3	(265.2	(214.5	(15.4	(15.5
	,2324.1)	,2467.1)	,0.21)	,625.9)	,517.6)	,-0.83)	,203.1)	,206.7)	,1128)	,1208.3)	,229.8)	,231.1)	,518.1)	,575.3)	,163.3)	,183.7)	,2.5)	,2.5)	,12.2)	,9.6)	,326.5)	,328.8)	,598.4)	,488)	,33.3)	,32.7)
	2062.5	2176.7	0.15	92.5	77.7	-0.54	131.2	133.1	1016.4	1063.4	156	155.4	530.6	593.9	57.8	61.3	0.6	0.7	6.8	5.6	163.1	163.3	67.6	54.5	24.9	23.2
Female	(1474.2	(1537.7	(0.10	(60.7	(51	(-0.57	(91.8	(93.6	(666.7	(690.1	(97.4	(96.4	(342.6	(374.8	(40.2	(41.4	(0.2	(0.3	(1.4	(1	(105.2	(105.3	(41.5	(33.2	(15.8	(14.5
	,2715.1)	,2868.5)	,0.20)	,130.9)	,110.4)	,-0.51)	,172.1)	,174)	,1441.4)	,1511.2)	,231.6)	,230.6)	,790.1)	,890.2)	,81.5)	,85.6)	,1.2)	,1.3)	,14)	,11.8)	,243.3)	,245)	,101.7)	,83.4)	,36.5)	,34.5)
	1934.1	2051.2	0.17	258.1	207	-1.10	141.6	144.3	912.8	969.6	155.1	155	440.2	494.2	86.8	92.7	1	1	5.9	4.7	190.7	189.7	233.7	183.7	24.4	23.4
Both	(1401.9	(1470.9	(0.13	(169.4	(136	(-1.32	(100.8	(102	(609.3	(634.1	(96.4	(95.8	(284	(312.2	(60.2	(63.3	(0.4	(0.4	(1	(0.7	(124.6	(123.2	(149.8	(116.4	(15.8	(15.4
	,2516.3)	,2673.4)	,0.21)	,368.2)	,294.7)	,-0.91)	,184.5)	,188.6)	,1276.4)	,1364.1)	,230)	,230.5)	,656.8)	,742.6)	,120.4)	,130.5)	,1.9)	,1.8)	,13.1)	,10.7)	,283)	,282.6)	,339.8)	,266.9)	,34.4)	,33)
**Central Sub-Saharan Africa**																									
	2477.5	2536.2	0.08	251.5	267.5	0.18	107.6	107	1611.6	1644	118.4	119.3	294.8	308.1	126.1	135	0.4	0.4	2.9	5.8	215.8	216.6	226	237.5	25.5	30
Male	(1734.1	(1771.1	(0.06	(156.4	(167.7	(0.12	(67.6	(67.8	(1043.5	(1042.4	(68.2	(67.4	(174.9	(173.8	(85.3	(91.3	(0.1	(0.1	(0.5	(0.7	(138.3	(137.7	(135.2	(143.1	(13.1	(17.6
	,3312.4)	,3420)	,0.09)	,381.7)	,387.8)	,0.22)	,155.6)	,151.4)	,2356.9)	,2431.7)	,185.3)	,186.8)	,468.8)	,500.9)	,179.5)	,191.3)	,0.8)	,0.8)	,9.9)	,15.3)	,320.6)	,323.8)	,354.5)	,354.2)	,40.8)	,46.5)
	2694.1	2790.9	0.14	79.5	74.1	-0.22	112.9	112.3	1763.4	1821.4	119.3	119.2	474.9	506.8	62.4	66.4	0.2	0.2	3.2	6	157.9	158.6	62.6	55.7	16.9	18.4
Female	(1861.8	(1942.3	(0.11	(47.9	(45.4	(-0.24	(70.5	(72.3	(1128.8	(1159.4	(67	(68.8	(284.5	(292.1	(40.2	(44	(0.1	(0.1	(0.7	(1	(100.9	(100.9	(35.1	(31.8	(7.8	(8.9
	,3627.5)	,3785.9)	,0.18)	,119.5)	,110.5)	,-0.20)	,161.5)	,161.7)	,2579)	,2678.6)	,189.1)	,188.7)	,756.5)	,828)	,90.4)	,97.1)	,0.4)	,0.4)	,8.9)	,15)	,234.7)	,239.3)	,100.2)	,88.3)	,29.1)	,31.6)
	2593	2681.4	0.12	159.3	161	0.02	110.4	110	1692.8	1743.8	118.9	119.2	390.5	422.3	92.2	96.4	0.3	0.3	3.1	6	185	183.4	138.3	137.9	21	23.1
Both	(1812.1	(1861.5	(0.10	(101.1	(103.2	(-0.02	(71.7	(72.8	(1090	(1114.2	(68.7	(69.5	(238.3	(247	(61.9	(66.3	(0.1	(0.1	(0.7	(0.9	(120.1	(117.6	(84.2	(84.1	(12.2	(14
	,3472)	,3601.5)	,0.15)	,236.5)	,230.6)	,0.05)	,152.8)	,152.3)	,2461)	,2558.1)	,185.2)	,185.6)	,618.9)	,679.3)	,129.3)	,136.2)	,0.6)	,0.5)	,9.3)	,15)	,273.9)	,273.1)	,214)	,204)	,32.5)	,34.8)
**East Asia**																									
	1599.2	1744.5	0.26	192.9	185.8	-0.16	181.6	187.1	631.9	749.3	46	45.8	385.1	388.8	117.7	140	2.2	1.9	6.4	4	228.4	227.5	134.6	159	58.3	26.8
Male	(1190.1	(1304.2	(0.24	(127.4	(118.8	(-0.24	(131.4	(136.4	(437.1	(516.7	(28.3	(28.2	(250.3	(252	(80.5	(95.4	(1	(0.9	(1.2	(0.4	(146.1	(147.2	(81.7	(96.8	(37.4	(17.3
	,2042.2)	,2238.6)	,0.28)	,267.4)	,265.7)	,-0.10)	,231.2)	,238.9)	,868.1)	,1038.7)	,68.5)	,69.1)	,567.7)	,568.3)	,165.6)	,196.7)	,3.8)	,3.4)	,15.1)	,10.1)	,344.2)	,341.1)	,200.1)	,234.9)	,84)	,38.6)

Female	2007.8 (1467.3 ,2644.8)	2208.6 (1610 ,2905.3)	0.30 (0.28 ,0.32)	123.5 (82.8 ,170.1)	77.5 (50.9 ,107.9)	-1.55 (-1.61 ,-1.50)	166.9 (122.3 ,211.4)	172.8 (127 ,218.2)	971.9 (668 ,1334.8)	1153 (798.1 ,1585.1)	44.8 (28 ,67.5)	44.8 (28 ,67.1)	607.5 (404.2 ,881.5)	619 (410.2 ,889.6)	39 (26.4 ,54.7)	43.7 (29.2 ,61.9)	1 (0.4 ,1.8)	0.9 (0.4 ,1.6)	9 (3.1 ,17.1)	6.9 (2.2 ,13)	167.8 (108.8 ,253.1)	167.5 (107.6 ,250.8)	37 (22 ,57.2)	37 (21.9 ,56.8)	86.5 (55.7 ,124)	40.5 (25.3 ,59.6)
Both	1819.6 (1345.3 ,2363.7)	1988.7 (1472 ,2583.9)	0.29 (0.27 ,0.30)	157.3 (105.3 ,214.8)	129.5 (84.3 ,181.9)	-0.68 (-0.73 ,-0.62)	174.3 (127.1 ,219.9)	179.9 (131.9 ,228.1)	813.8 (561.2 ,1117.6)	960.9 (664.9 ,1325.1)	45.2 (28.1 ,67.4)	45.1 (28.1 ,67.7)	504.8 (333.8 ,730.3)	510 (338.9 ,735.5)	76.4 (52 ,107.6)	89.8 (60.8 ,126.3)	1.6 (0.8 ,2.8)	1.4 (0.6 ,2.5)	7.8 (2.2 ,15.9)	5.5 (1.5 ,11.6)	195.9 (125.4 ,293.9)	195.9 (126.4 ,293.3)	84 (50.9 ,125.4)	95.6 (58 ,141.2)	73.3 (47.3 ,104.7)	33.9 (21.8 ,49.2)
**Eastern Europe**																										
Male	1858.9 (1345.3 ,2396.5)	1897.6 (1381 ,2444.1)	0.06 (0.06 ,0.07)	860.7 (555.9 ,1200.6)	847.4 (554 ,1188)	-0.09 (-0.14 ,-0.05)	110 (78.7 ,143.8)	116.4 (82.6 ,150.6)	970.5 (643.2 ,1358.3)	950.1 (631 ,1351.4)	109.9 (67.8 ,163.9)	110.3 (69 ,164.2)	290 (189.3 ,421.8)	328.4 (210.3 ,481.4)	151 (103.9 ,212.3)	165.2 (113.2 ,231.4)	0.9 (0.4 ,1.7)	0.9 (0.4 ,1.7)	5.1 (0.5 ,13.4)	4.3 (0.3 ,11.8)	221.4 (143.2 ,333)	222.1 (143.9 ,334.5)	783.9 (504.6 ,1114.7)	787.2 (505.8 ,1115.9)	76.7 (49.2 ,109.3)	60.2 (40.2 ,83.2)
Female	2308.2 (1638 ,3049.2)	2319 (1658.7 ,3037.4)	0.01 (0.0045 ,0.02)	306.5 (203.1 ,420.8)	232.6 (156.3 ,319.9)	-0.81 (-0.92 ,-0.71)	111.5 (79.5 ,145.3)	115.4 (81.9 ,149)	1293.4 (854.3 ,1829.9)	1219.9 (806.6 ,1727)	115 (71.6 ,171.9)	114.4 (71.7 ,169.4)	538.7 (355.1 ,779.6)	617.7 (407.4 ,896.4)	76.4 (52.2 ,105.3)	79.7 (55.3 ,110.6)	0.4 (0.1 ,0.8)	0.4 (0.2 ,0.8)	7 (1.1 ,15.3)	6.4 (0.9 ,13.8)	165.8 (107.2 ,248.3)	165.2 (106.8 ,248.8)	234.2 (147.1 ,340)	172.5 (109.4 ,248.5)	72.4 (46.8 ,104.5)	60.1 (39.2 ,85)
Both	2165.7 (1546.6 ,2829.2)	2162.7 (1563.2 ,2811.2)	0.00 (-0.01 ,0.0041)	491.9 (321.9 ,681.5)	465.9 (307 ,646.8)	-0.16 (-0.19 ,-0.14)	111 (79.8 ,144.1)	115.8 (83 ,148.8)	1189.3 (786.8 ,1677.8)	1118.5 (742.1 ,1588.2)	113.3 (71.2 ,168.8)	112.8 (70.7 ,166.9)	461.1 (304.4 ,667.3)	511.8 (338.5 ,742.3)	100.7 (69 ,139.3)	111.5 (77.3 ,155.2)	0.6 (0.2 ,1.1)	0.6 (0.3 ,1.2)	6.4 (1 ,14.7)	5.6 (0.7 ,12.9)	183.4 (118.4 ,276.7)	186 (120 ,279.2)	417.2 (266.9 ,597.9)	405.1 (259.3 ,575.8)	74.7 (48.4 ,107.5)	60.8 (40.3 ,84)
**Eastern Sub-Saharan Africa**																										
Male	2474 (1760.5 ,3254.9)	2552.2 (1815 ,3358.4)	0.12 (0.11 ,0.13)	381.8 (243.6 ,547.4)	377.4 (247.4 ,534.4)	0.07 (-0.02 ,0.16)	104.9 (72.6 ,141.6)	109.3 (76.2 ,145.7)	1598.4 (1061.1 ,2240.5)	1638.7 (1072.8 ,2296.2)	130.1 (79.2 ,197.6)	130.8 (79.4 ,197.9)	286.3 (177.2 ,437.8)	306.6 (189 ,471.1)	128.4 (88.4 ,178.8)	139.3 (95.3 ,195.3)	0.3 (0.1 ,0.7)	0.3 (0.1 ,0.7)	5.5 (1.4 ,13.6)	5.4 (1 ,14.3)	220 (143.3 ,326.6)	221.8 (144.2 ,328.8)	354.5 (221.6 ,516.1)	344.6 (219.3 ,497.9)	27.3 (17.5 ,39.4)	32.8 (21.8 ,45.6)
Female	3079.5 (2156.9 ,4074.8)	3129.9 (2207.4 ,4147.2)	0.07 (0.05 ,0.09)	133.9 (87.5 ,189.4)	124.8 (84.2 ,176.5)	-0.27 (-0.34 ,-0.22)	109.8 (75.9 ,145.6)	113.7 (79.3 ,151.9)	2121.1 (1394.8 ,2969.4)	2116.3 (1390.9 ,2984.6)	131.1 (79.8 ,197.8)	131.5 (80.2 ,198.1)	487.9 (307.9 ,743)	533.1 (340.5 ,810.5)	62.9 (43.5 ,87.6)	67.3 (45.9 ,93.6)	0.2 (0.1 ,0.4)	0.2 (0.1 ,0.4)	6.1 (1.9 ,13.4)	6.2 (1.3 ,14.4)	160.5 (103.7 ,240.4)	161.7 (104.9 ,240.5)	114.8 (73.7 ,166.6)	106.4 (70.1 ,155)	19 (11.2 ,29.2)	18.4 (11.3 ,27.6)
Both	2777.2 (1960.9 ,3656.9)	2859.8 (2029.4 ,3767.9)	0.11 (0.09 ,0.13)	261.8 (170.2 ,373.3)	246.2 (164 ,346.6)	-0.16 (-0.26 ,-0.0011)	107.3 (75.2 ,143)	111.6 (78 ,148.9)	1859.8 (1229.1 ,2598.1)	1892.5 (1242 ,2662.5)	130.6 (79.7 ,197.3)	131.2 (80.3 ,197)	387.5 (246 ,589.2)	427.7 (272.7 ,648.4)	95.8 (66.3 ,132.9)	101.1 (69.1 ,140.5)	0.3 (0.1 ,0.5)	0.2 (0.1 ,0.5)	5.8 (1.7 ,13.6)	5.8 (1.2 ,14.2)	190.1 (123.5 ,282.5)	189.6 (123.5 ,279.6)	238.7 (152.1 ,345.2)	221.3 (143.4 ,318.4)	23.1 (14.7 ,33.8)	24.9 (16.4 ,35.2)
**High-income Asia Pacific**																										
Male	1336.8 (1019.5 ,1651.7)	1403.8 (1070.3 ,1734.3)	0.16 (0.16 ,0.17)	148.2 (95.9 ,212.5)	184.8 (119.1 ,260.7)	0.58 (0.53 ,0.62)	146.9 (104.2 ,190.8)	142.7 (101.2 ,187.2)	347.6 (236.5 ,480.5)	375.8 (250.2 ,531.2)	116.4 (72.5 ,173.2)	115.3 (71 ,172.3)	216.4 (141 ,319.5)	228.4 (144.9 ,338.7)	278.1 (191 ,385.1)	311.6 (214.4 ,435.5)	0.8 (0.3 ,1.6)	0.8 (0.3 ,1.6)	4.4 (0.3 ,12.2)	1.2 (0 ,4.6)	226.2 (146.8 ,332.2)	227.9 (147.1 ,336.5)	121.7 (75.5 ,182.8)	160.2 (99.7 ,233.3)	26.5 (17 ,37.9)	24.6 (15.9 ,35.5)
Female	1454.2 (1069.9 ,1857.1)	1556.7 (1141.7 ,2000.6)	0.22 (0.21 ,0.24)	76.3 (49.8 ,106.7)	71.9 (46.3 ,103.1)	2.03 (1.96 ,2.09)	167.4 (120.3 ,215)	171.4 (122 ,222.2)	520.9 (349.7 ,725.1)	567.2 (378.7 ,798.2)	133.1 (82.7 ,197.1)	132.5 (82.8 ,197)	352 (229.3 ,522.4)	395 (250 ,585.4)	111.9 (76.8 ,155.5)	126.2 (86.8 ,175.8)	0.3 (0.1 ,0.6)	0.3 (0.1 ,0.6)	5.8 (1.5 ,11.8)	0.7 (0 ,2.6)	163 (105.1 ,246.2)	163.3 (105.8 ,245)	47.6 (28.5 ,72.3)	45.1 (26.3 ,71.6)	28.6 (17.9 ,42.1)	26.8 (16.4 ,39.6)
Both	1407.8 (1053.9 ,1775.6)	1487.3 (1113.7 ,1869.4)	0.18 (0.17 ,0.19)	107.7 (70.3 ,151.4)	126 (83.1 ,175.6)	1.25 (1.20 ,1.30)	158.9 (114.5 ,204.7)	158 (113.5 ,204.9)	449.7 (303.8 ,623.3)	479.6 (320.8 ,676.3)	125.9 (78.2 ,186.7)	124.3 (77.4 ,184.5)	294.9 (192.8 ,435.2)	317.1 (203.7 ,466)	182.9 (125.5 ,253.5)	213.3 (146.5 ,297.1)	0.5 (0.2 ,1)	0.5 (0.2 ,1.1)	5.2 (1.1 ,11.9)	1 (0 ,3.5)	189.7 (123.2 ,282.5)	193.5 (125.5 ,287.1)	79.9 (50.3 ,119.4)	100.3 (63.3 ,144.6)	27.8 (17.7 ,39.7)	25.7 (16.5 ,36.9)
**High-income North America**																										
Male	1593.6	1654.6	0.14	271.4	325	0.58	195.4	184.8	431.2	472.9	119.4	117.9	391.7	420.1	191.1	199.9	1.1	1.2	10.6	8.4	253	249.4	219.9	203	51.5	121.9

	(1207.8	(1247.1	(0.12	(179	(224.6	(0.53	(141.4	(134.7	(292.2	(315.8	(78.1	(77.6	(254.6	(274.5	(131.4	(139.1	(0.5	(0.5	(2.9	(1.7	(164.7	(163.5	(138.5	(129.2	(34.5	(83.4
	,2007.8)	,2077.8)	,0.17)	,375.9)	,435.4)	,0.62)	,251.4)	,238.2)	,593.5)	,662.4)	,171)	,168)	,575.4)	,620)	,264.2)	,278.1)	,2.1)	,2.2)	,21.4)	,18)	,369.2)	,365.7)	,316.5)	,289.1)	,72)	,161.1)
	2004.5	2160	0.27	159.1	288.6	2.03	200.5	199.8	677.8	772.3	124	123.6	747.4	812.7	86.8	88.5	0.6	0.6	11.7	9.7	155.6	152.8	95	101.5	64.1	187
Female	(1473.1	(1587.8	(0.23	(108	(202.1	(1.96	(145.4	(145.9	(462.2	(523.5	(82.3	(82.1	(500.8	(545.8	(59.5	(60.5	(0.2	(0.2	(4.3	(3.1	(100.6	(99.4	(59.2	(64.2	(41.6	(127.5
	,2605.4)	,2796.2)	,0.32)	,217.8)	,380.8)	,2.09)	,259.2)	,254.9)	,942.8)	,1076.1)	,176.6)	,174.9)	,1065.3)	,1156.9)	,119.8)	,122.8)	,1.2)	,1.2)	,20.6)	,17.7)	,227.7)	,222.8)	,139)	,147.3)	,89.6)	,252.2)
	1827.3	1924.8	0.21	209.4	305.4	1.25	198.6	192.9	570.9	632.8	122	120.9	594.4	630.5	132	140.2	0.8	0.9	11.3	9.1	197.3	197.5	150.7	148.9	58.7	156.4
Both	(1357.2	(1438.4	(0.17	(140.6	(213.6	(1.20	(144.7	(140.7	(389.6	(427.2	(80.4	(80	(398	(421.8	(91	(96.8	(0.4	(0.4	(3.9	(2.8	(129	(129.8	(95.1	(94.6	(38.7	(106.8
	,2339)	,2463.9)	,0.25)	,286.5)	,402.9)	,1.30)	,256.2)	,245.8)	,792.1)	,882)	,173.8)	,171)	,851.6)	,905.5)	,182.3)	,194.6)	,1.6)	,1.6)	,21)	,17.7)	,287.7)	,288.3)	,217.6)	,212.7)	,81.1)	,208.5)
**North Africa and Middle East**																										
	1775.3	1832.5	0.12	79.6	80.3	0.02	130.2	134.6	764.5	791.4	144.9	143.7	358.3	384.5	118.6	132.2	2.1	2	36.6	25	220.2	219.2	51.1	46.9	28.6	33.4
Male	(1301.3	(1332.4	(0.11	(52	(53.5	(0.0015	(89.9	(93	(500.5	(518.6	(87.7	(86.6	(222.9	(234.4	(81.2	(90.4	(1	(0.9	(15.5	(8.9	(143.4	(142.7	(29.8	(27.8	(17.9	(21.7
	,2301.2)	,2377.8)	,0.15)	,112.8)	,111.5)	,0.03)	,174.8)	,179.2)	,1091.2)	,1144.1)	,221)	,219.3)	,548.9)	,590.3)	,164.6)	,185.8)	,3.5)	,3.4)	,63.5)	,45.8)	,328.9)	,324.8)	,78.3)	,71.3)	,41.5)	,47.3)
	2300.9	2424.5	0.18	54.2	53.9	-0.02	121.6	123.9	1150.3	1212.3	154.4	153.2	622.4	688.4	59.2	63.6	1.1	1.1	31.3	22.9	160.7	159.1	20.3	19.2	33.9	34.7
Female	(1631.6	(1715.1	(0.15	(34.5	(35.4	(-0.03	(83	(86.1	(749.2	(784.6	(93.1	(92.3	(390.6	(426.9	(40.7	(43.6	(0.5	(0.5	(13.8	(9.6	(104.1	(103	(11.4	(10.7	(20.3	(21.7
	,3068.3)	,3236.9)	,0.21)	,77.1)	,76.1)	,-0.01)	,161.9)	,164.5)	,1636.5)	,1768.8)	,237.2)	,232.6)	,957.9)	,1055.1)	,82.9)	,88.6)	,2.1)	,2.1)	,53.4)	,40.4)	,240.3)	,237.8)	,33.3)	,31.2)	,51.7)	,51)
	2035.8	2127.2	0.16	67.1	67.2	0.0018	126	129.3	955.2	1000.4	149.6	148.4	489.5	536.3	89.4	98	1.6	1.6	33.9	24	190.7	189.2	35.8	33.1	31.3	34.1
Both	(1469.1	(1523.3	(0.13	(43.9	(45	(-0.01	(87.6	(90.2	(622	(651.2	(89.9	(90	(310.1	(333.8	(61.3	(67	(0.8	(0.7	(14.6	(9.2	(125.1	(123	(20.8	(19.5	(19.7	(22.2
	,2671.8)	,2807.2)	,0.19)	,93.7)	,92.9)	,0.01)	,167.2)	,171.5)	,1357.3)	,1453.6)	,229.1)	,224.6)	,747.1)	,816.9)	,124.2)	,137.2)	,2.8)	,2.7)	,58.6)	,43.3)	,282.8)	,281.3)	,55.8)	,50.9)	,46.1)	,48.4)
**Oceania**																										
	1533.8	1559.4	0.05	126.8	131	0.06	164.6	162.2	578.8	582.7	71.6	71.5	379.7	402.5	110.6	113	1.1	1.1	8.9	8.9	218.4	217.6	104.7	108.4	22.1	22.6
Male	(1130.4	(1136.6	(0.04	(79.4	(81.6	(0.02	(106.7	(104.8	(385.9	(370.9	(40.1	(38.5	(224.6	(230.2	(74.5	(76.6	(0.4	(0.3	(1.7	(1.8	(140	(141	(61.2	(65.4	(11.8	(11.9
	,1996.5)	,2057.6)	,0.06)	,184.9)	,193.7)	,0.09)	,232.4)	,223.5)	,832.2)	,845.6)	,115.9)	,115.9)	,601.3)	,658.8)	,156)	,158.6)	,2.2)	,2.2)	,19.8)	,19.2)	,328.7)	,321.8)	,159.2)	,166.4)	,36.3)	,37.9)
	1866.5	1903	0.05	58.4	57.2	-0.08	136.8	136.3	804.3	807.6	69.5	68.9	634.7	669.6	49.3	49.8	0.5	0.5	11.9	11.5	159.4	158.7	31.1	30.9	27.3	26.3
Female	(1328.9	(1342.9	(0.03	(35.9	(34.9	(-0.09	(90.9	(88.6	(532.9	(524.3	(39	(38.4	(377.4	(395.1	(32.3	(32.5	(0.1	(0.1	(3.9	(3.9	(101.4	(100.6	(16.2	(16.3	(14.2	(14
	,2497.4)	,2617.7)	,0.06)	,87.8)	,83.6)	,-0.06)	,193.8)	,195.9)	,1151.9)	,1165.2)	,111.4)	,109)	,1005.1)	,1108.4)	,72.1)	,72)	,1.2)	,1.1)	,22.3)	,21.6)	,242.8)	,240.2)	,51.8)	,50.3)	,44)	,43.3)
	1697.6	1725.6	0.05	93.5	95.5	0.03	151.2	149.9	689.4	691.1	70.6	70.3	505	531.5	80.8	82.7	0.8	0.8	10.4	10.2	189.4	189.2	68.8	71.1	24.7	24.4
Both	(1233.2	(1247.9	(0.03	(60.3	(61	(-0.0022	(100.9	(102.2	(457.6	(448.1	(40.4	(39.7	(308.7	(312.8	(54.7	(56.4	(0.3	(0.3	(3.1	(2.9	(121.7	(122.3	(40.7	(43.1	(14.1	(14
	,2232.2)	,2306.1)	,0.06)	,133.1)	,136.5)	,0.05)	,209.1)	,205.4)	,982.2)	,999.5)	,111.7)	,110.5)	,790.2)	,858.2)	,113.8)	,115.7)	,1.6)	,1.6)	,20.8)	,19.9)	,285.7)	,280.8)	,104.3)	,107.6)	,38.5)	,37.3)
**South Asia**																										
	2096.5	2186	0.16	344	304.7	-0.43	169.6	185.7	1087.5	1138.8	92.5	92.9	360.9	388.4	98.6	108.7	0.4	0.4	66.9	49.3	220.3	221.8	317.4	276.7	26.6	28
Male	(1532.7	(1596.1	(0.13	(216.4	(195	(-0.46	(121.4	(132.6	(726.2	(756.2	(58	(57.4	(235.2	(248.7	(68.2	(74.6	(0.1	(0.2	(30.5	(23.1	(142.9	(143.9	(195	(173.5	(16.7	(18.1
	,2698.8)	,2817.1)	,0.18)	,503.2)	,438.8)	,-0.41)	,222.9)	,243.8)	,1513.7)	,1584.2)	,136.3)	,137.7)	,528.8)	,571.7)	,137.2)	,151)	,0.8)	,0.8)	,114.9)	,83.2)	,330)	,330.8)	,472.3)	,407.1)	,39.1)	,40.5)
	2418.1	2491.3	0.12	105.1	103.1	-0.07	137.2	151.4	1446.3	1470.5	87.7	87.9	463.5	504.3	47.8	51.6	0.2	0.2	74.3	63.4	161.2	162	63.6	64.2	41.5	38.9
Female	(1739.1	(1810	(0.06	(69.3	(68.4	(-0.08	(97.7	(108.4	(969.7	(984.6	(55	(54.5	(306.2	(334.2	(32.9	(35.4	(0.1	(0.1	(36	(33.7	(104.3	(105.9	(37.6	(38.3	(25.9	(24.9
	,3139)	,3242.5)	,0.18)	,148.2)	,144.2)	,-0.06)	,179.1)	,196.6)	,2001.5)	,2011.7)	,128.7)	,130)	,676.1)	,737)	,66.4)	,72.1)	,0.4)	,0.4)	,123.1)	,101.2)	,240.2)	,243.8)	,98.8)	,98.6)	,61.4)	,56.6)
	2250.1	2342.8	0.12	230.2	201.3	-0.46	154.2	168.3	1258.5	1308.7	90.2	90.3	410.1	448.3	74.4	79.4	0.3	0.3	70.4	56.6	192	190.9	196.4	167.6	33.8	33.7
Both	(1633.5	(1709.1	(0.08	(147	(130.8	(-0.48	(110.3	(120.6	(843.5	(872.6	(56.8	(56	(270.1	(294.5	(51.8	(54.3	(0.1	(0.1	(33	(28.3	(124.6	(124.3	(120.3	(104.2	(21.4	(21.9
	,2903.9)	,3040.7)	,0.16)	,331.3)	,287)	,-0.44)	,201.2)	,219)	,1742.5)	,1801.1)	,132.5)	,133.4)	,595.6)	,655.4)	,103.9)	,110)	,0.6)	,0.6)	,118.8)	,92.1)	,286)	,286.3)	,293.6)	,247.3)	,49.2)	,48.3)
**Southeast Asia**																										
Male	1494	1575.1	0.17	146.1	140.1	-0.13	162.2	172.4	562.7	590.3	85.2	85.5	333.7	372.6	109.2	122.4	0.9	0.9	17.6	8.5	222.4	222.5	129.8	123.8	16.4	16.3

	(1108	(1168.8	(0.17	(93.4	(90	(-0.14	(112.3	(122.1	(380.8	(398.8	(51.5	(51.4	(213.2	(236.7	(75.2	(83.7	(0.4	(0.4	(5.6	(1.7	(145.3	(144.3	(80.3	(75.6	(9.9	(10
	,1908.5)	,2028.3)	,0.18)	,212.5)	,203.1)	,-0.11)	,215.6)	,227.4)	,786.3)	,830.6)	,128.8)	,130)	,496.1)	,560.1)	,152.4)	,171.5)	,1.7)	,1.7)	,33.9)	,18.1)	,332)	,330.7)	,193.7)	,184.7)	,24.5)	,24.4)
	1700.4	1801	0.19	51.3	50.6	-0.05	136	144	679.5	712.6	83.5	83.4	557.1	627.8	52.8	56.4	0.4	0.4	27.7	13.4	163.4	163	28.4	27.4	22.9	23.2
Female	(1236.4	(1314.5	(0.18	(33.3	(33	(-0.06	(94.5	(99.6	(459.1	(479.5	(50.2	(50.5	(361.1	(406.4	(36	(38.6	(0.2	(0.2	(12.3	(4.9	(105.6	(105.1	(16.5	(16.2	(13.7	(13.8
	,2235.1)	,2380.4)	,0.19)	,72.8)	,71.5)	,-0.04)	,181.1)	,191.6)	,946)	,997.2)	,127.3)	,126.8)	,834)	,944.4)	,75)	,79.6)	,0.8)	,0.9)	,47.9)	,24.9)	,244.2)	,242.4)	,44.9)	,43.1)	,34.7)	,34.7)
	1605.9	1698.9	0.18	95.2	91.2	-0.13	148.3	157.1	625.5	656.7	84.3	84.3	455	512.8	78.9	86.4	0.6	0.7	23	11.2	190.3	189.8	75.1	71	20.1	20.2
Both	(1178.7	(1249.2	(0.18	(61.8	(59.6	(-0.14	(103.3	(111	(425.3	(441.5	(51	(50.9	(294.6	(333.8	(54.4	(59.1	(0.3	(0.3	(9.1	(3.4	(123.3	(123.1	(46.6	(44	(12.3	(12.7
	,2083.4)	,2222)	,0.19)	,136.2)	,129.4)	,-0.12)	,196.5)	,206.7)	,873.5)	,916.9)	,127.6)	,127.8)	,674.3)	,767)	,110.1)	,121.2)	,1.2)	,1.2)	,41.4)	,21.8)	,283.6)	,281.9)	,112.6)	,105.7)	,29.9)	,30)
**Southern Latin America**																										
	1527.1	1532	0.03	325.3	242.4	-0.98	151	151.8	495.9	475.1	151.3	143.7	314	335.6	176	191.7	0.8	0.8	11.3	7.5	226.6	225.9	285.3	200.7	40.1	41.7
Male	(1130.5	(1126.4	(0.01	(207.7	(161.8	(-1.04	(95.8	(98.1	(326.6	(306.7	(87.9	(81.9	(185.7	(188	(120.2	(129.8	(0.2	(0.2	(2.7	(1.1	(143.3	(142.2	(175.6	(130.3	(22.6	(24.9
	,1974.7)	,1962.2)	,0.06)	,473.6)	,339.9)	,-0.92)	,213.1)	,214.3)	,728.9)	,697.4)	,237.4)	,225.8)	,501.3)	,544.7)	,249.7)	,270.8)	,1.6)	,1.6)	,23)	,16.9)	,334.9)	,336.2)	,427.7)	,290)	,62.5)	,63.6)
	2021.6	2063.3	0.09	125.7	118.2	-0.21	163.9	165.6	747.9	699.3	167.8	168.4	693.8	782	76.1	80.2	0.4	0.4	13.7	10.3	157.9	157.2	91.5	85.1	34.2	33
Female	(1463.7	(1458.8	(0.06	(77.5	(74.5	(-0.23	(105.5	(107.7	(489.7	(445.6	(97.2	(97.9	(436.2	(465.6	(50.2	(53.1	(0.1	(0.1	(5.4	(3.3	(100.2	(100	(51.3	(48.9	(18.1	(18.6
	,2674.3)	,2745)	,0.14)	,186.1)	,174.8)	,-0.19)	,229.1)	,230.6)	,1088.6)	,1028.4)	,261.8)	,259.8)	,1058.8)	,1227.8)	,109)	,113.8)	,0.9)	,0.8)	,24.7)	,19.1)	,236.4)	,235.4)	,145.1)	,133.7)	,54.9)	,52.4)
	1805.2	1826.2	0.06	214.1	173.5	-0.70	158.4	159.6	638.2	599.6	160.5	157.3	526.8	582.6	120.3	130.1	0.6	0.6	12.7	9.1	187.8	187.5	177.3	136.6	36.8	36.9
Both	(1322.7	(1313.7	(0.03	(142.4	(118.6	(-0.75	(106.2	(106	(420.6	(387.4	(95.1	(92.2	(332.1	(349.5	(82.6	(88.1	(0.2	(0.2	(4.5	(2.7	(120.1	(120.3	(112.8	(90	(22	(22.9
	,2343.7)	,2393.6)	,0.10)	,303.3)	,240.4)	,-0.65)	,218.2)	,216.8)	,921.3)	,877)	,249.1)	,241)	,809.1)	,912.7)	,169.7)	,182.9)	,1.1)	,1.2)	,23.7)	,17.7)	,278.2)	,277.3)	,257.2)	,195.2)	,55.4)	,54.2)
**Southern Sub-Saharan Africa**																										
	1913.3	2026.6	0.20	427.3	427.3	0.0025	118.3	118.9	1006.8	1092.4	123	121.9	298.7	323.8	140.2	145.7	0.3	0.3	2.1	1.9	223.8	221.8	336.8	347.1	90.5	80.3
Male	(1392.8	(1488.1	(0.18	(279.1	(284.3	(-0.02	(82.6	(84.9	(677	(733	(75.3	(75.5	(190.4	(207.9	(96.3	(99.8	(0.1	(0.1	(0.3	(0.2	(144.1	(144	(211.4	(217.5	(60.7	(55.3
	,2480)	,2626.3)	,0.21)	,595.6)	,593.6)	,0.02)	,157.4)	,157.3)	,1391.5)	,1528.5)	,186.5)	,182.4)	,442.4)	,491)	,196.1)	,203.1)	,0.6)	,0.7)	,6.8)	,6.1)	,333.4)	,333.2)	,491.7)	,499.6)	,124.4)	,108.6)
	2556.8	2638.4	0.10	128.7	120	-0.25	123.2	123.4	1542.3	1564.8	123.7	121.6	530	591.1	68.6	70.4	0.2	0.2	3.6	3.7	165.3	163.2	74.3	67.8	54.4	52.2
Female	(1836.8	(1902.3	(0.06	(86.1	(81.9	(-0.28	(86.9	(87.3	(1036.1	(1047.7	(75.9	(76.2	(348.2	(390.7	(47	(48.1	(0.1	(0.1	(0.7	(0.7	(106.9	(105.7	(45.8	(42.5	(34.6	(34.1
	,3382.8)	,3465.9)	,0.14)	,178.7)	,163.7)	,-0.23)	,162.8)	,162)	,2147.6)	,2188.7)	,184.4)	,181.6)	,770.7)	,867.9)	,96.8)	,98)	,0.3)	,0.3)	,8.6)	,8.8)	,248)	,240.9)	,110.6)	,100.6)	,78.2)	,73.2)
	2291.8	2399.5	0.16	255.8	245.2	-0.14	121.1	121.6	1321.6	1380.5	123.4	121.7	433.8	485.4	99	100.8	0.2	0.2	3	3	189.7	186.4	186.1	181.3	69.7	63.8
Both	(1660.3	(1743.3	(0.13	(170.6	(165	(-0.15	(86	(87.1	(890.3	(926.1	(76.3	(75.8	(283.4	(319.4	(68.3	(68.9	(0.1	(0.1	(0.5	(0.5	(121.8	(121.4	(116.5	(113.9	(46.3	(43.6
	,3004.4)	,3132.4)	,0.20)	,353.4)	,336.9)	,-0.13)	,159.1)	,159.1)	,1835)	,1925.5)	,184)	,180.9)	,634.4)	,718.4)	,138.3)	,140.2)	,0.5)	,0.5)	,7.8)	,7.6)	,283.3)	,277.1)	,271.9)	,261.2)	,97.1)	,87.4)
**Tropical Latin America**																										
	1992.9	2129.2	0.23	391.8	485.5	0.69	146.9	152.2	745.9	765.8	185.8	185.9	589	694.2	93.4	101.4	2	2	7.9	5.6	221.9	222.2	362.8	454.7	29	30.8
Male	(1468	(1550.2	(0.21	(250.2	(306.5	(0.63	(104	(107.9	(502.1	(506.7	(117.2	(118.2	(383.6	(443.4	(64.4	(69	(0.9	(0.9	(2.1	(1	(142.6	(142.9	(226.1	(281.2	(18.8	(20.4
	,2552)	,2753.3)	,0.24)	,563.6)	,695.9)	,0.74)	,191.2)	,198.8)	,1030.4)	,1076.5)	,271)	,273.8)	,860.3)	,1021.8)	,131.6)	,140.8)	,3.7)	,3.6)	,16.3)	,12.2)	,333.4)	,333.8)	,528.5)	,660.5)	,41.6)	,43.2)
	2761.1	2995.4	0.28	117	122.1	0.15	125.2	128.7	1320.3	1288.7	193.4	193.4	900.9	1164	46.1	47.9	0.9	0.9	9.5	7.1	164.7	164.7	89.7	94.6	27.3	27.5
Female	(1999.5	(2134.6	(0.25	(76.3	(80	(0.13	(90	(90.8	(885.2	(844.6	(122.1	(122.3	(607	(780.3	(31.3	(32.4	(0.4	(0.4	(3.4	(2.1	(106.2	(106.3	(55.8	(58.5	(17.1	(17.5
	,3646.5)	,3944.7)	,0.32)	,165.2)	,172.1)	,0.17)	,164.5)	,166.9)	,1836.1)	,1791.6)	,283.6)	,284.4)	,1280.4)	,1663.2)	,64.6)	,67.5)	,1.8)	,1.7)	,17.2)	,13.5)	,247.6)	,246.8)	,133.5)	,140.6)	,39.5)	,39.8)
	2406.1	2608.4	0.28	243.3	284.4	0.55	135.4	139.4	1053.6	1054.4	189.9	190	758	954.8	68.1	71.9	1.4	1.4	8.8	6.4	190.9	190.2	215.3	255.4	28.1	29
Both	(1747.2	(1875.9	(0.26	(156.2	(181.7	(0.50	(97.2	(100.1	(709	(694.6	(120.6	(119.8	(511	(638.7	(46.9	(48.9	(0.6	(0.6	(2.8	(1.7	(122.8	(122.3	(134.2	(158.7	(18.3	(19.1
	,3142.9)	,3405.6)	,0.31)	,347.7)	,406.9)	,0.60)	,176.1)	,179.8)	,1466.1)	,1460.9)	,276.7)	,279)	,1084.3)	,1368.7)	,95.5)	,100.1)	,2.7)	,2.6)	,16.8)	,12.9)	,287)	,284.8)	,314.4)	,371.9)	,39.9)	,40.6)
**Western Europe**																										
Male	1891.8	1971.9	0.15	257.9	295.8	0.46	163.3	163.3	718.2	726.8	189.9	193	419.3	477	162.8	177.4	0.8	0.8	11.3	6.9	226.2	226.7	222.6	253.4	35.2	42.4

	(1398.3	(1446.2	(0.14	(175.1	(205.8	(0.43	(115.4	(117.5	(487.8	(488.6	(116.7	(118.1	(259.3	(295.8	(111.5	(121.5	(0.3	(0.3	(3.7	(1.6	(146.1	(145.6	(146.7	(171.8	(22.7	(28.8
	,2439.8)	,2550.2)	,0.17)	,352.2)	,403.8)	,0.49)	,210.2)	,212.3)	,987.9)	,1023.2)	,286)	,288.6)	,640)	,738.2)	,225.1)	,247.7)	,1.5)	,1.6)	,21.8)	,14.9)	,333.2)	,334.3)	,309.9)	,355.4)	,49)	,57.6)
	2520.6	2690.4	0.25	98.8	122.9	0.70	181.1	180	1071.4	1106.5	220.8	225.9	814.5	946.4	56.8	60.4	0.3	0.4	16.5	12	159.1	158.9	65.4	72.4	33.4	50.5
Female	(1827.6	(1943.6	(0.20	(66.7	(86	(0.68	(129.1	(127.3	(730.9	(739	(135.3	(138.9	(532.3	(620.8	(38.8	(40.9	(0.1	(0.1	(7.8	(4.9	(103.7	(103.4	(40.5	(47.4	(21.9	(34
	,3300.1)	,3550.9)	,0.29)	,136.2)	,165.2)	,0.71)	,235.3)	,232.6)	,1464.2)	,1579.7)	,327.3)	,336.3)	,1207.6)	,1409.8)	,79.3)	,84.6)	,0.7)	,0.7)	,27.2)	,20.6)	,237.4)	,238.2)	,97)	,105)	,47.7)	,68.3)
	2258.7	2356.2	0.17	167.7	204.9	0.66	173.8	172.3	924.6	930	207.4	210.3	648.8	727.7	102.1	115.3	0.6	0.6	14.3	9.6	187.1	190.4	133.1	157.8	34.6	47.1
Both	(1645.8	(1714.2	(0.13	(113.9	(143.2	(0.64	(124.3	(123.7	(631.2	(621.6	(128	(129.6	(425.1	(472.9	(70.2	(79.1	(0.2	(0.3	(6.2	(3.5	(121.7	(123.4	(87.1	(106.9	(22.9	(32.3
	,2943.7)	,3083.1)	,0.19)	,228.3)	,276.9)	,0.68)	,223.2)	,221.7)	,1267.3)	,1318.5)	,307.8)	,313.2)	,964)	,1089.6)	,141.9)	,161.1)	,1.1)	,1.1)	,24.8)	,17.9)	,277.7)	,282)	,187.4)	,222)	,48.1)	,63.1)
**Western Sub-Saharan Africa**																										
	2109.1	2170.9	0.10	105.3	107.8	0.08	110.5	114.9	1278.7	1312.9	114.9	115.7	249	259.3	131.2	141.1	0.3	0.3	3.2	3.5	221.3	223	82.7	83.2	22.6	24.6
Male	(1505.4	(1543.9	(0.08	(68.7	(71.8	(0.07	(77	(79.9	(849.1	(875.1	(70.2	(71	(158.3	(162.9	(90.3	(96.4	(0.1	(0.1	(0.8	(0.6	(144.9	(145.4	(51.7	(52.7	(14.7	(16.3
	,2746.5)	,2832.8)	,0.12)	,148.9)	,150.6)	,0.09)	,145.9)	,151.4)	,1777.8)	,1838.3)	,172.6)	,174.2)	,373.4)	,393.6)	,182.4)	,196.2)	,0.7)	,0.7)	,8.8)	,10)	,329)	,330.9)	,121.8)	,121.9)	,32.1)	,34.2)
	2622.9	2629	0.02	91.2	98.4	0.23	131.1	135.6	1779.1	1756.2	113.7	114.3	368.5	387.3	64.6	68.7	0.2	0.2	3.7	4.4	162	162.2	73.5	80.1	17.7	18.2
Female	(1851.4	(1854.6	(-0.01	(59.7	(65.6	(0.20	(92.8	(95.9	(1175.7	(1158.9	(70.3	(69.8	(238.6	(245.9	(44.6	(47.3	(0.1	(0.1	(1.2	(1	(105.2	(104.9	(46.3	(50.9	(10.8	(11.2
	,3479.2)	,3478.1)	,0.05)	,130.1)	,140.2)	,0.25)	,172.2)	,178.9)	,2482.3)	,2446.6)	,170.1)	,172.1)	,551.4)	,589.1)	,90.2)	,95.5)	,0.4)	,0.4)	,9.3)	,10.7)	,241.4)	,242.4)	,110.1)	,119)	,27.1)	,27.5)
	2370	2413.6	0.07	99.7	102.8	0.10	120.5	125.9	1531.6	1547.8	114.3	114.9	310.2	327.2	98.2	102.8	0.3	0.3	3.5	4	191.4	190.8	79.7	81.6	20	21.2
Both	(1681.8	(1709.5	(0.04	(65.4	(68.8	(0.08	(85	(88.6	(1017.9	(1026.1	(70.4	(70.6	(199.5	(207.5	(67.7	(70.4	(0.1	(0.1	(1	(0.8	(124.7	(124.1	(50.4	(52.5	(12.9	(13.9
	,3110.5)	,3169.5)	,0.08)	,140.9)	,144)	,0.11)	,157.8)	,165.8)	,2129.7)	,2156.6)	,170.7)	,173.2)	,462.3)	,497.1)	,136.5)	,143.3)	,0.5)	,0.5)	,9)	,10.3)	,284.4)	,282.5)	,117.7)	,118.8)	,28.9)	,30.2)

Note: YLDS-years lived with disability; AAPC-average annual percentage changes; 95% CI-95% confidence intervals(AAPC); 95% UI-95% uncertainty interval

eTable 7. Age Standardised Prevalence and EAPC of Mental disorders, Substance use disorders in people aged ≥60 years in 204 countries and territories, from 1990 to 2021

Location	Rate (95% UI/95%CI)																									
	Mental disorders		Schizophrenia		Depressive disorders		Bipolar disorder		Anxiety disorders		Autism spectrum disorders		Attention-deficit/ hyperactivity disorder		Idiopathic developmental intellectual disability		Other mental disorders		Substance use disorders		Alcohol use disorders		Drug use disorders	
	2021	EAPC,%	2021	EAPC,%	2021	EAPC,%	2021	EAPC,%	2021	EAPC,%	2021	EAPC,%	2021	EAPC,%	2021	EAPC,%	2021	EAPC,%	2021	EAPC,%	2021	EAPC,%	2021	EAPC,%
	15752.1	0.12	184.9	0	6726.2	0.19	730.6	0.03	4918.5	0.36	436	-0.06	93.2	-0.31	972.5	-0.45	2681.2	-0.11	525.3	-0.27	384.6	-0.52	141.2	0.52
**Afghanistan**	(13228.9	(0.09	(142.7	(-0.06	(4947.4	(0.16	(536.5	(0.03	(3204.7	(0.29	(353.9	(-0.09	(51.6	(-0.33	(462.2	(-0.8	(2144.3	(-0.11	(395.6	(-0.3	(258.4	(-0.56	(111.9	(0.46
	,18736.9)	,0.15)	,233.9)	,0.05)	,9033.1)	,0.22)	,988.9)	,0.03)	,7375.5)	,0.44)	,533.1)	,-0.04)	,147.8)	,-0.29)	,1468.5)	,-0.09)	,3334.7)	,-0.1)	,685.9)	,-0.24)	,544.5)	,-0.49)	,174.1)	,0.59)
	13558.9	0.05	214.5	0.22	4696.1	0.08	552.2		5119.1	0.13	721	0.42	63.9	-0.04	206.4	-2.08	2717.7	0.02	2117.7	-0.1	1971.6	-0.18	149.1	1.32
**Albania**	(11343.3	(0.01	(169.8	(0.2	(3526.1	(0.02	(402.5	0 (0,0)	(3277.9	(0.04	(586.3	(0.39	(31	(-0.05	(30.3	(-2.26	(2171.4	(0.02	(1466.2	(-0.28	(1324.2	(-0.36	(122	(1.11
	,16309.3)	,0.1)	,270.4)	,0.24)	,6168.2)	,0.13)	,733.2)		,7745.1)	,0.23)	,861.4)	,0.46)	,106)	,-0.03)	,395.2)	,-1.91)	,3385)	,0.03)	,3012)	,0.08)	,2869.6)	,0)	,179.7)	,1.53)
	14519.3	-0.02	224.2	0.08	5935.6	-0.04	743.4	-0.01	4479.1	0.05	575.8	0.34	100.3	0.07	557.5	-0.94	2750.3	0.02	470.4	-0.18	359	-0.34	111.9	0.36
**Algeria**	(12270.2	(-0.05	(177.2	(0.06	(4355.3	(-0.08	(548.3	(-0.01	(2772.8	(0	(469.6	(0.33	(55.5	(0.06	(243.4	(-1.1	(2197.6	(0.02	(340.6	(-0.2	(232.9	(-0.37	(87.9	(0.33
	,17426)	,0.01)	,280.4)	,0.09)	,8016.9)	,0)	,996.3)	,-0.01)	,6716.4)	,0.1)	,694.5)	,0.35)	,158.3)	,0.08)	,874.3)	,-0.78)	,3425.9)	,0.03)	,633.4)	,-0.16)	,521.1)	,-0.31)	,138.7)	,0.39)
**American Samoa**	12823.9	0.02	277.1	-0.2	4459.5	-0.01	356.9	0 (0,0)	4930.6	0.05	518.1	0.09	69.4	-0.02	110.2	0.97	2732.4	0.01	891.4	-0.38	748.1	-0.47	144.5	0.11
	(10662.3	(0	(220.4	(-0.21	(3420	(-0.03	(254.8		(3128.3	(0.01	(419.1	(0.07	(34	(-0.04	(20.5	(0.9	(2183.2	(0.01	(659.9	(-0.41	(522.7	(-0.5	(110.8	(0.09

	,15374.8)	,0.04)	,344.3)	,-0.18)	,5839.1)	,0.01)	,483.8)		,7493.7)	,0.08)	,624.8)	,0.1)	,116.1)	,0)	,245.1)	,1.03)	,3403.9)	,0.01)	,1177.6)	,-0.35)	,1033)	,-0.44)	,183.6)	,0.12)
	15113	0.03	304.7	-0.04	5136.8	-0.07	1049	0.08	5907.1	0.13	693.1	0.14	59.9	0.01	109.3	-0.64	2763	-0.01	1551.1	-0.24	1391.2	-0.28	162.6	0.17
**Andorra**	(12519.4	(-0.01	(241.8	(-0.04	(3796.5	(-0.14	(776.4	(0.07	(3796.8	(0.07	(566.2	(0.11	(27.3	(-0.01	(19.2	(-0.79	(2229.8	(-0.01	(1115.8	(-0.32	(961.6	(-0.38	(130.8	(0.11
	,18057)	,0.07)	,378)	,-0.03)	,6794.8)	,-0.01)	,1404.7)	,0.1)	,8722.8)	,0.2)	,834.6)	,0.17)	,102.7)	,0.03)	,236.8)	,-0.48)	,3407.7)	,0)	,2076.9)	,-0.15)	,1921.3)	,-0.19)	,202.1)	,0.23)
	17594.4	0.03	204.5	0.26	10532.4	0.01	602		3977.5	0.16	564.3	0.17	22.3	-0.11	48.2	-0.74	2677.8	-0.05	1673.3	-0.06	1574.2	-0.07	101.1	0.17
**Angola**	(14480.5	(0	(161.2	(0.22	(7733.7	(-0.02	(436.1	0 (0,0)	(2538	(0.11	(456.4	(0.15	(9	(-0.11	(10.9	(-1.02	(2139.4	(-0.05	(1237.8	(-0.14	(1135.5	(-0.16	(79.4	(0.15
	,21701.2)	,0.06)	,258.1)	,0.3)	,14424)	,0.04)	,811.8)		,6140.4)	,0.21)	,681.2)	,0.2)	,41.2)	,-0.1)	,157.1)	,-0.46)	,3333.8)	,-0.05)	,2176.4)	,0.02)	,2077.9)	,0.01)	,126.3)	,0.18)
	13983.5	0.05	248	0.1	5103.2	0.03	825.8		5050.1	0.07	510.9	0.23	229	0.11	84.5	-0.64	2709.5	0.04	3386.6	0.18	3241.7	0.19	150.8	0.01
**Antigua and Barbuda**	(11672	(-0.01	(194.2	(0.09	(3761.5	(-0.07	(603.8	0 (0,0)	(3137.6	(0	(412.5	(0.22	(130	(0.1	(11	(-0.73	(2164.6	(0.04	(2494.6	(0.04	(2346.7	(0.04	(116.3	(-0.04
	,16753.6)	,0.1)	,314)	,0.11)	,6855.5)	,0.13)	,1099.3)		,7570.4)	,0.14)	,614)	,0.24)	,356.9)	,0.12)	,190.9)	,-0.54)	,3374.4)	,0.04)	,4503.5)	,0.31)	,4358.7)	,0.33)	,194)	,0.06)
	12537.3	-0.11	266.5	0.01	3311.1	-0.54	786.6	-0.06	5137.6	0.07	729.4	0.2	48.8	0	185.5	-0.7	2692.7	0.01	1608.6	-1.24	1382.5	-1.43	229.9	0.14
**Argentina**	(10638.2	(-0.16	(211.7	(0	(2533.2	(-0.63	(579.3	(-0.07	(3253.2	(-0.02	(596.2	(0.19	(22.3	(-0.01	(38.6	(-0.81	(2171.1	(0.01	(1304	(-1.39	(1078.1	(-1.6	(189.7	(0.06
	,15206.3)	,-0.05)	,335.3)	,0.03)	,4279.5)	,-0.45)	,1045.3)	,-0.05)	,7809.3)	,0.16)	,878.7)	,0.21)	,86.6)	,0)	,357.8)	,-0.58)	,3322.8)	,0.01)	,1998.3)	,-1.1)	,1770.8)	,-1.26)	,270.5)	,0.22)
	14156.6	0.13	204.7	0.2	6918.6	0.28	560.1		3625.8	0.1	663.7	0.27	61.6	-0.03	206.4	-1.84	2666.6	0.02	2922.1	-0.89	2785.2	-0.91	140.7	-0.34
**Armenia**	(11951.5	(0.08	(161.7	(0.16	(5126.4	(0.22	(405	0 (0,0)	(2259.1	(0.01	(537.4	(0.26	(29.8	(-0.04	(36.7	(-2.24	(2130.4	(0.01	(2083.6	(-1.07	(1941.1	(-1.1	(107.9	(-0.4
	,16827.5)	,0.17)	,255.9)	,0.25)	,9152.4)	,0.33)	,755.8)		,5480.8)	,0.2)	,797.6)	,0.27)	,102.1)	,-0.01)	,410.8)	,-1.43)	,3319.5)	,0.02)	,3839.5)	,-0.7)	,3699.7)	,-0.72)	,179.8)	,-0.27)
	13617.6	0.06	355	0.03	4317.3	-0.02	993.7	0.01	4411.8	0.13	967	0.3	179.5	0.01	116.9	1.81	3048.3	0.02	1703.8	0.23	1458.6	0.23	249.2	0.2
**Australia**	(11627.2	(0.03	(303.1	(0.02	(3176.8	(-0.18	(769.7	(0	(2725.5	(-0.12	(790.8	(0.27	(97.1	(-0.01	(20.8	(1.13	(2564.2	(0.02	(1321.7	(0.19	(1078.8	(0.19	(204.7	(0.16
	,16197.1)	,0.1)	,409.2)	,0.03)	,5728.2)	,0.14)	,1260.1)	,0.01)	,6779.4)	,0.38)	,1166)	,0.33)	,288.1)	,0.02)	,247.5)	,2.51)	,3649.5)	,0.02)	,2131.1)	,0.26)	,1890.1)	,0.27)	,296.4)	,0.24)
	14879.9	-0.08	298.7	-0.02	4299.6	-0.38	1052.3	0.06	6537.1	-0.01	645.8	0.43	70.9	0.11	134.9	-1.37	2709.8	0.09	2951.4	1.76	2787.1	1.85	170	0.52
**Austria**	(12462.6	(-0.13	(237.1	(-0.03	(3263.3	(-0.45	(780.5	(0.04	(4152.7	(-0.09	(526.3	(0.39	(34.5	(0.09	(25.4	(-1.45	(2185.5	(0.08	(2273.5	(1.6	(2106.2	(1.68	(138	(0.42
	,17761)	,-0.04)	,375.3)	,-0.01)	,5661)	,-0.31)	,1405.5)	,0.07)	,9605.3)	,0.07)	,777.9)	,0.48)	,121.3)	,0.13)	,277.4)	,-1.29)	,3344.1)	,0.1)	,3746.4)	,1.91)	,3580.3)	,2.01)	,206.2)	,0.62)
	12947.1	0.02	209.4	0.26	6201.5	0.03	551.4	-0.01	3095.6	-0.01	623.5	0.35	62.3	0.03	157.1	-1.65	2674	0.06	2882.9	-0.29	2753.6	-0.29	133	-0.21
**Azerbaijan**	(11004.2	(-0.03	(165.9	(0.17	(4622.5	(-0.04	(398	(-0.01	(1937.6	(-0.09	(506.6	(0.33	(30.2	(0.01	(27	(-2.37	(2136.1	(0.05	(2025.2	(-0.49	(1890.9	(-0.51	(103.7	(-0.25
	,15318.7)	,0.06)	,263.2)	,0.35)	,8258.3)	,0.1)	,739.5)	,0)	,4755.3)	,0.08)	,746)	,0.36)	,103.3)	,0.04)	,319.3)	,-0.92)	,3329.3)	,0.06)	,4044.9)	,-0.08)	,3911.2)	,-0.07)	,168.3)	,-0.18)
	14108.2	0.06	246.7	0.02	5194.4	0.03	817		5179	0.13	476.1	0.18	223.4	0.02	72.2	0.15	2685.9	0.03	2463.5	-0.1	2313.8	-0.11	154.1	-0.04
**Bahamas**	(11881.5	(0.01	(193.8	(0	(3831	(-0.06	(599.7	0 (0,0)	(3318.9	(0.04	(386.1	(0.15	(126.8	(0.01	(9.4	(-0.03	(2146	(0.02	(1851.6	(-0.18	(1701.2	(-0.19	(116.5	(-0.09
	,17010.3)	,0.11)	,311.3)	,0.03)	,7060.6)	,0.12)	,1100.1)		,7944.8)	,0.21)	,574.7)	,0.2)	,348.1)	,0.03)	,166.9)	,0.32)	,3343.9)	,0.03)	,3180.1)	,-0.02)	,3033.8)	,-0.02)	,201.9)	,0)
	15373.4	-0.11	244	0.11	6743.8	-0.29	741.1	-0.01	4746.9	-0.01	629.9	0.42	106.2	0.17	313	-0.58	2774.3	0.04	508.4	-0.71	412.7	-0.85	96.1	0
**Bahrain**	(12887.9	(-0.15	(192.2	(0.1	(5012.2	(-0.36	(544.6	(-0.02	(3082.3	(-0.06	(513.9	(0.39	(58.8	(0.12	(69.5	(-0.65	(2218.1	(0.03	(372.6	(-0.75	(280.9	(-0.9	(71.6	(-0.07
	,18517)	,-0.07)	,305.9)	,0.11)	,9157.7)	,-0.22)	,985)	,-0.01)	,7226)	,0.05)	,761.2)	,0.45)	,167.6)	,0.21)	,545.4)	,-0.51)	,3457.7)	,0.05)	,672.5)	,-0.66)	,579.1)	,-0.81)	,126.3)	,0.06)
	16528	0.15	281.9	0.08	9184.6	0.29	477.5		4213.4	0.25	413.4	0.28	45.7	-0.08	110.2	-4.14	2755.7	-0.04	1476.6	-0.38	1370.3	-0.42	107.7	0.18
**Bangladesh**	(13771.6	(0.11	(219.9	(0.07	(6908	(0.23	(344.3	0 (0,0)	(2678	(0.17	(333.2	(0.26	(21.4	(-0.09	(14.4	(-4.34	(2202.5	(-0.04	(996	(-0.42	(887.6	(-0.46	(82.1	(0.13
	,19988.2)	,0.19)	,356.3)	,0.1)	,12389.4)	,0.35)	,645.8)		,6455.4)	,0.32)	,499.6)	,0.3)	,76.9)	,-0.07)	,260.4)	,-3.95)	,3431.9)	,-0.04)	,2066.4)	,-0.34)	,1965.6)	,-0.38)	,137.5)	,0.23)
	14415.4	0.08	260.4	-0.02	5467.4	0.13	825.2		5152.7	0.07	505.4	0.22	224.4	0.12	107.6	0.47	2694.5	0.04	1875.7	0.07	1727.2	0.08	151.7	-0.03
**Barbados**	(12040.6	(0.04	(204.6	(-0.05	(4035.3	(0.07	(606.8	0 (0,0)	(3325.1	(-0.01	(409.6	(0.21	(127.4	(0.11	(12.8	(0.29	(2152.9	(0.04	(1417.4	(-0.01	(1264.4	(-0.01	(110.3	(-0.07
	,17368.7)	,0.13)	,329.3)	,0.01)	,7344)	,0.19)	,1106.8)		,7935.4)	,0.14)	,611.5)	,0.22)	,349.9)	,0.13)	,229.4)	,0.65)	,3354.7)	,0.05)	,2481.1)	,0.15)	,2333.5)	,0.17)	,201.1)	,0.01)
	16759.5	-0.02	199.4	0.19	8329.3	-0.09	556.6		5226.3	0.08	673.5	0.34	60.3	0.05	134.3	-1.51	2621.3	0.02	6187	1.52	5959.1	1.56	242.7	0.62
**Belarus**	(14080.7	(-0.08	(155.7	(0.15	(6137.7	(-0.16	(404.9	0 (0,0)	(3301.9	(-0.04	(552.2	(0.32	(29.2	(0.04	(17.3	(-1.9	(2094.7	(0.02	(4543.2	(1.31	(4318.3	(1.34	(204.4	(0.41
	,20098.7)	,0.04)	,250.4)	,0.23)	,11374.2)	,-0.02)	,750.8)		,7894.2)	,0.2)	,813.6)	,0.37)	,100)	,0.06)	,289.8)	,-1.13)	,3262.7)	,0.02)	,7999.6)	,1.74)	,7775.4)	,1.79)	,283)	,0.83)

	14442.4	0.13	295.7	-0.01	4807.4	0.26	1045.7	0.06	5553.8	0.11	646.4	0.31	58.2	0.07	148	-1.19	2718.1	0.05	2434.4	0.98	2216.2	0.99	223.3	0.97
**Belgium**	(12167.3	(0.06	(233.6	(-0.02	(3508.5	(0.13	(778.4	(0.05	(3618.3	(0.01	(524.3	(0.3	(26.5	(0.06	(31.1	(-1.28	(2192.5	(0.05	(1913.9	(0.89	(1694	(0.88	(187	(0.93
	,17308.7)	,0.2)	,371.1)	,0)	,6425.6)	,0.38)	,1399.3)	,0.07)	,8635.1)	,0.21)	,776.8)	,0.33)	,99.8)	,0.07)	,289.5)	,-1.1)	,3354.3)	,0.06)	,3184.9)	,1.08)	,2967.4)	,1.09)	,265.2)	,1)
	14171	0.09	230.8	0.08	5491	0.14	816.2		4812.1	0.12	484.4	0.17	234.5	-0.01	152.1	-0.57	2744.4	0.02	2201.2	0.16	2054.7	0.18	150.4	-0.08
**Belize**	(11962.6	(0.05	(180.6	(0.07	(4066	(0.08	(600.6	0 (0,0)	(3074.7	(0.05	(390	(0.15	(133.3	(-0.03	(25.5	(-0.66	(2193.4	(0.01	(1634.3	(0.07	(1488.9	(0.08	(113.7	(-0.13
	,16792.6)	,0.13)	,291.1)	,0.1)	,7271.3)	,0.2)	,1091.9)		,7271.2)	,0.2)	,588.3)	,0.19)	,365.6)	,0.02)	,303.7)	,-0.47)	,3418.1)	,0.02)	,2915.5)	,0.25)	,2774.1)	,0.28)	,195.9)	,-0.04)
	16425.1	0.16	203.7	0.13	10081.2	0.23	569.1		3067.1	0.14	583.6	0.14	23.1	0.02	75.2	0.84	2698.6	-0.03	977.1	0.15	882.6	0.16	95.3	0.11
**Benin**	(13552.3	(0.14	(158.9	(0.12	(7333.6	(0.21	(413	0 (0,0)	(1904.8	(0.11	(471.1	(0.13	(9.3	(0.01	(18.7	(0.65	(2155.9	(-0.03	(718.6	(0.08	(625.7	(0.07	(75	(0.09
	,19947.9)	,0.17)	,259.4)	,0.14)	,13523.8)	,0.25)	,770.9)		,4730.5)	,0.17)	,701.4)	,0.16)	,43.4)	,0.04)	,231.4)	,1.02)	,3361)	,-0.02)	,1300.1)	,0.22)	,1208.1)	,0.24)	,119)	,0.13)
	14498.7	-0.05	266.1	0.14	5630.6	-0.26	839.9		5071.2	0.1	559.5	0.32	225.3	0.03	44.5	-1.51	2689.6	0.03	1762.9	-0.31	1596	-0.34	170.1	-0.03
**Bermuda**	(12276.3	(-0.09	(208.5	(0.13	(4109.1	(-0.32	(616.5	0 (0,0)	(3244.8	(0.04	(454.4	(0.3	(128.1	(0.02	(5.4	(-1.65	(2148.7	(0.02	(1349.3	(-0.42	(1181.2	(-0.46	(132.5	(-0.13
	,17337.5)	,-0.01)	,336.2)	,0.16)	,7683.3)	,-0.2)	,1123.2)		,7622.4)	,0.16)	,678.9)	,0.35)	,351)	,0.03)	,120)	,-1.37)	,3349)	,0.03)	,2326.4)	,-0.2)	,2160.9)	,-0.21)	,215.3)	,0.07)
	16021.9	-0.11	295.6	0.21	8417.6	-0.15	477.4		4064	0.05	441	0.36	35.9	-0.02	490.5	-1.84	2738.1	0.02	3385.8	0.24	3274.8	0.24	114.9	0.16
**Bhutan**	(13326.6	(-0.14	(230.2	(0.2	(6192.7	(-0.21	(345.8	0 (0,0)	(2615.4	(0.03	(358.1	(0.35	(15.7	(-0.03	(202.7	(-1.87	(2187.9	(0.01	(2360.8	(0.18	(2250	(0.18	(87.3	(0.13
	,19214.3)	,-0.08)	,375)	,0.22)	,11315.8)	,-0.1)	,644.7)		,6149.8)	,0.07)	,530.9)	,0.38)	,64.2)	,-0.01)	,779.8)	,-1.8)	,3410.9)	,0.03)	,4696.2)	,0.29)	,4594.3)	,0.3)	,148.4)	,0.18)
	16935.4	0.04	230.9	0.13	6825.6	-0.07	811.5		6704.7	0.22	439.7	0.32	155.1	0.02	185.1	-1.53	2705.5	0.01	2230.2	-0.38	2080.8	-0.42	153.2	0.25
**Bolivia (Plurinational State of)**	(14085.5	(-0.04	(180.6	(0.12	(4925.6	(-0.15	(594	0 (0,0)	(4242.2	(0.08	(357.6	(0.31	(85.2	(0.01	(35.8	(-1.59	(2161.5	(0.01	(1651	(-0.54	(1498.1	(-0.59	(123.8	(0.24
	,20679.6)	,0.11)	,291.2)	,0.14)	,9210.7)	,0.01)	,1088.7)		,10129)	,0.36)	,534.4)	,0.33)	,250.2)	,0.02)	,337.4)	,-1.46)	,3369.1)	,0.01)	,3008.1)	,-0.21)	,2859.3)	,-0.24)	,186.4)	,0.27)
	14590.5	-0.2	212.2	0.36	5976	-0.44	548.7	-0.01	5026.7	0.03	722.5	0.4	62.7	0.05	194.7	-3.41	2683.8	0.03	2831.7	-1.99	2750.7	-2.04	83.3	0.26
**Bosnia and Herzegovina**	(12309.2	(-0.26	(167.2	(0.32	(4340	(-0.53	(398.3	(-0.01	(3174.6	(-0.05	(588	(0.36	(30.4	(0.04	(24.6	(-3.73	(2143.8	(0.03	(2047.8	(-2.14	(1967	(-2.19	(63.3	(0.24
	,17612.9)	,-0.14)	,266.1)	,0.4)	,8111.8)	,-0.34)	,733.4)	,-0.01)	,7656.3)	,0.12)	,869.1)	,0.44)	,104)	,0.07)	,383.4)	,-3.1)	,3342)	,0.03)	,3815.7)	,-1.84)	,3742.1)	,-1.89)	,107)	,0.29)
	16051.2	0.11	217.4	0.27	8676.1	0.15	600.4		4165.7	0.17	585.9	0.19	22.1	-0.03	41.5	-1.33	2657.4	-0.02	2436.4	-0.12	2271.3	-0.16	170	0.41
**Botswana**	(13375.5	(0.08	(171.5	(0.26	(6229.1	(0.09	(434.1	0 (0,0)	(2614.2	(0.11	(473.4	(0.13	(9	(-0.03	(8	(-1.71	(2123.5	(-0.03	(1764.8	(-0.22	(1603.9	(-0.26	(140.5	(0.39
	,19416.7)	,0.15)	,272.3)	,0.29)	,11716.1)	,0.2)	,807.7)		,6255.3)	,0.23)	,706.9)	,0.24)	,40.9)	,-0.02)	,125.2)	,-0.95)	,3307.4)	,-0.01)	,3231.7)	,-0.03)	,3071.3)	,-0.06)	,203.1)	,0.43)
	18469.8	0.18	239.2	0.13	6336.7	-0.22	962.8		8792	0.64	417.1	0.18	124.3	-0.03	133.7	-0.83	2782.6	-0.01	2975.3	-0.03	2758.3	-0.05	224.5	0.15
**Brazil**	(16345.6	(0.05	(199.7	(0.12	(5162.6	(-0.3	(768.8	0 (0,0)	(6835.6	(0.35	(340.3	(0.18	(65	(-0.04	(25.2	(-0.89	(2225.7	(-0.01	(2254.4	(-0.24	(2020.8	(-0.27	(183.4	(0.09
	,20964.4)	,0.31)	,284)	,0.15)	,7725.6)	,-0.14)	,1192.9)		,11267.4)	,0.93)	,499.7)	,0.19)	,202.5)	,-0.03)	,259.6)	,-0.78)	,3479.6)	,-0.01)	,3863.5)	,0.18)	,3651)	,0.18)	,272.4)	,0.21)
	9520	0.04	254.6	-0.06	2365.8	0.1	612.6	0.01	2894.5	0.09	971.2	0.09	53.4	-0.06	5.5	-4.91	2725.4	-0.04	1024.1	-0.89	831.4	-0.65	194.6	-1.82
**Brunei Darussalam**	(8204.7	(0.02	(203.1	(-0.08	(1770.5	(0.05	(445.8	(0.01	(1797.2	(0.05	(792.7	(0.07	(24.8	(-0.08	(0.1	(-5.56	(2197.9	(-0.04	(757.8	(-0.94	(568.5	(-0.69	(159.1	(-2.02
	,11249.8)	,0.06)	,317.9)	,-0.05)	,3115.1)	,0.16)	,821.5)	,0.02)	,4367.3)	,0.13)	,1169.1)	,0.12)	,93.1)	,-0.04)	,39.6)	,-4.26)	,3363.4)	,-0.03)	,1382.2)	,-0.84)	,1195.9)	,-0.61)	,234.8)	,-1.63)
	15041.3	-0.12	219	0.16	6309.7	-0.38	475.9		5357.3	0.14	675.6	0.1	62.4	-0.03	149.5	-1.11	2669.6	-0.03	2178.1	-0.99	2048.9	-1.05	132.2	0.23
**Bulgaria**	(12742.4	(-0.19	(170.5	(0.12	(4651.9	(-0.49	(346.1	0 (0,0)	(3434.8	(0.02	(552	(0.07	(30.3	(-0.05	(24.5	(-1.37	(2132.2	(-0.04	(1518.5	(-1.1	(1387.7	(-1.17	(105.4	(0.21
	,18105.8)	,-0.05)	,275.3)	,0.19)	,8311.6)	,-0.28)	,637.9)		,8305.7)	,0.26)	,809.9)	,0.13)	,103.5)	,-0.02)	,306)	,-0.85)	,3324.1)	,-0.03)	,3032.5)	,-0.88)	,2900.3)	,-0.93)	,162.4)	,0.25)
	16399.8	0.04	197	0.15	10111.3	0.02	565.5		2961.2	0.07	572.9	0.11	23.2	-0.03	130.3	1.55	2707.7	-0.02	1087.2	0.04	984.4	0.02	103.8	0.27
**Burkina Faso**	(13528.5	(0	(152.6	(0.14	(7417.3	(-0.04	(409.9	0 (0,0)	(1864.6	(0.05	(463.4	(0.09	(9.3	(-0.03	(22.1	(1.35	(2163.3	(-0.02	(812.2	(-0.05	(711.2	(-0.08	(84.5	(0.25
	,19866)	,0.07)	,250.8)	,0.16)	,13458.9)	,0.08)	,765)		,4552.9)	,0.1)	,689.7)	,0.12)	,43.5)	,-0.02)	,324.8)	,1.75)	,3372)	,-0.01)	,1408.5)	,0.13)	,1308)	,0.12)	,125.9)	,0.29)
	17464.3	-0.29	175.3	-0.08	10343.6	-0.6	645	-0.01	3758.6	0.04	580.6	0.38	23.6	0.21	226.8	1.86	2750.6	0.08	2307.1	0.06	2209.9	0.05	99.7	0.18
**Burundi**	(14575.7	(-0.35	(133.6	(-0.1	(7582.1	(-0.67	(469.6	(-0.01	(2439.5	(-0.02	(464.1	(0.36	(9.5	(0.2	(40.7	(1.77	(2198.2	(0.07	(1707.9	(0	(1610.7	(0	(77	(0.16
	,20919.8)	,-0.24)	,226.3)	,-0.07)	,13789.3)	,-0.52)	,864.5)	,-0.01)	,5710.6)	,0.1)	,698.5)	,0.41)	,43)	,0.23)	,492.8)	,1.96)	,3427.7)	,0.08)	,3108.9)	,0.11)	,3010.5)	,0.11)	,126.1)	,0.2)
**Cabo Verde**	17526.6	0.22	224.4	0.27	11129	0.33	578.7	0 (0,0)	3180.7	0.15	646.9	0.24	22.5	0.01	64.5	-0.95	2655	-0.03	905.5	0.47	802.1	0.46	104.3	0.54
	(14362.3	(0.17	(177.3	(0.25	(8161	(0.26	(421.4		(1960.6	(0.09	(522.7	(0.2	(9.1	(-0.03	(14.9	(-1.12	(2121.1	(-0.05	(657.3	(0.39	(555.4	(0.37	(84.2	(0.51

	,21325.8)	,0.26)	,281.5)	,0.29)	,15065.2)	,0.41)	,779.7)		,4946.9)	,0.21)	,779.6)	,0.27)	,42.1)	,0.04)	,185.6)	,-0.78)	,3304.8)	,-0.02)	,1234.2)	,0.55)	,1131.4)	,0.55)	,127.2)	,0.57)
	14130	-0.04	235.3	0.23	5124.3	-0.1	407.4		5633.6	0.19	446.3	0.28	48.2	-0.07	357.2	-2.27	2641.7	-0.03	879.1	-0.47	786.9	-0.52	92.9	-0.05
**Cambodia**	(11794.9	(-0.07	(183.9	(0.19	(3921.4	(-0.13	(291.4	0 (0,0)	(3548.5	(0.13	(361	(0.26	(22.5	(-0.08	(130.9	(-2.54	(2111.3	(-0.04	(653.3	(-0.51	(560.7	(-0.57	(69.4	(-0.08
	,17286.5)	,-0.01)	,297.1)	,0.27)	,6620.9)	,-0.07)	,549.5)		,8507.1)	,0.25)	,539.9)	,0.29)	,83.6)	,-0.06)	,592.9)	,-1.99)	,3287.2)	,-0.03)	,1168)	,-0.43)	,1080.2)	,-0.47)	,120.2)	,-0.02)
	16737.7	0.13	203.9	0.07	10429.7	0.19	565.5		3043	0.07	592.7	0.17	23.4	0.02	63.7	1.7	2714.1	-0.01	1025.2	0.16	908.8	0.1	117.5	0.62
**Cameroon**	(13692.5	(0.11	(158.5	(0.05	(7514.6	(0.16	(406.3	0 (0,0)	(1884.1	(0.04	(480.5	(0.13	(9.4	(0.01	(15.8	(1.47	(2168.5	(-0.01	(750.3	(0.09	(634	(0.02	(97.6	(0.58
	,20521.7)	,0.15)	,258.2)	,0.09)	,14140.9)	,0.23)	,757.8)		,4736.7)	,0.11)	,716.4)	,0.21)	,43.9)	,0.02)	,196)	,1.94)	,3380.8)	,-0.01)	,1401.5)	,0.23)	,1278.5)	,0.18)	,140.5)	,0.66)
	11792.2	-0.1	288.6	0	3139.4	-0.5	688.4	0.07	4204.4	-0.01	868.9	0.25	91.8	0.04	215.9	-0.6	2859.5	0.05	1847.3	0.47	1429.8	0.51	424	0.34
**Canada**	(10085.3	(-0.17	(272.4	(-0.01	(2345.2	(-0.68	(523.6	(0.06	(2699.7	(-0.11	(708.2	(0.23	(45.5	(0.02	(39.8	(-0.63	(2325.6	(0.05	(1485.3	(0.36	(1081.5	(0.38	(364.2	(0.32
	,14113.6)	,-0.03)	,305.4)	,0)	,4147.3)	,-0.32)	,889.1)	,0.09)	,6440.3)	,0.09)	,1037.6)	,0.27)	,153.3)	,0.06)	,417.4)	,-0.57)	,3561.5)	,0.06)	,2261.3)	,0.57)	,1836.8)	,0.63)	,495.7)	,0.37)
	17724.2	0.05	171.6	-0.06	10514.9	0.01	600.7		4164.1	0.22	501.2	-0.01	22.3	-0.04	147.3	1.66	2659.1	-0.03	1618.6	-0.3	1529.1	-0.31	91.3	-0.02
**Central African Republic**	(14763.4	(0.03	(129.7	(-0.07	(7649.9	(-0.02	(430.2	0 (0,0)	(2595	(0.17	(400.3	(-0.03	(9.1	(-0.05	(38	(1.46	(2124	(-0.04	(1178.2	(-0.4	(1087.9	(-0.42	(70.8	(-0.03
	,21567.9)	,0.08)	,220.4)	,-0.05)	,14131.2)	,0.03)	,804.6)		,6428.7)	,0.27)	,609.8)	,0)	,41.2)	,-0.02)	,374.9)	,1.85)	,3310.7)	,-0.03)	,2103.9)	,-0.2)	,2016.6)	,-0.21)	,115.5)	,-0.01)
	17922.1	0.09	196.1	0.15	11707.4	0.11	565.7		2971.4	0.04	589.5	0.29	24.4	0.19	73.3	-0.62	2780.2	0.07	978.4	0.26	890.6	0.28	88.7	0.02
**Chad**	(14674.7	(0	(151.8	(0.13	(8470.8	(-0.03	(409.7	0 (0,0)	(1879	(0.01	(473.2	(0.26	(9.8	(0.18	(21.3	(-0.7	(2222.4	(0.07	(730.2	(0.19	(641.9	(0.21	(69.6	(0
	,21886.9)	,0.18)	,249.5)	,0.18)	,15803.6)	,0.26)	,759.4)		,4542.1)	,0.08)	,713.1)	,0.31)	,45.9)	,0.2)	,228.6)	,-0.55)	,3463.1)	,0.08)	,1293.2)	,0.32)	,1203.6)	,0.35)	,110.5)	,0.05)
	13952.1	-0.1	271.9	0.1	4412.5	-0.52	779.7	-0.06	5574.3	0.17	785.3	0.36	49.1	0.02	155.8	-1.89	2700.8	0.01	1908.1	-0.41	1693.5	-0.49	218.7	0.34
**Chile**	(11825.2	(-0.16	(215.3	(0.09	(3257.8	(-0.63	(569	(-0.07	(3653.1	(0.1	(638.5	(0.34	(22.3	(0.01	(29.9	(-1.97	(2177.9	(0.01	(1526	(-0.56	(1310.8	(-0.66	(176.5	(0.28
	,16998.8)	,-0.05)	,339.6)	,0.11)	,5981.9)	,-0.42)	,1045.5)	,-0.05)	,8412.6)	,0.24)	,941.5)	,0.37)	,85.9)	,0.03)	,317.3)	,-1.81)	,3332.5)	,0.01)	,2398.6)	,-0.25)	,2181.9)	,-0.32)	,264.9)	,0.41)
	14463	-0.03	300.5	0.05	6561.2	0.26	223.4	0.01	4609.6	-0.55	507.4	0.61	122.1	-0.36	113.4	-1.58	2809.5	0.01	1178.5	0.25	1038.5	0.73	141.5	-2.44
**China**	(12961.5	(-0.12	(259.7	(0.02	(5503.6	(0.14	(172.3	(-0.01	(3550	(-0.73	(413.8	(0.58	(65.5	(-0.43	(24	(-1.68	(2246.5	(0.01	(881.3	(-0.16	(742.8	(0.26	(111.4	(-2.6
	,16167.3)	,0.05)	,347.2)	,0.09)	,7827)	,0.39)	,283.5)	,0.02)	,6013.7)	,-0.36)	,612.5)	,0.64)	,199.6)	,-0.3)	,226.7)	,-1.48)	,3516.8)	,0.01)	,1552.7)	,0.65)	,1412.2)	,1.21)	,176.1)	,-2.27)
	13162.8	-0.12	245.4	0.09	4127	-0.36	666		5343	-0.03	552.9	0.23	99.5	-0.09	108.3	-1.02	2690.5	-0.03	2206.2	-0.63	2050.1	-0.66	160.3	-0.22
**Colombia**	(10824.6	(-0.22	(192.8	(0.07	(3090.6	(-0.49	(508.2	0 (0,0)	(3341.6	(-0.2	(451	(0.22	(51.9	(-0.1	(12.2	(-1.23	(2149.8	(-0.04	(1532.5	(-0.73	(1377.2	(-0.77	(126.1	(-0.24
	,16096.4)	,-0.03)	,310.2)	,0.11)	,5397.6)	,-0.22)	,859)		,8206.3)	,0.14)	,670.1)	,0.24)	,161.1)	,-0.08)	,236.4)	,-0.81)	,3349.4)	,-0.03)	,3026)	,-0.52)	,2871.5)	,-0.54)	,199.4)	,-0.19)
	16601.3	0.04	196.9	0.01	9479.7	-0.01	650.5		3828.5	0.24	583.9	0.1	22.3	-0.11	105.9	0.85	2690.2	-0.04	1905.3	-0.24	1808.5	-0.26	98.8	0.21
**Comoros**	(13727.2	(-0.01	(154.2	(0	(6904.4	(-0.09	(470.5	0 (0,0)	(2413.6	(0.18	(471.4	(0.08	(9	(-0.12	(19.1	(0.77	(2149.5	(-0.04	(1391.2	(-0.3	(1298.1	(-0.32	(77.4	(0.19
	,19976.7)	,0.1)	,248.3)	,0.03)	,12817.1)	,0.08)	,878.8)		,5832.9)	,0.3)	,703.8)	,0.13)	,41.2)	,-0.09)	,280.5)	,0.94)	,3348.8)	,-0.04)	,2525.3)	,-0.19)	,2427.5)	,-0.21)	,123.9)	,0.23)
	17300	-0.05	200.1	0.12	10341.4	-0.13	601.8		3769.6	0.02	588.8	0.41	22.8	0.13	70.4	1.04	2706.4	0.05	1774.6	0.01	1668.4	-0.01	108.5	0.43
**Congo**	(14303.4	(-0.07	(157.7	(0.09	(7459.7	(-0.17	(435.8	0 (0,0)	(2368.3	(-0.03	(474.4	(0.38	(9.2	(0.12	(16.7	(0.88	(2162	(0.05	(1261.5	(-0.1	(1157.9	(-0.13	(87.2	(0.39
	,21054.1)	,-0.02)	,250.7)	,0.15)	,13977.1)	,-0.09)	,806.2)		,5804.5)	,0.07)	,705.7)	,0.45)	,41.9)	,0.14)	,199.3)	,1.2)	,3371)	,0.06)	,2375.6)	,0.13)	,2268.3)	,0.1)	,133.5)	,0.48)
	13494	0.07	299	0.04	5141.4	0.07	358.6		4954.3	0.17	572.3	0.13	69.3	-0.13	69.3	-0.68	2725.6	-0.05	1137.9	-0.58	1005.5	-0.65	133.7	-0.07
**Cook Islands**	(11364	(0.06	(238.5	(0.03	(3848.8	(0.06	(259.4	0 (0,0)	(3135.2	(0.14	(465.1	(0.1	(34	(-0.16	(12.1	(-0.85	(2177.5	(-0.06	(858.1	(-0.63	(726.5	(-0.7	(99.9	(-0.1
	,16189.5)	,0.09)	,370.1)	,0.06)	,6780.5)	,0.09)	,484.7)		,7343.7)	,0.21)	,695.7)	,0.15)	,116.1)	,-0.1)	,166)	,-0.51)	,3395.1)	,-0.05)	,1485.8)	,-0.54)	,1356.3)	,-0.6)	,174)	,-0.04)
	14396.2	0.1	249.9	0.11	5984.1	0.14	759.1		4716.3	0.2	583.9	0.12	102	-0.06	107.7	-0.93	2698.8	-0.03	2093.8	-0.51	1974	-0.53	122.9	-0.14
**Costa Rica**	(12010.3	(0.07	(196.3	(0.1	(4406.7	(0.1	(557.8	0 (0,0)	(2964.9	(0.12	(471.2	(0.11	(54	(-0.07	(16.3	(-1.04	(2156.3	(-0.03	(1525.8	(-0.66	(1398.8	(-0.7	(94.5	(-0.19
	,17199.6)	,0.14)	,317.1)	,0.12)	,8023.5)	,0.19)	,1017.8)		,7149.7)	,0.28)	,707.6)	,0.12)	,170.4)	,-0.05)	,233)	,-0.82)	,3359.9)	,-0.02)	,2763.1)	,-0.35)	,2645.6)	,-0.36)	,158.1)	,-0.1)
	15234.8	-0.2	221.3	0.14	6689.6	-0.54	549.3	-0.01	5035.8	0.04	764	0.4	62.9	0.1	129.1	-1.51	2681.1	0.06	4296	-0.09	4137.5	-0.11	165.6	0.73
**Croatia**	(12719.1	(-0.26	(175.4	(0.13	(4825.8	(-0.63	(398.4	(-0.01	(3111.6	(-0.04	(624.7	(0.39	(30.5	(0.09	(18.5	(-1.68	(2141.5	(0.06	(3297.5	(-0.21	(3138	(-0.24	(131.7	(0.64
	,18400.1)	,-0.14)	,275.2)	,0.16)	,8865.3)	,-0.45)	,733)	,-0.01)	,7757)	,0.12)	,923.7)	,0.4)	,104.4)	,0.11)	,281.9)	,-1.34)	,3338.9)	,0.06)	,5521.3)	,0.04)	,5364.7)	,0.02)	,204.1)	,0.82)

	16303.9	-0.08	233.9	0.04	7562.1	-0.29	830.3		5102.9	0.15	527	0.1	227.3	-0.07	136.5	0.24	2705.3	-0.02	2325.6	0.95	2186.1	1.04	143.6	-0.22
**Cuba**	(13714.5	(-0.14	(183.5	(0.01	(5592	(-0.38	(610	0 (0,0)	(3286	(0.07	(430.2	(0.09	(129.1	(-0.07	(16.7	(-0.18	(2161.5	(-0.03	(1876.3	(0.82	(1744.3	(0.89	(109.4	(-0.24
	,19432.2)	,-0.03)	,292.9)	,0.07)	,10201.6)	,-0.2)	,1108)		,7858.9)	,0.23)	,634.4)	,0.11)	,354.2)	,-0.06)	,297.1)	,0.66)	,3368.9)	,-0.02)	,2861.2)	,1.08)	,2720.1)	,1.18)	,186.9)	,-0.19)
	15049.2	0.01	293.1	-0.01	4424.8	-0.09	1051.7	0.05	6542.8	0.05	657.3	0.45	58.5	0.11	180.4	-1.19	2732.1	0.04	1345.8	-0.21	1198.5	-0.25	149.4	0.17
**Cyprus**	(12569.4	(-0.02	(232.9	(-0.02	(3296.9	(-0.14	(776.1	(0.04	(4192.9	(0.01	(536.2	(0.42	(26.7	(0.1	(44.2	(-1.32	(2204.2	(0.03	(996.9	(-0.32	(851	(-0.38	(122.1	(0.15
	,18302.7)	,0.04)	,364.1)	,0)	,5790.6)	,-0.03)	,1411.1)	,0.07)	,9883.4)	,0.1)	,790.4)	,0.48)	,100.4)	,0.13)	,343.9)	,-1.06)	,3371.2)	,0.04)	,1800)	,-0.1)	,1649.8)	,-0.13)	,181)	,0.18)
	14271.5	-0.24	224.2	0.13	6045.7	-0.62	549.2	-0.01	4625.4	-0.03	765.3	0.38	63.3	0.1	105.9	-1.4	2688.4	0.05	3521.7	0.82	3373	0.82	154.5	0.8
**Czech Republic**	(12008.4	(-0.3	(177.5	(0.12	(4465.5	(-0.73	(401.2	(-0.01	(2874.8	(-0.1	(620.2	(0.36	(30.8	(0.1	(14.8	(-1.5	(2147.5	(0.05	(2670.2	(0.68	(2520	(0.67	(119.5	(0.78
	,17035)	,-0.18)	,280)	,0.13)	,7854.3)	,-0.5)	,740.2)	,-0.01)	,7007.9)	,0.05)	,916)	,0.41)	,105.1)	,0.1)	,239.9)	,-1.29)	,3348.5)	,0.05)	,4548.3)	,0.96)	,4407.5)	,0.97)	,193.7)	,0.82)
	15156.2	0.08	204.6	0.07	8688.9	0.1	566.7		3006.2	0.11	613.2	0.18	24	-0.01	90.6	1.1	2745.3	-0.02	1024.3	-0.01	928.2	-0.02	97	0.14
**C 涔块 e d'Ivoire**	(12619.9	(0.07	(160.2	(0.04	(6336.6	(0.08	(409.2	0 (0,0)	(1860.3	(0.08	(491.7	(0.15	(9.6	(-0.03	(14.9	(0.92	(2193.8	(-0.03	(780.5	(-0.06	(684.8	(-0.09	(77.8	(0.13
	,18301)	,0.09)	,259.3)	,0.09)	,11544.5)	,0.12)	,759.6)		,4620.8)	,0.14)	,743.7)	,0.22)	,45.1)	,0.01)	,242.8)	,1.28)	,3420.8)	,-0.01)	,1339.8)	,0.05)	,1240.6)	,0.04)	,119.6)	,0.15)
	14189.9	-0.06	236.8	-0.34	6049.8	-0.2	237.7		4898.8	-0.04	494.8	0.37	87.8	0.17	300.5	1.34	2642.1	0.05	705.5	0.13	554.6	0.29	151.7	-0.39
**Democratic People's Republic of Korea**	(11936.2	(-0.07	(187.5	(-0.35	(4518.2	(-0.21	(169.4	0 (0,0)	(3120.1	(-0.07	(403.3	(0.33	(45	(0.15	(85.3	(1.18	(2110.1	(0.05	(541.1	(0.12	(391.3	(0.27	(120.4	(-0.42
	,16843.3)	,-0.04)	,293.8)	,-0.32)	,7895.5)	,-0.18)	,327.9)		,7379.8)	,-0.02)	,598.5)	,0.4)	,142)	,0.19)	,516)	,1.51)	,3290.2)	,0.06)	,916)	,0.15)	,758.4)	,0.31)	,185.9)	,-0.36)
	17129.1	0.07	180.2	-0.1	10055.2	0.04	602		3846.1	0.15	556.6	0.12	22.5	0.05	190.9	3.13	2671.3	-0.04	1547.3	-0.11	1453.1	-0.14	95.9	0.25
**Democratic Republic of the Congo**	(14292.4	(0.05	(138.8	(-0.16	(7331.8	(0.02	(437	0 (0,0)	(2379.1	(0.11	(447.1	(0.05	(9.1	(0.02	(34.4	(2.6	(2133.6	(-0.06	(1130.5	(-0.27	(1035.4	(-0.3	(75.1	(0.18
	,20826.4)	,0.09)	,229.1)	,-0.04)	,13574.8)	,0.07)	,809.1)		,5838.3)	,0.19)	,671.1)	,0.19)	,41.4)	,0.08)	,431.1)	,3.67)	,3326.4)	,-0.02)	,2052.1)	,0.05)	,1953.2)	,0.03)	,120.6)	,0.31)
	14850.3	-0.19	365.3	1	5222.5	-0.72	1057	0.09	5580.7	0.02	692.4	0.5	57.3	-0.02	29.1	-1.64	2726.5	0.05	3642.2	1.33	3427.2	1.37	223.5	0.8
**Denmark**	(12546	(-0.25	(316.5	(0.81	(3874.8	(-0.87	(780.1	(0.07	(3648.8	(-0.03	(570.8	(0.48	(25.9	(-0.03	(3.4	(-1.75	(2199.3	(0.05	(2751.6	(1.1	(2539.3	(1.12	(189.3	(0.75
	,17698.7)	,-0.13)	,422.4)	,1.19)	,7021.8)	,-0.58)	,1405.4)	,0.11)	,8306.4)	,0.08)	,828.7)	,0.51)	,98.7)	,-0.02)	,68.9)	,-1.54)	,3364.4)	,0.05)	,4921.4)	,1.56)	,4700.6)	,1.62)	,261.3)	,0.84)
	16627.9	0.04	198.6	0.02	9758	0.02	644.8		3468.9	0.04	628.8	0.29	24	0.1	85.2	0.72	2762.2	0.05	2412.8	0.19	2307.4	0.17	108	0.5
**Djibouti**	(13781.5	(0.01	(155.4	(-0.01	(7167.9	(-0.01	(466.7	0 (0,0)	(2175.5	(-0.01	(508.8	(0.25	(9.6	(0.09	(15	(0.56	(2207.7	(0.04	(1765.3	(0.11	(1659.6	(0.1	(85.1	(0.48
	,19974.8)	,0.06)	,249.5)	,0.04)	,13011.7)	,0.05)	,866.7)		,5243.1)	,0.09)	,755.9)	,0.33)	,43.6)	,0.11)	,243.3)	,0.88)	,3442.8)	,0.05)	,3193.3)	,0.26)	,3090.6)	,0.25)	,136)	,0.52)
	14095.3	0.02	233.8	0.17	5227.3	-0.03	818.4	-0.02	5045.8	-0.03	477.7	0.38	238.8	0.63	127.4	-0.48	2712.3	0.12	2756.5	0.51	2579.1	0.52	183.1	0.44
**Dominica**	(11879.9	(-0.02	(182.2	(0.15	(3886.1	(-0.1	(597.1	(-0.02	(3193	(-0.09	(388.6	(0.33	(136.8	(0.52	(16.8	(-0.53	(2166.7	(0.09	(2003.1	(0.35	(1832.4	(0.34	(140.9	(0.41
	,17017.7)	,0.06)	,294.8)	,0.19)	,7122.7)	,0.05)	,1100.8)	,-0.01)	,7685.7)	,0.03)	,573.7)	,0.43)	,372.3)	,0.74)	,264.5)	,-0.44)	,3381.4)	,0.14)	,3732.7)	,0.68)	,3559.6)	,0.7)	,234.4)	,0.46)
	15809.2	0.14	239.4	0.19	7152.2	0.26	820.2		5039.4	0.14	464.7	0.14	229.7	-0.01	109.9	-1.5	2715.6	-0.01	1591.8	-0.11	1465.9	-0.14	128.1	0.21
**Dominican Republic**	(13268.9	(0.07	(186.1	(0.18	(5248.5	(0.11	(598.2	0 (0,0)	(3173.4	(0.07	(377.6	(0.1	(130.6	(-0.02	(12.8	(-1.57	(2169.6	(-0.01	(1141.4	(-0.23	(1020.1	(-0.26	(99.7	(0.18
	,19079.8)	,0.2)	,301.7)	,0.2)	,9715.7)	,0.41)	,1107.3)		,7714.9)	,0.21)	,557.9)	,0.17)	,357.9)	,0)	,232.5)	,-1.44)	,3382.3)	,-0.01)	,2127.3)	,0)	,1999.9)	,-0.01)	,163.8)	,0.23)
	15692	0.19	240.6	0.1	6069.9	0.35	825		6014.7	0.22	480.6	0.24	155.9	-0.02	155	-0.8	2711.2	-0.01	1608.2	-1.27	1483.7	-1.37	126.7	0.27
**Ecuador**	(13110.1	(0.13	(188	(0.09	(4434.6	(0.23	(606	0 (0,0)	(3852.6	(0.12	(387.6	(0.22	(85.6	(-0.03	(27.7	(-0.9	(2166.2	(-0.02	(1153.7	(-1.44	(1028	(-1.55	(101.4	(0.25
	,18816.9)	,0.25)	,303.9)	,0.11)	,7921.3)	,0.48)	,1099.2)		,9078.2)	,0.32)	,581.3)	,0.27)	,251.4)	,-0.02)	,290.9)	,-0.71)	,3376.5)	,-0.01)	,2172.5)	,-1.1)	,2047.2)	,-1.18)	,159.6)	,0.28)
	14302.8	0.07	217.4	0.19	6013.9	0.24	704	-0.01	4001.9	0.13	559.1	0.35	101.8	0.07	748.6	-1.3	2782.8	0.04	446.6	0.18	321.2	-0.04	125.7	0.81
**Egypt**	(12169.6	(0.01	(171.8	(0.18	(4441.9	(0.15	(513.7	(-0.01	(2520.2	(0.02	(452.7	(0.33	(56.3	(0.05	(332.4	(-1.36	(2224	(0.04	(326.3	(0.13	(206.2	(-0.11	(101.1	(0.78
	,16928.4)	,0.12)	,275.8)	,0.19)	,7941.8)	,0.33)	,937.5)	,-0.01)	,6132.3)	,0.24)	,674)	,0.37)	,160.7)	,0.09)	,1158.1)	,-1.24)	,3465.4)	,0.05)	,592.9)	,0.23)	,468.4)	,0.03)	,155.3)	,0.84)
	14462.8	0.02	232	0.11	5986.2	-0.12	747		4878.4	0.26	518.9	0.25	99	-0.1	141.5	-1.09	2668.5	-0.04	4182.8	-0.93	4084.3	-0.95	104	0.09
**El Salvador**	(12022.4	(-0.03	(183.2	(0.11	(4399.4	(-0.19	(551	0 (0,0)	(3095.9	(0.19	(420.3	(0.21	(52.2	(-0.11	(17.8	(-1.18	(2133.3	(-0.04	(2958.5	(-1.1	(2864.7	(-1.12	(81.2	(0.07
	,17383.4)	,0.07)	,294.9)	,0.12)	,8103)	,-0.05)	,998.8)		,7252.7)	,0.34)	,626.9)	,0.28)	,165.7)	,-0.1)	,284.3)	,-1.01)	,3320.2)	,-0.04)	,5654.5)	,-0.76)	,5559.9)	,-0.78)	,131)	,0.1)
**Equatorial Guinea**	17550.1	0.06	225	0.88	10579.4	0.05	603.1	0 (0,0)	3863.7	0.09	598.8	0.62	21.9	-0.07	19.9	-7.23	2662.5	-0.01	1585.2	-0.02	1479.2	-0.07	108	0.57
	(14386.5	(0.02	(178	(0.74	(7654.6	(0.01	(436.5		(2434.8	(0.05	(485.1	(0.58	(8.8	(-0.08	(4.1	(-8.44	(2128.1	(-0.01	(1163.7	(-0.1	(1057.4	(-0.14	(85.3	(0.54

	,21764.4)	,0.09)	,281.3)	,1.03)	,14491)	,0.1)	,816.6)		,5945.5)	,0.13)	,717.7)	,0.67)	,40.6)	,-0.06)	,73.9)	,-6.01)	,3313.3)	,0)	,2059.1)	,0.05)	,1954.1)	,0.01)	,134.5)	,0.6)
	17957.4	-0.03	188.1	0.09	10898	-0.1	645.1		3998.6	0.11	528.3	0.15	21.6	-0.07	121.5	0.21	2628.7	-0.01	2245.9	0.07	2152.6	0.07	95.5	0.11
**Eritrea**	(14942.6	(-0.05	(145.8	(0.06	(7893.1	(-0.13	(465.6	0 (0,0)	(2543.6	(0.07	(425.9	(0.13	(8.7	(-0.09	(22.2	(-0.35	(2101	(-0.01	(1659.4	(0.03	(1563.8	(0.02	(73.7	(0.08
	,21796.7)	,-0.01)	,240.4)	,0.12)	,14863.6)	,-0.07)	,864)		,6009.3)	,0.15)	,641.7)	,0.16)	,40.2)	,-0.06)	,303.7)	,0.77)	,3270.9)	,-0.01)	,2964.5)	,0.12)	,2870.1)	,0.12)	,121.5)	,0.13)
	16692.1	-0.34	207.5	0.25	8613.4	-0.71	558.4	-0.01	4824.2	0	703.6	0.45	61.2	0.1	104.5	-2.26	2640.3	0.05	3177.4	0.36	2951.8	0.38	233.8	0.14
**Estonia**	(13919.9	(-0.41	(163.8	(0.23	(6297.4	(-0.83	(407.3	(-0.01	(3043.7	(-0.08	(579.6	(0.44	(29.7	(0.09	(13.6	(-2.44	(2109.3	(0.05	(2471.6	(0.11	(2246.9	(0.11	(193	(0.1
	,19906.7)	,-0.27)	,259.5)	,0.27)	,11451.2)	,-0.59)	,745.6)	,-0.01)	,7300.9)	,0.07)	,841.3)	,0.47)	,101.6)	,0.11)	,233.9)	,-2.08)	,3287)	,0.05)	,4121)	,0.61)	,3888.9)	,0.64)	,281.1)	,0.19)
	16783.7	0.18	206.6	0.14	9366.3	0.29	600.3		4343.8	0.22	544.7	-0.04	21.5	-0.14	70.6	-0.23	2614.2	-0.06	2139.7	-0.25	1948.5	-0.3	196.5	0.28
**Eswatini**	(13931.4	(0.14	(163.3	(0.13	(6762.6	(0.21	(434.1	0 (0,0)	(2736.6	(0.12	(437.7	(-0.08	(8.7	(-0.15	(13	(-0.5	(2089.6	(-0.06	(1510.9	(-0.37	(1329.3	(-0.42	(163.8	(0.23
	,20298.7)	,0.23)	,261)	,0.15)	,12636.7)	,0.37)	,810.5)		,6763.8)	,0.32)	,660)	,0)	,40.1)	,-0.13)	,181.2)	,0.03)	,3253.4)	,-0.05)	,2911.4)	,-0.14)	,2713.9)	,-0.18)	,231.4)	,0.33)
	17579.6	-0.06	190.6	0.24	10415.1	-0.15	680		3782.5	0.05	593.8	0.36	18	-0.09	111	1.09	2837.1	-0.02	3752.7	-0.36	3652.9	-0.37	104.5	-0.04
**Ethiopia**	(15165.3	(-0.12	(156.3	(0.19	(8126.5	(-0.26	(534.4	0 (0,0)	(2865.8	(-0.04	(481.2	(0.35	(6.4	(-0.11	(21.7	(0.76	(2268.8	(-0.03	(2853.6	(-0.44	(2754.4	(-0.45	(82.2	(-0.06
	,20190.8)	,0)	,229.6)	,0.29)	,12992.4)	,-0.03)	,850.2)		,5096.2)	,0.14)	,714.4)	,0.36)	,34.7)	,-0.07)	,300.3)	,1.43)	,3552.3)	,-0.01)	,4818.7)	,-0.28)	,4718.2)	,-0.28)	,130.7)	,-0.02)
	13433.8	0.07	266.7	0.01	4970.1	0.09	353.6		5151.4	0.14	472.2	-0.01	68.2	-0.03	152.5	0.03	2687.1	-0.03	654.5	-0.4	525.7	-0.46	129.4	-0.14
**Fiji**	(11184.3	(0.05	(211.2	(0	(3762.1	(0.07	(250	0 (0,0)	(3284.1	(0.1	(380.6	(-0.02	(33.5	(-0.04	(33.3	(-0.07	(2146.5	(-0.04	(477.5	(-0.45	(354.7	(-0.52	(96.3	(-0.17
	,16390.2)	,0.09)	,334.1)	,0.02)	,6492.1)	,0.12)	,481.8)		,7925.2)	,0.18)	,569.8)	,0.01)	,114.3)	,-0.02)	,302.2)	,0.13)	,3347)	,-0.03)	,882.4)	,-0.34)	,756.7)	,-0.4)	,169)	,-0.11)
	13957.3	0.19	317.7	-0.17	5538.3	0.02	1052.3	0.06	4265.9	0.67	583.9	0.43	77.5	0.16	184.6	-1.19	2715	0.09	2029.7	1.16	1764.2	1.16	270.9	1.13
**Finland**	(11922.2	(0.1	(250.4	(-0.2	(4168.5	(-0.09	(786.2	(0.04	(2684.4	(0.38	(476.4	(0.4	(37.6	(0.15	(42.7	(-1.33	(2189.8	(0.08	(1632.5	(1.03	(1365.9	(1.04	(235.3	(0.94
	,16548.9)	,0.29)	,397.1)	,-0.14)	,7361.3)	,0.14)	,1400.3)	,0.07)	,6432.5)	,0.95)	,706.7)	,0.47)	,131.6)	,0.17)	,344.1)	,-1.06)	,3350.6)	,0.09)	,2588.9)	,1.28)	,2317.2)	,1.29)	,308.7)	,1.32)
	16551.7	-0.06	290.2	-0.02	5508.9	-0.11	1050.3	0.06	7196.2	-0.09	643	0.35	35.1	0.03	198	-1.11	2707.7	0.04	2118.9	0.21	1830.5	0	293.6	1.84
**France**	(13816.2	(-0.12	(228.1	(-0.03	(4087.4	(-0.33	(774.7	(0.04	(4653.7	(-0.25	(524.7	(0.33	(14.5	(0.02	(57.2	(-1.14	(2183.6	(0.04	(1687.5	(0.14	(1399.6	(-0.08	(249.4	(1.74
	,19949.2)	,0)	,364)	,-0.01)	,7265.2)	,0.11)	,1404)	,0.07)	,10692.1)	,0.06)	,770.2)	,0.38)	,64.4)	,0.04)	,368.7)	,-1.08)	,3341.4)	,0.04)	,2608)	,0.28)	,2314)	,0.08)	,344.9)	,1.93)
	17688.3	0	217.3	0.03	10566.2	-0.05	603.4		3973.4	0.07	619.5	0.42	23.1	0.21	28.7	0.78	2703.8	0.05	1908.1	0.12	1795.8	0.11	115	0.37
**Gabon**	(14417.6	(-0.02	(171.8	(0.02	(7658.7	(-0.08	(437	0 (0,0)	(2477.1	(0.01	(503.9	(0.37	(9.3	(0.18	(6.4	(0.7	(2159.9	(0.03	(1417.5	(0.02	(1302.1	(0	(92.3	(0.32
	,21560.4)	,0.03)	,271.9)	,0.03)	,14422.1)	,-0.02)	,810.5)		,6148.4)	,0.12)	,745.8)	,0.46)	,42.3)	,0.24)	,104.1)	,0.86)	,3369)	,0.06)	,2552.8)	,0.23)	,2442.1)	,0.22)	,142.1)	,0.41)
	19638.6	0.07	199.4	0.05	13426.4	0.09	567		3144.4	0.13	601.6	0.06	23.5	-0.12	96.2	1.92	2712.9	-0.04	996.7	0.23	901.5	0.25	96.1	0.04
**Gambia**	(16203.4	(0.05	(154.8	(0.04	(9791.2	(0.06	(411.5	0 (0,0)	(1953.5	(0.08	(486.1	(0.05	(9.5	(-0.13	(20.1	(1.74	(2167.3	(-0.04	(729.7	(0.17	(632.9	(0.19	(77.1	(0.03
	,23838.7)	,0.1)	,252.7)	,0.06)	,17761.7)	,0.12)	,758.1)		,4796.3)	,0.18)	,726.3)	,0.08)	,44.2)	,-0.11)	,273.3)	,2.09)	,3380.1)	,-0.04)	,1311.8)	,0.29)	,1215.4)	,0.32)	,119.1)	,0.05)
	14961.6	0.02	204.1	0.12	8269.9	0.04	553.8		3175.9	0.03	630.5	0.09	61	0	210.1	-0.94	2646.8	0.02	2158.9	-1.09	2023.4	-1.14	138.5	-0.24
**Georgia**	(12491.2	(-0.02	(163.5	(0.04	(6084.8	(-0.01	(400.9	0 (0,0)	(1961.9	(-0.05	(515.9	(0.07	(29.6	(-0.02	(41.1	(-1.67	(2114.8	(0.02	(1535.9	(-1.19	(1397.2	(-1.25	(108.5	(-0.28
	,17952.4)	,0.06)	,256.7)	,0.19)	,10971.9)	,0.1)	,746)		,4941.2)	,0.11)	,757)	,0.11)	,101.2)	,0.01)	,395.8)	,-0.21)	,3294.2)	,0.03)	,2943.4)	,-0.98)	,2808.5)	,-1.02)	,175.6)	,-0.21)
	16520.7	0.3	268.9	-0.08	5119.5	0.35	948	0.07	7743	0.46	648.2	0.44	22.1	0.12	94.3	-1.89	2716.6	0.1	2063.7	0.7	1897.4	0.73	169.9	0.45
**Germany**	(13688.1	(0.16	(213.3	(-0.1	(3845.5	(0.21	(723.7	(0.05	(4916.5	(0.18	(524.8	(0.38	(8.8	(0.09	(13.9	(-1.98	(2191.1	(0.09	(1631.9	(0.61	(1471.1	(0.63	(141.2	(0.44
	,20011.4)	,0.44)	,334.6)	,-0.06)	,6795.5)	,0.49)	,1224.6)	,0.09)	,11336.5)	,0.74)	,785.1)	,0.49)	,42.2)	,0.14)	,215.1)	,-1.81)	,3352.6)	,0.1)	,2651.6)	,0.79)	,2495.4)	,0.83)	,201.8)	,0.47)
	16268.9	0.11	212.1	0.23	10007.8	0.15	567.1		2997.5	0.14	586.2	0.07	22.6	-0.1	59.4	0.68	2672	-0.05	991.8	0.1	870.1	0.01	122.9	0.8
**Ghana**	(13425.8	(0.08	(166.5	(0.21	(7347.5	(0.12	(411.2	0 (0,0)	(1879.8	(0.09	(475.9	(0.06	(9.1	(-0.11	(13.3	(0.45	(2135.2	(-0.06	(745.9	(0.04	(625.1	(-0.06	(101.2	(0.76
	,19764.1)	,0.13)	,266.7)	,0.25)	,13538.5)	,0.18)	,767.2)		,4553.6)	,0.19)	,703.1)	,0.09)	,42.3)	,-0.08)	,182.5)	,0.91)	,3326.1)	,-0.05)	,1274.8)	,0.16)	,1154.4)	,0.08)	,147.7)	,0.84)
	17915.6	-0.09	290.1	-0.02	7750.6	-0.32	1055.2	0.09	6464.2	0.11	639.4	0.12	57.6	0.02	196.9	-0.94	2715.6	0.01	1024.5	-0.31	908.5	-0.38	117.1	0.31
**Greece**	(14861.1	(-0.19	(229.1	(-0.03	(5593.9	(-0.56	(781.5	(0.08	(4150.5	(0.02	(519.3	(0.1	(26.2	(0.01	(47	(-1.16	(2190.4	(0.01	(718.9	(-0.44	(600.1	(-0.53	(92.3	(0.24
	,21538.1)	,0.01)	,362)	,-0.02)	,10546.1)	,-0.07)	,1419.6)	,0.11)	,9635.5)	,0.21)	,774.6)	,0.14)	,98.9)	,0.03)	,370.9)	,-0.73)	,3350.9)	,0.01)	,1433.1)	,-0.17)	,1314.3)	,-0.23)	,145.8)	,0.38)

	13588.6	-0.05	328	0.1	4538.7	-0.22	686	0.01	4858.2	-0.11	742.7	0.53	100.9	0.23	138.6	-1.13	2943	0.18	3129.3	-0.38	2845.6	-0.4	292.7	-0.22
**Greenland**	(11419.2	(-0.09	(256.3	(0.06	(3328	(-0.27	(499.9	(0.01	(3098.1	(-0.2	(604.1	(0.51	(50	(0.19	(26.1	(-1.31	(2391.5	(0.16	(2306.6	(-0.48	(2024.5	(-0.5	(238.6	(-0.3
	,16310.4)	,-0.01)	,415.5)	,0.14)	,6052.3)	,-0.17)	,923.4)	,0.02)	,7398.4)	,-0.02)	,893.8)	,0.56)	,166.3)	,0.27)	,287.6)	,-0.96)	,3662.5)	,0.19)	,4057.8)	,-0.29)	,3767.4)	,-0.31)	,355)	,-0.14)
	14340.5	0.05	237.4	0.2	5464.1	0.03	815.6	0	5116.1	0.12	478.5	0.25	235.4	0.26	110.8	-1.18	2693.1	0.02	2490	0.42	2328.4	0.43	166.7	0.35
**Grenada**	(11889.7	(0.02	(186	(0.2	(4042.7	(-0.03	(602.4	(-0.01	(3180.2	(0.06	(389.5	(0.23	(134.3	(0.24	(14.2	(-1.24	(2151.4	(0	(1820.7	(0.24	(1657.3	(0.24	(129.6	(0.32
	,17434.4)	,0.09)	,300.7)	,0.21)	,7394.8)	,0.09)	,1085)	,0)	,7851.4)	,0.17)	,575)	,0.28)	,366.8)	,0.28)	,236.2)	,-1.12)	,3356.6)	,0.04)	,3353.8)	,0.61)	,3189.1)	,0.62)	,212)	,0.38)
	13654	0.08	309.4	-0.08	5208.6	0.09	358.2		5120	0.17	541	-0.01	68.8	-0.07	48.7	-0.05	2708	-0.03	870.5	-0.49	727.4	-0.56	144.2	-0.08
**Guam**	(11443.8	(0.05	(248.2	(-0.09	(3910.4	(0.05	(254.8	0 (0,0)	(3275.5	(0.12	(437.3	(-0.04	(33.7	(-0.1	(7.7	(-0.14	(2163.4	(-0.04	(644.2	(-0.54	(505.1	(-0.63	(109.7	(-0.1
	,16555.7)	,0.11)	,383)	,-0.07)	,6837.6)	,0.12)	,487.8)		,7640.6)	,0.23)	,656.5)	,0.02)	,115.2)	,-0.04)	,130)	,0.03)	,3373.5)	,-0.03)	,1158.8)	,-0.43)	,1018.8)	,-0.49)	,183.8)	,-0.06)
	15538.4	-0.02	230.8	0.08	7354.8	-0.15	738		4692.2	0.23	494.9	0.23	102.2	-0.08	133.9	-1.04	2705.8	-0.03	4109.4	-1.02	4010.7	-1.04	103.8	-0.12
**Guatemala**	(12876.9	(-0.14	(180.4	(0.08	(5401.3	(-0.43	(539.4	0 (0,0)	(2945.1	(0.15	(399.5	(0.2	(54.1	(-0.09	(17.4	(-1.13	(2162	(-0.03	(2988.7	(-1.18	(2892.2	(-1.21	(82.8	(-0.24
	,18851.7)	,0.11)	,290.2)	,0.08)	,9909.8)	,0.13)	,983.5)		,7081)	,0.3)	,602)	,0.26)	,170.7)	,-0.08)	,276.1)	,-0.95)	,3368.5)	,-0.03)	,5595.5)	,-0.85)	,5497.2)	,-0.87)	,127.7)	,0.01)
	16078.5	0.11	198	0.03	9638.3	0.17	565.9		3106.2	0.02	587.9	0.22	23.9	0.1	62.8	1.03	2750.6	0.03	969.2	0.24	881	0.26	89	0.12
**Guinea**	(13384.3	(0.09	(154.5	(0.01	(7017.5	(0.14	(409.4	0 (0,0)	(1961.1	(-0.02	(469.9	(0.19	(9.6	(0.08	(19.7	(0.82	(2198.2	(0.02	(717.2	(0.17	(627.6	(0.17	(71	(0.1
	,19385.1)	,0.13)	,250.3)	,0.05)	,12855.3)	,0.2)	,760.5)		,4762.9)	,0.07)	,708.1)	,0.25)	,44.9)	,0.12)	,202.9)	,1.25)	,3426.6)	,0.04)	,1291.6)	,0.32)	,1201.8)	,0.35)	,110.8)	,0.13)
	16243	0.17	194.1	0.06	9957.8	0.26	564.4		3042.6	0.14	544.7	0.1	23.1	-0.03	82.9	2.26	2690.2	-0.04	1031.9	0.11	943.5	0.11	89.3	0.03
**Guinea-Bissau**	(13310.7	(0.16	(150.4	(0.05	(7192.1	(0.23	(409.8	0 (0,0)	(1910	(0.09	(439.6	(0.07	(9.3	(-0.04	(21.8	(1.93	(2149.1	(-0.05	(772.3	(0.03	(683	(0.03	(70.3	(0.02
	,19705)	,0.19)	,249.2)	,0.06)	,13372.7)	,0.28)	,751.4)		,4671.6)	,0.18)	,659.3)	,0.14)	,43.2)	,-0.01)	,245.6)	,2.6)	,3350.3)	,-0.03)	,1374.1)	,0.18)	,1288)	,0.19)	,110.5)	,0.04)
	16614.4	0.15	225.6	0.17	7879.8	0.28	806		5260.5	0.14	431.6	0.09	226.6	-0.02	138.2	-1.02	2699.2	-0.01	2195.1	-0.3	2073	-0.31	125.2	-0.13
**Guyana**	(13873	(0.11	(176.2	(0.16	(5754.7	(0.23	(586.4	0 (0,0)	(3265.7	(0.06	(348	(0.07	(128.8	(-0.03	(19.1	(-1.11	(2156.4	(-0.02	(1614.4	(-0.41	(1495.7	(-0.42	(96.3	(-0.16
	,19840)	,0.19)	,286.1)	,0.18)	,10817.7)	,0.33)	,1076.6)		,7946.1)	,0.23)	,523)	,0.1)	,353.1)	,-0.01)	,273.9)	,-0.92)	,3361.3)	,-0.01)	,2918.7)	,-0.2)	,2800.4)	,-0.2)	,162)	,-0.1)
	14799	0.08	200.6	-0.06	5907.6	0.05	804.4		5150	0.16	390.7	0.14	226.5	-0.07	266	0.54	2715.7	0.01	2451.5	-0.24	2310.2	-0.26	145.5	0.04
**Haiti**	(12390.3	(0.03	(156.2	(-0.07	(4405.3	(-0.02	(587.2	0 (0,0)	(3243.6	(0.09	(315.7	(0.13	(128.5	(-0.08	(57.2	(0.44	(2169.8	(0	(1743.8	(-0.36	(1601.1	(-0.39	(112	(0.02
	,17847.5)	,0.12)	,256.8)	,-0.05)	,7994.9)	,0.11)	,1073)		,7886.4)	,0.24)	,472.9)	,0.15)	,353.1)	,-0.05)	,490.9)	,0.63)	,3381.2)	,0.01)	,3352.8)	,-0.13)	,3214)	,-0.14)	,188.2)	,0.05)
	15247.2	0.2	225.2	0.05	6951.9	0.35	740.2		4737	0.24	488.8	0.14	102.4	-0.05	183.5	-0.58	2705.4	-0.01	3709	0.56	3524.9	0.54	194.1	1.03
**Honduras**	(12687.7	(0.17	(176.2	(0.04	(5037.6	(0.3	(539.9	0 (0,0)	(2968.7	(0.14	(395.4	(0.12	(54.2	(-0.06	(30.3	(-0.7	(2161.6	(-0.02	(2598.5	(0.38	(2412.2	(0.35	(161.8	(0.99
	,18492.7)	,0.24)	,282)	,0.05)	,9463.7)	,0.4)	,985.8)		,7552.5)	,0.35)	,587.7)	,0.16)	,171)	,-0.04)	,352.5)	,-0.45)	,3368.4)	,0)	,5015.1)	,0.74)	,4845.8)	,0.73)	,232)	,1.08)
	14745	-0.16	221	0.16	6329.6	-0.45	541.7		4940.9	0.06	716.9	0.29	61.8	0.04	118	-1.4	2657.6	0.01	3753.5	-0.75	3637.1	-0.77	121.2	0.13
**Hungary**	(12341.9	(-0.24	(174	(0.15	(4580	(-0.62	(392.5	0 (0,0)	(3157.3	(0	(584.3	(0.28	(30	(0.03	(16.4	(-1.5	(2123.1	(0	(2823.9	(-0.91	(2708.7	(-0.94	(93.9	(0.09
	,17639.2)	,-0.09)	,277.5)	,0.17)	,8506.6)	,-0.29)	,728)		,7469.6)	,0.13)	,860.8)	,0.3)	,102.4)	,0.06)	,255.1)	,-1.3)	,3308.5)	,0.02)	,4884.6)	,-0.58)	,4768.5)	,-0.6)	,152.4)	,0.17)
	13962.9	-0.04	300.1	-0.02	4168.3	-0.16	1022.3	-0.1	5520.9	0	796.3	0.28	69.2	0.04	125.2	-1.34	2740.2	0.03	2013.3	0.38	1683.5	0.23	334.8	1.24
**Iceland**	(11712.8	(-0.08	(236.9	(-0.03	(3161.7	(-0.24	(756.8	(-0.12	(3551.5	(-0.04	(651.7	(0.27	(30	(0.03	(20.9	(-1.41	(2210.9	(0.03	(1587.1	(0.34	(1261.8	(0.18	(290.5	(1.17
	,16590.5)	,-0.01)	,372.5)	,-0.01)	,5454.6)	,-0.09)	,1359)	,-0.07)	,8213.1)	,0.03)	,949.7)	,0.3)	,125.3)	,0.05)	,269.6)	,-1.27)	,3380.8)	,0.04)	,2480.7)	,0.42)	,2153)	,0.28)	,381.5)	,1.32)
	16282.3	-0.19	291.6	0.36	7754.1	-0.21	454.5	-0.01	4116.8	-0.05	472.9	0.19	22.2	0.03	1401.1	-1.08	2809.2	-0.04	1982.1	-0.45	1834.6	-0.47	150.4	-0.09
**India**	(14591.3	(-0.29	(243	(0.31	(6402.2	(-0.35	(353	(-0.01	(3166.4	(-0.28	(384.4	(0.16	(9.2	(0.01	(765.2	(-1.15	(2246.6	(-0.04	(1487.9	(-0.61	(1333.6	(-0.65	(116.5	(-0.14
	,18231.7)	,-0.09)	,346.3)	,0.41)	,9388)	,-0.08)	,570.6)	,-0.01)	,5312.3)	,0.18)	,562.1)	,0.22)	,41.6)	,0.06)	,2054.1)	,-1.01)	,3515.9)	,-0.03)	,2587.9)	,-0.28)	,2440)	,-0.3)	,189.4)	,-0.04)
	12613.6	0.01	260.4	0.19	4203	0	426.5		4762.5	0.14	456.7	0.27	42.3	0.05	272.3	-1.89	2808.3	0	550.2	0.17	473.3	0.19	77.3	0.06
**Indonesia**	(11194.3	(-0.03	(219.8	(0.15	(3435.8	(-0.04	(330.2	0 (0,0)	(3677.2	(0.07	(372.2	(0.25	(18.9	(0.04	(80.8	(-2.14	(2245.7	(0	(418.8	(0.12	(341.9	(0.13	(58.2	(0.04
	,14287.4)	,0.04)	,309.3)	,0.23)	,5090.9)	,0.05)	,537.2)		,6271.2)	,0.21)	,546.9)	,0.3)	,73.7)	,0.07)	,460)	,-1.65)	,3515.3)	,0.01)	,707.4)	,0.23)	,628.6)	,0.26)	,100.3)	,0.07)
**Iran (Islamic Republic of)**	17317.3	0.18	226.3	0.16	6357.1	0.34	807.6	0.01	6953	0.37	591.8	0.24	259.9	-0.16	506	-1.89	2825.9	-0.04	468	-0.47	310.7	-0.5	157.7	-0.4
	(15325.9	(0.14	(190.3	(0.15	(4961.4	(0.29	(641.1	(0.01	(5375.3	(0.28	(484	(0.21	(151.1	(-0.19	(195.8	(-2.06	(2259.7	(-0.05	(375.6	(-0.58	(223.1	(-0.55	(127.5	(-0.64

	,19714.9)	,0.22)	,267)	,0.18)	,8018.8)	,0.39)	,1002.7)	,0.02)	,9038.2)	,0.46)	,708.5)	,0.27)	,407.6)	,-0.13)	,802.5)	,-1.71)	,3536.9)	,-0.03)	,576.3)	,-0.35)	,412.4)	,-0.44)	,194)	,-0.16)
	14043	0.04	220.6	0.19	5191.3	0.12	688.1		4979.4	0.13	503.7	0.32	113.6	0.04	423.4	-1.48	2714.2	0	422.7	-0.08	318.7	-0.14	104.4	0.13
**Iraq**	(11750.3	(-0.03	(175.6	(0.15	(3833.8	(0.05	(502.2	0 (0,0)	(3092.4	(-0.05	(410.7	(0.3	(63.4	(0.03	(134.3	(-1.78	(2168.5	(0	(307.5	(-0.11	(207.2	(-0.18	(82.3	(0.11
	,16923.4)	,0.11)	,275.2)	,0.23)	,7027.7)	,0.18)	,924.9)		,7428.8)	,0.3)	,611.2)	,0.35)	,180.1)	,0.06)	,712.2)	,-1.17)	,3380.9)	,0.01)	,570.6)	,-0.04)	,465)	,-0.1)	,130.6)	,0.16)
	16411.4	0.18	355.4	0.19	5300.4	0.53	850.6	0.08	7224.5	0.04	811.9	0.47	86.3	0.04	106.2	-2.99	2732	0.04	2355.6	0.42	2102.5	0.35	258.7	1.07
**Ireland**	(13590.6	(0.15	(278.5	(0.16	(3891.4	(0.42	(645.1	(0.06	(4657	(-0.03	(659.3	(0.44	(43.9	(0.03	(14.6	(-3.23	(2204	(0.04	(1757.8	(0.23	(1502.2	(0.14	(217.7	(1.03
	,19840.9)	,0.21)	,447.3)	,0.23)	,7125.5)	,0.64)	,1114.9)	,0.1)	,10753.2)	,0.11)	,975.9)	,0.51)	,143.5)	,0.06)	,238.6)	,-2.75)	,3370.8)	,0.05)	,3063.2)	,0.61)	,2815.4)	,0.57)	,300.3)	,1.11)
	15168	0.09	292.9	-0.02	6684.7	-0.18	1203.7	0.06	4345.8	0.71	571.7	0.24	65	0.1	194.5	-0.99	2713	0.02	623	-0.93	466.4	-0.9	157.5	-1.02
**Israel**	(12926.4	(0.01	(230.9	(-0.04	(4998.4	(-0.23	(886.4	(0.04	(2776.5	(0.43	(467.7	(0.22	(31.1	(0.09	(48.9	(-1.03	(2188	(0.02	(469.6	(-1	(315.6	(-0.98	(129.4	(-1.23
	,17909.6)	,0.17)	,367.3)	,-0.01)	,8884.2)	,-0.12)	,1622.3)	,0.07)	,6623.4)	,0.98)	,687.9)	,0.25)	,110.1)	,0.11)	,356.4)	,-0.95)	,3347.5)	,0.02)	,792.9)	,-0.86)	,634.9)	,-0.82)	,189.9)	,-0.82)
	15949.2	-0.02	281.2	-0.04	5687.6	-0.26	1066.9	0.13	6257.9	0.14	734.4	0.32	35.2	0.06	156.6	-0.78	2745.2	0.04	1245.1	-0.21	1051.7	-0.27	195.6	0.16
**Italy**	(14112.7	(-0.08	(235.9	(-0.04	(4531.6	(-0.33	(850.4	(0.1	(4832.9	(-0.02	(603.5	(0.28	(15.3	(0.05	(31.4	(-0.89	(2217.7	(0.04	(963.7	(-0.32	(774.3	(-0.38	(160.4	(0
	,18017.2)	,0.03)	,333.5)	,-0.03)	,7010.6)	,-0.19)	,1328.8)	,0.16)	,8074.2)	,0.29)	,878.3)	,0.35)	,63.5)	,0.07)	,310.6)	,-0.68)	,3385.3)	,0.04)	,1582.9)	,-0.09)	,1390.9)	,-0.16)	,237.2)	,0.32)
	13985.5	0.11	232.7	0.05	5182.4	0.14	823.6		4928.9	0.17	499.7	0.14	230.5	0.07	151.4	-0.17	2716.2	0.02	1261.4	-0.29	1097.2	-0.33	166.4	-0.04
**Jamaica**	(11704.7	(0.06	(182	(0.04	(3867	(0.05	(600.3	0 (0,0)	(3093.9	(0.09	(407.3	(0.11	(130.9	(0.05	(18.9	(-0.22	(2170.2	(0.01	(893.6	(-0.37	(734.4	(-0.42	(128.3	(-0.08
	,16869.7)	,0.15)	,293.1)	,0.06)	,7008.6)	,0.22)	,1109.1)		,7537.9)	,0.25)	,599.3)	,0.17)	,359.2)	,0.09)	,307.1)	,-0.12)	,3383.3)	,0.02)	,1723.7)	,-0.21)	,1564.4)	,-0.24)	,211.5)	,0.01)
	10148.7	0.07	264.1	0.17	2935.9	0.18	629.3	0	2689.3	-0.13	1234.9	0.49	44.3	0.07	16.7	-4.55	2752.8	0.04	605.7	-0.22	456.1	-0.24	150.4	-0.14
**Japan**	(9157.5	(0.04	(222.4	(0.06	(2428.9	(0.1	(496.6	(-0.01	(2054.4	(-0.22	(1020.3	(0.45	(19.8	(0.07	(0.1	(-4.89	(2223.4	(0.04	(480	(-0.29	(333.6	(-0.32	(119.2	(-0.22
	,11238.1)	,0.1)	,311.8)	,0.27)	,3523.5)	,0.25)	,791)	,0.01)	,3487.2)	,-0.03)	,1468.8)	,0.53)	,79.7)	,0.08)	,93.1)	,-4.21)	,3394)	,0.05)	,758)	,-0.15)	,606.5)	,-0.17)	,188.3)	,-0.07)
	14614.2	-0.13	220.1	0.1	5794.4	-0.31	747		4695.3	0.11	605.2	0.34	101.4	-0.03	564.3	-1.52	2751.5	0.01	420	-0.24	326.1	-0.31	94.2	-0.03
**Jordan**	(12255.8	(-0.16	(174.6	(0.08	(4277.7	(-0.36	(548.5	0 (0,0)	(2939.4	(0.06	(491.2	(0.32	(56.2	(-0.04	(194.3	(-1.74	(2198.5	(0	(301.4	(-0.31	(210.6	(-0.37	(70.7	(-0.1
	,17531)	,-0.1)	,275.3)	,0.12)	,7687.5)	,-0.26)	,1002.2)		,7017.3)	,0.17)	,728.8)	,0.36)	,159.9)	,-0.01)	,922.2)	,-1.3)	,3428.5)	,0.02)	,567.6)	,-0.18)	,473.7)	,-0.24)	,123.1)	,0.04)
	13534.9	-0.22	213	0.26	7230.5	-0.45	548.2		2763	0.01	621.8	0.4	60.9	0.03	115.5	-1.47	2633.1	0.04	4629.3	0.03	4348.5	-0.04	294.7	1.51
**Kazakhstan**	(11395.2	(-0.26	(169.8	(0.2	(5324.1	(-0.52	(397.7	0 (0,0)	(1746.5	(-0.05	(505.8	(0.35	(29.5	(0.01	(17.6	(-1.89	(2103.7	(0.03	(3606.8	(-0.38	(3329.4	(-0.48	(250.9	(1.44
	,16097.9)	,-0.18)	,266.2)	,0.31)	,9584.1)	,-0.38)	,738.5)		,4238.4)	,0.06)	,751.3)	,0.44)	,100.9)	,0.06)	,251.4)	,-1.04)	,3277.7)	,0.04)	,6078.3)	,0.45)	,5803.6)	,0.4)	,341.4)	,1.59)
	18029.9	-0.09	194.7	0.08	10991.3	-0.22	668.9		3743.1	0.22	589.6	0.01	17.6	0.01	120.6	0.21	2784.1	-0.04	1203.1	0.07	1113	0.05	91.1	0.28
**Kenya**	(15931.4	(-0.14	(161	(0.06	(9022.2	(-0.3	(525.8	0 (0,0)	(2876.6	(0.17	(481	(-0.03	(6.3	(0	(25.8	(0	(2226.6	(-0.04	(925.4	(-0.08	(834.8	(-0.11	(71.5	(0.26
	,20405.2)	,-0.04)	,233.4)	,0.1)	,13338.2)	,-0.14)	,834.4)		,4829.3)	,0.27)	,700.8)	,0.05)	,33.9)	,0.03)	,287.9)	,0.42)	,3483)	,-0.03)	,1543.4)	,0.22)	,1455.2)	,0.22)	,114.6)	,0.3)
	13384.1	0.03	229.2	-0.14	4874.9	-0.08	350.5		5229.9	0.14	409.8	0.01	66.1	-0.07	256.6	1.37	2647.8	-0.03	691.8	-0.33	553.7	-0.42	138.8	0.05
**Kiribati**	(11201	(0.01	(179.5	(-0.15	(3689.4	(-0.11	(248.2	0 (0,0)	(3352.9	(0.1	(327.8	(0	(32.5	(-0.09	(69.6	(1.25	(2114.8	(-0.04	(513.4	(-0.36	(378.7	(-0.46	(106.9	(0.03
	,16207.9)	,0.05)	,286.9)	,-0.12)	,6328.2)	,-0.05)	,478.5)		,7799.9)	,0.17)	,496.6)	,0.03)	,111.1)	,-0.05)	,479.5)	,1.49)	,3296.4)	,-0.03)	,918.7)	,-0.29)	,781.8)	,-0.37)	,177.5)	,0.07)
	13796.6	0.1	250.9	0.06	5528.8	0.21	755.1	0.01	4144.8	0.15	679.9	0.17	106	-0.13	279.3	-0.83	2813.3	-0.02	494.9	0.07	366.3	-0.17	129.1	0.88
**Kuwait**	(11757.2	(0.08	(197.8	(0.04	(4028.8	(0.17	(556.3	(0.01	(2586.5	(0.1	(552.3	(0.14	(58.5	(-0.17	(51.2	(-1.01	(2248.9	(-0.03	(364.4	(0.01	(237.6	(-0.29	(102.4	(0.75
	,16280.3)	,0.13)	,312.1)	,0.09)	,7467.4)	,0.24)	,1008.2)	,0.01)	,6139.4)	,0.21)	,818.2)	,0.2)	,167.5)	,-0.1)	,503.5)	,-0.65)	,3504.1)	,0)	,660.4)	,0.13)	,531.2)	,-0.06)	,159.4)	,1.01)
	14177.6	-0.17	190.4	-0.01	7748	-0.39	551.3	0	2752.3	0.03	615.5	0.32	61.5	0.02	315.1	0.36	2656.7	0.04	3993.5	-0.99	3835.7	-1.01	165.6	-0.31
**Kyrgyzstan**	(11929.1	(-0.22	(150	(-0.06	(5698.2	(-0.47	(401	(-0.01	(1718.1	(-0.06	(501.8	(0.31	(29.8	(0	(80.4	(-0.25	(2122.4	(0.03	(3044.1	(-1.31	(2877.2	(-1.35	(135.3	(-0.47
	,16754.7)	,-0.12)	,240.1)	,0.05)	,10296.9)	,-0.31)	,745.6)	,0)	,4283.6)	,0.11)	,742.8)	,0.33)	,102)	,0.05)	,551.2)	,0.98)	,3306.8)	,0.05)	,5295.2)	,-0.66)	,5139)	,-0.68)	,201.4)	,-0.14)
	14009.5	-0.05	249.3	0.33	4471.3	-0.13	420		6099.4	0.16	462.4	0.37	50.9	0.01	284.8	-2.94	2713	0.01	1038.6	-0.66	945.2	-0.72	94.3	-0.08
**Lao People's Democratic Republic**	(11515	(-0.08	(196.1	(0.28	(3378.3	(-0.16	(301.1	0 (0,0)	(3905.8	(0.11	(374.6	(0.35	(23.8	(0	(93.6	(-3.17	(2167.4	(0	(737.2	(-0.72	(644	(-0.79	(69.3	(-0.12
	,17103)	,-0.02)	,309.6)	,0.37)	,5829.9)	,-0.09)	,569.8)		,9337)	,0.21)	,557)	,0.4)	,88.3)	,0.03)	,477.2)	,-2.71)	,3379.6)	,0.01)	,1408.5)	,-0.6)	,1316.9)	,-0.65)	,124.4)	,-0.05)

	17014.3	-0.28	204.3	0.22	8496.2	-0.64	556.1	-0.01	5354.6	0.06	668	0.27	60.6	0.08	115.9	-1.57	2628	0.03	4015.4	0.26	3810	0.28	215.3	-0.03
**Latvia**	(14179.1	(-0.37	(162.4	(0.19	(6088.9	(-0.79	(405.5	(-0.01	(3273.2	(-0.03	(541.8	(0.25	(29.4	(0.06	(15.1	(-1.89	(2099.8	(0.03	(2933.8	(0.12	(2713.9	(0.14	(177.8	(-0.24
	,20396.3)	,-0.19)	,256.1)	,0.26)	,11337.4)	,-0.49)	,740.7)	,-0.01)	,7989.9)	,0.16)	,802)	,0.29)	,100.5)	,0.09)	,250.2)	,-1.24)	,3271.1)	,0.04)	,5167.6)	,0.41)	,4967.5)	,0.42)	,255.9)	,0.17)
	17257.5	0.12	223.6	0.09	7005.8	0.34	874.1	0.01	6398.2	0.09	649.8	0.26	96.3	-0.15	511.2	-1.32	2697.1	-0.03	492.5	-0.03	352.9	-0.34	140.2	0.91
**Lebanon**	(14379.6	(0.04	(178.2	(0.08	(5055.3	(0.21	(659.9	(0.01	(4087	(-0.04	(528.7	(0.24	(53.4	(-0.15	(177	(-1.47	(2155.9	(-0.04	(368.7	(-0.06	(233	(-0.38	(112.7	(0.88
	,21226.9)	,0.19)	,278.7)	,0.11)	,9673.8)	,0.47)	,1144.1)	,0.01)	,9774.9)	,0.22)	,780.8)	,0.29)	,152.2)	,-0.14)	,827.6)	,-1.18)	,3356.7)	,-0.02)	,647.9)	,0)	,507.9)	,-0.3)	,170.7)	,0.94)
	19368	0.13	190.6	0.2	12173.6	0.19	601.2		4371.5	0.13	517.2	0.04	21.4	0.11	107.8	-1.37	2603.8	0.02	2348.1	0.58	2155.4	0.57	198.3	0.68
**Lesotho**	(15742.4	(0.08	(147.2	(0.19	(8723.4	(0.12	(433.9	0 (0,0)	(2745.2	(0.04	(416.7	(-0.02	(8.6	(0.07	(17.9	(-1.46	(2081.5	(0	(1681.4	(0.5	(1484.1	(0.49	(164.5	(0.62
	,23912.8)	,0.18)	,242.4)	,0.21)	,16603.8)	,0.25)	,810.8)		,6843.1)	,0.21)	,625.1)	,0.1)	,39.9)	,0.15)	,265.8)	,-1.28)	,3240.6)	,0.03)	,3159.7)	,0.65)	,2968.2)	,0.64)	,233.8)	,0.74)
	16759.6	0.2	191.8	0.09	10216.4	0.28	567.3		3138.1	0.16	614	0.25	23.9	-0.02	185.6	1.53	2747	-0.03	946.6	-0.04	848.3	-0.04	99.1	-0.02
**Liberia**	(13874.2	(0.18	(147.5	(0.05	(7521.6	(0.24	(407.1	0 (0,0)	(1948.5	(0.13	(496	(0.24	(9.6	(-0.05	(29.8	(1.1	(2194.8	(-0.04	(702.6	(-0.11	(606.2	(-0.12	(77.8	(-0.03
	,20176)	,0.23)	,245.5)	,0.13)	,13584)	,0.33)	,759.8)		,4765.4)	,0.19)	,738.5)	,0.26)	,44.9)	,0.01)	,420.7)	,1.95)	,3423.2)	,-0.03)	,1249.3)	,0.03)	,1153.2)	,0.04)	,123.8)	,0)
	15231.1	0.16	221.2	-0.18	6213.3	0.23	740.9	0.01	5008.6	0.17	556.3	0.15	99.6	-0.1	588.8	1.15	2732.2	-0.02	494.3	0.05	354.2	-0.27	140.5	1.02
**Libya**	(12724.7	(0.13	(175.5	(-0.23	(4516.5	(0.2	(542.6	(0.01	(3247.3	(0.1	(451.1	(0.11	(55.2	(-0.12	(253.7	(0.76	(2183	(-0.03	(368	(0	(231.5	(-0.34	(115.2	(0.97
	,18591.9)	,0.2)	,276)	,-0.13)	,8291.2)	,0.26)	,995.1)	,0.01)	,7543.7)	,0.24)	,672)	,0.2)	,157.2)	,-0.07)	,932.7)	,1.54)	,3403)	,-0.01)	,648.5)	,0.1)	,507.7)	,-0.21)	,170.1)	,1.06)
	17852.8	-0.06	207.2	0.25	8606.2	-0.18	558		6205.3	0.09	670.6	0.16	60.6	0.05	106.4	-1.92	2631.4	0.01	3603.6	-0.78	3418.3	-0.84	194	0.81
**Lithuania**	(14615.4	(-0.12	(163	(0.23	(6263.9	(-0.27	(406.5	0 (0,0)	(3971.6	(0	(546	(0.15	(29.4	(0.04	(13.9	(-2.16	(2102.5	(0.01	(2728.6	(-0.85	(2538.5	(-0.92	(163.1	(0.55
	,21656.6)	,0)	,259.4)	,0.28)	,11760.8)	,-0.1)	,748.7)		,9351)	,0.19)	,805.6)	,0.18)	,100.5)	,0.06)	,231.3)	,-1.67)	,3275.2)	,0.01)	,4580.9)	,-0.7)	,4393.2)	,-0.76)	,229.5)	,1.06)
	14746.6	-0.16	308	0.02	4848.9	-0.5	1046.3	0.05	5856.1	-0.07	665.6	0.47	58.8	0.12	95.1	-1.85	2729.4	0.09	2332	0.22	2088.7	0.18	248.5	0.56
**Luxembourg**	(12249.7	(-0.2	(244.2	(0.01	(3647	(-0.58	(777.2	(0.04	(3760.9	(-0.14	(543.5	(0.44	(26.8	(0.11	(15.8	(-1.99	(2202	(0.08	(1767.7	(0.14	(1514.7	(0.1	(208.5	(0.43
	,17793)	,-0.11)	,382.6)	,0.03)	,6363.4)	,-0.42)	,1393.8)	,0.07)	,8998.5)	,-0.01)	,801.3)	,0.49)	,100.8)	,0.13)	,205)	,-1.71)	,3367.8)	,0.09)	,2952.7)	,0.29)	,2710.1)	,0.25)	,293.6)	,0.69)
	17817.6	0.04	188.4	0.05	10571.8	-0.02	647.3		4017	0.24	580.6	0.08	22.9	-0.01	152.8	0.71	2716.2	-0.03	2096.2	-0.11	1999.2	-0.12	99.1	0.09
**Madagascar**	(14653.4	(0.01	(146	(0.03	(7767.1	(-0.06	(471.8	0 (0,0)	(2546.9	(0.19	(468.3	(0.05	(9.2	(-0.03	(23.5	(0.61	(2170.1	(-0.04	(1518.1	(-0.18	(1420.4	(-0.19	(77.2	(0.06
	,21748.6)	,0.07)	,240.5)	,0.07)	,14093.3)	,0.02)	,869.3)		,6262.8)	,0.29)	,701.5)	,0.1)	,42.1)	,0.01)	,363.7)	,0.82)	,3383.4)	,-0.02)	,2811.8)	,-0.04)	,2709.6)	,-0.05)	,125.5)	,0.12)
	16430.9	0	179.7	0.07	9072.8	-0.11	646.6		4074.2	0.25	562.2	0.15	22.3	-0.05	160.5	1.06	2668.1	-0.04	2166.7	0.03	2067.4	0.02	101.6	0.13
**Malawi**	(13834.4	(-0.04	(139	(0.06	(6737.9	(-0.17	(470	0 (0,0)	(2595.2	(0.2	(451.9	(0.13	(9	(-0.06	(27.7	(0.93	(2131.4	(-0.04	(1624	(-0.04	(1523.9	(-0.04	(79.1	(0.12
	,19829)	,0.04)	,228.9)	,0.09)	,11939)	,-0.05)	,873.1)		,6376.1)	,0.3)	,675)	,0.16)	,41.2)	,-0.04)	,378)	,1.2)	,3321.6)	,-0.04)	,2855.5)	,0.09)	,2753.8)	,0.09)	,127.6)	,0.14)
	15337.9	-0.01	284.9	0.23	6006	-0.12	413.7		6084.4	0.11	552.6	0.36	20	0.04	136	-2.25	2729.4	0.03	626.4	-0.51	524.8	-0.6	102.2	0.01
**Malaysia**	(12738.7	(-0.08	(227.9	(0.21	(4528.5	(-0.24	(297.5	0 (0,0)	(3877	(0.02	(447.4	(0.34	(8.1	(0.02	(23.4	(-2.4	(2180.7	(0.02	(460.4	(-0.54	(360.8	(-0.64	(78.2	(-0.04
	,18626.3)	,0.06)	,355.3)	,0.25)	,7796.8)	,0)	,564.9)		,9173.4)	,0.2)	,671.7)	,0.38)	,37.1)	,0.06)	,277.3)	,-2.09)	,3399.4)	,0.03)	,836.2)	,-0.47)	,731.6)	,-0.56)	,131.4)	,0.06)
	13673.6	-0.07	279.8	0.18	5440.9	-0.29	417.7	-0.01	4683.1	0.32	574.4	0.28	52.3	-0.16	188.6	-2.53	2757.7	-0.1	693.9	-0.64	592.1	-0.75	102.4	0.08
**Maldives**	(11625.1	(-0.1	(222.2	(0.16	(4092.9	(-0.34	(299.4	(-0.01	(3008.3	(0.27	(464.9	(0.26	(24.4	(-0.19	(42.1	(-2.58	(2203.8	(-0.11	(491.9	(-0.68	(391.3	(-0.8	(77.1	(0.04
	,16258.4)	,-0.04)	,347.5)	,0.2)	,7104.6)	,-0.25)	,563.9)	,-0.01)	,6975.2)	,0.37)	,694.8)	,0.3)	,90.7)	,-0.12)	,355.7)	,-2.48)	,3435.8)	,-0.1)	,957.9)	,-0.6)	,857.7)	,-0.71)	,132.3)	,0.11)
	13870.7	0.04	198.9	0.18	7557.3	0.02	568		2727.4	0.11	594.9	0.21	23.8	0.02	123.1	0.39	2750.9	0.01	876.4	0.02	792.1	0.04	84.9	-0.11
**Mali**	(11704	(0.01	(156.2	(0.17	(5547.9	(-0.04	(413.1	0 (0,0)	(1689.8	(0.06	(482.2	(0.2	(9.5	(0.02	(20.2	(0.18	(2198.4	(0.01	(639.2	(-0.06	(556.5	(-0.05	(66.5	(-0.14
	,16556.6)	,0.08)	,252.6)	,0.19)	,10082.2)	,0.08)	,763.1)		,4149)	,0.17)	,718.6)	,0.23)	,44.7)	,0.03)	,309.1)	,0.6)	,3426.6)	,0.02)	,1174.7)	,0.1)	,1093.1)	,0.13)	,106.5)	,-0.08)
	14980.1	-0.02	296.3	0.04	4490.7	-0.14	1055.1	0.07	6414.9	0.02	656.6	0.34	58.4	0.14	166.9	-1.8	2725.2	0.05	1182.9	0.07	1024.5	0.02	160.2	0.36
**Malta**	(12495.1	(-0.06	(234.7	(0.03	(3367.5	(-0.2	(782.2	(0.05	(4118.4	(-0.02	(537.6	(0.33	(26.6	(0.13	(35.1	(-1.86	(2198.5	(0.04	(851.4	(-0.04	(696.2	(-0.09	(131.6	(0.24
	,18326.6)	,0.01)	,371.9)	,0.05)	,5963.9)	,-0.07)	,1406.1)	,0.09)	,9662.5)	,0.07)	,792)	,0.35)	,100.2)	,0.15)	,324.1)	,-1.73)	,3362.7)	,0.06)	,1597.3)	,0.18)	,1436.4)	,0.13)	,193.3)	,0.48)
**Marshall Islands**	13096	-0.01	243.5	-0.05	4736.8	-0.08	351.2	0.01	4901.9	-0.03	471.5	0.23	69.1	0.04	238.2	0.24	2742.5	0.06	1012.4	0.06	865.9	0.06	147.7	0.08
	(11078.4	(-0.03	(192.6	(-0.06	(3525.7	(-0.1	(251.3	(0.01	(3142.2	(-0.08	(381.5	(0.22	(33.7	(0.01	(60.2	(0.18	(2192	(0.05	(752.2	(0.02	(607.9	(0.01	(114.4	(0.06

	,15743.6)	,0.01)	,307.3)	,-0.05)	,6222.1)	,-0.06)	,476.5)	,0.01)	,7427)	,0.02)	,567.5)	,0.25)	,115.7)	,0.07)	,433.8)	,0.29)	,3414.7)	,0.07)	,1329.3)	,0.1)	,1179.8)	,0.1)	,186.8)	,0.09)
	14520.7	0.02	209.8	0.12	8233.5	-0.01	568.2		2724.8	-0.01	642.7	0.41	23.5	0.03	99.5	0.46	2740.9	0.04	829.5	-0.23	729.7	-0.3	100.5	0.25
**Mauritania**	(12216	(-0.02	(165.4	(0.1	(6046.7	(-0.07	(410.9	0 (0,0)	(1746.5	(-0.05	(520.4	(0.4	(9.4	(0.02	(15.2	(0.39	(2190.4	(0.04	(602.3	(-0.3	(502.5	(-0.38	(80.4	(0.24
	,17455.3)	,0.06)	,263.9)	,0.14)	,11097.7)	,0.05)	,762.2)		,4190.8)	,0.03)	,775.9)	,0.42)	,44.2)	,0.04)	,267.4)	,0.54)	,3413.6)	,0.05)	,1118.7)	,-0.16)	,1015.4)	,-0.22)	,123.6)	,0.27)
	14590.8	-0.13	277.2	0.18	6326.3	-0.31	413		4936.2	0.05	538.4	0.18	50.4	0.01	169.4	-1.61	2690	0.01	1603.8	0	1457.7	0.05	148.5	-0.43
**Mauritius**	(12316.4	(-0.19	(222.3	(0.16	(4763.3	(-0.47	(296.5	0 (0,0)	(3135.2	(-0.01	(438.7	(0.16	(23.5	(0	(38.8	(-1.74	(2148.9	(0.01	(1208	(-0.07	(1057.9	(-0.03	(113.6	(-0.48
	,17393.5)	,-0.06)	,341.4)	,0.2)	,8348.8)	,-0.16)	,563.3)		,7225.5)	,0.1)	,649.3)	,0.2)	,87.3)	,0.03)	,321.9)	,-1.48)	,3350.3)	,0.02)	,2102.8)	,0.07)	,1959.3)	,0.13)	,189.8)	,-0.38)
	14206.8	0.03	247.4	0.08	6334.8	-0.04	839.8		4078.5	0.18	530.8	0.2	50.8	-0.01	102.4	-0.58	2799.1	-0.02	1784.2	-1.41	1626.7	-1.45	160.5	-0.92
**Mexico**	(12719.8	(-0.03	(207.2	(0.08	(5150.6	(-0.17	(665.2	0 (0,0)	(3131.1	(0.07	(433.4	(0.18	(23.2	(-0.01	(16.4	(-0.67	(2238.8	(-0.02	(1352.7	(-1.56	(1198	(-1.62	(130.1	(-1.26
	,15935.9)	,0.09)	,294.4)	,0.09)	,7665.8)	,0.09)	,1045.2)		,5332.6)	,0.29)	,634.2)	,0.22)	,86.6)	,0)	,215.8)	,-0.5)	,3501.4)	,-0.02)	,2289.4)	,-1.25)	,2134.8)	,-1.28)	,196.6)	,-0.59)
	13293.3	0.03	240.9	-0.08	4853	-0.04	352.1		5050.1	0.13	466.1	0.13	68.8	-0.02	243.3	0.32	2695	-0.03	961.8	-0.1	815.8	-0.11	147.2	-0.01
**Micronesia (Federated States of)**	(11234	(0.02	(188.8	(-0.08	(3679.9	(-0.05	(252.1	0 (0,0)	(3250.4	(0.1	(377.3	(0.1	(33.8	(-0.05	(62.8	(0.28	(2152.5	(-0.04	(711.7	(-0.13	(572.3	(-0.15	(113.3	(-0.03
	,15814.9)	,0.05)	,300.3)	,-0.07)	,6405.3)	,-0.02)	,482.5)		,7583.1)	,0.16)	,558.6)	,0.15)	,115.1)	,0.01)	,441.3)	,0.36)	,3357.7)	,-0.02)	,1291.6)	,-0.07)	,1140.9)	,-0.08)	,186.3)	,0)
	15810.9	0.03	337.4	-0.01	5870.4	0.02	1043.6	0.07	6067.1	0.03	659.3	0.21	58.3	0.05	38.4	-1.43	2724.5	0.04	1127.3	-0.2	948.3	-0.26	180.8	0.14
**Monaco**	(13149.8	(0	(267.3	(-0.02	(4224.5	(-0.02	(769.7	(0.05	(3827.6	(-0.04	(534.1	(0.19	(26.6	(0.03	(4.8	(-1.48	(2197.9	(0.03	(800.6	(-0.34	(625.1	(-0.43	(146.4	(0.1
	,19227.9)	,0.06)	,422.9)	,0.01)	,8015.6)	,0.05)	,1379.1)	,0.08)	,9181.2)	,0.09)	,795.1)	,0.24)	,100)	,0.06)	,98.3)	,-1.37)	,3362)	,0.04)	,1554)	,-0.05)	,1369.4)	,-0.09)	,222.4)	,0.18)
	14114.8	0.02	202	0.32	7946.7	0.08	547.2	0.01	2661.6	-0.04	576.2	0.3	60.7	-0.14	158.7	-2.08	2651.5	-0.03	3961.8	1.35	3816.6	1.41	151.7	0.06
**Mongolia**	(11860	(-0.01	(160.1	(0.28	(5713.3	(0.05	(393.1	(0	(1655.8	(-0.16	(470.7	(0.27	(29.4	(-0.16	(22.7	(-2.46	(2118.8	(-0.03	(2742	(0.93	(2591.9	(0.98	(117.9	(-0.02
	,16932.7)	,0.04)	,254)	,0.36)	,10554.3)	,0.11)	,738)	,0.01)	,4055.6)	,0.08)	,698.3)	,0.33)	,100.7)	,-0.11)	,323.9)	,-1.7)	,3300.1)	,-0.03)	,5380.5)	,1.76)	,5238.1)	,1.84)	,189.7)	,0.13)
	14900.5	0.02	215.6	0.13	6097.9	-0.02	545.9		5266.2	0.09	738	0.24	63.2	0.04	158.6	-1.15	2686.7	0.02	2467.2	-0.2	2372.2	-0.2	97.4	-0.02
**Montenegro**	(12556.2	(-0.04	(170.8	(0.1	(4486.9	(-0.09	(397.1	0 (0,0)	(3317.7	(-0.02	(603.1	(0.22	(30.7	(0.02	(25	(-1.47	(2145.9	(0.02	(1701.9	(-0.32	(1609.9	(-0.33	(74.6	(-0.05
	,17780)	,0.08)	,269.1)	,0.16)	,8128.7)	,0.04)	,730.7)		,7994.5)	,0.19)	,881.6)	,0.27)	,104.8)	,0.06)	,324.7)	,-0.83)	,3346.6)	,0.02)	,3367.3)	,-0.08)	,3281.1)	,-0.08)	,124.3)	,0)
	16232.3	0.02	213.1	0.11	7599.9	0.03	736.2		4732.7	0.17	538.2	0.23	100.3	0.04	615	-1.09	2732.8	-0.01	495	0.07	374	-0.05	121.5	0.45
**Morocco**	(13605.8	(-0.02	(168.7	(0.1	(5550.5	(-0.01	(540.8	0 (0,0)	(3002.2	(0.09	(443.4	(0.2	(55.5	(0.01	(256.1	(-1.18	(2183.7	(-0.01	(370.4	(0.04	(252.8	(-0.08	(97.1	(0.43
	,19496.1)	,0.05)	,266.5)	,0.12)	,10305.3)	,0.07)	,982.9)		,7223.4)	,0.25)	,644.3)	,0.26)	,158.2)	,0.06)	,959.2)	,-0.99)	,3404.6)	,0)	,651.4)	,0.1)	,530.9)	,-0.01)	,150.2)	,0.46)
	18387.4	0.16	181.4	0.3	11149	0.24	645.1		4176	0.23	549.2	0.01	22.5	-0.05	123.9	-1.23	2671.5	-0.05	2413.2	0.52	2296.1	0.51	120.3	0.72
**Mozambique**	(15279.7	(0.14	(139.8	(0.28	(8096.6	(0.22	(469.8	0 (0,0)	(2627.1	(0.18	(440.5	(-0.01	(9.1	(-0.06	(27.6	(-1.5	(2133.9	(-0.06	(1811.4	(0.43	(1697.2	(0.42	(97.6	(0.67
	,22344.4)	,0.19)	,234.6)	,0.32)	,14983.5)	,0.27)	,862)		,6237.7)	,0.28)	,663.3)	,0.03)	,41.5)	,-0.05)	,319.1)	,-0.97)	,3326.6)	,-0.05)	,3116.2)	,0.6)	,3000.2)	,0.59)	,147.2)	,0.76)
	12604.5	-0.04	243.8	0.4	3919.9	0.07	410.1		5188.5	0.2	456.3	0.27	49.1	-0.09	278	-3.71	2666.3	-0.04	849.2	-0.56	749.6	-0.6	100.4	-0.22
**Myanmar**	(10458.9	(-0.08	(195	(0.35	(2924.3	(0.01	(289.5	0 (0,0)	(3340.7	(0.13	(373	(0.25	(23	(-0.09	(81.2	(-4.06	(2130.6	(-0.04	(616.9	(-0.59	(522.7	(-0.64	(74.5	(-0.25
	,15197.8)	,0)	,303.8)	,0.45)	,5132.5)	,0.12)	,559.3)		,7843.4)	,0.27)	,554.4)	,0.29)	,85.2)	,-0.09)	,482.6)	,-3.35)	,3318.7)	,-0.04)	,1163.2)	,-0.52)	,1067.2)	,-0.56)	,131.3)	,-0.2)
	15009.1	0.05	212.3	0.2	7440	-0.02	601.1		4277.5	0.25	586.1	0.19	21.9	-0.07	55.2	-0.9	2642.7	-0.03	2478.4	-0.11	2283.5	-0.14	201	0.26
**Namibia**	(12492.4	(0	(167.9	(0.18	(5465.8	(-0.11	(433.4	0 (0,0)	(2643.7	(0.15	(475.1	(0.17	(8.8	(-0.08	(8.8	(-0.98	(2112	(-0.04	(1835.5	(-0.2	(1647.8	(-0.24	(167.8	(0.23
	,18267.2)	,0.1)	,266.5)	,0.22)	,10108.7)	,0.07)	,810.3)		,6617.5)	,0.34)	,710.3)	,0.21)	,40.6)	,-0.06)	,165.7)	,-0.81)	,3288.7)	,-0.03)	,3231)	,-0.02)	,3046.1)	,-0.05)	,239.2)	,0.29)
	13606.7	0.15	265.3	-0.35	5253	0.19	352.1	-0.02	5128.9	0.36	448.5	-0.28	65.9	-0.34	125.5	2.01	2669.5	-0.16	983	-0.54	840.2	-0.63	144	0.07
**Nauru**	(11240	(0.14	(210.7	(-0.47	(3886.4	(0.18	(248.7	(-0.02	(3255.7	(0.34	(362.3	(-0.33	(32.3	(-0.36	(26.5	(1.25	(2133	(-0.17	(730.9	(-0.57	(592	(-0.67	(110.9	(0.06
	,16390.3)	,0.17)	,331)	,-0.22)	,6837.9)	,0.2)	,479.6)	,-0.02)	,7706.7)	,0.39)	,542.8)	,-0.23)	,110.9)	,-0.31)	,258.6)	,2.77)	,3322.7)	,-0.16)	,1265.6)	,-0.5)	,1124.9)	,-0.59)	,184.2)	,0.07)
	18854.6	0.18	271.9	0.05	10950	0.32	475.7		4396	0.31	421.2	0.22	35	-0.1	846.9	-0.98	2712.5	-0.03	1825	0.02	1719.1	0.04	107.9	-0.17
**Nepal**	(15509.9	(0.08	(211.9	(0.04	(7865.6	(0.12	(343.7	0 (0,0)	(2794.5	(0.23	(341.6	(0.2	(15.3	(-0.11	(433.9	(-1.06	(2167.2	(-0.04	(1247.6	(-0.02	(1137.5	(-0.02	(79.5	(-0.22
	,23007.9)	,0.28)	,347)	,0.05)	,14582.9)	,0.52)	,638.9)		,6663.6)	,0.39)	,509.9)	,0.24)	,62.6)	,-0.09)	,1245.3)	,-0.9)	,3377.6)	,-0.03)	,2616.4)	,0.07)	,2513.2)	,0.09)	,141)	,-0.12)

	16163.4	-0.02	401.3	-0.2	4958.6	-0.06	932.8	0.07	7310.6	-0.03	672.2	0.36	67.4	0.13	106.1	-1.69	2729	0.07	1803.4	0.87	1585.4	0.9	221.8	0.68
**Netherlands**	(13458.1	(-0.13	(329.3	(-0.25	(3645.2	(-0.15	(723.5	(0.05	(4640.5	(-0.27	(548.4	(0.34	(31.3	(0.1	(16.5	(-1.81	(2201.5	(0.07	(1390	(0.76	(1171.7	(0.77	(183.7	(0.66
	,19521.3)	,0.1)	,484.8)	,-0.15)	,6782.7)	,0.04)	,1191.1)	,0.08)	,10992)	,0.21)	,809.2)	,0.38)	,115.4)	,0.16)	,228.3)	,-1.58)	,3367.3)	,0.07)	,2268.4)	,0.98)	,2052.1)	,1.02)	,267.5)	,0.7)
	13438.2	-0.02	335.7	0.04	3111.6	-0.33	1124.5	0	5562.6	0.06	934	0.3	102.1	0.09	125.5	2.39	2875.9	0.02	2887.6	1.13	2681.3	1.21	213	0.21
**New Zealand**	(11618.7	(-0.07	(281.9	(0.04	(2466.1	(-0.44	(899.3	(0	(3984.5	(-0.04	(765.4	(0.28	(51.6	(0.05	(22.6	(1.71	(2308.4	(0.02	(2007.4	(0.67	(1787.5	(0.71	(169.1	(0.19
	,15425.1)	,0.04)	,397.1)	,0.05)	,3901.9)	,-0.23)	,1403.1)	,0.01)	,7662.8)	,0.15)	,1122.6)	,0.32)	,171.4)	,0.13)	,272.4)	,3.07)	,3580.4)	,0.02)	,4080.7)	,1.58)	,3867.7)	,1.71)	,264.3)	,0.24)
	14391.9	0.11	232	0.06	5863.2	0.14	753.6		4843.1	0.21	528.5	0.2	101.5	-0.07	188	-0.58	2689	-0.02	3886.2	0.09	3798.4	0.1	92	-0.26
**Nicaragua**	(12130.2	(0.07	(182.2	(0.05	(4264.4	(0.09	(554.4	0 (0,0)	(3102.5	(0.12	(430.5	(0.18	(53.7	(-0.07	(26.6	(-0.76	(2148.7	(-0.02	(2836.7	(-0.14	(2752.8	(-0.14	(70.7	(-0.28
	,17256.3)	,0.15)	,291.3)	,0.07)	,7865.7)	,0.19)	,1002.4)		,7426.8)	,0.29)	,633)	,0.21)	,169.6)	,-0.06)	,361.8)	,-0.39)	,3347.4)	,-0.01)	,5168.9)	,0.32)	,5077.7)	,0.33)	,118.6)	,-0.24)
	15451.7	0.15	190.9	0.07	9186.6	0.19	567.5		2806.5	0.18	574	0.08	23.5	-0.12	166.8	2.93	2728.9	-0.04	902	0.01	817.7	0.03	85	-0.17
**Niger**	(12957.6	(0.14	(147.2	(0.05	(6826.9	(0.17	(411.7	0 (0,0)	(1751.2	(0.14	(464	(0.07	(9.4	(-0.14	(32.1	(2.63	(2180.1	(-0.04	(677.6	(-0.07	(594.8	(-0.06	(65.7	(-0.22
	,18499.6)	,0.16)	,243.4)	,0.1)	,12280.3)	,0.2)	,760.5)		,4333.8)	,0.22)	,690.3)	,0.1)	,44.2)	,-0.1)	,402.8)	,3.23)	,3399.6)	,-0.03)	,1189.5)	,0.09)	,1104.5)	,0.12)	,108.2)	,-0.11)
	14924.6	-0.11	224.8	0.29	8422.2	-0.26	583.8	0	2986.7	0.2	589.9	0.13	17.8	-0.29	79.5	0.06	2795.8	-0.03	1001.3	0.15	900.9	0.1	101.3	0.63
**Nigeria**	(13111.3	(-0.17	(187.6	(0.23	(6747.4	(-0.41	(458.3	(0	(2302.2	(0.04	(480	(0.08	(6.6	(-0.35	(19.4	(-0.14	(2236.5	(-0.06	(769.9	(0.06	(668.9	(-0.01	(83.3	(0.58
	,16914.1)	,-0.05)	,268.8)	,0.36)	,10378)	,-0.12)	,731.2)	,0.01)	,3874.5)	,0.36)	,703.9)	,0.18)	,34.7)	,-0.23)	,205.9)	,0.26)	,3493.9)	,-0.01)	,1290.4)	,0.25)	,1188.8)	,0.2)	,121.6)	,0.69)
	13605.1	0.04	273.3	0.06	5199.8	0.03	355.1		5085.9	0.07	501.2	0.16	69	0	137.8	-0.47	2688.3	0	918.8	-0.08	777.2	-0.09	142.7	-0.03
**Niue**	(11339.3	(0.02	(216.5	(0.05	(3808.3	(0.01	(254.3	0 (0,0)	(3235.9	(0.03	(407.9	(0.14	(34.1	(-0.03	(26.8	(-0.62	(2146.8	(-0.01	(687.3	(-0.14	(542.4	(-0.16	(108	(-0.04
	,16468.3)	,0.05)	,340.3)	,0.08)	,6952.5)	,0.04)	,480.7)		,7671.4)	,0.11)	,602.1)	,0.17)	,115.5)	,0.03)	,278)	,-0.32)	,3350.1)	,0.01)	,1201)	,-0.02)	,1060.3)	,-0.02)	,182.9)	,-0.03)
	14372.3	-0.02	213.2	0.12	5576	-0.18	543.5		5181	0.16	717.8	0.33	64	0.06	185.3	-1.02	2707.3	0	2352.6	-0.59	2254.2	-0.61	100.8	-0.02
**North Macedonia**	(12124.1	(-0.07	(168.2	(0.1	(4190.1	(-0.25	(393.4	0 (0,0)	(3290.7	(0.05	(589	(0.31	(31.2	(0.05	(30.3	(-1.17	(2162.7	(-0.01	(1692.2	(-0.72	(1593.6	(-0.74	(77.6	(-0.08
	,17273.7)	,0.04)	,266.9)	,0.14)	,7363.3)	,-0.12)	,730.3)		,7677.3)	,0.27)	,864.9)	,0.34)	,106.3)	,0.07)	,362.3)	,-0.86)	,3372.9)	,0)	,3241.3)	,-0.46)	,3144.5)	,-0.48)	,128.2)	,0.04)
	13169.1	0.12	300.7	-0.32	4681.9	0.19	357.8		5042.8	0.21	562.4	-0.11	71.9	-0.16	62.7	2.07	2751.5	-0.06	1332.6	-0.1	1154.2	-0.19	180.7	0.57
**Northern Mariana Islands**	(10982.8	(0.09	(240.7	(-0.35	(3578.5	(0.17	(256.6	0 (0,0)	(3227.7	(0.16	(455.7	(-0.15	(35.6	(-0.23	(11.2	(1.73	(2199.5	(-0.07	(1028.5	(-0.14	(858	(-0.24	(143.8	(0.45
	,15893.4)	,0.14)	,373)	,-0.29)	,6124.8)	,0.21)	,484)		,7574.7)	,0.26)	,677.2)	,-0.07)	,120)	,-0.09)	,159.1)	,2.43)	,3427.9)	,-0.05)	,1684.2)	,-0.05)	,1511.8)	,-0.14)	,222.9)	,0.7)
	15975.2	0	302.4	0.03	4726.5	0.18	978.7	0.13	7438.1	-0.15	595.7	0.34	29.7	0.09	114.6	-1.46	2767	0.05	1775.5	-0.19	1487.5	-0.49	292.4	1.92
**Norway**	(14086.2	(-0.02	(253.4	(0.02	(3757.8	(0.11	(768.2	(0.11	(5812.1	(-0.21	(486.9	(0.32	(12	(0.08	(20.9	(-1.57	(2235.1	(0.05	(1389.7	(-0.25	(1097.1	(-0.56	(246.3	(1.61
	,18386.9)	,0.02)	,357.5)	,0.04)	,5822.7)	,0.26)	,1234.6)	,0.16)	,9687)	,-0.09)	,712.2)	,0.36)	,54.7)	,0.1)	,233.9)	,-1.36)	,3410.4)	,0.05)	,2253.4)	,-0.12)	,1967.4)	,-0.43)	,346.2)	,2.23)
	14689.4	0.12	237.9	0.13	6117	0.28	685.6	0.01	4763.7	0.13	601.8	0.25	103	-0.07	292.5	-1.27	2737.8	-0.02	519.3	-0.18	428.2	-0.21	91.5	-0.07
**Oman**	(12291.8	(0.09	(186.2	(0.11	(4514.4	(0.25	(499.6	(0	(2974.7	(0.06	(485.7	(0.21	(57.2	(-0.1	(56.5	(-1.43	(2187.6	(-0.03	(379.4	(-0.3	(290.7	(-0.34	(68.1	(-0.15
	,17729.3)	,0.15)	,299.3)	,0.16)	,8213.4)	,0.31)	,919.2)	,0.01)	,7250.9)	,0.21)	,727.7)	,0.28)	,162.3)	,-0.05)	,522.8)	,-1.11)	,3413.3)	,-0.01)	,698.3)	,-0.06)	,602.5)	,-0.08)	,120.5)	,0.01)
	16596.9	-0.02	283.1	0.05	8244.3	-0.23	490.6		4422.1	0.17	415.7	0.07	29.2	-0.07	902.8	1.32	2851.9	-0.04	2469.1	-0.03	2349.9	-0.02	122.2	-0.11
**Pakistan**	(14419.4	(-0.06	(236.1	(0.04	(6427.1	(-0.3	(381.1	0 (0,0)	(3279.3	(0.12	(336.4	(0.04	(12	(-0.08	(448.5	(1.24	(2280.3	(-0.04	(1855.1	(-0.05	(1731.3	(-0.05	(93.9	(-0.22
	,19017.8)	,0.03)	,337.6)	,0.06)	,10417.1)	,-0.15)	,617.2)		,5988.7)	,0.22)	,499.3)	,0.09)	,53)	,-0.06)	,1371.1)	,1.4)	,3570.3)	,-0.04)	,3193.1)	,0)	,3080.9)	,0.01)	,156.9)	,-0.01)
	13386	0.03	278.1	-0.01	5112	0.02	354		4882.4	0.05	523.3	0.13	69	-0.01	112	0.17	2740.4	0.01	817.1	-0.24	671.6	-0.3	146.5	-0.01
**Palau**	(11149.2	(0.02	(220.5	(-0.03	(3800.7	(0	(252.1	0 (0,0)	(3133.5	(0.02	(421.8	(0.11	(33.8	(-0.02	(21.4	(0.1	(2189.7	(0.01	(604.9	(-0.27	(463.7	(-0.33	(112.7	(-0.03
	,16206.8)	,0.04)	,348.2)	,0)	,6744.6)	,0.03)	,482.4)		,7511.2)	,0.08)	,632.1)	,0.15)	,115.6)	,0.01)	,239.4)	,0.23)	,3412.6)	,0.02)	,1093.7)	,-0.22)	,946.1)	,-0.26)	,186.8)	,0.01)
	18397.1	0.01	210.5	0.07	9283.6	0.04	744.1	-0.01	5158.6	0.06	566.8	0.27	99.9	0.14	925.1	-0.72	2710	0.02	419.4	-0.19	323.9	-0.2	95.8	-0.18
**Palestine**	(15445.1	(-0.04	(166.3	(0.05	(6759.6	(-0.05	(545.1	(-0.01	(3321.3	(0.01	(463.3	(0.23	(55.3	(0.11	(468	(-0.8	(2165	(0.01	(306	(-0.22	(212.8	(-0.23	(71.3	(-0.24
	,22272.7)	,0.06)	,266.9)	,0.08)	,12672.5)	,0.14)	,989.8)	,-0.01)	,7834.3)	,0.11)	,684.2)	,0.31)	,157.5)	,0.17)	,1358.6)	,-0.64)	,3376.5)	,0.03)	,552.9)	,-0.16)	,457.7)	,-0.16)	,126.7)	,-0.11)
**Panama**	13763.1	0.04	256.1	0.2	5711.7	-0.04	760	0 (0,0)	4288.7	0.22	570.3	0.15	104.1	-0.04	88.5	-1.8	2720.3	-0.02	2123.7	-0.35	1997	-0.37	129.8	-0.05
	(11555.1	(-0.01	(201.9	(0.19	(4198	(-0.09	(554.9		(2666.9	(0.13	(467	(0.14	(55.3	(-0.04	(13.5	(-1.97	(2173.5	(-0.02	(1573.2	(-0.5	(1448.8	(-0.53	(99.3	(-0.06

	,16324.9)	,0.08)	,323.7)	,0.22)	,7703.1)	,0.02)	,1022.9)		,6396.3)	,0.31)	,683.7)	,0.16)	,173.7)	,-0.04)	,200.2)	,-1.62)	,3388.4)	,-0.02)	,2790.9)	,-0.21)	,2662.8)	,-0.22)	,168.8)	,-0.04)
	13099.3	0.01	250.5	-0.03	4837.1	-0.05	354.1		4781.6	0.02	474.8	0.11	71.4	0.06	219.2	0.59	2770.8	0.03	942.9	0.18	816.8	0.2	127.1	0.04
**Papua New Guinea**	(10902.5	(-0.01	(197.4	(-0.04	(3654.2	(-0.07	(252.4	0 (0,0)	(2965.6	(-0.02	(385.9	(0.1	(35	(0.04	(50.9	(0.41	(2214.8	(0.03	(697.2	(0.14	(575.4	(0.16	(94.6	(0.01
	,15860.2)	,0.02)	,314.2)	,-0.01)	,6323.1)	,-0.04)	,481)		,7229.4)	,0.06)	,577.1)	,0.11)	,119.2)	,0.07)	,416.8)	,0.76)	,3451.2)	,0.03)	,1233.7)	,0.21)	,1107.7)	,0.24)	,165.6)	,0.06)
	16656.2	0.12	242.5	0.1	5224.5	0.21	916.1		7884.7	0.14	436.5	0.18	154.8	0.02	165.5	-0.41	2710	0	3516.3	0.95	3422.3	0.98	97.9	-0.09
**Paraguay**	(13636.4	(0.07	(189.8	(0.08	(3864.7	(0.1	(675.9	0 (0,0)	(5046.6	(0.06	(352.8	(0.17	(83.6	(0.02	(30.1	(-0.53	(2165.2	(0	(2481.6	(0.77	(2377.6	(0.8	(75.5	(-0.11
	,20338.3)	,0.16)	,306.2)	,0.11)	,7036.7)	,0.33)	,1225.6)		,11628.9)	,0.21)	,528.5)	,0.18)	,249.9)	,0.03)	,307.3)	,-0.3)	,3375.8)	,0)	,4764.2)	,1.13)	,4674.3)	,1.17)	,124.5)	,-0.07)
	14318	-0.01	244.9	0.17	4118.9	-0.31	827.9		6408.9	0.2	498.3	0.4	156.3	-0.02	141.3	-1.61	2717.4	0	1312.3	-1.14	1208.6	-1.23	105.1	0.03
**Peru**	(11747	(-0.08	(191.8	(0.15	(3103.8	(-0.42	(604.6	0 (0,0)	(4077	(0.08	(407.6	(0.39	(85.7	(-0.02	(24.7	(-1.74	(2171.2	(0	(975.4	(-1.26	(872.6	(-1.36	(82.6	(-0.02
	,17522.9)	,0.07)	,310.3)	,0.19)	,5417.9)	,-0.2)	,1102.8)		,9685.9)	,0.33)	,603)	,0.41)	,251.9)	,-0.01)	,275.1)	,-1.47)	,3384.3)	,0)	,1732.3)	,-1.03)	,1631.1)	,-1.1)	,131.5)	,0.09)
	14431.3	-0.07	254.4	0.1	5349.5	-0.29	428.4		5633.1	0.2	473.8	0.01	41.6	-0.02	265.6	-1.37	2781.6	-0.04	955.5	-0.75	858.2	-0.81	98.2	-0.2
**Philippines**	(12831.5	(-0.11	(214.3	(0.07	(4374.9	(-0.34	(333.1	0 (0,0)	(4379.2	(0.13	(386.1	(-0.01	(18.6	(-0.03	(71.6	(-1.63	(2224.9	(-0.04	(733.7	(-0.8	(634.8	(-0.86	(74.3	(-0.23
	,16322.3)	,-0.03)	,301.3)	,0.13)	,6458.7)	,-0.23)	,539.3)		,7269.6)	,0.26)	,568)	,0.03)	,72.4)	,-0.02)	,472.5)	,-1.11)	,3479.6)	,-0.04)	,1224)	,-0.7)	,1127.9)	,-0.75)	,126.8)	,-0.16)
	12539.5	-0.03	221.9	0.25	4059.8	-0.13	566.3	-0.01	4647.5	0.02	712.6	0.39	52.7	0.07	131.7	-2.26	2766.5	0.03	3999.8	0.9	3877.2	0.93	128.5	-0.06
**Poland**	(11112.3	(-0.1	(186.4	(0.24	(3317.8	(-0.29	(445.9	(-0.01	(3579.5	(-0.05	(581.9	(0.37	(24.6	(0.07	(18.7	(-2.34	(2212.7	(0.03	(3031.6	(0.69	(2905.9	(0.72	(100.6	(-0.08
	,14143.9)	,0.04)	,261.3)	,0.26)	,4927.6)	,0.04)	,709.2)	,-0.01)	,6041.3)	,0.1)	,851.9)	,0.4)	,91.3)	,0.08)	,271.9)	,-2.18)	,3459.2)	,0.03)	,5166.8)	,1.1)	,5052)	,1.14)	,160.1)	,-0.03)
	19856.8	-0.11	289.3	0	7509.9	-0.58	1003.5	-0.03	9017.7	0.22	616.5	0.35	57	0.03	195	-1.28	2698	0.02	2380.7	0.15	2233.1	0.13	151.2	0.55
**Portugal**	(16515.7	(-0.23	(228	(0	(5503	(-0.83	(765	(-0.04	(6004.8	(0.07	(503.2	(0.33	(25.9	(0.03	(49.5	(-1.4	(2175.6	(0.02	(1742.6	(0.01	(1592.2	(-0.02	(123.9	(0.49
	,23960.8)	,0)	,362.8)	,0.01)	,10047)	,-0.34)	,1307.2)	,-0.03)	,13143.7)	,0.37)	,741.2)	,0.37)	,98)	,0.03)	,359.3)	,-1.17)	,3329.3)	,0.02)	,3113)	,0.29)	,2968.3)	,0.28)	,184.2)	,0.61)
	13655.6	0.05	253.9	0.09	4696	-0.04	832.9		5093.2	0.18	541.8	0.22	223.1	-0.02	63.7	-1.02	2689.5	-0.01	2295.7	-1.22	2135.6	-1.29	164.5	-0.25
**Puerto Rico**	(11561.8	(0.01	(199.5	(0.07	(3528.5	(-0.11	(615.3	0 (0,0)	(3269.7	(0.12	(439.9	(0.2	(126.6	(-0.03	(8.2	(-1.23	(2149	(-0.01	(1686.5	(-1.41	(1522.9	(-1.49	(128.9	(-0.42
	,16391.5)	,0.09)	,319.6)	,0.11)	,6205.8)	,0.04)	,1108.5)		,7509.4)	,0.24)	,658.1)	,0.23)	,347.9)	,-0.01)	,156.2)	,-0.8)	,3348.2)	,-0.01)	,3024.9)	,-1.04)	,2872.6)	,-1.09)	,209.3)	,-0.08)
	14098.6	-0.01	264.6	0.21	5776.2	-0.06	737.5	0	4247.5	0.06	748.8	0.46	110.8	0.07	173.5	-1.86	2827.6	0	491.3	-0.16	394.3	-0.23	97.3	0.12
**Qatar**	(11827.7	(-0.03	(209.3	(0.18	(4258.7	(-0.1	(538.4	(-0.01	(2718.1	(0	(607.5	(0.42	(61.4	(0.04	(27.7	(-2.07	(2260.4	(-0.01	(353.9	(-0.22	(255.7	(-0.29	(73.3	(0.04
	,16790.5)	,0.02)	,332.6)	,0.24)	,7746.1)	,-0.02)	,981.6)	,0)	,6433.7)	,0.12)	,903.3)	,0.5)	,175.1)	,0.1)	,346.7)	,-1.65)	,3523.5)	,0.02)	,671.8)	,-0.11)	,575.1)	,-0.17)	,126.5)	,0.19)
	10843.1	0.25	253.7	0.03	3278.7	0.86	559.4	0	3297.2	-0.12	1155.9	0.75	52.8	0.2	5.4	-9.69	2708.3	0.09	2981.2	-0.23	2823	-0.23	163.5	-0.39
**Republic of Korea**	(9299.7	(0.18	(203.1	(0.02	(2461.3	(0.55	(409.8	(-0.01	(2073.6	(-0.23	(943.2	(0.71	(24.2	(0.18	(0	(-10.34	(2184.3	(0.09	(2166.1	(-0.46	(2005	(-0.46	(128.9	(-0.5
	,12818)	,0.32)	,313.8)	,0.04)	,4234.4)	,1.17)	,757.3)	,0)	,5112.1)	,-0.02)	,1376.9)	,0.8)	,92.6)	,0.23)	,33.1)	,-9.03)	,3342.3)	,0.1)	,3935.4)	,-0.01)	,3779)	,0.01)	,203.3)	,-0.29)
	15731.1	-0.13	185.5	0.08	7004.9	-0.38	553.1		5328.4	0.08	644.7	0.24	60.6	0.02	254.8	-0.61	2644.7	0.01	3726.8	0.52	3584.9	0.56	148	-0.49
**Republic of Moldova**	(13289	(-0.19	(145.9	(0.03	(5240.9	(-0.46	(398.1	0 (0,0)	(3364.1	(0	(524.5	(0.21	(29.3	(0.01	(54	(-1.12	(2113.4	(0.01	(2918	(0.32	(2778.1	(0.35	(117.7	(-0.56
	,18914.8)	,-0.08)	,234)	,0.13)	,9448.3)	,-0.29)	,745.6)		,8206.6)	,0.16)	,773.1)	,0.26)	,100.4)	,0.03)	,467.9)	,-0.09)	,3291)	,0.01)	,4879.3)	,0.71)	,4746.5)	,0.77)	,183.1)	,-0.42)
	13948.6	0.03	218.2	0.23	5545.1	-0.02	638.2		4759	0.16	686.7	0.33	62.2	-0.01	134.8	-1.91	2670.6	-0.01	2833.5	-1.58	2742.4	-1.62	93.9	-0.21
**Romania**	(11766.6	(-0.02	(172.6	(0.2	(4137.7	(-0.08	(476.4	0 (0,0)	(3027.6	(0.04	(563.7	(0.32	(30.2	(-0.02	(19.8	(-2.15	(2133.2	(-0.01	(2123.9	(-1.78	(2027.2	(-1.82	(74.5	(-0.31
	,16780.6)	,0.09)	,273.9)	,0.26)	,7309.5)	,0.05)	,838.8)		,7363.1)	,0.27)	,823.5)	,0.35)	,103.2)	,0)	,281)	,-1.66)	,3325.1)	,-0.01)	,3741.4)	,-1.38)	,3652)	,-1.41)	,116.7)	,-0.11)
	14257.7	-0.12	201.1	0.34	5994	-0.36	571.1	-0.01	4732.6	0.01	650.8	0.42	50.6	0.06	115.3	-1.1	2719.6	0.05	4955.1	-0.26	4733.6	-0.24	234.7	-0.89
**Russian Federation**	(12651.7	(-0.18	(168.9	(0.25	(4788.5	(-0.46	(447.5	(-0.01	(3678.8	(-0.08	(532	(0.39	(23.6	(0.04	(14.7	(-1.56	(2176.3	(0.05	(3801.2	(-0.43	(3572.9	(-0.4	(190	(-1.11
	,16080.4)	,-0.07)	,237.4)	,0.42)	,7383.8)	,-0.27)	,716)	,0)	,6080.9)	,0.1)	,779.4)	,0.45)	,87.5)	,0.08)	,249.8)	,-0.63)	,3394.3)	,0.05)	,6358.3)	,-0.1)	,6138.3)	,-0.07)	,284.6)	,-0.67)
	18510.8	-0.21	189.3	0.19	11458.8	-0.43	646.1		3965.1	0.23	563.7	0.33	22	-0.01	144.9	-0.25	2653.9	-0.04	4557.4	-0.16	4463	-0.17	99.7	0.12
**Rwanda**	(15285	(-0.26	(146.6	(0.15	(8420.9	(-0.5	(468	0 (0,0)	(2487.2	(0.18	(455.2	(0.28	(8.9	(-0.04	(30.4	(-0.62	(2120.6	(-0.05	(3303	(-0.29	(3204.3	(-0.29	(77.2	(0.1
	,22579.1)	,-0.16)	,240.9)	,0.23)	,15440.5)	,-0.36)	,873.6)		,5986)	,0.27)	,678.1)	,0.38)	,40.8)	,0.02)	,326.7)	,0.13)	,3303)	,-0.03)	,5968.1)	,-0.03)	,5871.1)	,-0.04)	,127.4)	,0.13)

	15730.6	0.05	247.3	0.18	7091.7	0.02	815.9		5065.9	0.1	475.8	0.3	227.8	0.12	71	-1.02	2688	0.02	4503	0.07	4359.9	0.07	151.1	0.08
**Saint Kitts and Nevis**	(12932.8	(0.01	(192.9	(0.17	(4951.6	(-0.02	(595.6	0 (0,0)	(3241.7	(0.03	(386.1	(0.26	(129.7	(0.1	(9.5	(-1.06	(2147.2	(0.02	(3288.6	(-0.05	(3149.3	(-0.05	(115.9	(0.04
	,18958)	,0.08)	,312)	,0.2)	,9837.8)	,0.07)	,1089.6)		,7635.4)	,0.17)	,570.9)	,0.34)	,354.8)	,0.14)	,166.6)	,-0.98)	,3347.7)	,0.02)	,5952.4)	,0.18)	,5819.3)	,0.18)	,195.7)	,0.12)
	14533.4	0.06	236	0.1	5619.4	0.06	820.3		5141.1	0.07	490.6	0.27	230.1	0.14	117.9	-0.52	2710.3	0.05	2576.5	-0.16	2417.4	-0.17	163.8	0.02
**Saint Lucia**	(12151	(0.01	(184.8	(0.09	(4146.2	(-0.02	(602	0 (0,0)	(3250.2	(-0.01	(398.7	(0.25	(130.8	(0.13	(16.8	(-0.58	(2165.6	(0.04	(1929.8	(-0.3	(1774.4	(-0.32	(121.2	(-0.01
	,17473.1)	,0.11)	,297.5)	,0.11)	,7526.4)	,0.14)	,1096.3)		,7721)	,0.15)	,591.8)	,0.3)	,358.5)	,0.15)	,248.2)	,-0.45)	,3376)	,0.05)	,3359.7)	,-0.02)	,3209.2)	,-0.02)	,215.6)	,0.05)
	14444.3	0.08	234.1	0.18	5670.1	0.15	814.5	-0.01	4959.6	0	485.9	0.31	237.6	0.24	124	-0.81	2741.3	0.08	2686.9	0.22	2541.2	0.23	150.4	0.11
**Saint Vincent and the Grenadines**	(12103.6	(0.04	(183.1	(0.16	(4145.7	(0.09	(595.8	(-0.01	(3125.8	(-0.06	(393.4	(0.29	(135.5	(0.23	(16.2	(-0.87	(2190.5	(0.08	(1971	(0.11	(1824.5	(0.11	(114.6	(0.06
	,17322.7)	,0.11)	,293.8)	,0.19)	,7552.1)	,0.21)	,1094.7)	,-0.01)	,7438.1)	,0.07)	,583.6)	,0.32)	,370.6)	,0.25)	,260.5)	,-0.75)	,3415.9)	,0.09)	,3499.2)	,0.33)	,3355.6)	,0.35)	,195.7)	,0.15)
	13108.3	-0.01	257.5	0.02	4696.8	-0.13	354.8		4974.5	0.1	499.6	0.12	69.3	-0.01	194.5	-0.45	2720.3	0	928.6	-0.15	778.7	-0.18	151.1	0
**Samoa**	(10968	(-0.03	(204.5	(0.01	(3543.6	(-0.15	(252	0 (0,0)	(3193.5	(0.07	(406.6	(0.11	(34	(-0.03	(43.9	(-0.56	(2173.3	(-0.01	(696	(-0.18	(549.9	(-0.21	(113.6	(-0.02
	,15719.1)	,0.01)	,321.2)	,0.03)	,6103.5)	,-0.1)	,481.8)		,7578)	,0.13)	,599.6)	,0.13)	,115.9)	,0.01)	,368.8)	,-0.34)	,3389.2)	,0)	,1198.3)	,-0.12)	,1046.3)	,-0.14)	,194.9)	,0.01)
	15915.1	0.04	302.7	-0.07	5885.9	0.02	1060.7	0.09	6072.9	0.07	673.8	0.2	58.1	0.04	134.1	-0.58	2734.8	0.04	1152.8	-0.13	981.2	-0.17	173.5	0.1
**San Marino**	(13091.7	(0	(239.6	(-0.09	(4142.3	(-0.03	(785.9	(0.07	(3890.3	(-0.01	(550.4	(0.17	(26.5	(0.03	(25.7	(-0.93	(2206.4	(0.04	(831.7	(-0.27	(664.5	(-0.32	(139.9	(0.03
	,19226.3)	,0.08)	,378.2)	,-0.04)	,8116.6)	,0.07)	,1404.7)	,0.1)	,9101.4)	,0.14)	,810.5)	,0.22)	,99.8)	,0.06)	,273.9)	,-0.22)	,3373.9)	,0.04)	,1558.7)	,0)	,1389.3)	,-0.02)	,215)	,0.17)
	14135.1	0.08	210.9	0.16	7592.3	0.13	571.3		2983.1	0.05	630.9	0.24	23.3	-0.01	125.8	-0.3	2704.6	0	921.9	-0.03	810.7	-0.08	112.2	0.41
**Sao Tome and Principe**	(11859.5	(0.06	(164.2	(0.13	(5583.5	(0.09	(415.8	0 (0,0)	(1834.2	(0.01	(511	(0.22	(9.4	(-0.02	(16.3	(-0.43	(2160.6	(0	(687.5	(-0.11	(580.5	(-0.18	(91.6	(0.36
	,16899.4)	,0.11)	,264.7)	,0.18)	,10234.1)	,0.17)	,766.4)		,4606.4)	,0.09)	,756.5)	,0.26)	,43.8)	,0)	,292.3)	,-0.16)	,3369.2)	,0.01)	,1206.4)	,0.05)	,1093.3)	,0.01)	,136.3)	,0.45)
	14089.3	0.13	241.3	0.11	5964.4	0.27	747.1	-0.02	4203.6	0.05	626.8	0.54	92.5	0.13	174.1	-1	2821.1	0.05	471.5	-0.15	366.7	-0.25	105.2	0.23
**Saudi Arabia**	(11827.4	(0.11	(191.9	(0.09	(4427.3	(0.24	(550.3	(-0.03	(2603.7	(0	(506.2	(0.49	(49.8	(0.06	(25.3	(-1.12	(2254.4	(0.03	(337.3	(-0.17	(237.5	(-0.28	(80.2	(0.18
	,16678.6)	,0.15)	,301.1)	,0.13)	,7958.3)	,0.3)	,1002.5)	,-0.01)	,6515.6)	,0.09)	,757.8)	,0.58)	,146.4)	,0.19)	,342.5)	,-0.88)	,3514)	,0.06)	,629.6)	,-0.12)	,522.7)	,-0.23)	,136.5)	,0.29)
	15660.6	0.08	207.7	0.12	9316	0.1	568.3		2968.7	0.12	600.9	0.09	23.3	-0.06	73.1	1.64	2717.1	-0.03	890.8	0.06	796.4	0.07	95.1	-0.05
**Senegal**	(13093.8	(0.05	(161.6	(0.1	(6848.6	(0.07	(412.3	0 (0,0)	(1889.7	(0.06	(484.8	(0.09	(9.4	(-0.07	(16.2	(1.49	(2171	(-0.04	(658.6	(-0.02	(566.8	(-0.02	(75.5	(-0.06
	,18957.1)	,0.11)	,262)	,0.13)	,12727.1)	,0.14)	,764.1)		,4508.1)	,0.18)	,722.8)	,0.1)	,43.8)	,-0.05)	,222)	,1.8)	,3384.3)	,-0.03)	,1159.7)	,0.14)	,1064.5)	,0.16)	,117.2)	,-0.04)
	14575.9	-0.1	213.6	0.14	6214.8	-0.32	543.5		4784.2	0.11	725.1	0.37	62.9	0.03	170.2	-1.21	2690.9	0.01	3597.1	-0.16	3508.6	-0.17	91.9	0.17
**Serbia**	(12286.2	(-0.14	(168.3	(0.11	(4633.4	(-0.38	(394.9	0 (0,0)	(3037.7	(0.04	(587.4	(0.35	(30.6	(0.02	(27	(-1.51	(2149.7	(0.01	(2656.6	(-0.3	(2564.6	(-0.32	(71.4	(0.13
	,17492.1)	,-0.05)	,267.7)	,0.17)	,8267.4)	,-0.26)	,725.6)		,7257.2)	,0.18)	,867.8)	,0.38)	,104.5)	,0.03)	,342.1)	,-0.91)	,3351.2)	,0.02)	,4664.9)	,-0.02)	,4580.5)	,-0.03)	,116)	,0.21)
	13178.5	-0.02	283.5	0.15	4732.1	-0.14	413.1		4976.3	0.05	537.6	0.35	51.3	0.15	142.1	-1.11	2707.2	0.05	866.7	-0.12	739.7	-0.14	128	-0.02
**Seychelles**	(11152.2	(-0.05	(226.2	(0.12	(3590.3	(-0.18	(297.3	0 (0,0)	(3180	(-0.02	(435.1	(0.33	(23.9	(0.12	(30.9	(-1.29	(2162.6	(0.04	(622	(-0.21	(497	(-0.23	(95.9	(-0.06
	,15793.7)	,0.02)	,351.3)	,0.17)	,6119.6)	,-0.1)	,560.2)		,7535.3)	,0.11)	,646.9)	,0.37)	,89)	,0.18)	,280.8)	,-0.93)	,3373)	,0.06)	,1157.4)	,-0.04)	,1033.3)	,-0.05)	,166.1)	,0.03)
	16262.5	0.21	194.8	0.04	9716.7	0.33	564.2		3171.7	0.1	594.1	0.12	23.8	0.01	140.6	1.92	2737.2	-0.02	949.8	0.11	855.5	0.13	95.2	-0.03
**Sierra Leone**	(13550.5	(0.18	(151.3	(0.01	(7171.1	(0.27	(409.2	0 (0,0)	(1960.9	(0.08	(479.9	(0.07	(9.6	(-0.02	(24.2	(1.71	(2186.9	(-0.03	(702.7	(0.02	(609.1	(0.03	(75.4	(-0.04
	,19656.8)	,0.24)	,249.1)	,0.07)	,13006.5)	,0.38)	,764.1)		,4761.1)	,0.13)	,719)	,0.16)	,44.7)	,0.05)	,347.2)	,2.13)	,3411)	,-0.01)	,1267.2)	,0.2)	,1173.2)	,0.22)	,118.6)	,-0.02)
	10036	-0.38	249.9	0.09	2843.1	-1.33	641.5	0.01	2736.3	-0.09	1173.3	0.45	54.8	0.15	2.4	-10.81	2739.7	0.04	818	0.19	662.5	0.24	156.7	-0.03
**Singapore**	(8597.8	(-0.43	(198.5	(0.08	(2112.2	(-1.49	(472.6	(0	(1689.3	(-0.12	(970.6	(0.44	(25.5	(0.12	(0	(-11.62	(2210.8	(0.03	(576.2	(0.12	(425.7	(0.17	(123.5	(-0.07
	,11673)	,-0.33)	,309.9)	,0.1)	,3815.8)	,-1.17)	,855.2)	,0.01)	,4199.8)	,-0.05)	,1397.5)	,0.47)	,95.2)	,0.18)	,20.8)	,-10)	,3380.2)	,0.05)	,1129.2)	,0.25)	,966.6)	,0.31)	,196.7)	,0.01)
	14415	-0.07	223.9	0.2	5898.3	-0.25	547.1	0	4993.7	0.07	717.6	0.28	62.6	0.07	116.4	-1.67	2669	0.01	2975.9	0.02	2842.8	0.02	137.4	-0.12
**Slovakia**	(12146.5	(-0.15	(176.1	(0.18	(4351	(-0.41	(398.7	(-0.01	(3142.3	(-0.01	(586.3	(0.27	(30.4	(0.06	(16.3	(-1.84	(2131.9	(0	(2128.9	(-0.12	(2000.7	(-0.12	(108.1	(-0.14
	,17335.2)	,0)	,281.7)	,0.21)	,7833.5)	,-0.09)	,732.7)	,0)	,7687.1)	,0.14)	,865.2)	,0.29)	,103.8)	,0.09)	,256.2)	,-1.5)	,3323.5)	,0.02)	,4056.2)	,0.15)	,3922.5)	,0.16)	,170.8)	,-0.09)
**Slovenia**	15134.5	-0.3	226.9	0.14	6844.8	-0.7	551.4	-0.02	4729.8	-0.09	801.6	0.56	63.9	0.21	105.6	-1.61	2698.5	0.09	5282	0.4	5144.5	0.4	145.5	0.66
	(12830.3	(-0.35	(177.3	(0.13	(4980.9	(-0.77	(403.4	(-0.02	(3019.3	(-0.16	(654.8	(0.54	(31.1	(0.19	(14.5	(-1.75	(2155.7	(0.09	(4001.6	(0.26	(3859.8	(0.25	(114.5	(0.58

	,17950.3)	,-0.25)	,285.5)	,0.15)	,9155.9)	,-0.62)	,741.1)	,-0.02)	,7161.6)	,-0.02)	,974)	,0.58)	,106)	,0.23)	,243.8)	,-1.48)	,3361.8)	,0.09)	,6705.2)	,0.55)	,6560.2)	,0.55)	,182.2)	,0.73)
	13303	0.07	233.8	-0.14	4904.4	0.03	352.7	-0.01	4930.2	0.22	463.9	-0.06	69.6	-0.13	291.4	0.44	2738.2	-0.08	968.9	-0.2	842.7	-0.22	127.2	-0.08
**Solomon Islands**	(11176.9	(0.05	(183.6	(-0.17	(3706.8	(0.01	(251.5	(-0.01	(3093.8	(0.19	(374.7	(-0.08	(34.1	(-0.15	(83.3	(0.29	(2187.8	(-0.08	(732.1	(-0.24	(606.6	(-0.26	(95.4	(-0.09
	,15925.9)	,0.09)	,295.1)	,-0.11)	,6376.5)	,0.05)	,482.5)	,-0.01)	,7407.7)	,0.25)	,563.4)	,-0.05)	,116.4)	,-0.12)	,507.8)	,0.59)	,3411.4)	,-0.07)	,1267.1)	,-0.17)	,1142.1)	,-0.18)	,163.8)	,-0.08)
	18126.4	0.08	158.8	-0.07	11025.7	0.09	645.8		3838.1	0.18	506.6	-0.07	21.8	-0.09	389.3	0.65	2643.6	-0.07	1953.6	0.08	1865	0.08	90.4	-0.06
**Somalia**	(15025.3	(0.05	(118	(-0.09	(8035.8	(0.05	(467.6	0 (0,0)	(2405	(0.12	(406.3	(-0.09	(8.8	(-0.1	(109.8	(0.46	(2112.9	(-0.07	(1427.7	(-0.02	(1340.3	(-0.02	(69.5	(-0.08
	,22058.2)	,0.12)	,205.2)	,-0.04)	,14834.3)	,0.13)	,869.9)		,5902.8)	,0.24)	,607.4)	,-0.05)	,40.5)	,-0.08)	,771.9)	,0.84)	,3289.5)	,-0.06)	,2544.1)	,0.18)	,2459.6)	,0.19)	,115.3)	,-0.05)
	16263.3	0.01	212.4	0.13	8348.9	-0.05	622.5		4637	0.09	595.7	0.22	17	0.05	50.9	-0.77	2746.8	0	2369.4	-0.06	2038.4	-0.05	340.1	-0.15
**South Africa**	(14452.8	(-0.06	(177.5	(0.11	(6856.9	(-0.16	(487.5	0 (0,0)	(3567.3	(0.01	(486.5	(0.16	(6	(0.04	(10	(-0.93	(2197.7	(-0.01	(1836	(-0.13	(1499.6	(-0.13	(287	(-0.4
	,18356.1)	,0.07)	,253.5)	,0.14)	,10187.9)	,0.05)	,778.5)		,6121.5)	,0.18)	,713.1)	,0.28)	,32.9)	,0.07)	,148.7)	,-0.61)	,3431.2)	,0.01)	,3008.7)	,0)	,2676.2)	,0.03)	,399.9)	,0.09)
	17573.3	0.12	190.1	-0.1	10055	0.14	645	0.01	4319.6	0.28	600.5	-0.02	23.6	-0.19	51.3	1.28	2763.8	-0.07	2111.9	-0.57	2013.9	-0.59	100.1	-0.04
**South Sudan**	(14528.5	(0.1	(147.9	(-0.14	(7383.9	(0.1	(468.2	(0	(2722.7	(0.23	(482.8	(-0.03	(9.5	(-0.2	(19.3	(0.9	(2208.8	(-0.07	(1565.9	(-0.65	(1473.9	(-0.68	(77.9	(-0.08
	,21221.7)	,0.15)	,243.8)	,-0.06)	,13523.5)	,0.17)	,871.7)	,0.01)	,6692)	,0.33)	,726.3)	,-0.01)	,43)	,-0.17)	,158.5)	,1.66)	,3443.3)	,-0.06)	,2811.4)	,-0.48)	,2712.5)	,-0.5)	,126.9)	,-0.01)
	16379.2	0.44	294.9	0	6553.8	0.96	1046.9	0.09	5418.2	0.45	754.4	0.42	146.4	0.1	552.3	-1.48	2709.8	0.03	1411.2	-0.06	1142.9	-0.09	271.9	0.07
**Spain**	(14013.2	(0.31	(269.2	(0	(4926.1	(0.74	(781.5	(0.07	(3433	(0.18	(618.5	(0.38	(78.5	(0.07	(226.4	(-1.61	(2185.6	(0.03	(1065.7	(-0.21	(798.1	(-0.23	(222.8	(-0.17
	,19487.3)	,0.57)	,323.2)	,0.01)	,8619.9)	,1.17)	,1399.2)	,0.11)	,8042.7)	,0.71)	,894.9)	,0.47)	,239)	,0.13)	,874.5)	,-1.35)	,3344.2)	,0.03)	,1878.7)	,0.09)	,1604.5)	,0.06)	,322.6)	,0.31)
	13161.6	-0.08	267.1	0.22	4469.8	-0.38	415.8		5174.7	0.27	565	0.2	49.7	-0.11	203.7	-2.07	2680.1	-0.08	1191	-1.15	1081	-1.22	111.3	-0.43
**Sri Lanka**	(11038.9	(-0.12	(212.4	(0.19	(3345.1	(-0.44	(300.6	0 (0,0)	(3287.5	(0.21	(458.1	(0.17	(23.2	(-0.12	(48.6	(-2.23	(2141.1	(-0.08	(869.4	(-1.36	(759.5	(-1.44	(84.1	(-0.47
	,15849.7)	,-0.04)	,329.9)	,0.26)	,5860.6)	,-0.32)	,558.9)		,7925.9)	,0.33)	,681)	,0.23)	,86.3)	,-0.1)	,386.1)	,-1.9)	,3336.8)	,-0.07)	,1603.9)	,-0.95)	,1491.7)	,-1)	,142.4)	,-0.39)
	14666.4	-0.03	202.4	0.15	5816.6	0.1	726.9	-0.01	4467.6	0.05	530.7	0.31	103.2	0.02	911.5	-1.47	2781.3	0.03	474.8	-0.15	370.4	-0.29	104.8	0.4
**Sudan**	(12419.3	(-0.07	(158.4	(0.14	(4247.1	(0.06	(535.3	(-0.01	(2771.9	(-0.02	(428.3	(0.31	(57	(0.01	(440.2	(-1.58	(2223.2	(0.02	(349.6	(-0.17	(246.5	(-0.33	(81.8	(0.38
	,17369.7)	,0)	,254.1)	,0.16)	,7818.7)	,0.14)	,973.2)	,0)	,6796.8)	,0.12)	,643.3)	,0.32)	,162.9)	,0.04)	,1359.8)	,-1.36)	,3463.4)	,0.03)	,636.5)	,-0.12)	,532.1)	,-0.25)	,131.1)	,0.42)
	16431.2	0.17	218.4	0.1	7809.5	0.3	815.6		5138.6	0.19	448.5	0.04	227	-0.1	109	-0.63	2693.7	-0.04	2054.7	-0.14	1918.3	-0.16	139.8	0.03
**Suriname**	(13629.5	(0.12	(175.4	(0.08	(5580.7	(0.25	(597.3	0 (0,0)	(3228.8	(0.1	(360.8	(0.03	(129.3	(-0.12	(13.9	(-0.77	(2151.7	(-0.05	(1488.3	(-0.21	(1355.8	(-0.22	(108	(0
	,19814.7)	,0.21)	,269.6)	,0.12)	,10583.6)	,0.36)	,1086)		,7644.6)	,0.27)	,540.9)	,0.05)	,353.4)	,-0.08)	,233.6)	,-0.5)	,3354.9)	,-0.04)	,2785.6)	,-0.08)	,2653.1)	,-0.09)	,179.8)	,0.05)
	15586.6	0.05	355.7	-0.05	5827.4	0.06	1077.3	0.07	5534	0.04	733.7	0.32	75.6	0.15	196.7	-0.5	2767.1	0.04	2287.3	0.17	2082.9	0.02	209.2	2.21
**Sweden**	(13712.9	(0.02	(297	(-0.05	(4580.5	(0.01	(855.3	(0.05	(3986.4	(-0.02	(590.7	(0.29	(38.5	(0.13	(46.1	(-0.73	(2235.5	(0.04	(1706.7	(0.08	(1502	(-0.07	(175.3	(2.12
	,17852.2)	,0.08)	,419.2)	,-0.05)	,7443.6)	,0.11)	,1362.1)	,0.08)	,7517.9)	,0.1)	,888.5)	,0.35)	,123.9)	,0.17)	,374.5)	,-0.27)	,3410.6)	,0.05)	,2962.8)	,0.25)	,2762.4)	,0.12)	,247.6)	,2.3)
	17518.3	-0.04	304.4	-0.03	6181.2	-0.35	1059.1	0.1	7577.5	0.13	672.7	0.32	77.2	0.08	123.1	-1.08	2725.2	0.06	2647.7	0.11	2456.5	0.11	196.8	0.09
**Switzerland**	(14754.9	(-0.14	(242	(-0.04	(4503	(-0.67	(785.8	(0.08	(5133.9	(0.08	(547.7	(0.3	(37.7	(0.08	(22.9	(-1.12	(2198.3	(0.06	(2040.6	(0.05	(1846.5	(0.04	(159.4	(0.06
	,20812.5)	,0.07)	,382.1)	,-0.01)	,8400.1)	,-0.03)	,1404.4)	,0.12)	,10790.7)	,0.18)	,808.9)	,0.35)	,130.3)	,0.09)	,255.1)	,-1.03)	,3362.9)	,0.06)	,3462.6)	,0.16)	,3277.6)	,0.17)	,239.7)	,0.12)
	14996.8	0.06	208.5	0.03	5732.4	0.11	740	0.01	4996.4	0.16	579.2	0.28	100.4	-0.04	802.7	-0.69	2754.7	-0.01	609.4	0.32	469.6	0.14	140.4	1.04
**Syrian Arab Republic**	(12637.1	(0.02	(165.6	(0.01	(4246.2	(0.05	(544.4	(0.01	(3172.8	(0.1	(468.9	(0.24	(55.6	(-0.05	(326.8	(-0.81	(2200.8	(-0.02	(460.3	(0.27	(322.1	(0.07	(115.8	(1
	,18111.4)	,0.09)	,260.7)	,0.05)	,7815.8)	,0.16)	,987)	,0.01)	,7616)	,0.22)	,703.4)	,0.32)	,158.4)	,-0.04)	,1280.5)	,-0.57)	,3431.3)	,-0.01)	,803.6)	,0.37)	,662.5)	,0.2)	,169.1)	,1.07)
	13602.3	0.07	312.3	-0.02	5356	0.13	250.5		4847.9	0.15	747.6	0.17	59.2	-0.2	28.9	-3.42	2704.8	-0.08	503.1	0.09	364	0.19	139.7	-0.19
**Taiwan (Province of China)**	(11438.3	(0.06	(249.4	(-0.05	(4062.7	(0.11	(179	0 (0,0)	(3081.1	(0.12	(608.2	(0.14	(28.9	(-0.25	(2.9	(-3.65	(2160.9	(-0.09	(388.7	(-0.01	(256.8	(0.07	(110.6	(-0.23
	,16189.9)	,0.09)	,388.7)	,0.01)	,6981.5)	,0.15)	,341)		,7362.9)	,0.17)	,902.6)	,0.2)	,99.5)	,-0.16)	,97.8)	,-3.18)	,3368.7)	,-0.08)	,646.7)	,0.18)	,505.4)	,0.32)	,172.6)	,-0.14)
	12892.1	-0.11	188.5	-0.01	6151.8	-0.32	550.9	-0.01	2915.1	-0.03	603.4	0.25	63.6	0.06	324.6	0.61	2721.8	0.08	2829.8	-0.65	2707.1	-0.66	126.1	-0.36
**Tajikistan**	(10914.7	(-0.15	(148.1	(-0.08	(4566.3	(-0.4	(396.5	(-0.01	(1829.3	(-0.11	(492.4	(0.23	(30.8	(0.01	(67.1	(-0.13	(2174.6	(0.07	(1958.8	(-0.96	(1840.4	(-0.98	(96.9	(-0.42
	,15193.8)	,-0.07)	,238.8)	,0.06)	,8132.1)	,-0.24)	,735.2)	,0)	,4486.2)	,0.06)	,728)	,0.26)	,105.5)	,0.1)	,588.8)	,1.35)	,3389.4)	,0.09)	,3924.4)	,-0.34)	,3806.6)	,-0.35)	,158.5)	,-0.3)

	13313.5	0.11	270.9	0.21	5213.7	0.33	416.1		4456.7	0.1	550.3	0.35	127.8	-0.06	270.3	-2.1	2695	0	1106.2	-0.77	974.7	-0.87	132.8	0.04
**Thailand**	(11346.8	(0.09	(215.4	(0.19	(3952.1	(0.3	(296.8	0 (0,0)	(2813.3	(0.06	(447.2	(0.33	(69.4	(-0.07	(47.9	(-2.21	(2153.1	(0	(787.6	(-0.82	(658.9	(-0.94	(101	(-0.03
	,15706)	,0.12)	,337.6)	,0.23)	,6741.8)	,0.36)	,563.3)		,6808.4)	,0.13)	,666.7)	,0.37)	,204.8)	,-0.05)	,495.4)	,-1.98)	,3355.6)	,0)	,1527)	,-0.72)	,1402.1)	,-0.81)	,169.4)	,0.1)
	13057	-0.04	229.8	0.21	4789.2	-0.11	397.9		4759.1	0.17	474.6	0.18	51	-0.08	287.8	-2.34	2724	-0.03	922.2	-0.3	824.1	-0.33	98.9	-0.07
**Timor-Leste**	(11022.7	(-0.07	(181.2	(0.16	(3616.4	(-0.14	(283.8	0 (0,0)	(3020.2	(0.12	(385.4	(0.16	(23.8	(-0.1	(75.7	(-2.61	(2176.3	(-0.04	(680.9	(-0.33	(586.5	(-0.37	(73.3	(-0.1
	,15431.3)	,-0.01)	,285.4)	,0.25)	,6335.9)	,-0.08)	,538.2)		,7113.7)	,0.23)	,574.1)	,0.21)	,88.4)	,-0.06)	,504.1)	,-2.06)	,3392.8)	,-0.02)	,1256.1)	,-0.26)	,1157.4)	,-0.29)	,129.9)	,-0.03)
	16791.4	0.18	197.7	0.08	10285.6	0.26	567.3		3305.8	0.2	566.3	-0.02	22.6	-0.11	108.9	1.96	2655.6	-0.09	940.6	0.14	854.1	0.13	87.2	0.22
**Togo**	(14004.1	(0.16	(152.8	(0.07	(7440.7	(0.24	(409.2	0 (0,0)	(2080.7	(0.17	(455.1	(-0.07	(9.1	(-0.16	(22	(1.67	(2121.3	(-0.1	(695	(0.07	(611.2	(0.06	(70.3	(0.2
	,20514.9)	,0.19)	,251.2)	,0.09)	,13880.2)	,0.28)	,759.9)		,5060.8)	,0.23)	,685.1)	,0.02)	,42.2)	,-0.07)	,298.2)	,2.26)	,3305.8)	,-0.08)	,1238.3)	,0.21)	,1148.8)	,0.2)	,107.1)	,0.24)
	13537.3	0.06	260.2	0.04	5156.3	0.06	354.8		5015.1	0.15	511.4	0.12	68.4	-0.08	161.5	-0.9	2709.7	-0.04	861	-0.26	725.2	-0.3	136.8	-0.04
**Tokelau**	(11258.9	(0.04	(205.7	(0.02	(3767	(0.05	(253.2	0 (0,0)	(3203.7	(0.12	(417.2	(0.08	(33.6	(-0.16	(31.1	(-0.96	(2165.1	(-0.05	(649.6	(-0.3	(512.6	(-0.34	(103	(-0.05
	,16159.2)	,0.07)	,325.5)	,0.06)	,6836.7)	,0.07)	,483.3)		,7528.6)	,0.18)	,620)	,0.16)	,114.5)	,-0.01)	,324.7)	,-0.83)	,3375.1)	,-0.03)	,1117.8)	,-0.23)	,988.3)	,-0.26)	,175.2)	,-0.03)
	12998.2	0.05	259.7	-0.03	4527.3	0.03	357.2		5033.2	0.13	502	0.02	68.4	-0.06	196.2	-0.09	2703.3	-0.03	860.6	-0.17	723.3	-0.2	138.2	0.03
**Tonga**	(10893.7	(0.03	(204.7	(-0.04	(3434.9	(0.01	(254.6	0 (0,0)	(3236.6	(0.1	(406.5	(0	(33.6	(-0.07	(48.5	(-0.21	(2159.7	(-0.04	(654.5	(-0.22	(515.7	(-0.27	(103.4	(0.01
	,15618.7)	,0.07)	,323.6)	,-0.02)	,5915.1)	,0.05)	,487.6)		,7575.9)	,0.17)	,605.8)	,0.03)	,114.6)	,-0.05)	,381.7)	,0.02)	,3367.2)	,-0.03)	,1102.1)	,-0.11)	,963.4)	,-0.13)	,179.1)	,0.04)
	14841.4	0.03	248.5	0.57	6229.8	-0.05	816		4915.1	0.17	485.9	0.22	230.1	0.03	65.9	-2.16	2709.7	0.01	1985.9	0.24	1835.3	0.23	154.1	0.37
**Trinidad and Tobago**	(12429.5	(-0.02	(195.3	(0.45	(4515.2	(-0.13	(596.5	0 (0,0)	(3100.2	(0.08	(392.5	(0.21	(130.9	(0.02	(7.7	(-2.4	(2165	(0	(1422	(0.05	(1272.9	(0.02	(121.7	(0.32
	,17830.6)	,0.09)	,314)	,0.69)	,8418.7)	,0.03)	,1090.8)		,7509.2)	,0.26)	,586)	,0.23)	,358.5)	,0.04)	,156)	,-1.91)	,3375.3)	,0.01)	,2655.2)	,0.42)	,2506.4)	,0.43)	,194.8)	,0.42)
	17759.6	0.04	222	0.11	8856.2	0.08	749.9	0.01	5140.1	0.21	586.5	0.18	99.6	-0.05	598.2	-1.39	2721	-0.03	463.3	-0.08	350.5	-0.26	113.2	0.55
**Tunisia**	(14930.7	(-0.01	(174.9	(0.1	(6489.7	(0	(548.9	(0.01	(3271.7	(0.13	(475.1	(0.17	(55.2	(-0.06	(259	(-1.45	(2174	(-0.04	(338.4	(-0.12	(233.2	(-0.31	(89.9	(0.51
	,21351.9)	,0.08)	,277.6)	,0.12)	,12031.3)	,0.16)	,999.1)	,0.01)	,7812.8)	,0.3)	,706.8)	,0.19)	,157.2)	,-0.03)	,939.7)	,-1.33)	,3389.8)	,-0.03)	,613.2)	,-0.05)	,498)	,-0.22)	,141.1)	,0.58)
	13661.9	0.09	223.9	0.14	5351	-0.11	754		4272	0.66	578	0.42	145.9	-0.02	387.6	-1.49	2701.8	0	480.5	-0.17	387.9	-0.22	93	0.04
**Turkey**	(11485.6	(0.03	(182.9	(0.13	(3909.1	(-0.17	(551.9	0 (0,0)	(2632.5	(0.47	(467.5	(0.4	(83.1	(-0.03	(106.6	(-1.56	(2158.2	(0	(340.6	(-0.2	(251.6	(-0.26	(70.4	(0
	,16437.9)	,0.16)	,270.9)	,0.16)	,7084.1)	,-0.05)	,1001.9)		,6490.2)	,0.85)	,694.1)	,0.43)	,229)	,-0.01)	,656.8)	,-1.41)	,3365.4)	,0.01)	,655.5)	,-0.13)	,562.2)	,-0.17)	,121.7)	,0.08)
	13409.2	-0.1	210.6	0.29	6886.2	-0.23	550		2956	0.12	598.1	0.26	61.6	0	136.3	-2.24	2663.7	0.03	2868.9	-0.57	2734.1	-0.58	138.7	-0.25
**Turkmenistan**	(11390.8	(-0.14	(166.7	(0.22	(5118.3	(-0.29	(399.2	0 (0,0)	(1792.3	(0.04	(488.5	(0.25	(29.8	(-0.03	(22.9	(-2.81	(2128.2	(0.03	(2079.4	(-0.91	(1942.8	(-0.94	(108.5	(-0.27
	,15997.3)	,-0.06)	,264.2)	,0.35)	,9138.9)	,-0.16)	,740.5)		,4610)	,0.19)	,715.3)	,0.27)	,102.2)	,0.03)	,284.1)	,-1.66)	,3315.7)	,0.04)	,3822.5)	,-0.22)	,3685.4)	,-0.22)	,174.1)	,-0.24)
	13605.8	0.03	244.1	0	5177.9	0.02	352.8		5080.9	0.07	462.4	0.23	67.5	0	239	-0.47	2688.5	0.01	901.3	-0.19	764.8	-0.21	137.5	-0.05
**Tuvalu**	(11309.7	(0.01	(192.1	(-0.02	(3838.3	(0	(250.8	0 (0,0)	(3219	(0.03	(371.6	(0.22	(33.1	(-0.03	(62.1	(-0.66	(2147.5	(0.01	(674.8	(-0.24	(542.9	(-0.27	(104	(-0.06
	,16446)	,0.04)	,304.8)	,0.02)	,6863.7)	,0.03)	,478.1)		,7677.8)	,0.1)	,560.6)	,0.25)	,113.2)	,0.02)	,424.5)	,-0.28)	,3348)	,0.01)	,1177.5)	,-0.13)	,1041.4)	,-0.15)	,177.6)	,-0.04)
	22374.1	-0.04	188.4	0.22	15540.7	-0.12	645.1		4071	0.35	576.1	0.08	22.2	-0.13	139.8	-0.73	2665.6	-0.06	1540.8	0.03	1440	0.03	102.4	0.04
**Uganda**	(18354.5	(-0.1	(145.9	(0.2	(11332.6	(-0.21	(468.4	0 (0,0)	(2560.2	(0.3	(462.8	(0.06	(9	(-0.14	(30.8	(-0.86	(2129.6	(-0.07	(1164.1	(-0.02	(1058.8	(-0.02	(78.9	(0.01
	,27552.3)	,0.03)	,239.6)	,0.23)	,20819.6)	,-0.02)	,871.3)		,6210.4)	,0.41)	,694.9)	,0.11)	,41.1)	,-0.11)	,325.8)	,-0.59)	,3318.3)	,-0.06)	,1998)	,0.08)	,1896.3)	,0.09)	,129.8)	,0.06)
	16218	-0.29	189.7	0.03	8520.4	-0.65	573		4285.4	0.1	634.5	0.19	50.5	0.02	199.8	0.01	2722.2	0.03	3259.5	-0.8	3095.1	-0.77	171.1	-1.28
**Ukraine**	(13785.3	(-0.36	(157.8	(-0.02	(6448.5	(-0.77	(448.2	0 (0,0)	(3014	(-0.02	(518.5	(0.17	(23.6	(0	(31.9	(-0.49	(2178.6	(0.03	(2426.9	(-1.01	(2253.1	(-0.99	(136.8	(-1.5
	,18913.3)	,-0.23)	,226.1)	,0.08)	,11062.9)	,-0.53)	,719.7)		,5939.1)	,0.22)	,761.8)	,0.21)	,87.3)	,0.04)	,393.9)	,0.5)	,3397.1)	,0.03)	,4252.3)	,-0.59)	,4096.2)	,-0.56)	,210.5)	,-1.06)
	13223.6	-0.08	252.2	-0.12	5226.8	-0.2	723.8	-0.05	3789.6	-0.32	675.3	0.65	40.3	0.26	251	0.78	2964.9	0.21	679.2	0.03	503.5	-0.23	176.6	0.89
**United Arab Emirates**	(11224.7	(-0.11	(200.7	(-0.14	(3831.9	(-0.25	(527.2	(-0.05	(2397.9	(-0.38	(549.2	(0.6	(19	(0.24	(41.9	(0.67	(2369.6	(0.19	(505.8	(-0.09	(336.1	(-0.33	(145	(0.68
	,15634.7)	,-0.05)	,315.6)	,-0.1)	,7087)	,-0.16)	,972)	,-0.04)	,5695.1)	,-0.26)	,815)	,0.7)	,68.7)	,0.28)	,461.1)	,0.88)	,3692.9)	,0.23)	,902.1)	,0.15)	,725.5)	,-0.13)	,212.6)	,1.1)
**United Kingdom**	15465.5	0.25	251.2	-0.12	6076.4	0.04	1179.9	0.05	5394	0.78	545.2	0.63	29.4	0.13	182.9	-1.18	2754.5	0.05	2140.1	1.78	1840.2	2.01	306.6	0.58
	(13704.7	(0.21	(210.4	(-0.22	(4895.2	(-0.02	(940.2	(0.04	(4162.5	(0.64	(444.5	(0.58	(12	(0.12	(44.7	(-1.26	(2222	(0.05	(1707.9	(1.65	(1412.7	(1.86	(255.2	(0.53

	,17434.1)	,0.29)	,297.5)	,-0.01)	,7442.3)	,0.1)	,1485.9)	,0.06)	,7003.7)	,0.93)	,650.8)	,0.68)	,55.5)	,0.15)	,339.8)	,-1.1)	,3392.5)	,0.06)	,2669.2)	,1.9)	,2371.6)	,2.16)	,363.6)	,0.63)
**United Republic of Tanzania**	17437.5 (14534.7,21287.3)	0.01 (-0.02,0.03)	193.4 (151.9,245.1)	0.19 (0.16,0.22)	10291.4 (7527.8,13956)	-0.05 (-0.08,-0.02)	646.3 (467.9,869.9)	0 (0,0)	3893.5 (2454.5,6042.7)	0.18 (0.14,0.22)	599.9 (482.1,725.6)	0.16 (0.14,0.19)	23 (9.2,42.2)	0.02 (0,0.04)	111.5 (18.2,282.2)	-0.64 (-0.83,-0.45)	2716.3 (2170.2,3383.9)	-0.01 (-0.01,0)	2244.5 (1644,2961.8)	0.14 (0.04,0.23)	2118.8 (1518.6,2831.3)	0.12 (0.02,0.21)	128.7 (103.6,159.5)	0.52 (0.48,0.56)
**United States of America**	14240 (12754.5,16015.3)	-0.07 (-0.17,0.03)	338.4 (282.5,399.5)	0.02 (-0.01,0.05)	4095.7 (3405,4909.2)	0.03 (-0.07,0.13)	607.7 (544,678.8)	0 (0,0.01)	6026 (4691.3,7765.2)	-0.26 (-0.51,-0.02)	816.6 (668.8,976)	0.28 (0.24,0.31)	73.8 (34.8,126.5)	0.01 (-0.15,0.17)	176.6 (32.1,351.6)	-0.64 (-0.67,-0.6)	2906.9 (2344.1,3623.5)	0.06 (0.06,0.06)	2294.9 (1852.9,2816.1)	1.19 (0.98,1.4)	1665.8 (1238.9,2171.7)	0.76 (0.53,0.99)	640.9 (545.9,758.1)	2.82 (2.7,2.94)
**United States Virgin Islands**	14503.2 (12201.4,17592.7)	0.11 (0.07,0.15)	259.7 (203.1,328.9)	0.16 (0.11,0.21)	5686.5 (4164.6,7625.7)	0.17 (0.12,0.22)	821.2 (600.9,1096.3)	0 (0,0)	5081.3 (3272.8,7847.3)	0.15 (0.08,0.22)	504 (406.5,609.1)	0.13 (0.12,0.14)	227.9 (129.6,355)	0.01 (0,0.02)	50 (6.6,129.3)	-1.66 (-2.13,-1.19)	2698.5 (2155.9,3360.4)	0.01 (0.01,0.01)	3405.3 (2513,4526.5)	0.33 (0.21,0.45)	3238.7 (2348.8,4368)	0.34 (0.21,0.47)	174 (137.6,218.8)	0.15 (0.12,0.18)
**Uruguay**	13360.7 (11217.4,15987)	0.11 (0.07,0.14)	268.8 (210.7,337.4)	0.05 (0.02,0.07)	4017.6 (2950,5489.4)	0.25 (0.17,0.34)	777.3 (568.1,1038.7)	-0.04 (-0.05,-0.03)	5363.2 (3392.4,7985.8)	0.13 (0.06,0.2)	743.8 (604.8,904)	0.17 (0.16,0.18)	48.8 (22.1,85.5)	0 (-0.01,0.01)	167.1 (32.8,334.2)	-1.1 (-1.25,-0.95)	2683.3 (2163.1,3311)	0 (-0.01,0)	1536.9 (1234.9,1907.3)	0.35 (0.15,0.55)	1342 (1045.3,1716.7)	0.43 (0.21,0.66)	197.9 (159.1,240.9)	-0.19 (-0.25,-0.13)
**Uzbekistan**	13153.5 (11197.8,15628.4)	-0.15 (-0.19,-0.11)	197.9 (155.9,248.5)	0.18 (0.14,0.22)	6862.6 (5105.8,9116.3)	-0.3 (-0.37,-0.23)	546.5 (393.3,733.4)	-0.01 (-0.01,0)	2608.3 (1614.5,3990)	-0.03 (-0.09,0.03)	610.7 (496,736.4)	0.28 (0.27,0.29)	62.5 (30.3,103.8)	0.05 (0.02,0.08)	206.2 (33.9,392.5)	-1.24 (-1.61,-0.86)	2682.7 (2143.2,3340.3)	0.05 (0.04,0.06)	1989.7 (1393.6,2736.2)	-2.38 (-2.9,-1.86)	1855.5 (1256.9,2598.3)	-2.51 (-3.06,-1.97)	136.8 (107.6,170.5)	0.06 (0.02,0.11)
**Vanuatu**	13314.7 (11236.9,16051.7)	0.05 (0.03,0.07)	239.8 (188.9,300.1)	-0.1 (-0.11,-0.09)	4890.1 (3708.4,6362.7)	-0.01 (-0.03,0.02)	352.6 (252.3,479.8)	-0.01 (-0.01,-0.01)	5009.5 (3274.1,7727.7)	0.19 (0.15,0.23)	462 (374,557.9)	-0.08 (-0.09,-0.07)	68.9 (33.7,115.3)	-0.12 (-0.13,-0.11)	247.7 (57.6,459.5)	0.23 (0.17,0.3)	2723.6 (2176.4,3392.6)	-0.06 (-0.06,-0.05)	897.9 (670.9,1161.1)	-0.19 (-0.22,-0.16)	774.1 (545,1035.2)	-0.22 (-0.25,-0.19)	124.7 (94.8,161.2)	-0.02 (-0.03,-0.01)
**Venezuela (Bolivarian Republic of)**	14314.1 (11907.5,17148.7)	0.03 (0.01,0.06)	241.2 (189.6,303.2)	0.03 (0,0.06)	6204.3 (4473,8291.3)	-0.06 (-0.1,-0.02)	747.2 (545.1,1005.2)	0 (0,0)	4395.3 (2709.4,6681.4)	0.2 (0.15,0.24)	531.6 (432.2,640.1)	0.2 (0.17,0.22)	179.8 (97.5,286.7)	-0.04 (-0.05,-0.02)	112 (13.6,245.6)	-0.41 (-0.63,-0.2)	2693.8 (2152.1,3354.3)	-0.01 (-0.01,-0.01)	1667.3 (1167,2292.6)	-1.34 (-1.5,-1.18)	1570.7 (1069.1,2205.3)	-1.4 (-1.57,-1.23)	98.6 (74.5,128.7)	-0.12 (-0.14,-0.09)
**Vietnam**	10952.8 (9398,12931.6)	-0.09 (-0.14,-0.04)	274.7 (219.1,340)	0.3 (0.27,0.32)	4371.3 (3319.9,5799.7)	-0.03 (-0.09,0.02)	415.1 (295.5,562)	0 (0,0)	2891 (1861,4469.9)	0.03 (-0.1,0.17)	522.3 (426.1,627.5)	0.36 (0.34,0.37)	49.4 (23.2,85.8)	0.01 (-0.01,0.02)	233.8 (88.1,400.2)	-3.27 (-3.44,-3.09)	2662.4 (2127,3314.3)	0 (0,0.01)	1155 (826.2,1551.8)	0.55 (0.48,0.61)	1038.7 (710.8,1430.9)	0.6 (0.53,0.68)	117.6 (90.2,150.5)	0.07 (0.04,0.11)
**Yemen**	15122.7 (12731.5,18012.8)	0.03 (0,0.05)	193.5 (150.6,244.3)	0.01 (-0.03,0.04)	6440 (4709.7,8833.6)	0.14 (0.12,0.16)	733.6 (536.4,982.3)	0 (0,0)	4458.6 (2820.7,6700.2)	-0.05 (-0.09,-0.01)	491.2 (397.5,591.8)	0.23 (0.19,0.27)	70.4 (37.4,113.7)	-0.08 (-0.1,-0.06)	928.5 (428.7,1439.5)	-0.45 (-0.68,-0.22)	2724.3 (2177,3392.5)	0.03 (0.02,0.04)	463.4 (344.9,617.9)	-0.07 (-0.09,-0.04)	361.3 (244.1,515.6)	-0.2 (-0.24,-0.17)	102.5 (79.5,129.4)	0.47 (0.45,0.49)
**Zambia**	16591 (13710.6,19832.9)	0.13 (0.09,0.18)	195.1 (153.2,247.6)	0.24 (0.19,0.28)	9197.8 (6710.8,12198.8)	0.12 (0.05,0.19)	644.4 (468.6,866.5)	0 (0,0.01)	4145.5 (2583,6336.7)	0.41 (0.35,0.47)	581.6 (469.5,702.3)	0.06 (0.01,0.11)	22.6 (9.2,41.7)	-0.13 (-0.15,-0.11)	76.3 (14.9,216.9)	0.3 (-0.11,0.71)	2697.9 (2155.4,3359.7)	-0.1 (-0.1,-0.09)	2329.6 (1734.3,3051.9)	-0.1 (-0.17,-0.04)	2219.8 (1624.4,2939.8)	-0.11 (-0.18,-0.04)	112.7 (89.7,140.1)	0.02 (0,0.04)
**Zimbabwe**	14513.2 (12196.1,17481.2)	0.11 (0.09,0.14)	186.9 (145.7,239.9)	-0.13 (-0.17,-0.09)	7557.6 (5621.8,10093.5)	0.2 (0.17,0.23)	601.1 (432.7,811.4)	0 (0,0)	3588.8 (2314.5,5514.2)	0.16 (0.1,0.22)	540.4 (433,654.2)	-0.37 (-0.41,-0.33)	21.5 (8.6,40.1)	-0.28 (-0.29,-0.27)	126.1 (30.4,293.5)	1.9 (1.54,2.26)	2646.5 (2115.9,3292.8)	-0.08 (-0.08,-0.07)	1581.2 (1149.5,2131.2)	-0.66 (-0.75,-0.56)	1353.8 (925.9,1898.8)	-0.74 (-0.84,-0.64)	232.2 (195.8,272.3)	-0.11 (-0.15,-0.08)

Note: EAPC-estimated annual percentage changes ; 95% CI-95% confidence intervals(EAPC); 95% UI-95% uncertainty interval

eTable 8. Age Standardised YLDs and EAPC of Mental disorders, Substance use disorders in people aged ≥60 years in 204 countries and territories, from 1990 to 2021

Location	Rate (95% UI/95%CI)																									
	Mental disorders		Schizophrenia		Depressive disorders		Bipolar disorder		Anxiety disorders		Autism spectrum disorders		Attention-deficit/ Hyperactivity disorder		Idiopathic developmental intellectual disability		Other mental disorders		Substance use disorders		Alcohol use disorders		Drug use disorders	
	2021	EAPC,%	2021	EAPC,%	2021	EAPC,%	2021	EAPC,%	2021	EAPC,%	2021	EAPC,%	2021	EAPC,%	2021	EAPC,%	2021	EAPC,%	2021	EAPC,%	2021	EAPC,%	2021	EAPC,%
Afghanistan	2143.4 (1530.2 ,2885)	0.1 (0.07 ,0.13)	104.6 (65.6 ,153.3)	-0.09 (-0.14 ,-0.03)	1079 (675.6 ,1592.4)	0.14 (0.11 ,0.17)	140.5 (80.5 ,219.5)	-0.04 (-0.05 ,-0.03)	522.5 (300.4 ,858.9)	0.31 (0.23 ,0.38)	73.5 (48.8 ,105.5)	-0.12 (-0.15 ,-0.1)	1 (0.4 ,2)	-0.35 (-0.39 ,-0.31)	42.7 (19.7 ,70.7)	-0.46 (-0.8 ,-0.13)	179.6 (115.5 ,268.1)	-0.17 (-0.18 ,-0.16)	76.6 (48.8 ,108.9)	0.05 (-0.01 ,0.1)	34.9 (19.2 ,56.2)	-0.55 (-0.59 ,-0.52)	41.7 (23.5 ,64.3)	0.63 (0.55 ,0.72)
Albania	1870.1 (1323.8 ,2534.2)	0.13 (0.08 ,0.18)	127.6 (82.3 ,179.4)	0.23 (0.21 ,0.26)	739.5 (469.5 ,1120.7)	0.11 (0.05 ,0.18)	111 (63.8 ,174.3)	0.02 (0.01 ,0.03)	567.2 (317.2 ,942.4)	0.15 (0.06 ,0.24)	125.7 (85.3 ,177.3)	0.44 (0.4 ,0.48)	0.7 (0.2 ,1.5)	-0.01 (-0.04 ,0.01)	8.8 (1.4 ,18.9)	-2.04 (-2.27 ,-1.8)	189.6 (122.4 ,284.5)	0.04 (0.04 ,0.05)	221 (138.5 ,337)	0.12 (-0.07 ,0.3)	183 (106.7 ,291.3)	-0.17 (-0.36 ,0.01)	38 (22 ,58.2)	2.15 (1.8 ,2.5)
Algeria	2046.5 (1442.3 ,2756.7)	-0.02 (-0.05 ,0.01)	131.9 (83.7 ,187.1)	0.05 (0.03 ,0.07)	966.3 (598.3 ,1519)	-0.07 (-0.11 ,-0.02)	147.7 (85.9 ,232.9)	-0.03 (-0.04 ,-0.03)	488.9 (264.5 ,786.4)	0.02 (-0.02 ,0.07)	99.4 (66 ,140.5)	0.32 (0.31 ,0.32)	1.2 (0.4 ,2.2)	0.06 (0.04 ,0.09)	21.4 (7.1 ,39.9)	-0.97 (-1.11 ,-0.82)	189.6 (121.5 ,284.6)	0 (-0.01 ,0.01)	63.7 (39.5 ,92.3)	-0.01 (-0.04 ,0.02)	33.3 (17.9 ,54.5)	-0.36 (-0.38 ,-0.33)	30.4 (15.9 ,48.5)	0.41 (0.36 ,0.45)
American Samoa	1650.2 (1188.6 ,2216.7)	-0.05 (-0.07 ,-0.03)	160.7 (106 ,221.7)	-0.25 (-0.27 ,-0.24)	607.5 (393.8 ,903.8)	-0.06 (-0.09 ,-0.02)	70.3 (38.8 ,113.1)	-0.04 (-0.05 ,-0.03)	530.5 (302.3 ,884.8)	-0.01 (-0.04 ,0.03)	88.8 (60.1 ,128.1)	0.06 (0.04 ,0.07)	0.8 (0.3 ,1.6)	-0.06 (-0.09 ,-0.03)	5.5 (1.2 ,12.1)	0.89 (0.74 ,1.05)	186.2 (117.1 ,279)	-0.03 (-0.04 ,-0.03)	96.4 (61.9 ,140)	-0.33 (-0.35 ,-0.31)	67.8 (39.8 ,105.3)	-0.51 (-0.54 ,-0.48)	28.6 (15.6 ,46.8)	0.12 (0.09 ,0.15)
Andorra	2195.3 (1557.4 ,2955.7)	0 (-0.04 ,0.05)	182.9 (121.8 ,253.4)	-0.04 (-0.05 ,-0.03)	822.8 (510.3 ,1239.1)	-0.1 (-0.18 ,-0.02)	212.3 (126.6 ,331.7)	0.07 (0.06 ,0.09)	655.8 (373.6 ,1051.9)	0.11 (0.04 ,0.18)	121.7 (81.6 ,172.1)	0.13 (0.11 ,0.16)	0.7 (0.2 ,1.5)	0.02 (-0.01 ,0.06)	5.4 (1.2 ,11.9)	-0.75 (-0.93 ,-0.58)	193.8 (123.4 ,287.5)	-0.03 (-0.03 ,-0.02)	160.9 (99.7 ,235.9)	-0.18 (-0.27 ,-0.1)	131.4 (77.5 ,200.2)	-0.29 (-0.39 ,-0.19)	29.5 (16.3 ,46.4)	0.29 (0.19 ,0.4)
Angola	2769.7 (1886.3 ,3862.6)	0.05 (0.02 ,0.08)	120 (76 ,169.2)	0.26 (0.21 ,0.3)	1813.2 (1107.1 ,2779.2)	0.02 (-0.02 ,0.06)	119.3 (67.9 ,188.2)	0 (-0.01 ,0.01)	433.8 (243.6 ,742.9)	0.16 (0.11 ,0.21)	97.4 (64.7 ,137.8)	0.18 (0.15 ,0.2)	0.3 (0.1 ,0.6)	-0.07 (-0.1 ,-0.04)	1.8 (0.3 ,6.1)	-1.12 (-1.43 ,-0.81)	184.1 (117.2 ,275.2)	-0.05 (-0.05 ,-0.04)	169.2 (105.9 ,246.1)	-0.01 (-0.08 ,0.07)	144.8 (86.3 ,217.6)	-0.06 (-0.14 ,0.02)	24.4 (13.2 ,39.5)	0.33 (0.31 ,0.36)
Antigua and Barbuda	1983 (1404.2 ,2680.1)	0.02 (-0.05 ,0.08)	146.2 (94.1 ,207.6)	0.05 (0.03 ,0.07)	833.4 (520.8 ,1295)	0 (-0.12 ,0.12)	165 (95.7 ,258.1)	-0.04 (-0.05 ,-0.04)	556.4 (311.7 ,912.1)	0.03 (-0.04 ,0.1)	88.3 (58.8 ,127)	0.19 (0.18 ,0.2)	2.6 (1.2 ,4.7)	0.07 (0.05 ,0.09)	3.4 (0.3 ,8.5)	-0.57 (-0.68 ,-0.46)	187.6 (120.2 ,281.3)	0 (0 ,0.01)	321.9 (201 ,471.7)	0.13 (0 ,0.25)	300.2 (182.6 ,446.7)	0.14 (0 ,0.29)	21.7 (11.7 ,35.4)	-0.11 (-0.16 ,-0.05)
Argentina	1740.5 (1247 ,2276.3)	-0.17 (-0.23 ,-0.11)	158.9 (101.6 ,221.1)	0 (-0.02 ,0.02)	529.9 (337.1 ,785.3)	-0.6 (-0.7 ,-0.5)	158.1 (91.1 ,243.6)	-0.08 (-0.09 ,-0.06)	568.4 (312 ,917.5)	0.06 (-0.03 ,0.15)	127.2 (86 ,180.2)	0.19 (0.18 ,0.2)	0.6 (0.1 ,1.2)	-0.03 (-0.07 ,0.01)	9.6 (2.6 ,19)	-0.79 (-0.91 ,-0.67)	187.9 (119.4 ,280.5)	0 (-0.01 ,0.01)	167.2 (112.6 ,233.4)	-1.16 (-1.3 ,-1.02)	128.6 (82.7 ,188)	-1.44 (-1.61 ,-1.27)	38.6 (22.1 ,58.8)	-0.02 (-0.07 ,0.03)
Armenia	2109 (1492.3 ,2890.8)	0.21 (0.17 ,0.26)	122.2 (78.8 ,174.2)	0.22 (0.18 ,0.27)	1161.5 (738.9 ,1730.6)	0.33 (0.27 ,0.39)	112.5 (63 ,176.6)	0.01 (0 ,0.02)	400.4 (223.7 ,666.7)	0.11 (0.01 ,0.2)	115.9 (78 ,164.2)	0.27 (0.26 ,0.28)	0.7 (0.2 ,1.5)	-0.01 (-0.05 ,0.03)	9.7 (2 ,20.1)	-1.99 (-2.41 ,-1.57)	186.1 (119.1 ,278.3)	0.03 (0.02 ,0.03)	294.9 (184 ,429.5)	-0.85 (-1.03 ,-0.68)	258.6 (154.8 ,385.9)	-0.9 (-1.1 ,-0.71)	36.3 (19.5 ,58)	-0.48 (-0.58 ,-0.39)
Australia	1979.8 (1448.6 ,2600.6)	0.04 (0.01 ,0.08)	211.5 (147.2 ,276.8)	0.02 (0.01 ,0.03)	694.8 (431.5 ,1054.6)	-0.04 (-0.23 ,0.14)	199.2 (121.1 ,298.4)	-0.01 (-0.01 ,0)	486.6 (271.4 ,792.9)	0.11 (-0.13 ,0.36)	167.9 (114.1 ,236.6)	0.29 (0.26 ,0.32)	2.1 (0.9 ,3.9)	-0.01 (-0.04 ,0.02)	6 (1.6 ,12.9)	1.62 (0.97 ,2.28)	211.8 (136.8 ,303.1)	0.01 (0 ,0.01)	179 (121 ,254.1)	0.27 (0.23 ,0.3)	135.3 (85.4 ,202.9)	0.21 (0.18 ,0.25)	43.7 (26 ,65.5)	0.44 (0.38 ,0.49)
Austria	2088.2 (1476 ,2808.2)	-0.11 (-0.17 ,-0.06)	178.6 (117.8 ,249.6)	-0.01 (-0.02 ,0.01)	660.1 (409.4 ,985.6)	-0.46 (-0.55 ,-0.37)	212.3 (124.7 ,323.8)	0.07 (0.05 ,0.08)	727.3 (406.2 ,1149.1)	0.01 (-0.07 ,0.09)	113 (76.2 ,160.6)	0.44 (0.4 ,0.48)	0.8 (0.3 ,1.8)	0.14 (0.1 ,0.18)	6.6 (1.7 ,14)	-1.52 (-1.61 ,-1.43)	189.4 (120.1 ,278.1)	0.1 (0.09 ,0.11)	294.7 (193.6 ,418.9)	1.7 (1.55 ,1.86)	261.6 (167.8 ,381.2)	1.85 (1.68 ,2.02)	33.1 (18.6 ,50.6)	0.74 (0.55 ,0.93)

	1913.1	0.04	125.1	0.25	1028.2	0.03	111.9	-0.02	342.8	-0.01	109.3	0.34	0.7	0.06	7.6	-1.41	187.5	0.05	290.9	-0.32	257.6	-0.31	33.2	-0.42
Azerbaijan	(1329.5	(-0.01	(81.1	(0.16	(635.1	(-0.06	(63.9	(-0.02	(190.4	(-0.09	(73.8	(0.32	(0.2	(0.02	(1.5	(-2.11	(119.7	(0.04	(181.9	(-0.52	(153.6	(-0.53	(17.4	(-0.47
	,2616.1)	,0.1)	,176)	,0.33)	,1576.6)	,0.11)	,176.1)	,-0.01)	,569.6)	,0.06)	,155.2)	,0.35)	,1.5)	,0.09)	,16.3)	,-0.7)	,281.7)	,0.06)	,438.3)	,-0.13)	,398)	,-0.09)	,53.5)	,-0.36)
	2014.5	0.03	146.5	-0.02	854	0.01	164.5	-0.03	573.9	0.1	82.9	0.15	2.5	0	2.9	0.19	187.2	-0.01	238.3	-0.15	216.2	-0.15	22.1	-0.17
Bahamas	(1408.8	(-0.03	(93.5	(-0.04	(523.4	(-0.1	(95.6	(-0.04	(322.5	(0.01	(55	(0.12	(1.2	(-0.02	(0.3	(0	(120.8	(-0.01	(151.1	(-0.22	(132.4	(-0.23	(11.5	(-0.24
	,2689.1)	,0.09)	,208.1)	,-0.01)	,1306.7)	,0.11)	,258.6)	,-0.03)	,964.4)	,0.18)	,119.4)	,0.17)	,4.7)	,0.02)	,7.6)	,0.38)	,278.8)	,0)	,349.1)	,-0.08)	,322.4)	,-0.07)	,36.1)	,-0.11)
	2207.1	-0.19	140.3	0.05	1100.4	-0.37	144.8	-0.07	509.9	-0.06	107.3	0.38	1.2	0.13	14.6	-0.29	188.7	0	63.1	-0.57	37.8	-0.89	25.3	-0.03
Bahrain	(1546.3	(-0.24	(90.5	(0.04	(687.5	(-0.45	(84	(-0.07	(281.3	(-0.11	(72.3	(0.34	(0.4	(0.08	(4.4	(-0.44	(120.4	(-0.01	(38.8	(-0.62	(20.3	(-0.94	(12.6	(-0.13
	,2928.1)	,-0.14)	,199.3)	,0.07)	,1648.3)	,-0.3)	,224.1)	,-0.06)	,851.6)	,0)	,153.6)	,0.41)	,2.3)	,0.18)	,27.5)	,-0.14)	,280.7)	,0.02)	,92.6)	,-0.52)	,59.8)	,-0.85)	,42.7)	,0.06)
	2550	0.25	166.2	0.1	1558.3	0.34	95.6	0.02	462.2	0.26	72	0.28	0.5	-0.09	4.3	-4.44	190.8	-0.02	152.3	-0.3	127.6	-0.41	24.7	0.3
Bangladesh	(1744.1	(0.2	(106.1	(0.08	(965.1	(0.27	(52.8	(0	(256.1	(0.19	(46.8	(0.26	(0.2	(-0.12	(0.4	(-4.65	(121.5	(-0.03	(92	(-0.34	(72.8	(-0.44	(12.5	(0.21
	,3531.6)	,0.3)	,231.8)	,0.11)	,2360.2)	,0.41)	,153.4)	,0.03)	,761.1)	,0.33)	,102.5)	,0.3)	,1.1)	,-0.05)	,11)	,-4.22)	,286.8)	,-0.02)	,231.2)	,-0.26)	,201.9)	,-0.37)	,41.7)	,0.39)
	2080	0.07	155.1	-0.05	906.7	0.13	165.7	-0.04	570.3	0.04	88.1	0.18	2.6	0.1	4.2	0.47	187.5	0.01	182	0.01	161.3	0.05	20.7	-0.31
Barbados	(1480.9	(0.02	(100.4	(-0.08	(569	(0.05	(95.7	(-0.04	(327.2	(-0.04	(58	(0.17	(1.2	(0.08	(0.3	(0.28	(119.3	(0.01	(115.6	(-0.07	(98.7	(-0.04	(10.9	(-0.38
	,2830.4)	,0.11)	,215)	,-0.02)	,1387.8)	,0.2)	,257.4)	,-0.03)	,938.4)	,0.11)	,125)	,0.19)	,4.7)	,0.12)	,10.2)	,0.66)	,279.7)	,0.02)	,270.5)	,0.08)	,246.2)	,0.13)	,34.6)	,-0.24)
	2527.8	-0.02	117.2	0.18	1422.2	-0.1	111.2	0	573.1	0.08	116.6	0.35	0.7	0.01	5.8	-1.58	181	0.02	620.9	1.46	549.7	1.57	71.2	0.73
Belarus	(1738.1	(-0.08	(74.7	(0.14	(895	(-0.18	(63.3	(0	(315.7	(-0.04	(79.4	(0.32	(0.2	(-0.03	(0.8	(-1.97	(115.8	(0.02	(411.4	(1.25	(352.4	(1.35	(45.1	(0.47
	,3396)	,0.05)	,165.8)	,0.23)	,2124.4)	,-0.03)	,176.5)	,0.01)	,915.1)	,0.2)	,164.3)	,0.38)	,1.4)	,0.05)	,14)	,-1.2)	,270.2)	,0.03)	,885.5)	,1.68)	,799.7)	,1.79)	,103.9)	,0.99)
	2077.4	0.15	175.5	-0.03	767.6	0.28	210	0.05	613.4	0.1	113	0.3	0.7	0.03	7.3	-1.37	189.9	0.04	254.5	1.05	208.2	0.98	46.2	1.34
Belgium	(1495.4	(0.07	(115.4	(-0.04	(486	(0.14	(123.1	(0.04	(355.2	(-0.01	(74.9	(0.29	(0.2	(-0.01	(2	(-1.46	(120	(0.04	(168.8	(0.97	(131.7	(0.88	(28.2	(1.25
	,2720)	,0.22)	,243.4)	,-0.02)	,1149.5)	,0.42)	,328.8)	,0.06)	,1022.7)	,0.2)	,159.4)	,0.31)	,1.4)	,0.07)	,14.6)	,-1.28)	,280.7)	,0.05)	,364.2)	,1.12)	,311.4)	,1.09)	,69.2)	,1.42)
	2037.4	0.08	137.9	0.03	914.7	0.13	163.9	-0.04	534.6	0.09	84.8	0.13	2.7	-0.04	7	-0.64	191.8	-0.02	213.4	0.11	192.7	0.14	20.7	-0.19
Belize	(1457.3	(0.03	(88.5	(0.01	(578.3	(0.06	(95.4	(-0.05	(307.8	(0.01	(56.3	(0.11	(1.2	(-0.07	(1.3	(-0.74	(122.1	(-0.02	(133.7	(0.02	(117.3	(0.04	(11.1	(-0.23
	,2720.2)	,0.13)	,193.9)	,0.04)	,1400.5)	,0.21)	,257.6)	,-0.04)	,891)	,0.16)	,120.9)	,0.15)	,4.9)	,-0.01)	,15.2)	,-0.55)	,289.3)	,-0.01)	,315.3)	,0.2)	,291.3)	,0.24)	,32.6)	,-0.14)
	2594.8	0.22	120.6	0.15	1733.3	0.28	113.4	0.02	336	0.16	101.4	0.16	0.3	0.02	3	0.59	186.8	-0.01	100.5	0.13	80.8	0.16	19.7	0.03
Benin	(1799	(0.2	(76.7	(0.13	(1093.4	(0.25	(64.3	(0.01	(186	(0.13	(66.7	(0.14	(0.1	(-0.02	(0.4	(0.4	(118.8	(-0.01	(63.8	(0.07	(48.3	(0.08	(9.4	(-0.01
	,3609.2)	,0.23)	,170.9)	,0.16)	,2641.1)	,0.3)	,181)	,0.03)	,557.7)	,0.19)	,146)	,0.17)	,0.6)	,0.06)	,9.6)	,0.79)	,277.4)	,0)	,149.3)	,0.19)	,125.7)	,0.23)	,33.2)	,0.07)
	2130.3	-0.1	159.9	0.1	944.2	-0.31	170.1	-0.02	565.5	0.08	97.7	0.3	2.6	0.02	1.8	-1.5	188.4	0	177.2	-0.34	149.4	-0.37	27.9	-0.14
Bermuda	(1468.2	(-0.15	(102.1	(0.08	(569.8	(-0.39	(99	(-0.03	(310.1	(0.02	(65.3	(0.27	(1.1	(0	(0.1	(-1.66	(119.9	(0	(114.5	(-0.43	(92.1	(-0.5	(15.4	(-0.3
	,2891.4)	,-0.06)	,226.3)	,0.12)	,1455.3)	,-0.24)	,266.4)	,-0.02)	,928.6)	,0.14)	,138.3)	,0.32)	,4.8)	,0.03)	,5.6)	,-1.34)	,280.4)	,0.01)	,259.9)	,-0.25)	,226.1)	,-0.25)	,43.9)	,0.02)
	2423	-0.08	176.1	0.22	1413.9	-0.17	95.5	0.01	448.4	0.05	76.9	0.36	0.4	0	22	-1.57	189.8	0.02	333.3	0.24	306.1	0.24	27.1	0.25
Bhutan	(1685.4	(-0.12	(113.6	(0.2	(878.7	(-0.23	(53.7	(0	(253.2	(0.03	(51.4	(0.34	(0.1	(-0.04	(8.7	(-1.66	(121.5	(0.01	(201.7	(0.19	(180.7	(0.18	(13.6	(0.2
	,3317.8)	,-0.04)	,247.5)	,0.23)	,2137.8)	,-0.11)	,151.2)	,0.01)	,739.2)	,0.07)	,109.4)	,0.37)	,1)	,0.03)	,39.9)	,-1.48)	,280.4)	,0.03)	,501.4)	,0.29)	,467.9)	,0.3)	,45.1)	,0.29)
	2469.9	0.02	136.4	0.1	1161.3	-0.09	162.1	-0.02	736.6	0.21	76.3	0.31	1.8	0.01	8.4	-1.51	186.9	0	219	-0.38	193.3	-0.43	25.7	0.09
Bolivia (Plurinational State of)	(1711.9	(-0.05	(86.6	(0.09	(715	(-0.18	(96.7	(-0.02	(423.1	(0.07	(51.3	(0.29	(0.7	(-0.01	(2	(-1.58	(119.6	(-0.01	(139.4	(-0.53	(120	(-0.6	(14.6	(0.06
	,3391.7)	,0.1)	,193.1)	,0.11)	,1781.6)	,0)	,254.4)	,-0.01)	,1193.5)	,0.35)	,108.7)	,0.33)	,3.4)	,0.03)	,16.8)	,-1.45)	,277.8)	,0.01)	,323.2)	,-0.23)	,295.6)	,-0.26)	,40.2)	,0.13)
	2064.9	-0.23	124.1	0.34	968.9	-0.53	108.6	-0.02	546.5	0.01	124.3	0.4	0.7	0.03	7.8	-3.37	184	0.02	271.6	-1.93	253.4	-2.04	18.3	0.36
Bosnia and Herzegovina	(1473.6	(-0.3	(78.6	(0.3	(595.5	(-0.64	(61.4	(-0.03	(309.5	(-0.07	(85	(0.35	(0.2	(0	(1	(-3.63	(117.4	(0.01	(169.9	(-2.07	(152.6	(-2.19	(8.4	(0.32
	,2817.9)	,-0.16)	,174.1)	,0.38)	,1497.6)	,-0.42)	,170.1)	,-0.01)	,900.7)	,0.09)	,177.9)	,0.44)	,1.5)	,0.06)	,17.1)	,-3.11)	,274.7)	,0.02)	,406.2)	,-1.78)	,384.9)	,-1.89)	,31.5)	,0.4)
Botswana	2436.3	0.11	126.4	0.22	1453.3	0.13	118.4	-0.04	452.6	0.13	100.8	0.16	0.3	-0.03	2.1	-0.8	182.3	-0.06	254.1	-0.07	208.9	-0.18	45.2	0.47
	(1685.2	(0.08	(80.5	(0.2	(882.2	(0.08	(67.6	(-0.05	(252.4	(0.07	(67.1	(0.1	(0.1	(-0.06	(0.2	(-1.45	(116.4	(-0.07	(162	(-0.15	(126.8	(-0.28	(27.2	(0.43

	,3379.4)	,0.15)	,178.3)	,0.24)	,2244.6)	,0.19)	,185.1)	,-0.03)	,752)	,0.2)	,142.9)	,0.21)	,0.6)	,0)	,5.9)	,-0.14)	,269.5)	,-0.04)	,368.9)	,0.01)	,318.2)	,-0.08)	,67.3)	,0.5)
	2615	0.13	139.3	0.14	1058.9	-0.25	190.2	0.01	956.8	0.65	71.8	0.18	1.4	-0.03	6.4	-1.05	190.2	-0.01	283.3	-0.02	254	-0.04	29.2	0.16
Brazil	(1882.2	(0	(99.9	(0.12	(697.9	(-0.33	(119.9	(0	(640.6	(0.36	(48.9	(0.18	(0.6	(-0.04	(1.7	(-1.1	(122.5	(-0.01	(180.8	(-0.22	(157.9	(-0.27	(19.2	(0.09
	,3412.4)	,0.25)	,179.6)	,0.15)	,1466.8)	,-0.16)	,279.1)	,0.02)	,1372.8)	,0.93)	,100)	,0.19)	,2.6)	,-0.02)	,12.9)	,-1)	,284.9)	,0)	,405.7)	,0.18)	,370.2)	,0.19)	,40.9)	,0.23)
	1303.3	0.05	150.8	-0.06	355.2	0.12	122.4	0.01	317.6	0.09	167.8	0.09	0.6	-0.06	0.5	-4.18	188.5	-0.04	112.9	-1.43	77	-0.66	35.9	-2.75
Brunei Darussalam	(958.8	(0.03	(97	(-0.08	(217.3	(0.06	(69.1	(0.01	(177.4	(0.05	(113.2	(0.07	(0.1	(-0.1	(0	(-4.69	(120.5	(-0.05	(70.9	(-1.53	(43.6	(-0.7	(20.9	(-3.03
	,1673.4)	,0.07)	,211.7)	,-0.04)	,539.3)	,0.19)	,192)	,0.02)	,519.5)	,0.13)	,236.5)	,0.12)	,1.4)	,-0.02)	,2.1)	,-3.67)	,280.6)	,-0.03)	,163.8)	,-1.32)	,121.5)	,-0.62)	,54.8)	,-2.46)
	2160	-0.17	128.6	0.16	1039.2	-0.44	94.9	0.01	588.1	0.15	116.7	0.11	0.7	-0.04	7	-0.89	184.8	-0.02	215.6	-0.91	189.9	-1.05	25.7	0.43
Bulgaria	(1527.5	(-0.25	(82.2	(0.12	(645.7	(-0.56	(53.6	(0.01	(327.2	(0.03	(78.2	(0.08	(0.2	(-0.08	(1.2	(-1.15	(117.8	(-0.03	(133.2	(-1.01	(111.3	(-1.17	(14.3	(0.41
	,2913.9)	,-0.09)	,180.9)	,0.19)	,1552.1)	,-0.32)	,149.8)	,0.02)	,986)	,0.26)	,164.6)	,0.15)	,1.5)	,0)	,15.1)	,-0.63)	,277.3)	,-0.01)	,328)	,-0.8)	,300.7)	,-0.93)	,41.2)	,0.45)
	2605.4	0.08	117.7	0.19	1751	0.07	114.2	0.05	327.5	0.12	100.1	0.14	0.3	-0.03	5.3	1.19	189.2	0.03	115.9	0.11	91.2	0.05	24.7	0.34
Burkina Faso	(1800.8	(0.04	(74.5	(0.17	(1094.3	(0.01	(65	(0.04	(180.7	(0.09	(67	(0.13	(0.1	(-0.07	(0.6	(1.01	(121	(0.03	(74.4	(0.03	(55.3	(-0.05	(12.7	(0.29
	,3650.5)	,0.13)	,167.6)	,0.21)	,2662)	,0.14)	,181)	,0.06)	,554.9)	,0.14)	,143.2)	,0.16)	,0.6)	,0.01)	,14.2)	,1.37)	,279.6)	,0.04)	,167.5)	,0.2)	,138.9)	,0.16)	,39.8)	,0.39)
	2757.6	-0.38	105	-0.04	1802.1	-0.62	130	0.02	416.9	0.08	101.8	0.41	0.3	0.18	9.3	1.43	192.1	0.12	228.7	0.11	206.2	0.1	22.5	0.17
Burundi	(1885.5	(-0.44	(65.6	(-0.06	(1133	(-0.71	(75.2	(0.01	(228.6	(0.03	(68	(0.38	(0.1	(0.15	(1.2	(1.31	(123.3	(0.11	(141.8	(0.05	(124.4	(0.04	(11.6	(0.14
	,3826)	,-0.32)	,154.4)	,-0.02)	,2737)	,-0.54)	,201.4)	,0.03)	,691.4)	,0.14)	,144.4)	,0.44)	,0.6)	,0.21)	,21.6)	,1.54)	,287.4)	,0.13)	,338.2)	,0.16)	,311.4)	,0.16)	,37.6)	,0.2)
	2841.3	0.26	133.6	0.23	1941.5	0.34	115.6	-0.03	349.6	0.12	112.7	0.2	0.3	-0.03	3.5	0.5	184.5	-0.06	95.6	0.36	74	0.43	21.6	0.18
Cabo Verde	(1956.7	(0.2	(87.5	(0.21	(1232	(0.26	(66.5	(-0.04	(198.3	(0.06	(75.9	(0.17	(0.1	(-0.08	(0.4	(0.16	(117	(-0.07	(58.6	(0.3	(42.5	(0.34	(10.7	(0.13
	,3950.7)	,0.31)	,190.7)	,0.25)	,2939.9)	,0.42)	,183.3)	,-0.02)	,597.6)	,0.18)	,159.3)	,0.24)	,0.6)	,0.01)	,8.8)	,0.85)	,275)	,-0.04)	,141.7)	,0.43)	,116.9)	,0.51)	,35.8)	,0.22)
	1847.9	0.01	138.5	0.23	738.3	-0.13	80.9	0.01	615	0.21	77.6	0.28	0.6	-0.03	14.7	-2.31	182.2	-0.02	91.6	-0.43	72.7	-0.53	18.9	-0.05
Cambodia	(1307.8	(-0.01	(91	(0.19	(478	(-0.17	(44.6	(0	(336.2	(0.15	(51.5	(0.26	(0.2	(-0.06	(4.6	(-2.85	(116.2	(-0.02	(57.1	(-0.46	(43	(-0.57	(8.6	(-0.12
	,2488.9)	,0.04)	,190.5)	,0.28)	,1089.9)	,-0.09)	,131.9)	,0.02)	,1004.9)	,0.26)	,111.3)	,0.29)	,1.1)	,0)	,28.3)	,-1.76)	,273.3)	,-0.01)	,134.6)	,-0.4)	,113.8)	,-0.48)	,33.6)	,0.02)
	2661.8	0.18	121.1	0.09	1800.6	0.23	113.1	0.03	333.3	0.09	103.1	0.19	0.3	0.01	2.5	1.63	187.9	0.01	108.1	0.14	83.4	0.11	24.7	0.23
Cameroon	(1788.5	(0.16	(75.8	(0.06	(1109.2	(0.2	(64.5	(0.02	(183.1	(0.05	(68.4	(0.15	(0.1	(-0.02	(0.4	(1.39	(119.5	(0	(69	(0.07	(49.5	(0.03	(13.1	(0.2
	,3654.4)	,0.2)	,170.2)	,0.12)	,2663.2)	,0.27)	,174.9)	,0.03)	,556.6)	,0.12)	,145.8)	,0.23)	,0.6)	,0.05)	,8.3)	,1.87)	,281.4)	,0.02)	,163.2)	,0.2)	,132.8)	,0.19)	,39.4)	,0.26)
	1626.3	-0.17	172.2	-0.03	486.9	-0.64	138.6	0.05	465.6	-0.03	151.2	0.22	1.1	0.03	11	-0.67	199.7	0.03	229.4	0.45	132.6	0.48	96.8	0.41
Canada	(1181.1	(-0.26	(123.2	(-0.03	(300.8	(-0.87	(82.2	(0.04	(263.2	(-0.13	(102	(0.2	(0.3	(0	(3.2	(-0.71	(127	(0.03	(159.3	(0.36	(83.7	(0.35	(62.6	(0.35
	,2139.8)	,-0.08)	,220.6)	,-0.02)	,734.5)	,-0.41)	,214.5)	,0.07)	,764)	,0.07)	,215.9)	,0.25)	,2.1)	,0.07)	,21.7)	,-0.64)	,294.6)	,0.04)	,310.1)	,0.55)	,193.7)	,0.62)	,135)	,0.48)
	2732.1	0.06	99.8	-0.03	1789.8	0.03	118.1	0.02	450.1	0.25	86.1	0.01	0.3	-0.07	6.6	1.51	181.4	-0.01	161.1	-0.25	140.2	-0.29	20.9	0.01
Central African Republic	(1879.8	(0.04	(61.4	(-0.05	(1136.2	(0	(67.5	(0.01	(249.7	(0.2	(57.2	(-0.01	(0.1	(-0.1	(1.3	(1.36	(116.6	(-0.01	(103.2	(-0.35	(85.2	(-0.4	(10.2	(-0.01
	,3806.3)	,0.09)	,146.2)	,-0.02)	,2708.5)	,0.06)	,184.7)	,0.03)	,743.3)	,0.3)	,123.9)	,0.03)	,0.6)	,-0.03)	,16.6)	,1.66)	,269.1)	,0)	,235.1)	,-0.16)	,212)	,-0.19)	,35.1)	,0.04)
	2896.2	0.13	115.6	0.16	2044.1	0.14	112.8	0.01	325.2	0.06	102.8	0.29	0.3	0.21	2.9	-0.94	192.4	0.09	100.7	0.19	82	0.29	18.7	-0.2
Chad	(1986.8	(0.02	(72.8	(0.13	(1267.1	(-0.02	(64.6	(0	(185.4	(0.02	(68	(0.26	(0.1	(0.18	(0.5	(-1.05	(122.1	(0.07	(62.2	(0.13	(48	(0.21	(9	(-0.23
	,4031.6)	,0.23)	,166.9)	,0.19)	,3093.2)	,0.3)	,177)	,0.02)	,534.5)	,0.09)	,147.2)	,0.32)	,0.6)	,0.24)	,9.5)	,-0.84)	,291.5)	,0.1)	,149.8)	,0.25)	,127.1)	,0.36)	,32.3)	,-0.17)
	1994.1	-0.18	160.9	0.09	735.1	-0.61	155.7	-0.08	610.6	0.15	136.3	0.34	0.6	0.02	8	-2.04	186.9	-0.01	190.3	-0.4	156.4	-0.51	34	0.15
Chile	(1414.4	(-0.24	(103.2	(0.07	(442.9	(-0.73	(90	(-0.1	(352.6	(0.08	(91.9	(0.32	(0.1	(-0.01	(2	(-2.13	(118.4	(-0.01	(126.4	(-0.55	(99.6	(-0.69	(19	(0.09
	,2692.3)	,-0.12)	,224.7)	,0.1)	,1144.6)	,-0.5)	,246.7)	,-0.07)	,981)	,0.22)	,191.9)	,0.36)	,1.2)	,0.05)	,16.2)	,-1.96)	,276.7)	,0)	,269.7)	,-0.25)	,231.2)	,-0.34)	,51.7)	,0.21)
	1993.6	0.09	180.3	0.04	967.3	0.41	45	0	509	-0.56	88.9	0.59	1.4	-0.36	5.5	-1.55	196.2	0	131.5	-0.58	97.6	0.71	33.9	-3.12
China	(1475.4	(0.02	(132.4	(0.01	(670.3	(0.31	(28	(-0.01	(338.5	(-0.74	(60.3	(0.56	(0.6	(-0.43	(1.5	(-1.71	(126.5	(-0.01	(85.4	(-0.91	(59.1	(0.23	(21.8	(-3.31
	,2589.7)	,0.16)	,228.2)	,0.07)	,1333.9)	,0.52)	,67.5)	,0.01)	,732.9)	,-0.37)	,125.2)	,0.62)	,2.6)	,-0.3)	,11.6)	,-1.39)	,293.9)	,0.01)	,185)	,-0.25)	,144.1)	,1.19)	,49.2)	,-2.94)

	1823.7	-0.14	146.3	0.11	660.9	-0.41	134.3	0	592.5	-0.02	96.5	0.23	1.1	-0.06	4.2	-0.95	187.8	-0.02	215.8	-0.59	191.7	-0.64	24.1	-0.16
Colombia	(1269.6	(-0.25	(93.7	(0.09	(401.8	(-0.56	(79.7	(-0.01	(327	(-0.18	(64.8	(0.22	(0.4	(-0.08	(0.4	(-1.48	(118.4	(-0.02	(130.7	(-0.7	(111.7	(-0.76	(13.2	(-0.18
	,2444)	,-0.03)	,207.1)	,0.12)	,987.7)	,-0.25)	,202.9)	,0.01)	,974.3)	,0.15)	,136.9)	,0.25)	,2.3)	,-0.04)	,10.4)	,-0.41)	,282.2)	,-0.01)	,321.5)	,-0.48)	,295.1)	,-0.52)	,38.7)	,-0.13)
	2583.2	0.05	116.9	0.02	1621.5	0	130.5	0.02	421.5	0.26	101.7	0.12	0.3	-0.11	4.4	0.61	186.6	-0.03	189.2	-0.18	166.4	-0.26	22.9	0.4
Comoros	(1778	(-0.02	(73.3	(0	(1013.2	(-0.09	(75.4	(0.01	(240.5	(0.2	(68	(0.09	(0.1	(-0.15	(0.5	(0.52	(118.5	(-0.03	(122.3	(-0.24	(102.4	(-0.32	(11.9	(0.35
	,3591.1)	,0.11)	,165.8)	,0.04)	,2477.4)	,0.09)	,208.4)	,0.03)	,701.7)	,0.31)	,145.5)	,0.14)	,0.6)	,-0.07)	,12.2)	,0.71)	,279.9)	,-0.02)	,275.4)	,-0.13)	,249.2)	,-0.2)	,37.9)	,0.46)
	2720	-0.06	117.7	0.12	1779.1	-0.14	119.6	0	411.1	0.02	102	0.42	0.3	0.1	3.9	2.25	186.3	0.06	181	0.08	154.8	0	26.2	0.53
Congo	(1865	(-0.09	(74.6	(0.09	(1075.6	(-0.18	(69.2	(0	(235.7	(-0.03	(68.9	(0.39	(0.1	(0.07	(0.6	(1.77	(119.8	(0.05	(115.9	(-0.03	(93.5	(-0.12	(14.3	(0.46
	,3816.9)	,-0.03)	,168.4)	,0.16)	,2707)	,-0.1)	,186.7)	,0.01)	,683.9)	,0.07)	,142.2)	,0.46)	,0.6)	,0.14)	,10)	,2.73)	,277.5)	,0.07)	,269.9)	,0.18)	,238.8)	,0.12)	,41.3)	,0.6)
	1812.5	0.06	174.9	0	739.1	0.05	70.8	-0.03	538.2	0.14	98.3	0.09	0.8	-0.15	3.4	-0.58	186.9	-0.08	117.7	-0.56	92.1	-0.68	25.6	-0.12
Cook Islands	(1298.4	(0.04	(114.4	(-0.02	(464.1	(0.03	(38.7	(-0.04	(301.2	(0.11	(66.3	(0.07	(0.3	(-0.2	(0.6	(-0.71	(119	(-0.09	(75.3	(-0.6	(55.5	(-0.73	(13.5	(-0.17
	,2439.5)	,0.07)	,239.9)	,0.02)	,1115.1)	,0.08)	,113.9)	,-0.02)	,883.3)	,0.18)	,138.3)	,0.11)	,1.7)	,-0.1)	,8.6)	,-0.45)	,281.2)	,-0.07)	,169.5)	,-0.53)	,139.3)	,-0.63)	,41.6)	,-0.06)
	2115.2	0.12	147.9	0.08	1001.8	0.15	151.9	-0.02	519.4	0.18	101.3	0.09	1.2	-0.08	5.1	-0.41	186.6	-0.04	203.9	-0.51	183.5	-0.56	20.4	-0.05
Costa Rica	(1481.3	(0.08	(96.9	(0.07	(619.6	(0.1	(87.4	(-0.03	(285.1	(0.11	(68.4	(0.09	(0.4	(-0.11	(0.8	(-0.78	(119.8	(-0.05	(126.1	(-0.66	(110.3	(-0.72	(11.1	(-0.11
	,2856.3)	,0.16)	,207)	,0.1)	,1526.1)	,0.2)	,235.5)	,-0.02)	,873.6)	,0.26)	,143.7)	,0.1)	,2.4)	,-0.04)	,11.6)	,-0.04)	,278.9)	,-0.04)	,296.6)	,-0.35)	,272.6)	,-0.39)	,32.6)	,0.01)
	2222.5	-0.28	130.2	0.15	1107.6	-0.62	110	0	551	0.04	132	0.4	0.7	0.1	5.9	-1.57	185	0.06	417.5	-0.03	381.6	-0.11	35.9	0.99
Croatia	(1553	(-0.35	(83.1	(0.13	(683.5	(-0.72	(62.3	(-0.01	(309.3	(-0.04	(89.5	(0.39	(0.2	(0.06	(0.9	(-1.74	(118.2	(0.06	(276.5	(-0.16	(245.4	(-0.24	(21	(0.84
	,2999.3)	,-0.21)	,181.2)	,0.17)	,1683.7)	,-0.52)	,172.1)	,0.01)	,934.7)	,0.12)	,186.5)	,0.41)	,1.5)	,0.14)	,13.8)	,-1.4)	,274.2)	,0.07)	,595)	,0.09)	,555.8)	,0.02)	,55.8)	,1.14)
	2477.4	-0.16	140.6	0	1313.2	-0.35	167.7	-0.02	566.8	0.13	92.2	0.07	2.6	-0.09	5.6	0.28	188.7	-0.05	226.9	0.86	205.7	1.01	21.2	-0.4
Cuba	(1783	(-0.23	(89.3	(-0.03	(844.7	(-0.45	(97.7	(-0.03	(323.8	(0.05	(61.5	(0.06	(1.2	(-0.11	(0.5	(-0.12	(120.9	(-0.06	(149.1	(0.74	(132.4	(0.87	(11.4	(-0.45
	,3390.2)	,-0.1)	,199.2)	,0.04)	,2010.6)	,-0.25)	,261.7)	,-0.02)	,961.2)	,0.2)	,132.5)	,0.09)	,4.7)	,-0.08)	,13.3)	,0.69)	,283.8)	,-0.04)	,322.6)	,0.99)	,297.6)	,1.16)	,34.5)	,-0.35)
	2116.6	0.02	176.2	0.01	684.1	-0.1	212.4	0.07	727.1	0.07	115.4	0.45	0.7	0.14	8.9	-1.31	191.8	0.05	142	-0.16	112.7	-0.24	29.4	0.18
Cyprus	(1500.8	(-0.01	(114.3	(0	(423.3	(-0.17	(124.5	(0.06	(406.6	(0.02	(77.3	(0.42	(0.2	(0.11	(2.5	(-1.44	(122.5	(0.05	(89.7	(-0.26	(66.7	(-0.37	(16	(0.14
	,2849.3)	,0.06)	,245.2)	,0.01)	,1041)	,-0.02)	,328.4)	,0.08)	,1193.1)	,0.12)	,162.2)	,0.48)	,1.4)	,0.17)	,17.8)	,-1.17)	,282.2)	,0.06)	,209.8)	,-0.06)	,175.5)	,-0.12)	,46.1)	,0.22)
	2048.3	-0.32	131	0.11	983.3	-0.7	108.7	-0.02	503.7	-0.03	131.6	0.38	0.7	0.1	4.8	-1.48	184.4	0.06	342.1	0.87	311.8	0.83	30.4	1.29
Czech Republic	(1440.3	(-0.4	(83	(0.11	(616.8	(-0.83	(62.2	(-0.02	(279.2	(-0.1	(88.4	(0.35	(0.2	(0.08	(0.6	(-1.58	(117.6	(0.05	(219.4	(0.74	(195.1	(0.69	(16.3	(1.2
	,2792.1)	,-0.25)	,184.6)	,0.12)	,1468.5)	,-0.56)	,170.8)	,-0.01)	,822.3)	,0.05)	,185.4)	,0.4)	,1.5)	,0.13)	,11.7)	,-1.37)	,275.9)	,0.07)	,497.7)	,1)	,462.9)	,0.98)	,47.4)	,1.39)
	2337.3	0.15	121.3	0.12	1472.7	0.16	113.2	0.05	329.4	0.16	106.5	0.22	0.3	0.05	3.5	1.04	190.4	0.03	105.9	0.01	85.6	0.02	20.2	-0.03
C 湾坱 e d'Ivoire	(1617.6	(0.13	(75.7	(0.1	(915.3	(0.14	(63.8	(0.04	(184.4	(0.13	(71.1	(0.19	(0.1	(0.01	(0.4	(0.84	(122.3	(0.02	(66	(-0.04	(51	(-0.04	(10.2	(-0.07
	,3206.7)	,0.16)	,172)	,0.14)	,2201.4)	,0.19)	,178.5)	,0.06)	,550.7)	,0.18)	,152.8)	,0.26)	,0.6)	,0.09)	,10)	,1.24)	,284.9)	,0.04)	,155.7)	,0.06)	,132.4)	,0.08)	,33.8)	,0.01)
Democratic People's Republic of	1889.5	-0.12	143	-0.33	865.6	-0.25	48.2	0.01	544.4	-0.04	87.3	0.37	1	0.13	14.1	1.33	185.9	0.06	90.7	-0.1	52.7	0.3	37.9	-0.6
Korea	(1356.2	(-0.14	(90.8	(-0.35	(564.6	(-0.27	(25.6	(-0.01	(313.7	(-0.06	(57.9	(0.34	(0.3	(0.1	(4.4	(1.11	(117.1	(0.05	(58.2	(-0.12	(30.8	(0.28	(21	(-0.64
	,2539.9)	,-0.11)	,204.3)	,-0.32)	,1277.2)	,-0.23)	,80.8)	,0.02)	,885)	,-0.02)	,123.7)	,0.4)	,2.1)	,0.16)	,26.7)	,1.54)	,279.6)	,0.07)	,130.3)	,-0.08)	,82.7)	,0.33)	,59.1)	,-0.56)
	2643.1	0.09	106	-0.06	1713.4	0.08	119.2	0.04	417.7	0.18	95.8	0.15	0.3	0.03	7.6	2.79	183.1	0	156	-0.02	133.6	-0.1	22.4	0.44
Democratic Republic of the Congo	(1829.3	(0.06	(67.2	(-0.13	(1062.8	(0.05	(68.8	(0.03	(234.2	(0.14	(64.7	(0.08	(0.1	(0	(1.1	(2.17	(115.8	(-0.02	(97.1	(-0.18	(78.9	(-0.27	(11.8	(0.36
	,3589.9)	,0.11)	,150.4)	,0.01)	,2561.9)	,0.1)	,189.1)	,0.05)	,689.8)	,0.21)	,137.3)	,0.22)	,0.6)	,0.07)	,18.9)	,3.41)	,274.5)	,0.02)	,228.7)	,0.13)	,201.4)	,0.07)	,35.9)	,0.53)
	2214.7	-0.19	220.2	1.04	841.1	-0.83	214.8	0.13	622.3	0.06	121.9	0.5	0.7	-0.03	1.4	-2.15	192.4	0.08	376.6	1.37	325.1	1.39	51.5	1.25
Denmark	(1576.3	(-0.27	(153.9	(0.85	(524.6	(-1	(125.1	(0.11	(354.8	(0	(81.7	(0.49	(0.2	(-0.06	(0.2	(-2.25	(121.3	(0.07	(246.2	(1.16	(203.8	(1.13	(31.4	(1.17
	,2916.6)	,-0.1)	,288.8)	,1.23)	,1265.5)	,-0.66)	,332.9)	,0.15)	,988)	,0.11)	,170.1)	,0.52)	,1.4)	,0.01)	,3.5)	,-2.04)	,289.6)	,0.09)	,552.6)	,1.58)	,489)	,1.64)	,75.3)	,1.33)
Djibouti	2623.8	0.04	118.8	0.01	1685.4	0.03	130	0	382.9	0.04	110	0.29	0.3	0.08	3.3	0.6	193.1	0.05	239.4	0.23	214.3	0.18	25.1	0.67
	(1815.2	(0.02	(76.2	(-0.02	(1072.1	(0	(74.8	(0	(214.6	(-0.01	(73.7	(0.25	(0.1	(0.05	(0.4	(0.42	(122.9	(0.05	(152.2	(0.16	(131.2	(0.1	(13.1	(0.63

	,3640.9)	,0.07)	,166.5)	,0.04)	,2514.6)	,0.06)	,207.3)	,0.01)	,639)	,0.09)	,158.6)	,0.33)	,0.6)	,0.11)	,9.7)	,0.78)	,290)	,0.06)	,353)	,0.3)	,323.4)	,0.26)	,40.3)	,0.71)
	1999	-0.01	139	0.13	860.1	-0.05	164.3	-0.06	556.1	-0.06	83.2	0.36	2.7	0.62	5.2	-0.4	188.3	0.09	263.9	0.46	240	0.49	23.9	0.12
Dominica	(1408.2	(-0.06	(89.9	(0.11	(522.5	(-0.14	(96.6	(-0.06	(317.9	(-0.12	(55.7	(0.3	(1.2	(0.51	(0.6	(-0.46	(120	(0.06	(165.7	(0.3	(144.5	(0.32	(13.1	(0.06
	,2710.7)	,0.04)	,196.8)	,0.15)	,1343.8)	,0.04)	,253.9)	,-0.05)	,906.5)	,-0.01)	,119.2)	,0.41)	,4.9)	,0.73)	,12.3)	,-0.33)	,280.7)	,0.12)	,392.7)	,0.62)	,366.8)	,0.67)	,37.1)	,0.19)
	2372.1	0.16	142.9	0.15	1229.9	0.26	164.4	-0.04	558.1	0.1	80.8	0.09	2.6	-0.03	4.3	-1.55	188.9	-0.05	156.7	-0.11	136.3	-0.17	20.3	0.32
Dominican Republic	(1632.4	(0.07	(92.1	(0.13	(748.5	(0.09	(95.5	(-0.05	(316.6	(0.03	(54.8	(0.06	(1.1	(-0.05	(0.4	(-1.61	(121.6	(-0.05	(97.9	(-0.22	(80.9	(-0.29	(11.3	(0.28
	,3282.5)	,0.24)	,201.5)	,0.16)	,1833.1)	,0.42)	,254.8)	,-0.04)	,931.9)	,0.17)	,114.2)	,0.12)	,4.8)	,-0.01)	,10.5)	,-1.49)	,281.2)	,-0.04)	,233.3)	,-0.01)	,209.2)	,-0.05)	,32.2)	,0.37)
	2273.2	0.23	142.6	0.07	1021.6	0.38	165.4	-0.03	663.1	0.19	83.5	0.22	1.8	-0.04	7.3	-0.67	188	-0.04	159.4	-1.23	137.9	-1.4	21.5	0.21
Ecuador	(1594.9	(0.15	(93.2	(0.05	(622	(0.24	(97.5	(-0.04	(389.6	(0.09	(55.9	(0.19	(0.8	(-0.06	(1.8	(-0.76	(118.4	(-0.05	(99.5	(-1.39	(82.2	(-1.58	(11.8	(0.18
	,2995.3)	,0.3)	,201)	,0.08)	,1520.1)	,0.53)	,256.4)	,-0.03)	,1092.4)	,0.29)	,118.4)	,0.24)	,3.5)	,-0.02)	,14.7)	,-0.58)	,285.7)	,-0.03)	,239.8)	,-1.06)	,215.2)	,-1.21)	,33.8)	,0.24)
	2014.2	0.15	127.6	0.16	980.6	0.26	139.6	-0.04	438.3	0.1	96.5	0.32	1.2	0.05	38.6	-0.99	191.7	0.02	68.1	0.5	30	-0.06	38.1	1.01
Egypt	(1434.7	(0.08	(81.4	(0.14	(613.1	(0.15	(79.6	(-0.05	(240.3	(-0.01	(63.9	(0.3	(0.4	(0.02	(17.8	(-1.12	(123.5	(0.01	(43.2	(0.46	(15.7	(-0.13	(21.4	(0.97
	,2703.1)	,0.21)	,177.9)	,0.18)	,1474.7)	,0.37)	,219.1)	,-0.03)	,742.3)	,0.21)	,138.3)	,0.35)	,2.3)	,0.08)	,64.7)	,-0.86)	,286.7)	,0.03)	,98.7)	,0.54)	,49.5)	,0.02)	,58.8)	,1.05)
	2109.3	0	137.9	0.09	1002.7	-0.14	149.7	0	537.9	0.26	89.9	0.23	1.1	-0.11	5.5	-1.05	184.5	-0.05	398.8	-0.92	379.2	-0.96	19.6	0.08
El Salvador	(1466.3	(-0.05	(88.1	(0.08	(609.4	(-0.22	(86.7	(-0.01	(302.8	(0.19	(60.4	(0.19	(0.4	(-0.14	(0.6	(-1.43	(119	(-0.05	(245.9	(-1.09	(229.9	(-1.14	(10.5	(0.04
	,2876.2)	,0.05)	,200.1)	,0.11)	,1553.7)	,-0.06)	,237.4)	,0)	,893.4)	,0.33)	,129)	,0.27)	,2.3)	,-0.09)	,12.5)	,-0.68)	,277.7)	,-0.04)	,604.6)	,-0.75)	,583.8)	,-0.79)	,32.3)	,0.11)
	2766.8	0.15	131.1	0.92	1811.3	0.1	119.3	0.04	418.8	0.13	102.8	0.65	0.3	-0.05	0.8	-7.79	182.4	0.03	162.4	0.1	135.8	-0.03	26.6	0.84
Equatorial Guinea	(1917.4	(0.12	(83.9	(0.77	(1126.6	(0.05	(67.6	(0.03	(239	(0.09	(69.8	(0.6	(0.1	(-0.08	(0.1	(-9.08	(117	(0.02	(103.9	(0.03	(82.9	(-0.11	(14.5	(0.8
	,3902.7)	,0.17)	,184.1)	,1.07)	,2815.6)	,0.14)	,190.4)	,0.05)	,718.5)	,0.17)	,145.6)	,0.7)	,0.6)	,-0.03)	,3)	,-6.48)	,274.4)	,0.04)	,238.4)	,0.17)	,205.9)	,0.05)	,43.2)	,0.89)
	2832.5	-0.04	110.7	0.1	1879.2	-0.1	128.2	0.01	436.6	0.12	91.3	0.15	0.3	-0.13	5	-0.15	181.3	0	218.2	0.08	195.9	0.07	22.2	0.17
Eritrea	(1876.5	(-0.06	(69.3	(0.06	(1121.6	(-0.13	(73.9	(0	(240.9	(0.08	(61.6	(0.14	(0.1	(-0.17	(0.6	(-0.71	(114.9	(-0.01	(139.8	(0.03	(121.2	(0.02	(11.1	(0.12
	,3920.9)	,-0.02)	,159.1)	,0.14)	,2846.4)	,-0.07)	,200.3)	,0.01)	,719.8)	,0.16)	,130.2)	,0.16)	,0.6)	,-0.09)	,13.4)	,0.42)	,273.6)	,0)	,322)	,0.13)	,295)	,0.12)	,36.5)	,0.22)
	2554	-0.43	122.2	0.27	1480.3	-0.78	111.7	0.01	530.1	0.01	121.7	0.47	0.7	0.1	4.5	-2.45	182.7	0.07	339.4	0.37	275.2	0.4	64.2	0.2
Estonia	(1770.9	(-0.52	(77.3	(0.25	(930.9	(-0.92	(64.3	(0	(292.6	(-0.06	(82.2	(0.45	(0.2	(0.07	(0.5	(-2.65	(115.4	(0.06	(227.5	(0.14	(176.8	(0.13	(39.5	(0.14
	,3502.3)	,-0.35)	,174.6)	,0.29)	,2257.3)	,-0.65)	,176.8)	,0.02)	,866.6)	,0.09)	,171.4)	,0.49)	,1.5)	,0.13)	,11.3)	,-2.24)	,274.2)	,0.07)	,479.4)	,0.59)	,409.9)	,0.67)	,93.8)	,0.26)
	2560.3	0.17	119.4	0.05	1578.1	0.26	117.9	-0.07	469.6	0.14	93.3	-0.11	0.3	-0.18	3.6	0.37	178.2	-0.12	226	-0.22	177.9	-0.37	48.1	0.4
Eswatini	(1769.7	(0.11	(74.2	(0.04	(948.1	(0.17	(67.6	(-0.08	(263.5	(0.05	(62.4	(-0.15	(0.1	(-0.22	(0.5	(-0.12	(113.1	(-0.13	(144.8	(-0.32	(105.4	(-0.49	(28.9	(0.34
	,3530.4)	,0.23)	,170.8)	,0.07)	,2397.3)	,0.35)	,185.9)	,-0.06)	,795.6)	,0.24)	,132)	,-0.07)	,0.6)	,-0.14)	,9.4)	,0.86)	,264.7)	,-0.11)	,325.5)	,-0.12)	,272.9)	,-0.25)	,72.1)	,0.45)
	2759	-0.03	112.6	0.31	1795.5	-0.11	135.1	0.06	413.2	0.1	102.8	0.39	0.2	-0.05	4.4	-0.05	195.1	0.03	362.8	-0.28	339.4	-0.3	23.5	-0.01
Ethiopia	(1952.3	(-0.11	(79.2	(0.26	(1160.8	(-0.24	(84	(0.05	(269.3	(0.02	(70.2	(0.37	(0.1	(-0.08	(0.6	(-0.4	(126.2	(0.01	(234	(-0.36	(215.2	(-0.39	(14.8	(-0.02
	,3654.2)	,0.05)	,150)	,0.35)	,2563.6)	,0.02)	,199.8)	,0.06)	,608.7)	,0.19)	,142.5)	,0.4)	,0.5)	,-0.02)	,12.1)	,0.29)	,289.4)	,0.05)	,518.5)	,-0.21)	,492.7)	,-0.22)	,34.4)	,0.01)
	1753.9	0.05	154.5	-0.04	701.7	0.09	69.4	-0.04	555.4	0.11	81	-0.04	0.8	-0.06	7.6	-0.1	183.5	-0.06	72.1	-0.41	48	-0.48	24.2	-0.27
Fiji	(1263.6	(0.03	(101.5	(-0.05	(467.2	(0.06	(38.8	(-0.05	(305.1	(0.06	(54.3	(-0.06	(0.2	(-0.09	(2	(-0.19	(118	(-0.07	(45.1	(-0.47	(26.4	(-0.54	(12.3	(-0.33
	,2335.4)	,0.08)	,217.2)	,-0.02)	,1029.8)	,0.12)	,112.2)	,-0.03)	,927.4)	,0.15)	,117.6)	,-0.02)	,1.5)	,-0.02)	,15.4)	,-0.01)	,273.2)	,-0.05)	,108.3)	,-0.36)	,78.1)	,-0.43)	,39.9)	,-0.21)
	2051.1	0.16	189.9	-0.16	877.3	0.01	211.8	0.08	471.8	0.68	102	0.44	0.9	0.18	9.2	-1.36	188.3	0.1	240.3	1.28	165.8	1.17	74.6	1.52
Finland	(1477.1	(0.06	(125.1	(-0.19	(552.2	(-0.13	(123.4	(0.06	(261.9	(0.39	(67.9	(0.41	(0.3	(0.14	(2.8	(-1.52	(121.6	(0.09	(166.3	(1.14	(109	(1.04	(48.3	(1.29
	,2736.8)	,0.26)	,265.4)	,-0.13)	,1338.1)	,0.15)	,323.8)	,0.09)	,768.5)	,0.97)	,144.7)	,0.46)	,1.8)	,0.22)	,17.7)	,-1.2)	,282)	,0.11)	,334)	,1.41)	,245.5)	,1.29)	,104.6)	,1.75)
	2383.4	-0.06	173.9	-0.02	890.4	-0.13	211.7	0.06	796	-0.08	112.2	0.36	0.4	0.07	9.9	-1.24	189	0.05	249.6	0.67	171.5	0.02	78.1	2.62
France	(1725	(-0.14	(113.2	(-0.04	(563.6	(-0.39	(125.1	(0.05	(468.7	(-0.23	(75.5	(0.33	(0.1	(0.02	(3.2	(-1.27	(119.6	(0.05	(172.3	(0.61	(110.9	(-0.06	(50.6	(2.46
	,3212.9)	,0.02)	,241.8)	,-0.01)	,1336.5)	,0.13)	,325.9)	,0.07)	,1285.1)	,0.07)	,158.4)	,0.38)	,1)	,0.13)	,19.3)	,-1.2)	,281.4)	,0.06)	,341.1)	,0.73)	,249.3)	,0.09)	,111.1)	,2.77)

	2790.2	-0.01	127.2	0.01	1818.7	-0.06	119.5	-0.02	431.3	0.05	106.8	0.41	0.3	0.19	1.1	0.8	185.6	0.04	194.1	0.15	165.8	0.11	28.3	0.38
Gabon	(1922.8	(-0.04	(83.1	(0	(1117.6	(-0.1	(68.9	(-0.02	(247.6	(0	(71.7	(0.36	(0.1	(0.13	(0.1	(0.7	(119.1	(0.02	(124.3	(0.05	(100.3	(0	(15.8	(0.32
	,3931.3)	,0.01)	,177.8)	,0.02)	,2848.4)	,-0.03)	,189.4)	,-0.01)	,731.8)	,0.1)	,152.9)	,0.45)	,0.6)	,0.24)	,4)	,0.89)	,278.3)	,0.05)	,285.5)	,0.25)	,252)	,0.22)	,45.5)	,0.44)
	3231.6	0.07	117.8	0.03	2361.5	0.08	113.4	-0.01	342.5	0.11	104.4	0.04	0.3	-0.13	3.8	1.85	187.8	-0.05	103.7	0.14	83	0.23	20.7	-0.18
Gambia	(2151	(0.05	(73.6	(0.02	(1449.1	(0.05	(65.2	(-0.01	(194.2	(0.06	(69.1	(0.03	(0.1	(-0.16	(0.5	(1.68	(120.9	(-0.06	(66.1	(0.09	(50.2	(0.16	(10.3	(-0.2
	,4410.7)	,0.1)	,167.2)	,0.05)	,3437.4)	,0.12)	,180.2)	,0)	,564.1)	,0.15)	,147.7)	,0.06)	,0.6)	,-0.09)	,11.5)	,2.02)	,279.1)	,-0.05)	,153.1)	,0.19)	,128)	,0.3)	,33.9)	,-0.16)
	2299.3	0.01	120.6	0.09	1414.7	0.02	110.8	-0.04	349.6	0	109.6	0.06	0.7	0.02	9.8	-0.94	183.5	-0.01	224.2	-1.06	187.9	-1.16	36.3	-0.48
Georgia	(1562.9	(-0.03	(76.5	(0.02	(864.9	(-0.03	(63	(-0.05	(189.5	(-0.08	(74.6	(0.03	(0.2	(-0.02	(2.3	(-1.63	(116.3	(-0.02	(141.2	(-1.16	(112.6	(-1.28	(19.3	(-0.55
	,3147.1)	,0.06)	,170.5)	,0.16)	,2120.9)	,0.08)	,174.7)	,-0.04)	,583.5)	,0.08)	,155.6)	,0.09)	,1.5)	,0.06)	,20)	,-0.26)	,276.1)	,0)	,328.7)	,-0.96)	,286)	,-1.05)	,59.3)	,-0.41)
	2331.6	0.31	160.1	-0.09	818.6	0.38	190.6	0.07	854.7	0.45	113.5	0.43	0.3	0.13	4.6	-1.96	189.1	0.09	212.7	0.7	178	0.72	34.7	0.6
Germany	(1654.3	(0.18	(105.4	(-0.11	(519.6	(0.24	(112.8	(0.05	(488.4	(0.17	(76.4	(0.37	(0.1	(0.09	(1	(-2.05	(118.6	(0.07	(142.7	(0.63	(113.6	(0.62	(20.1	(0.53
	,3120.2)	,0.44)	,223.5)	,-0.06)	,1226.7)	,0.52)	,293.4)	,0.09)	,1362.5)	,0.73)	,160.2)	,0.48)	,0.6)	,0.17)	,10.4)	,-1.87)	,282.7)	,0.1)	,302.7)	,0.77)	,264.7)	,0.81)	,52.9)	,0.67)
	2593.2	0.15	125.8	0.23	1731.1	0.18	114.3	0.01	331	0.15	102.4	0.08	0.3	-0.09	2.3	0.52	186	-0.04	107.1	0.1	80.8	0.02	26.3	0.34
Ghana	(1743.3	(0.12	(80.2	(0.2	(1060.2	(0.15	(65	(0.01	(179.3	(0.11	(68.1	(0.06	(0.1	(-0.13	(0.3	(0.28	(118.4	(-0.04	(68.1	(0.05	(48.3	(-0.05	(14.1	(0.29
	,3602.6)	,0.18)	,178.2)	,0.25)	,2602.2)	,0.21)	,181.7)	,0.02)	,546.8)	,0.2)	,145.2)	,0.09)	,0.6)	,-0.06)	,7.3)	,0.76)	,275.9)	,-0.03)	,154.8)	,0.15)	,124.2)	,0.09)	,42.7)	,0.4)
	2747.1	-0.13	174.3	-0.01	1329.1	-0.35	212.9	0.11	718.3	0.13	111.8	0.12	0.7	0.02	9.8	-1.11	190.2	0.02	111.5	-0.19	85.6	-0.37	25.9	0.46
Greece	(1892.7	(-0.27	(113.2	(-0.02	(809.4	(-0.62	(124.5	(0.09	(415.8	(0.03	(76	(0.11	(0.2	(-0.02	(2.8	(-1.33	(121.2	(0.02	(68	(-0.31	(48.7	(-0.52	(13.3	(0.33
	,3733.3)	,0)	,241.3)	,0)	,2061.8)	,-0.08)	,332.8)	,0.12)	,1167.7)	,0.23)	,158.2)	,0.14)	,1.4)	,0.06)	,19.1)	,-0.88)	,285.3)	,0.03)	,170.9)	,-0.06)	,139)	,-0.22)	,42.2)	,0.59)
	1973.9	-0.03	195	0.14	760.6	-0.2	137.5	0.04	537.3	-0.07	129.9	0.54	1.2	0.27	7.3	-1.25	205	0.2	322.3	-0.39	265.6	-0.39	56.7	-0.39
Greenland	(1411.4	(-0.07	(129.7	(0.09	(468.2	(-0.26	(81.6	(0.03	(302.5	(-0.16	(86.9	(0.51	(0.4	(0.22	(1.7	(-1.43	(131.8	(0.18	(205.9	(-0.48	(160.5	(-0.48	(34.8	(-0.5
	,2652.4)	,0.01)	,272.1)	,0.19)	,1172.8)	,-0.14)	,213.6)	,0.05)	,880.5)	,0.02)	,185.3)	,0.57)	,2.4)	,0.32)	,15)	,-1.07)	,300.7)	,0.21)	,463.5)	,-0.29)	,399.1)	,-0.29)	,82.2)	,-0.29)
	2041.7	0.03	139.9	0.16	901.2	-0.01	162.9	-0.05	561.3	0.07	83.2	0.22	2.7	0.24	4.5	-1.12	186	-0.01	239.5	0.37	215.9	0.39	23.7	0.12
Grenada	(1439.6	(-0.02	(89.4	(0.15	(551.2	(-0.08	(94.6	(-0.05	(313	(0.01	(54.7	(0.19	(1.2	(0.22	(0.5	(-1.21	(117.7	(-0.03	(153.6	(0.19	(135.2	(0.2	(13.6	(0.09
	,2784.3)	,0.07)	,198.2)	,0.17)	,1384.5)	,0.06)	,252.7)	,-0.04)	,940.8)	,0.13)	,117.7)	,0.25)	,4.9)	,0.27)	,10.6)	,-1.04)	,277.3)	,0.01)	,356.7)	,0.54)	,328.8)	,0.59)	,37.1)	,0.14)
	1883.6	0.07	185.1	-0.1	769.2	0.1	72.3	-0.02	568.6	0.16	95.1	-0.01	0.8	-0.05	2.5	-0.13	189.9	-0.04	96.6	-0.47	67.9	-0.57	28.7	-0.25
Guam	(1354.3	(0.04	(122.7	(-0.11	(495.3	(0.06	(40.1	(-0.03	(313.3	(0.11	(63.3	(-0.05	(0.2	(-0.09	(0.3	(-0.21	(120.8	(-0.04	(60.2	(-0.52	(39.5	(-0.63	(14.5	(-0.28
	,2540.8)	,0.1)	,258.6)	,-0.09)	,1143.2)	,0.14)	,118.3)	,-0.02)	,926.8)	,0.22)	,134.4)	,0.02)	,1.7)	,0)	,6.6)	,-0.06)	,285.3)	,-0.03)	,141.1)	,-0.42)	,104.9)	,-0.5)	,46.4)	,-0.22)
	2321.1	-0.06	135.1	0.05	1250	-0.19	146.1	-0.03	512.4	0.21	85.2	0.2	1.2	-0.12	5.3	-1.07	185.9	-0.05	389.4	-1.04	368.8	-1.07	20.5	-0.37
Guatemala	(1614.6	(-0.23	(86	(0.04	(782.8	(-0.5	(84.7	(-0.04	(282	(0.13	(57.1	(0.17	(0.4	(-0.15	(0.6	(-1.47	(118.3	(-0.06	(247.9	(-1.19	(231.5	(-1.24	(11.4	(-0.58
	,3192.2)	,0.11)	,190)	,0.06)	,1900.3)	,0.12)	,227.7)	,-0.02)	,836.5)	,0.28)	,121.9)	,0.23)	,2.3)	,-0.09)	,12.2)	,-0.68)	,274.8)	,-0.04)	,591.8)	,-0.88)	,568.4)	,-0.9)	,32.9)	,-0.16)
	2526.2	0.15	117.6	0.03	1656.9	0.21	113.6	0.01	340.9	0.03	102.8	0.23	0.3	0.13	2.5	0.88	191.6	0.04	100.3	0.2	81.7	0.27	18.5	-0.06
Guinea	(1748.5	(0.13	(73.7	(0.01	(1032.8	(0.17	(65.5	(0	(194	(-0.01	(69.1	(0.2	(0.1	(0.09	(0.5	(0.62	(123.6	(0.02	(63	(0.13	(49.6	(0.18	(9.1	(-0.08
	,3475.3)	,0.18)	,168.3)	,0.06)	,2501.3)	,0.24)	,182.2)	,0.01)	,580)	,0.08)	,147)	,0.26)	,0.6)	,0.17)	,8.6)	,1.13)	,284.1)	,0.05)	,148.8)	,0.27)	,126.9)	,0.36)	,31)	,-0.04)
	2555.8	0.22	115.1	0.07	1710.1	0.29	112.2	0.01	333.7	0.15	94.5	0.11	0.3	-0.02	3.4	1.96	186.5	-0.03	106.1	0.07	87	0.11	19.1	-0.1
Guinea-Bissau	(1771.3	(0.2	(70.2	(0.06	(1056.6	(0.26	(63.8	(0.01	(183.6	(0.1	(63.8	(0.07	(0.1	(-0.04	(0.6	(1.67	(118.5	(-0.04	(67	(0.01	(51.9	(0.04	(9.4	(-0.12
	,3566.1)	,0.24)	,165.9)	,0.08)	,2582.3)	,0.32)	,178.1)	,0.02)	,562)	,0.19)	,134.9)	,0.14)	,0.6)	,0.01)	,10.3)	,2.26)	,280.7)	,-0.02)	,157)	,0.14)	,132.3)	,0.19)	,33.4)	,-0.08)
	2471.5	0.19	131.1	0.14	1343.7	0.29	158.8	-0.01	571.6	0.13	73.9	0.07	2.6	-0.04	5.5	-1.12	184.3	-0.02	208.4	-0.33	189.3	-0.34	19.1	-0.24
Guyana	(1719.2	(0.14	(82.7	(0.12	(823	(0.24	(91.4	(-0.02	(315.7	(0.04	(48.8	(0.06	(1.1	(-0.06	(0.7	(-1.23	(118.8	(-0.03	(130	(-0.43	(114.5	(-0.45	(10.1	(-0.27
	,3378.7)	,0.24)	,186)	,0.15)	,2068.2)	,0.35)	,246.7)	,0)	,947.3)	,0.21)	,105)	,0.09)	,4.7)	,-0.03)	,12.4)	,-1.02)	,270.5)	,-0.02)	,306.9)	,-0.23)	,284.9)	,-0.23)	,31)	,-0.21)
Haiti	2085.4	0.05	117.5	-0.1	978.5	0.04	159.4	-0.02	561.7	0.14	67.5	0.12	2.5	-0.07	11.4	0.44	186.7	-0.01	234.1	-0.26	213.6	-0.28	20.5	-0.01
	(1463.8	(0.01	(75.8	(-0.11	(612.3	(-0.03	(91.8	(-0.02	(318.7	(0.07	(43.8	(0.11	(1.1	(-0.1	(2.9	(0.31	(118.8	(-0.02	(145.3	(-0.37	(128	(-0.4	(11.8	(-0.05

	,2833.5)	,0.1)	,169.6)	,-0.08)	,1479.6)	,0.1)	,251.7)	,-0.01)	,918.8)	,0.21)	,96.7)	,0.14)	,4.8)	,-0.05)	,23.1)	,0.56)	,280.1)	,0)	,351.9)	,-0.15)	,328.4)	,-0.16)	,33)	,0.03)
	2258.3	0.23	132.1	-0.01	1179.8	0.37	147.2	-0.04	519.1	0.21	84.8	0.11	1.2	-0.06	7.3	-0.51	186.8	-0.04	367.7	0.6	326	0.5	41.7	1.44
Honduras	(1530.3	(0.19	(84.1	(-0.02	(716.7	(0.31	(83.9	(-0.05	(282.9	(0.11	(57	(0.09	(0.4	(-0.09	(1	(-0.93	(119.2	(-0.05	(231	(0.43	(197.5	(0.31	(25.7	(1.37
	,3083.3)	,0.27)	,188.5)	,0)	,1808.1)	,0.43)	,229.6)	,-0.03)	,887.8)	,0.32)	,119.7)	,0.13)	,2.3)	,-0.03)	,15.7)	,-0.08)	,277.1)	,-0.03)	,541.5)	,0.77)	,494.2)	,0.7)	,62.5)	,1.52)
	2131.5	-0.18	129.8	0.21	1040.7	-0.47	107.6	0.04	540.4	0.11	123.7	0.32	0.7	0.07	5.4	-1.47	183.1	0.06	359.7	-0.67	336.6	-0.73	23.1	0.21
Hungary	(1497.3	(-0.28	(82.3	(0.19	(640.6	(-0.66	(62	(0.03	(313.3	(0.04	(84.2	(0.31	(0.2	(0.03	(0.8	(-1.57	(116.1	(0.05	(228.4	(-0.84	(210.4	(-0.9	(12.1	(0.15
	,2871.5)	,-0.08)	,183.2)	,0.23)	,1567.4)	,-0.28)	,169.5)	,0.05)	,892.8)	,0.17)	,174.3)	,0.34)	,1.6)	,0.11)	,12.6)	,-1.37)	,276.4)	,0.06)	,521.2)	,-0.51)	,494.8)	,-0.55)	,38.2)	,0.27)
	1986.2	-0.05	181.7	0	639.8	-0.19	207.7	-0.08	616.4	0.01	140.2	0.29	0.8	0.03	6.1	-1.46	193.5	0.05	249	0.65	159.5	0.24	89.5	1.54
Iceland	(1431	(-0.09	(117.9	(-0.01	(405.2	(-0.29	(121.7	(-0.1	(355.2	(-0.03	(95.4	(0.27	(0.2	(-0.01	(1.6	(-1.54	(122.7	(0.04	(170.6	(0.62	(97.9	(0.19	(58.5	(1.44
	,2656.5)	,-0.01)	,252.7)	,0.02)	,974.5)	,-0.1)	,328.1)	,-0.05)	,975.9)	,0.05)	,194.2)	,0.31)	,1.7)	,0.06)	,13.2)	,-1.38)	,287.2)	,0.05)	,341.5)	,0.69)	,234)	,0.29)	,125.3)	,1.63)
	2301.9	-0.14	169	0.39	1263.5	-0.28	89.1	0.02	443.2	-0.02	81.1	0.21	0.3	0.04	65	-0.63	190.7	0	203.2	-0.4	167.8	-0.45	35.4	-0.13
India	(1686.7	(-0.27	(121.1	(0.34	(850.3	(-0.45	(55.6	(0.02	(291.8	(-0.25	(55.6	(0.19	(0.1	(0.02	(32.9	(-0.7	(124.1	(-0.01	(132	(-0.55	(104.9	(-0.63	(22.6	(-0.2
	,2979.6)	,-0.02)	,219.1)	,0.44)	,1729.5)	,-0.11)	,131.1)	,0.03)	,640.8)	,0.21)	,112.4)	,0.24)	,0.6)	,0.07)	,104.8)	,-0.57)	,285.3)	,0)	,288)	,-0.26)	,247)	,-0.28)	,50.7)	,-0.06)
	1624.4	0.07	155.4	0.2	571.9	0.02	85.5	0.01	523.7	0.14	79.8	0.28	0.5	0.06	13	-1.45	194.7	0.01	57.3	0.18	44	0.21	13.2	0.09
Indonesia	(1189.9	(0.03	(111.8	(0.17	(388.2	(-0.04	(52.8	(0.01	(347.1	(0.08	(54.5	(0.25	(0.2	(0.04	(4.5	(-1.81	(124.9	(0.01	(37.2	(0.14	(27.3	(0.14	(8.2	(0.05
	,2107.6)	,0.11)	,200.4)	,0.24)	,805.8)	,0.08)	,128.1)	,0.02)	,767.9)	,0.21)	,112.5)	,0.3)	,1)	,0.08)	,23.6)	,-1.08)	,291.1)	,0.02)	,81.3)	,0.22)	,65)	,0.27)	,19.6)	,0.12)
	2407.9	0.25	132.7	0.14	1037.2	0.36	160	-0.01	758	0.35	102	0.21	2.9	-0.17	21.2	-1.96	193.8	-0.06	73.9	-0.47	28.9	-0.5	45	-0.46
Iran (Islamic Republic of)	(1712.5	(0.2	(95.3	(0.11	(666.8	(0.3	(101.1	(-0.01	(500.1	(0.26	(69.9	(0.18	(1.4	(-0.2	(7.9	(-2.13	(125.7	(-0.07	(50.7	(-0.68	(17.9	(-0.56	(29.6	(-0.75
	,3182.7)	,0.3)	,171.2)	,0.16)	,1496.4)	,0.42)	,233.7)	,0)	,1093.1)	,0.44)	,142.2)	,0.25)	,5.1)	,-0.14)	,38.8)	,-1.79)	,289.7)	,-0.04)	,99.6)	,-0.27)	,43.1)	,-0.44)	,63.5)	,-0.16)
	1895.4	0.06	126.9	0.12	810.2	0.1	133.9	-0.05	536.6	0.08	85.4	0.27	1.3	0.01	17.3	-1.63	183.8	-0.04	57.5	-0.02	29.3	-0.19	28.2	0.16
Iraq	(1352.2	(-0.02	(82.2	(0.09	(514.1	(0.02	(77	(-0.06	(291.2	(-0.09	(57.7	(0.24	(0.5	(-0.03	(5.6	(-1.95	(119.5	(-0.05	(35	(-0.06	(15.4	(-0.23	(13.9	(0.11
	,2525.6)	,0.13)	,180.1)	,0.16)	,1224.9)	,0.17)	,209.9)	,-0.04)	,884)	,0.26)	,121.6)	,0.29)	,2.5)	,0.04)	,33.2)	,-1.32)	,268.8)	,-0.03)	,84.9)	,0.02)	,47.5)	,-0.15)	,45.4)	,0.2)
	2396.7	0.29	213	0.2	866.2	0.63	172.5	0.09	804.5	0.04	142.5	0.47	1	0.04	5.1	-3.27	191.8	0.05	259.1	0.64	198.9	0.36	60.2	1.83
Ireland	(1699.2	(0.25	(139.9	(0.16	(543.5	(0.5	(102.1	(0.07	(463	(-0.03	(96.3	(0.44	(0.3	(0.01	(1.1	(-3.53	(121.2	(0.05	(165.4	(0.47	(117.2	(0.15	(37.6	(1.76
	,3247.6)	,0.33)	,297.9)	,0.23)	,1308.3)	,0.75)	,265.7)	,0.1)	,1337.4)	,0.12)	,201)	,0.51)	,2)	,0.07)	,11.8)	,-3)	,284.7)	,0.06)	,371.4)	,0.8)	,302.6)	,0.57)	,86.3)	,1.91)
	2309.5	0.02	176.2	-0.02	1106.3	-0.24	243.2	0.05	482.5	0.7	100.4	0.23	0.8	0.1	9.6	-1.16	190.4	0.02	77.4	-1.04	43.7	-0.89	33.6	-1.21
Israel	(1640.5	(-0.05	(116.1	(-0.04	(690.7	(-0.31	(145.5	(0.04	(274.7	(0.42	(67.8	(0.21	(0.2	(0.07	(3	(-1.2	(120.6	(0.01	(49.9	(-1.16	(23.6	(-0.96	(19.8	(-1.48
	,3079)	,0.1)	,244.7)	,0)	,1678.8)	,-0.18)	,379.7)	,0.06)	,803.6)	,0.99)	,141.6)	,0.24)	,1.5)	,0.14)	,18.6)	,-1.13)	,283.8)	,0.03)	,110.4)	,-0.92)	,69.9)	,-0.82)	,51.5)	,-0.93)
	2336	-0.04	168	0	932	-0.28	214.9	0.16	693.1	0.17	128.3	0.34	0.4	0.08	7.8	-0.92	191.6	0.07	129.6	-0.19	98.3	-0.25	31.3	0
Italy	(1699.1	(-0.09	(121.7	(-0.01	(605.5	(-0.36	(134.6	(0.13	(463.8	(0.01	(88.2	(0.31	(0.1	(0.06	(2.4	(-1.03	(123.9	(0.06	(85.6	(-0.33	(61.1	(-0.36	(20.7	(-0.25
	,3030.9)	,0.02)	,216.2)	,0.01)	,1325.6)	,-0.2)	,315.7)	,0.19)	,986.1)	,0.32)	,178.3)	,0.37)	,0.9)	,0.1)	,15.6)	,-0.8)	,284.5)	,0.07)	,180)	,-0.05)	,144.9)	,-0.14)	,43.6)	,0.25)
	1996.1	0.1	139.4	0	855.1	0.13	166.5	-0.03	549	0.14	87.4	0.11	2.6	0.05	6.2	-0.11	190	-0.01	126.3	-0.32	102.5	-0.37	23.8	-0.11
Jamaica	(1396.5	(0.04	(89.9	(-0.01	(534.9	(0.03	(96.6	(-0.03	(299.8	(0.06	(59	(0.09	(1.2	(0.02	(0.7	(-0.17	(121.1	(-0.02	(78.1	(-0.4	(59.4	(-0.46	(13.5	(-0.16
	,2717.7)	,0.16)	,198.6)	,0.01)	,1310.6)	,0.23)	,258.6)	,-0.02)	,913.5)	,0.22)	,124.9)	,0.14)	,4.9)	,0.07)	,14.1)	,-0.05)	,284.3)	,-0.01)	,191.9)	,-0.24)	,162.3)	,-0.27)	,37.3)	,-0.06)
	1475.7	0.15	160.6	0.18	471.6	0.24	128.4	0.01	301.8	-0.11	217.3	0.51	0.5	0.09	1.2	-3.94	194.4	0.06	68.1	-0.24	43.3	-0.21	24.8	-0.28
Japan	(1107.1	(0.12	(116.7	(0.07	(317.6	(0.15	(81.4	(0.01	(197.1	(-0.21	(149.5	(0.47	(0.2	(0.08	(0	(-4.21	(125.8	(0.06	(45.4	(-0.31	(26.7	(-0.28	(16	(-0.38
	,1841.9)	,0.18)	,206.3)	,0.28)	,654.7)	,0.33)	,190.4)	,0.02)	,439.6)	,-0.02)	,302.7)	,0.55)	,1)	,0.1)	,4.1)	,-3.68)	,290)	,0.07)	,94.5)	,-0.16)	,64.7)	,-0.14)	,35.3)	,-0.17)
	2035.4	-0.17	128.5	0.05	930.5	-0.39	147.4	-0.04	511	0.09	104.2	0.3	1.2	-0.07	24.3	-1.43	188.3	-0.03	55.4	-0.21	30.3	-0.35	25.2	-0.04
Jordan	(1444.1	(-0.21	(82.9	(0.03	(580.5	(-0.45	(86	(-0.05	(275.6	(0.03	(70.6	(0.28	(0.4	(-0.11	(8	(-1.64	(121.3	(-0.04	(33.3	(-0.28	(16	(-0.41	(12.3	(-0.13
	,2765.2)	,-0.13)	,182.2)	,0.07)	,1429.7)	,-0.34)	,226.8)	,-0.03)	,845.1)	,0.14)	,148)	,0.32)	,2.3)	,-0.04)	,44.3)	,-1.22)	,279.7)	,-0.02)	,82.9)	,-0.14)	,50.3)	,-0.29)	,41.7)	,0.05)

	2040.3	-0.28	125.4	0.25	1208.1	-0.52	109.4	0	301.5	0	107.6	0.39	0.7	0.05	5.5	-0.89	182	0.03	491.1	0.18	404.8	-0.04	86.3	1.58
Kazakhstan	(1457.1	(-0.33	(79.5	(0.2	(770.1	(-0.6	(62.4	(-0.01	(172.8	(-0.06	(72.2	(0.34	(0.2	(0.02	(0.9	(-1.27	(115.8	(0.02	(332.2	(-0.19	(259.2	(-0.48	(55	(1.51
	,2746.8)	,-0.23)	,175.1)	,0.31)	,1780.9)	,-0.44)	,173.6)	,0)	,518.4)	,0.05)	,153.8)	,0.43)	,1.4)	,0.08)	,12.4)	,-0.5)	,272.5)	,0.04)	,710.5)	,0.56)	,609.2)	,0.4)	,120.9)	,1.66)
	2856.5	-0.11	114.9	0.1	1899.3	-0.22	133.1	0.02	408.5	0.23	102.3	0.03	0.2	0.02	6.3	-0.05	191.8	-0.01	124.1	0.16	101.9	0.12	22.2	0.33
Kenya	(2037.5	(-0.17	(82.3	(0.08	(1278.5	(-0.31	(83.7	(0.01	(270.3	(0.19	(70.4	(-0.01	(0.1	(0	(1.4	(-0.29	(123.5	(-0.03	(83.5	(0.03	(65.5	(-0.03	(14.6	(0.32
	,3723.3)	,-0.06)	,148.7)	,0.13)	,2622.9)	,-0.14)	,194.9)	,0.03)	,587.1)	,0.28)	,142.2)	,0.07)	,0.5)	,0.03)	,14.2)	,0.19)	,287.6)	,0)	,175.3)	,0.28)	,149.7)	,0.27)	,31.8)	,0.35)
	1720	-0.01	134.5	-0.14	684.2	-0.12	69.4	-0.01	565.8	0.13	70.9	0.02	0.8	-0.05	13.4	1.06	181.2	-0.03	79.8	-0.23	50.7	-0.42	29.1	0.12
Kiribati	(1243.9	(-0.04	(85	(-0.15	(445.4	(-0.16	(37.8	(-0.02	(322.9	(0.1	(46.5	(0	(0.3	(-0.08	(4.1	(0.95	(115.7	(-0.04	(50.7	(-0.26	(28.6	(-0.47	(15.9	(0.08
	,2290.6)	,0.01)	,190.6)	,-0.12)	,1005)	,-0.08)	,112.3)	,0)	,911.7)	,0.17)	,101.6)	,0.03)	,1.6)	,-0.02)	,26)	,1.18)	,268.4)	,-0.03)	,117.7)	,-0.2)	,81.5)	,-0.38)	,46.6)	,0.15)
	1954.8	0.09	146.5	-0.02	885.6	0.18	149.8	-0.05	449.8	0.1	116.5	0.11	1.2	-0.18	12.8	-0.89	192.7	-0.08	69.7	0.43	33.8	-0.23	35.8	1.18
Kuwait	(1386.8	(0.07	(96.2	(-0.04	(558.4	(0.15	(86.4	(-0.05	(252.2	(0.04	(77.5	(0.08	(0.5	(-0.22	(2.9	(-1.08	(122.8	(-0.1	(43.3	(0.38	(17.9	(-0.34	(20.1	(0.97
	,2621.5)	,0.12)	,204.9)	,0.01)	,1337.9)	,0.22)	,233.1)	,-0.04)	,748.3)	,0.16)	,164.4)	,0.14)	,2.5)	,-0.13)	,24.8)	,-0.71)	,287.4)	,-0.06)	,99.7)	,0.47)	,55)	,-0.11)	,56.5)	,1.38)
	2168.8	-0.23	113.8	0.02	1329.6	-0.42	111.6	0.02	304.5	0.05	108.1	0.34	0.7	0.07	14.4	0.15	185.9	0.06	406.8	-0.94	362.7	-0.99	44.1	-0.48
Kyrgyzstan	(1490.8	(-0.29	(72.2	(-0.04	(806.9	(-0.51	(63	(0.02	(166.6	(-0.03	(72.6	(0.33	(0.2	(0.04	(4.3	(-0.43	(119.8	(0.05	(264	(-1.25	(228.4	(-1.32	(24.9	(-0.71
	,2965.1)	,-0.17)	,163.2)	,0.08)	,2019)	,-0.33)	,175.3)	,0.03)	,509.6)	,0.13)	,153.2)	,0.35)	,1.5)	,0.11)	,28)	,0.74)	,277.3)	,0.08)	,590.2)	,-0.62)	,540.4)	,-0.66)	,67.7)	,-0.25)
	1816.7	0.02	149	0.34	624.5	-0.17	84.8	0.02	674.1	0.17	81.2	0.38	0.6	0.03	13.8	-2.57	188.8	0.01	106.6	-0.62	88.3	-0.72	18.3	-0.13
Lao People's Democratic Republic	(1293.8	(-0.01	(95.9	(0.29	(408.2	(-0.21	(47.3	(0.01	(379.5	(0.12	(53.9	(0.36	(0.2	(-0.01	(4.8	(-2.98	(120.8	(0.01	(65.3	(-0.67	(51.2	(-0.79	(8.4	(-0.2
	,2458.3)	,0.05)	,205.6)	,0.38)	,926.8)	,-0.12)	,134.3)	,0.03)	,1101)	,0.22)	,116.6)	,0.41)	,1.3)	,0.07)	,25.7)	,-2.17)	,283.3)	,0.02)	,157.9)	,-0.57)	,136.8)	,-0.65)	,31.2)	,-0.05)
	2574.1	-0.36	119.8	0.25	1456.4	-0.7	110.7	0.02	585.2	0.08	115	0.29	0.7	0.1	5	-1.72	181.2	0.06	412.6	0.26	353.5	0.31	59.1	-0.07
Latvia	(1789.9	(-0.47	(76.9	(0.21	(879.8	(-0.86	(62.7	(0.01	(322.4	(-0.01	(77.3	(0.27	(0.2	(0.07	(0.6	(-2.04	(115.4	(0.05	(270	(0.11	(220.3	(0.16	(35.7	(-0.34
	,3469.6)	,-0.25)	,169.4)	,0.29)	,2179.6)	,-0.53)	,174.1)	,0.03)	,960.9)	,0.18)	,162.7)	,0.31)	,1.5)	,0.13)	,11.8)	,-1.4)	,270.3)	,0.07)	,590.4)	,0.4)	,519.9)	,0.46)	,88.5)	,0.21)
	2462.8	0.17	129.2	0.03	1153.2	0.35	171.5	-0.04	691.1	0.06	110.5	0.22	1.1	-0.18	23	-1.33	183.1	-0.06	72.5	0.37	32.5	-0.38	40	1.12
Lebanon	(1748.1	(0.07	(82.4	(0.01	(722.2	(0.19	(102.7	(-0.04	(390.6	(-0.07	(74.4	(0.18	(0.4	(-0.21	(8.5	(-1.47	(116.8	(-0.08	(46.7	(0.34	(17.5	(-0.42	(22.7	(1.07
	,3373.7)	,0.26)	,181.2)	,0.05)	,1799.4)	,0.5)	,263.4)	,-0.03)	,1152.7)	,0.19)	,157)	,0.25)	,2.1)	,-0.15)	,41.5)	,-1.2)	,271.7)	,-0.05)	,103.2)	,0.4)	,52.9)	,-0.34)	,61.1)	,1.16)
	3067	0.08	110.3	0.08	2097.3	0.13	118	-0.09	470.7	0.04	88.7	-0.03	0.3	0.06	4.4	-1.46	177.4	-0.06	246.3	0.57	197.2	0.5	49.1	0.87
Lesotho	(2080.7	(0.03	(69	(0.06	(1297	(0.06	(67.1	(-0.1	(258	(-0.04	(59	(-0.09	(0.1	(0.01	(0.5	(-1.5	(112.6	(-0.07	(157.7	(0.5	(118.4	(0.43	(29.3	(0.81
	,4235.2)	,0.14)	,157.7)	,0.09)	,3151.6)	,0.2)	,183.7)	,-0.09)	,783.2)	,0.12)	,125.8)	,0.03)	,0.6)	,0.12)	,11.3)	,-1.42)	,263.7)	,-0.04)	,355.9)	,0.64)	,300)	,0.58)	,72.2)	,0.93)
	2592.4	0.25	110.8	0.08	1730.5	0.33	111.3	0	339.1	0.17	105.4	0.25	0.3	0	7.6	1.2	187.5	-0.03	97.4	-0.07	77.3	-0.05	20.1	-0.13
Liberia	(1780.8	(0.22	(69	(0.04	(1098.7	(0.28	(63.3	(-0.01	(187.1	(0.14	(70.4	(0.24	(0.1	(-0.05	(0.9	(0.75	(117.6	(-0.04	(61.5	(-0.14	(45.7	(-0.13	(10.3	(-0.18
	,3561.2)	,0.29)	,159.8)	,0.12)	,2582.2)	,0.38)	,175.4)	,0.01)	,568.3)	,0.2)	,148.3)	,0.26)	,0.6)	,0.04)	,18.9)	,1.64)	,280.2)	,-0.02)	,141.8)	,0)	,118.6)	,0.03)	,33.6)	,-0.09)
	2132.7	0.11	129.2	-0.25	1009.1	0.21	145.8	-0.05	543.6	0.11	95.5	0.09	1.1	-0.14	21.7	1.1	186.5	-0.08	73.4	0.46	32.8	-0.32	40.6	1.25
Libya	(1488.1	(0.08	(84.3	(-0.3	(625.9	(0.18	(84.7	(-0.06	(307.4	(0.04	(62.6	(0.05	(0.4	(-0.18	(7.8	(0.75	(118.7	(-0.09	(46.5	(0.41	(17.7	(-0.39	(23.2	(1.19
	,2886.2)	,0.14)	,180.4)	,-0.19)	,1524.2)	,0.24)	,229.3)	,-0.05)	,891.9)	,0.18)	,134.8)	,0.14)	,2.3)	,-0.11)	,40.6)	,1.45)	,280)	,-0.07)	,106.1)	,0.51)	,53.1)	,-0.26)	,62)	,1.31)
	2690.4	-0.08	121.6	0.25	1476.4	-0.21	110.8	0	679.2	0.09	115.7	0.16	0.7	0.06	4.6	-2.1	181.3	0.01	373.9	-0.63	317	-0.84	57	1
Lithuania	(1873.7	(-0.16	(75.6	(0.21	(928	(-0.31	(63.1	(-0.01	(381.7	(-0.01	(78.8	(0.14	(0.2	(0.03	(0.5	(-2.37	(115.8	(0	(249	(-0.7	(202.4	(-0.92	(35.2	(0.66
	,3766.1)	,-0.01)	,173.3)	,0.28)	,2275.4)	,-0.12)	,172.4)	,0.01)	,1124.2)	,0.19)	,162.7)	,0.18)	,1.5)	,0.1)	,11.2)	,-1.84)	,270.6)	,0.02)	,523.8)	,-0.57)	,463.2)	,-0.76)	,84.1)	,1.33)
	2128.4	-0.2	185.1	0.04	766.5	-0.6	211.7	0.07	651	-0.06	117	0.47	0.7	0.13	4.8	-1.96	191.6	0.1	254.6	0.28	196.6	0.19	58	0.61
Luxembourg	(1524.2	(-0.26	(120.6	(0.02	(496	(-0.7	(123.5	(0.05	(363.1	(-0.12	(78.4	(0.45	(0.2	(0.09	(1.2	(-2.1	(122.4	(0.09	(166.3	(0.18	(122.9	(0.11	(36	(0.41
	,2867.1)	,-0.15)	,256)	,0.05)	,1148)	,-0.5)	,325)	,0.08)	,1075.8)	,0.01)	,167.9)	,0.5)	,1.4)	,0.17)	,10.4)	,-1.81)	,285.6)	,0.1)	,361.8)	,0.38)	,294.8)	,0.27)	,84)	,0.81)
Madagascar	2822.3	0.05	112.4	0.09	1838.9	0	130.6	0.03	443.4	0.27	101.5	0.11	0.3	-0.01	6.1	0.5	189.1	0	208.3	-0.06	185.3	-0.09	23	0.2
	(1931.2	(0.02	(72.2	(0.07	(1126.2	(-0.04	(73.3	(0.02	(244.5	(0.22	(69	(0.08	(0.1	(-0.05	(0.7	(0.38	(121.7	(-0.01	(129.9	(-0.12	(114.2	(-0.16	(11.7	(0.15

	,3903)	,0.09)	,161.8)	,0.11)	,2721.9)	,0.04)	,204.9)	,0.04)	,748.4)	,0.32)	,142.6)	,0.13)	,0.6)	,0.03)	,15.7)	,0.62)	,281.7)	,0.01)	,312.1)	,0.01)	,285.4)	,-0.01)	,38)	,0.24)
	2514.9	0	107.6	0.12	1540.1	-0.1	129.2	0.03	447.7	0.28	98.1	0.17	0.3	-0.03	6.6	0.59	185.4	-0.01	215.1	0.08	191.1	0.06	24	0.23
Malawi	(1733.3	(-0.05	(68.3	(0.1	(959.8	(-0.17	(73.9	(0.02	(247.6	(0.23	(65.3	(0.15	(0.1	(-0.06	(0.8	(0.4	(119.2	(-0.01	(137.9	(0.01	(119.1	(-0.01	(12.7	(0.2
	,3370.4)	,0.05)	,154.7)	,0.14)	,2255.3)	,-0.03)	,201.6)	,0.04)	,740.1)	,0.33)	,141)	,0.19)	,0.6)	,-0.01)	,17.2)	,0.77)	,276)	,0)	,310.4)	,0.14)	,283.8)	,0.13)	,39.3)	,0.25)
	2122.5	0	169	0.23	915.3	-0.14	82.4	0	665.9	0.11	96	0.36	0.2	0.05	5.3	-2.2	188.3	0.04	70.6	-0.41	48.6	-0.58	22	0
Malaysia	(1501.1	(-0.09	(109.1	(0.2	(571	(-0.3	(46.2	(-0.01	(367.5	(0.02	(64.3	(0.34	(0.1	(0.01	(0.7	(-2.67	(121	(0.03	(43.6	(-0.45	(26.8	(-0.62	(10.3	(-0.08
	,2855.1)	,0.09)	,231.2)	,0.25)	,1367.3)	,0.01)	,129.7)	,0.02)	,1099.4)	,0.2)	,136)	,0.38)	,0.6)	,0.08)	,12.1)	,-1.72)	,281)	,0.05)	,105.8)	,-0.37)	,78.9)	,-0.54)	,37)	,0.08)
	1890.1	-0.09	168	0.2	815.3	-0.39	84.3	0	519.3	0.33	100.7	0.29	0.6	-0.17	9	-2.69	193.1	-0.09	75.2	-0.51	55.4	-0.74	19.7	0.19
Maldives	(1357.8	(-0.12	(110.3	(0.17	(519.3	(-0.45	(47.6	(-0.01	(301	(0.28	(67.3	(0.26	(0.2	(-0.21	(2.3	(-2.75	(122.1	(-0.1	(45.2	(-0.56	(29.9	(-0.79	(9.8	(0.11
	,2525.9)	,-0.06)	,234.2)	,0.22)	,1204.8)	,-0.34)	,136.3)	,0.01)	,858.2)	,0.38)	,143.3)	,0.31)	,1.3)	,-0.13)	,17.8)	,-2.62)	,289)	,-0.08)	,112.5)	,-0.47)	,89.8)	,-0.69)	,32.8)	,0.26)
	2077.2	0.07	118.2	0.19	1249.5	0.04	113.4	0.02	297.9	0.13	103.3	0.22	0.3	0.05	4.9	0.02	189.8	0.03	90.7	-0.01	72.6	0.05	18.1	-0.23
Mali	(1436.6	(0.02	(75.5	(0.18	(777.4	(-0.02	(65	(0.01	(165.2	(0.07	(69.3	(0.21	(0.1	(0.02	(0.5	(-0.18	(121.1	(0.02	(57.5	(-0.08	(43	(-0.04	(9	(-0.27
	,2878.7)	,0.12)	,169.3)	,0.21)	,1899.3)	,0.11)	,179)	,0.03)	,505.8)	,0.18)	,147.5)	,0.23)	,0.6)	,0.07)	,13.1)	,0.23)	,285.7)	,0.03)	,136.2)	,0.06)	,113.7)	,0.14)	,30.9)	,-0.2)
	2121.7	-0.03	178.5	0.04	698.2	-0.17	213.8	0.07	715.4	0.02	115.6	0.34	0.7	0.13	8	-2.05	191.5	0.05	134.3	0.12	96.8	0.03	37.5	0.35
Malta	(1498.4	(-0.06	(116.1	(0.03	(451.8	(-0.26	(124.6	(0.05	(409.4	(-0.02	(77	(0.32	(0.2	(0.1	(2.1	(-2.11	(122.3	(0.04	(85.8	(-0.02	(55.7	(-0.08	(21.6	(0.14
	,2830.7)	,0.01)	,248)	,0.05)	,1071.9)	,-0.09)	,334.4)	,0.09)	,1168.8)	,0.07)	,164.6)	,0.35)	,1.5)	,0.16)	,16.1)	,-1.98)	,285)	,0.06)	,194.3)	,0.25)	,151.2)	,0.14)	,57.2)	,0.56)
	1681.6	-0.07	141.8	-0.09	660.4	-0.13	68.9	-0.04	530.1	-0.07	80.7	0.19	0.8	0.02	11.6	0.12	187.2	0.02	109.1	0.02	78.6	0.01	30.5	0.04
Marshall Islands	(1207.9	(-0.09	(91.6	(-0.1	(419.6	(-0.16	(38.4	(-0.05	(301.6	(-0.11	(54.1	(0.17	(0.2	(-0.02	(3.4	(0.06	(121.1	(0.01	(70.8	(-0.01	(47.1	(-0.03	(16.8	(0
	,2250.6)	,-0.05)	,198.6)	,-0.08)	,975.8)	,-0.1)	,111)	,-0.03)	,886.2)	,-0.02)	,114.6)	,0.21)	,1.6)	,0.06)	,22.5)	,0.18)	,278.4)	,0.03)	,158.1)	,0.05)	,120.3)	,0.06)	,47.7)	,0.07)
	2254.1	0.05	126	0.15	1401.8	0.02	114.6	0.02	302.7	0.01	112.6	0.42	0.3	0.04	3.8	0.3	192.1	0.06	89.8	-0.21	67.8	-0.29	22	0.02
Mauritania	(1544.4	(0.01	(81.5	(0.13	(861.6	(-0.05	(65.2	(0.01	(169.1	(-0.03	(74.1	(0.4	(0.1	(0.01	(0.4	(0.22	(122.1	(0.05	(55.6	(-0.27	(39.3	(-0.37	(11.1	(0
	,3090.3)	,0.1)	,179.6)	,0.17)	,2105.8)	,0.09)	,176.3)	,0.03)	,510.2)	,0.06)	,160)	,0.43)	,0.6)	,0.07)	,11)	,0.38)	,285)	,0.07)	,133.9)	,-0.16)	,105.5)	,-0.21)	,36.3)	,0.04)
	2027.5	-0.2	162.2	0.13	964.1	-0.43	81.2	-0.03	534.8	0.02	92.5	0.15	0.6	0.02	8	-0.95	184.1	-0.03	170.6	-0.15	134.8	0.01	35.7	-0.7
Mauritius	(1458.2	(-0.29	(104.6	(0.11	(621.3	(-0.62	(45.8	(-0.04	(301.8	(-0.03	(62	(0.13	(0.2	(-0.01	(2.1	(-1.21	(117.5	(-0.03	(108	(-0.2	(79.7	(-0.08	(18.6	(-0.78
	,2696.1)	,-0.1)	,227.2)	,0.16)	,1425.7)	,-0.24)	,128.4)	,-0.02)	,856.5)	,0.07)	,130.6)	,0.17)	,1.2)	,0.05)	,16.3)	,-0.69)	,272.3)	,-0.02)	,251.4)	,-0.1)	,207.3)	,0.09)	,58.2)	,-0.63)
	2100.7	0.05	145.2	0.09	1052.9	-0.02	167	0.01	446	0.19	91.7	0.2	0.6	-0.01	4.7	-0.32	192.7	0	174.7	-1.35	150.4	-1.44	24.3	-0.76
Mexico	(1517.3	(-0.03	(105.6	(0.09	(701.2	(-0.16	(106.2	(0.01	(295.7	(0.08	(62.6	(0.18	(0.2	(-0.02	(0.9	(-0.55	(124.1	(0	(113.3	(-1.5	(94.4	(-1.61	(15.9	(-1.03
	,2723.7)	,0.12)	,186.7)	,0.1)	,1461.7)	,0.13)	,243.6)	,0.02)	,650.5)	,0.3)	,128.7)	,0.22)	,1.1)	,0)	,10.6)	,-0.09)	,287.6)	,0.01)	,250.1)	,-1.2)	,221.4)	,-1.27)	,34)	,-0.48)
	1724.1	-0.02	141.9	-0.11	685.9	-0.09	69.4	-0.04	548.2	0.09	80.6	0.1	0.8	0.01	12	0.2	185.4	-0.06	105.5	-0.13	74.9	-0.13	30.6	-0.11
Micronesia (Federated States of)	(1233.2	(-0.04	(90.6	(-0.12	(437.5	(-0.11	(38.5	(-0.05	(307.7	(0.07	(53.2	(0.07	(0.3	(-0.03	(3.6	(0.15	(118.1	(-0.07	(65.9	(-0.16	(43.4	(-0.17	(16.5	(-0.13
	,2328.2)	,0)	,202)	,-0.09)	,1021)	,-0.06)	,113.3)	,-0.03)	,901.2)	,0.12)	,115.4)	,0.12)	,1.7)	,0.05)	,23.2)	,0.25)	,277.4)	,-0.05)	,155.5)	,-0.1)	,118.4)	,-0.1)	,48.7)	,-0.08)
	2374.6	0.03	203.3	-0.01	970.2	0.01	212.4	0.06	678	0.02	116.2	0.21	0.7	0.04	1.9	-1.66	192	0.03	124.4	-0.2	89.4	-0.27	35	-0.02
Monaco	(1653.7	(-0.01	(130.7	(-0.03	(579.7	(-0.03	(122.3	(0.04	(383.9	(-0.05	(77.1	(0.18	(0.2	(0.01	(0.3	(-1.7	(121.8	(0.02	(77.6	(-0.34	(50.7	(-0.44	(19.5	(-0.1
	,3206.6)	,0.06)	,279.6)	,0)	,1519.2)	,0.05)	,332.1)	,0.07)	,1142.8)	,0.08)	,166.2)	,0.23)	,1.4)	,0.07)	,4.6)	,-1.61)	,285.2)	,0.03)	,185.6)	,-0.07)	,142.5)	,-0.11)	,55)	,0.05)
	2179.7	0.07	120	0.32	1363.9	0.09	110	0.01	293.7	-0.05	100.3	0.29	0.7	-0.16	6.8	-2.11	184.3	-0.04	396.7	1.27	357.8	1.42	38.9	0.1
Mongolia	(1510.7	(0.05	(77.8	(0.28	(835.6	(0.06	(63.3	(0	(166	(-0.17	(67	(0.26	(0.2	(-0.2	(1	(-2.49	(116.7	(-0.05	(244.8	(0.87	(209	(0.99	(21.4	(-0.01
	,3054.5)	,0.09)	,168.1)	,0.36)	,2087.7)	,0.12)	,174.5)	,0.02)	,493.1)	,0.08)	,143)	,0.32)	,1.5)	,-0.13)	,15.3)	,-1.72)	,277.2)	,-0.03)	,589.4)	,1.66)	,547.4)	,1.86)	,61)	,0.21)
	2131.4	0.01	127.1	0.11	996.9	-0.04	108.6	-0.02	577.2	0.07	127.8	0.23	0.7	0.05	7.3	-1.21	185.8	0.01	239.2	-0.21	220.2	-0.21	19	-0.15
Montenegro	(1521.5	(-0.05	(81.2	(0.08	(618.5	(-0.12	(62.3	(-0.03	(318.8	(-0.04	(86.2	(0.2	(0.2	(0.01	(1.3	(-1.53	(119.4	(0.01	(146.8	(-0.32	(129.6	(-0.34	(9.4	(-0.2
	,2897.1)	,0.07)	,179.9)	,0.14)	,1545.5)	,0.03)	,172)	,-0.02)	,936)	,0.17)	,180.9)	,0.25)	,1.5)	,0.08)	,16.3)	,-0.89)	,278.3)	,0.02)	,360.9)	,-0.09)	,338.3)	,-0.09)	,32.2)	,-0.1)

	2357.3	0	123.9	0.04	1272.2	-0.02	144.9	-0.05	511.7	0.11	92.2	0.18	1.1	0.02	24.6	-1.09	186.7	-0.06	69.3	0.19	34.7	-0.1	34.6	0.5
Morocco	(1639.4	(-0.04	(76.9	(0.03	(801.9	(-0.06	(82.8	(-0.06	(289.8	(0.03	(62.4	(0.16	(0.4	(-0.02	(9.2	(-1.18	(120.7	(-0.07	(42.8	(0.17	(18.8	(-0.14	(19.9	(0.48
	,3210.6)	,0.04)	,178.2)	,0.06)	,1935.5)	,0.03)	,226.1)	,-0.05)	,857.3)	,0.19)	,132.6)	,0.21)	,2.2)	,0.06)	,43.7)	,-1)	,279.4)	,-0.05)	,100.2)	,0.22)	,57.1)	,-0.06)	,54)	,0.53)
	2880.1	0.24	105.7	0.31	1911.6	0.29	126.8	0.03	452.4	0.26	95	0.03	0.3	-0.05	5.2	-1.62	183.2	-0.02	239.2	0.59	209.8	0.53	29.3	1.05
Mozambique	(1953.6	(0.22	(65.9	(0.28	(1183.8	(0.27	(72.8	(0.01	(251.8	(0.21	(63	(0.01	(0.1	(-0.08	(0.8	(-1.86	(118.5	(-0.04	(154.8	(0.5	(131.3	(0.44	(16.1	(0.98
	,3936.4)	,0.26)	,152.6)	,0.35)	,2847.9)	,0.32)	,197.6)	,0.04)	,747.5)	,0.3)	,135)	,0.05)	,0.6)	,-0.02)	,13.8)	,-1.38)	,273)	,-0.01)	,344.6)	,0.68)	,310.1)	,0.63)	,46.2)	,1.12)
	1579.4	0.1	144.7	0.41	509.5	0.1	81.8	0.01	568.5	0.21	79.1	0.28	0.6	-0.05	11.6	-3.5	183.6	-0.03	88.3	-0.54	69.4	-0.59	18.9	-0.37
Myanmar	(1117.4	(0.06	(92.1	(0.36	(329.2	(0.02	(45.4	(0.01	(318.1	(0.14	(51.5	(0.26	(0.2	(-0.09	(3.2	(-4.09	(117.1	(-0.04	(55.8	(-0.57	(40.5	(-0.62	(9.2	(-0.43
	,2115.9)	,0.15)	,207.3)	,0.46)	,760.7)	,0.18)	,132.4)	,0.02)	,928.2)	,0.28)	,113.6)	,0.29)	,1.2)	,-0.01)	,23.1)	,-2.9)	,273)	,-0.02)	,130.6)	,-0.51)	,108.3)	,-0.55)	,32.2)	,-0.32)
	2232.2	0.03	125.3	0.17	1231.6	-0.04	119.7	-0.02	468.5	0.23	101.6	0.17	0.3	-0.06	2.1	-1.04	183	-0.05	261.7	-0.05	212	-0.15	49.7	0.4
Namibia	(1553.2	(-0.03	(80.6	(0.15	(759.4	(-0.14	(68.8	(-0.03	(260	(0.14	(68.4	(0.15	(0.1	(-0.1	(0.2	(-1.15	(116.1	(-0.05	(169	(-0.13	(128.1	(-0.25	(30.5	(0.36
	,3050.9)	,0.09)	,179.7)	,0.19)	,1889.6)	,0.06)	,188)	,-0.01)	,800.9)	,0.32)	,145.3)	,0.19)	,0.6)	,-0.03)	,6.5)	,-0.93)	,269.8)	,-0.04)	,374.9)	,0.02)	,316.4)	,-0.06)	,73.3)	,0.44)
	1801	0.1	154.7	-0.4	755.7	0.17	69.3	-0.07	555.1	0.32	77.1	-0.31	0.8	-0.36	6.4	2.08	181.9	-0.2	106.5	-0.44	76.5	-0.67	29.9	0.2
Nauru	(1267.9	(0.07	(101.1	(-0.52	(466.9	(0.16	(37.7	(-0.08	(304.3	(0.29	(51	(-0.36	(0.2	(-0.39	(1.7	(1.29	(117.1	(-0.21	(67.7	(-0.47	(44.8	(-0.71	(16	(0.18
	,2450.5)	,0.12)	,217.9)	,-0.27)	,1129.3)	,0.19)	,111.7)	,-0.06)	,896.2)	,0.35)	,109.9)	,-0.26)	,1.6)	,-0.33)	,13.5)	,2.87)	,272.1)	,-0.2)	,152.8)	,-0.41)	,117)	,-0.63)	,47.4)	,0.21)
	2892.3	0.28	157.6	0.05	1869.4	0.38	93.5	0.01	476.3	0.33	72.3	0.22	0.4	-0.1	37.4	-1.04	185.2	-0.01	183.9	0.02	159.3	0.05	24.6	-0.2
Nepal	(1953.6	(0.14	(100.8	(0.03	(1152.6	(0.15	(52.6	(0.01	(267.1	(0.25	(48.4	(0.2	(0.1	(-0.14	(18.1	(-1.23	(118.9	(-0.02	(111.4	(-0.02	(92.7	(0	(12.1	(-0.28
	,4023.4)	,0.43)	,227.5)	,0.06)	,2808.4)	,0.6)	,148.2)	,0.02)	,811)	,0.41)	,102.8)	,0.24)	,0.9)	,-0.05)	,62.9)	,-0.85)	,277.5)	,0)	,279.5)	,0.05)	,251.8)	,0.11)	,42.2)	,-0.11)
	2356.9	-0.04	242	-0.2	798.3	-0.08	189.1	0.06	812	-0.04	118.1	0.35	0.8	0.15	5.2	-1.83	191.4	0.06	185.1	0.82	149.3	0.88	35.8	0.57
Netherlands	(1668	(-0.14	(166.6	(-0.26	(498.4	(-0.19	(115.9	(0.04	(465.7	(-0.28	(81	(0.33	(0.2	(0.1	(1.3	(-1.96	(122.9	(0.05	(121.8	(0.7	(92.8	(0.75	(20.6	(0.51
	,3182.5)	,0.06)	,331.2)	,-0.14)	,1236.4)	,0.03)	,286.8)	,0.08)	,1320.2)	,0.2)	,168.7)	,0.38)	,1.6)	,0.19)	,11.2)	,-1.71)	,285.6)	,0.07)	,261.8)	,0.93)	,218.4)	,1.01)	,55.7)	,0.63)
	1877.1	-0.03	199.9	0.06	466.1	-0.4	226	0.02	615.6	0.08	162.1	0.31	1.2	0.1	6.4	2.02	199.9	0.04	281.9	1.02	249.8	1.24	32.1	-0.27
New Zealand	(1369.6	(-0.08	(139.1	(0.04	(300.4	(-0.52	(141.7	(0.01	(387.7	(-0.01	(108.3	(0.29	(0.4	(0.05	(1.6	(1.38	(129.7	(0.03	(173.3	(0.6	(145.6	(0.73	(18.5	(-0.31
	,2434.9)	,0.01)	,268.4)	,0.08)	,692.9)	,-0.28)	,336.6)	,0.03)	,941.5)	,0.17)	,229)	,0.33)	,2.4)	,0.15)	,13.6)	,2.66)	,295.6)	,0.05)	,425)	,1.45)	,387.6)	,1.74)	,48.7)	,-0.22)
	2074.6	0.12	136.5	0.03	971.9	0.15	150	-0.03	531.1	0.19	91.3	0.17	1.2	-0.07	7.4	-0.52	185.3	-0.03	368.7	0.05	351	0.08	17.8	-0.39
Nicaragua	(1469.3	(0.08	(89.3	(0.01	(607	(0.09	(85.7	(-0.04	(302.2	(0.11	(60.8	(0.16	(0.4	(-0.1	(1	(-0.98	(116.2	(-0.04	(236.4	(-0.18	(221.9	(-0.17	(8.7	(-0.43
	,2828)	,0.16)	,193.3)	,0.04)	,1487.9)	,0.2)	,234.9)	,-0.02)	,892.8)	,0.27)	,131.1)	,0.19)	,2.3)	,-0.05)	,16.1)	,-0.06)	,278.5)	,-0.03)	,540.1)	,0.28)	,518.9)	,0.32)	,30.3)	,-0.34)
	2401.4	0.19	113.7	0.08	1567.2	0.23	114.1	0.02	309.4	0.21	100.4	0.1	0.3	-0.09	6.9	2.5	189.5	-0.02	94.2	-0.01	75.4	0.03	18.8	-0.15
Niger	(1692.3	(0.18	(71.1	(0.05	(1001	(0.21	(66.1	(0.02	(162.7	(0.16	(68	(0.08	(0.1	(-0.14	(1	(2.2	(121.9	(-0.03	(60.5	(-0.08	(45.9	(-0.07	(9.4	(-0.21
	,3300.1)	,0.2)	,161.7)	,0.11)	,2338.5)	,0.24)	,181.5)	,0.03)	,519.5)	,0.25)	,142.3)	,0.11)	,0.6)	,-0.05)	,18.1)	,2.8)	,282.6)	,-0.02)	,138.6)	,0.06)	,115.9)	,0.12)	,31.3)	,-0.08)
	2289.7	-0.11	133	0.33	1413	-0.27	116.5	0.04	326.7	0.24	102.6	0.15	0.2	-0.25	4.3	0.35	193.1	0	103.3	0.18	82.7	0.12	20.6	0.4
Nigeria	(1629.6	(-0.2	(95.5	(0.27	(934.9	(-0.43	(73.5	(0.03	(213.7	(0.08	(70.2	(0.11	(0.1	(-0.32	(0.8	(0.25	(124.8	(-0.02	(68.4	(0.09	(52.3	(0.02	(13.3	(0.36
	,3017.7)	,-0.02)	,173.4)	,0.4)	,2004.1)	,-0.11)	,172)	,0.04)	,472.9)	,0.41)	,142.9)	,0.2)	,0.5)	,-0.19)	,10.2)	,0.46)	,289.1)	,0.02)	,146)	,0.27)	,122)	,0.23)	,29.4)	,0.43)
	1809.5	0.01	159.8	0.01	749.4	0	70.1	-0.03	551.6	0.04	86.5	0.12	0.8	-0.03	6.8	-0.46	184.6	-0.04	99.6	-0.13	71	-0.13	28.6	-0.13
Niue	(1298.3	(-0.01	(104.7	(-0.01	(473.5	(-0.01	(38.7	(-0.04	(303	(-0.01	(57.3	(0.1	(0.2	(-0.08	(1.6	(-0.66	(119.3	(-0.04	(63.9	(-0.18	(41.7	(-0.19	(15.3	(-0.15
	,2437.1)	,0.03)	,226.4)	,0.03)	,1128.6)	,0.02)	,113.2)	,-0.02)	,893.3)	,0.08)	,123.2)	,0.14)	,1.6)	,0.01)	,14.4)	,-0.26)	,274.3)	,-0.03)	,145)	,-0.08)	,108.9)	,-0.06)	,45.9)	,-0.11)
	2007.8	-0.04	124.6	0.11	893.1	-0.23	108	-0.01	562.9	0.14	123.6	0.32	0.8	0.09	8.6	-0.8	186.1	-0.01	230.6	-0.57	208.2	-0.62	22.4	-0.07
North Macedonia	(1417.9	(-0.11	(80.3	(0.09	(568	(-0.31	(62.1	(-0.02	(318.9	(0.04	(84	(0.31	(0.2	(0.06	(1.7	(-0.98	(118	(-0.02	(144.6	(-0.69	(126.1	(-0.75	(11	(-0.15
	,2693.1)	,0.02)	,176.5)	,0.13)	,1362.1)	,-0.15)	,170.8)	,0)	,911.2)	,0.25)	,175)	,0.34)	,1.5)	,0.12)	,18.2)	,-0.62)	,280.3)	,0)	,348.2)	,-0.44)	,322.3)	,-0.49)	,36.9)	,0.02)
Northern Mariana Islands	1764.5	0.09	178.6	-0.34	664.9	0.23	71.9	-0.02	555.7	0.19	98.1	-0.12	0.8	-0.17	3.2	1.97	191.2	-0.07	138.5	-0.1	106.8	-0.22	31.7	0.29
	(1262.8	(0.06	(117.1	(-0.38	(421.4	(0.2	(39.5	(-0.03	(313.7	(0.14	(65.4	(-0.16	(0.3	(-0.24	(0.5	(1.63	(122.6	(-0.08	(89.6	(-0.14	(64.9	(-0.26	(17.4	(0.25

	,2380.1)	,0.12)	,248)	,-0.31)	,993.9)	,0.26)	,116)	,-0.01)	,918.2)	,0.23)	,140.9)	,-0.08)	,1.7)	,-0.1)	,8)	,2.31)	,287.9)	,-0.06)	,198.6)	,-0.07)	,158.8)	,-0.17)	,50.2)	,0.34)
	2255.1	0.09	181.6	0.08	744.9	0.26	197.8	0.18	826.6	-0.11	104.4	0.37	0.3	0.11	5.7	-1.62	193.7	0.09	223.2	0.41	140.3	-0.46	82.9	2.51
Norway	(1635.7	(0.06	(131.4	(0.06	(484	(0.17	(124.2	(0.15	(545.4	(-0.17	(71.4	(0.35	(0.1	(0.09	(1.5	(-1.74	(125.5	(0.08	(152.5	(0.28	(87.7	(-0.52	(55	(2.07
	,2921.5)	,0.12)	,232)	,0.09)	,1060.3)	,0.36)	,290.7)	,0.2)	,1186.4)	,-0.05)	,145.8)	,0.39)	,0.8)	,0.12)	,11.6)	,-1.5)	,286.1)	,0.09)	,304.3)	,0.53)	,206.7)	,-0.39)	,113.8)	,2.96)
	2092.5	0.19	139.5	0.13	994.1	0.32	136	0.01	518	0.13	103.7	0.24	1.2	-0.07	12.4	-1.31	187.7	-0.02	63.5	-0.16	39.4	-0.21	24.1	-0.07
Oman	(1487.2	(0.16	(87.4	(0.1	(621.8	(0.29	(79.8	(0	(289.4	(0.06	(68.9	(0.21	(0.4	(-0.11	(2.8	(-1.48	(119.6	(-0.03	(38.6	(-0.27	(21.9	(-0.34	(11.2	(-0.17
	,2828.8)	,0.22)	,196.7)	,0.16)	,1526.7)	,0.35)	,212.6)	,0.01)	,843.5)	,0.2)	,145.8)	,0.28)	,2.3)	,-0.03)	,25)	,-1.15)	,280)	,0)	,95.1)	,-0.05)	,63.3)	,-0.09)	,41)	,0.04)
	2403.3	-0.12	164.7	0.03	1360.3	-0.28	96.5	-0.02	479.4	0.16	71.6	0.05	0.3	-0.07	35.8	0.98	194.7	-0.05	245.1	-0.05	216.7	-0.03	28.4	-0.17
Pakistan	(1694.4	(-0.18	(115.5	(0.02	(871.6	(-0.37	(59.1	(-0.03	(297	(0.11	(48.3	(0.02	(0.1	(-0.11	(15.6	(0.92	(125.9	(-0.06	(158.8	(-0.08	(136.9	(-0.06	(16.7	(-0.33
	,3184.1)	,-0.06)	,216.9)	,0.04)	,1939.6)	,-0.19)	,144.5)	,-0.01)	,723.1)	,0.2)	,100.6)	,0.07)	,0.7)	,-0.03)	,62.4)	,1.04)	,291.8)	,-0.05)	,351.3)	,-0.02)	,318.1)	,0)	,43.8)	,-0.01)
	1776.5	-0.01	162.3	-0.07	732.3	-0.02	69.9	-0.04	528.6	0.01	89.7	0.09	0.8	-0.03	5.4	0.22	187.4	-0.04	90.9	-0.27	61.2	-0.34	29.7	-0.11
Palau	(1262.6	(-0.03	(103.7	(-0.09	(467.3	(-0.04	(38.3	(-0.05	(290.6	(-0.02	(59.7	(0.07	(0.3	(-0.07	(1.1	(0.13	(119.6	(-0.04	(57.3	(-0.29	(35.1	(-0.38	(16.3	(-0.14
	,2404)	,0)	,229.9)	,-0.06)	,1111.5)	,0)	,111.9)	,-0.04)	,923.2)	,0.04)	,128.6)	,0.1)	,1.7)	,0)	,12)	,0.31)	,282.6)	,-0.03)	,130.6)	,-0.24)	,95.2)	,-0.3)	,46.8)	,-0.08)
	2749.6	0.01	122.2	0.02	1595	0.02	146.7	-0.05	559.2	0.02	97.3	0.24	1.2	0.14	42.7	-0.46	185.4	-0.01	54.8	-0.26	30.1	-0.23	24.7	-0.3
Palestine	(1884.5	(-0.06	(79.6	(0	(973.8	(-0.1	(84.4	(-0.06	(315.2	(-0.03	(64.6	(0.2	(0.4	(0.1	(20.6	(-0.55	(118.1	(-0.02	(33.8	(-0.31	(16.2	(-0.26	(12.2	(-0.4
	,3820)	,0.08)	,173.3)	,0.04)	,2403.8)	,0.13)	,231.1)	,-0.04)	,907.1)	,0.07)	,138.4)	,0.27)	,2.3)	,0.18)	,70.7)	,-0.36)	,277.1)	,0)	,81.1)	,-0.21)	,48.6)	,-0.19)	,41.7)	,-0.21)
	2021.1	0.03	152.1	0.16	951	-0.06	152.8	-0.02	471.9	0.2	99.2	0.14	1.2	-0.09	4.2	-1.16	188.8	-0.04	206	-0.35	185.6	-0.39	20.4	-0.03
Panama	(1418.2	(-0.02	(99	(0.14	(598.8	(-0.12	(88.3	(-0.03	(265.8	(0.11	(67.6	(0.12	(0.4	(-0.12	(0.7	(-1.45	(119.7	(-0.04	(128.2	(-0.5	(113.1	(-0.55	(11	(-0.05
	,2667)	,0.08)	,211.8)	,0.18)	,1436.2)	,0.01)	,234.3)	,-0.02)	,750.7)	,0.29)	,141.4)	,0.15)	,2.4)	,-0.06)	,9.9)	,-0.86)	,281.4)	,-0.03)	,302.7)	,-0.21)	,280.3)	,-0.23)	,32.8)	,0)
	1711.1	-0.01	147.7	-0.02	686.8	-0.06	70.3	0	521.9	0.02	82.1	0.11	0.8	0.07	10.9	0.35	190.5	0.04	98.3	0.16	74.7	0.2	23.6	0.04
Papua New Guinea	(1215.9	(-0.03	(96.2	(-0.04	(429.1	(-0.08	(38.3	(0	(299	(-0.01	(55	(0.1	(0.2	(0.03	(3	(0.18	(122.1	(0.03	(60.1	(0.12	(43.5	(0.16	(11.8	(0.01
	,2315.1)	,0.01)	,206)	,0)	,1016)	,-0.05)	,113.3)	,0.01)	,865.5)	,0.06)	,115.9)	,0.12)	,1.7)	,0.12)	,21.7)	,0.53)	,283.4)	,0.04)	,145.5)	,0.2)	,116.5)	,0.24)	,38.9)	,0.08)
	2318.3	0.13	143.6	0.06	853.3	0.24	182.1	-0.03	867.4	0.11	75.6	0.15	1.8	0	7.8	-0.22	186.7	-0.02	335.4	0.89	317.1	0.97	18.3	-0.22
Paraguay	(1609.3	(0.08	(93.5	(0.05	(533.5	(0.1	(106	(-0.03	(500.5	(0.04	(50	(0.14	(0.7	(-0.03	(1.9	(-0.36	(118.9	(-0.03	(204.6	(0.72	(188.7	(0.79	(9.9	(-0.26
	,3197.6)	,0.19)	,203.5)	,0.08)	,1296.3)	,0.37)	,277.9)	,-0.02)	,1455.1)	,0.19)	,108.8)	,0.16)	,3.4)	,0.02)	,15.5)	,-0.09)	,278.4)	,-0.01)	,502)	,1.06)	,480.1)	,1.15)	,30)	,-0.19)
	1966.7	-0.03	147.2	0.18	650.8	-0.38	167.9	0	714.8	0.21	87.2	0.39	1.8	-0.02	6.7	-1.49	190.3	0.01	133	-1.06	113.2	-1.24	19.7	0.16
Peru	(1395.4	(-0.12	(95.4	(0.16	(411.1	(-0.51	(96.4	(0	(401.9	(0.08	(58.6	(0.38	(0.7	(-0.03	(1.4	(-1.61	(122	(0	(84.8	(-1.18	(69.1	(-1.36	(11	(0.09
	,2661.8)	,0.05)	,208.3)	,0.2)	,978.1)	,-0.24)	,265.4)	,0.01)	,1189.6)	,0.34)	,125.6)	,0.41)	,3.5)	,0)	,13.8)	,-1.37)	,281.9)	,0.01)	,194.3)	,-0.95)	,171.4)	,-1.11)	,31.9)	,0.23)
	1927.1	-0.09	150.5	0.11	788	-0.36	85.5	0.01	617.3	0.21	82.5	0.03	0.5	-0.02	10.6	-1.37	192.2	-0.02	97.9	-0.7	79.7	-0.79	18.2	-0.28
Philippines	(1405.8	(-0.14	(109.2	(0.08	(528.9	(-0.44	(53.5	(0.01	(411.1	(0.14	(56.9	(0.01	(0.2	(-0.03	(2.6	(-1.95	(124.5	(-0.03	(63.7	(-0.74	(50	(-0.85	(11.4	(-0.35
	,2520.6)	,-0.05)	,194.2)	,0.14)	,1102.3)	,-0.29)	,126.5)	,0.01)	,894.9)	,0.27)	,116)	,0.04)	,0.9)	,-0.01)	,21.5)	,-0.78)	,286.9)	,-0.02)	,139)	,-0.66)	,116.3)	,-0.73)	,26.8)	,-0.2)
	1679.2	0.02	130.4	0.28	609.1	-0.13	112.5	0.03	507.9	0.06	122.5	0.41	0.6	0.09	6	-2.24	190.2	0.06	378.9	0.91	360.1	0.98	18.9	-0.28
Poland	(1239.9	(-0.06	(93.2	(0.27	(411.7	(-0.35	(71.4	(0.02	(332.4	(-0.02	(85.1	(0.4	(0.2	(0.09	(1.1	(-2.33	(122.6	(0.06	(240.4	(0.7	(225.6	(0.76	(12.3	(-0.33
	,2161.1)	,0.11)	,167.7)	,0.29)	,863.8)	,0.09)	,165.1)	,0.03)	,731.8)	,0.13)	,170.3)	,0.43)	,1.2)	,0.1)	,13.2)	,-2.15)	,284.3)	,0.07)	,541.5)	,1.11)	,519.8)	,1.19)	,26.7)	,-0.22)
	2958	-0.2	173.2	0.01	1276	-0.65	201.9	-0.02	1000.1	0.23	108	0.36	0.7	0.07	9.7	-1.45	188.5	0.04	239.3	0.19	209.6	0.15	29.7	0.49
Portugal	(2050.5	(-0.35	(114.7	(0	(793.1	(-0.93	(122.2	(-0.03	(586	(0.08	(72.6	(0.34	(0.2	(0.04	(3.1	(-1.57	(120.7	(0.04	(153.1	(0.05	(128.8	(0	(15.8	(0.38
	,4014)	,-0.06)	,242.5)	,0.03)	,1952.6)	,-0.37)	,304.2)	,-0.01)	,1573)	,0.38)	,153.6)	,0.38)	,1.4)	,0.1)	,18.6)	,-1.33)	,281.4)	,0.05)	,350.3)	,0.33)	,313.7)	,0.29)	,46.8)	,0.61)
	1920.9	0.02	150.3	0.05	756.1	-0.07	166.9	-0.03	562	0.15	93.9	0.18	2.5	-0.05	2.6	-1	186.6	-0.04	222.9	-1.24	197.5	-1.33	25.4	-0.43
Puerto Rico	(1347.6	(-0.03	(98.7	(0.04	(467	(-0.16	(97.1	(-0.04	(314.4	(0.09	(62.6	(0.17	(1.1	(-0.07	(0.2	(-1.23	(117.6	(-0.05	(144.9	(-1.41	(124.2	(-1.53	(14.3	(-0.66
	,2570.1)	,0.07)	,216)	,0.07)	,1127.5)	,0.02)	,261.9)	,-0.03)	,897.4)	,0.21)	,132.8)	,0.19)	,4.8)	,-0.03)	,6.9)	,-0.77)	,279.5)	,-0.03)	,328.8)	,-1.07)	,296.7)	,-1.13)	,40.2)	,-0.2)

	2001	-0.03	152	0.15	922.8	-0.11	144	-0.05	453.8	0.01	127.5	0.41	1.2	0.06	8	-1.97	191.6	-0.04	60.8	-0.14	36	-0.29	24.8	0.1
Qatar	(1426.7	(-0.06	(98.7	(0.12	(573.6	(-0.16	(82.3	(-0.05	(259	(-0.05	(86.4	(0.37	(0.4	(0.01	(1.4	(-2.18	(122.7	(-0.06	(37.1	(-0.2	(18.8	(-0.36	(12.1	(0
	,2656.3)	,0)	,216.4)	,0.18)	,1393.4)	,-0.07)	,226.1)	,-0.04)	,745.2)	,0.07)	,178.6)	,0.45)	,2.4)	,0.11)	,16.8)	,-1.76)	,282.6)	,-0.02)	,89.8)	,-0.07)	,59.5)	,-0.23)	,41.4)	,0.2)
	1547.8	0.42	152	0.03	525	1.05	112.8	0.01	365.9	-0.11	201.8	0.77	0.6	0.22	0.4	-8.73	189.2	0.11	292.3	-0.24	264.1	-0.2	28.3	-0.63
Republic of Korea	(1132.8	(0.3	(98.2	(0.02	(330.1	(0.68	(63.6	(0	(204	(-0.21	(137.5	(0.73	(0.1	(0.16	(0	(-9.24	(119.9	(0.11	(184.7	(-0.46	(162.5	(-0.44	(15.7	(-0.78
	,1993.8)	,0.53)	,212.4)	,0.04)	,790.4)	,1.42)	,177)	,0.02)	,616.5)	,0)	,281.5)	,0.81)	,1.3)	,0.27)	,2)	,-8.22)	,279.7)	,0.12)	,424.2)	,-0.02)	,392)	,0.04)	,44.7)	,-0.48)
	2285.3	-0.18	109.8	0.1	1174.4	-0.42	110.1	0.01	584.4	0.09	111.7	0.25	0.7	0.04	11.4	-0.65	182.8	0.02	374.6	0.44	334.6	0.58	40	-0.64
Republic of Moldova	(1597.2	(-0.25	(69.3	(0.06	(744.9	(-0.52	(61.9	(0	(337.4	(0.01	(75.2	(0.22	(0.2	(0.01	(2.8	(-1.13	(116.6	(0.01	(253.6	(0.26	(220.9	(0.38	(22.3	(-0.73
	,3071.7)	,-0.12)	,156.8)	,0.15)	,1801.6)	,-0.33)	,173.2)	,0.02)	,973.7)	,0.17)	,156.8)	,0.27)	,1.5)	,0.07)	,23.2)	,-0.17)	,273.6)	,0.03)	,540.6)	,0.63)	,492.7)	,0.79)	,63.5)	,-0.54)
	1998.1	0.08	129.5	0.24	901.7	0	128.4	0.03	526.3	0.18	119.2	0.35	0.7	0	6.2	-2	185.9	0.01	277.1	-1.52	255.6	-1.6	21.5	-0.36
Romania	(1413.7	(0.02	(83.9	(0.21	(574.6	(-0.07	(75	(0.02	(291.5	(0.07	(80.8	(0.33	(0.2	(-0.03	(1.1	(-2.25	(118	(0.01	(181.1	(-1.72	(163.7	(-1.8	(10.9	(-0.55
	,2678.3)	,0.14)	,181.1)	,0.27)	,1363.9)	,0.07)	,200.2)	,0.04)	,897)	,0.29)	,170.7)	,0.36)	,1.5)	,0.04)	,13.8)	,-1.75)	,275.8)	,0.02)	,405.2)	,-1.32)	,380.9)	,-1.39)	,34.9)	,-0.18)
	2017.7	-0.16	117.2	0.34	969.5	-0.42	112.9	0	514.5	0.01	111.7	0.43	0.6	0.08	4.8	-1.35	186.5	0.06	503.2	-0.36	438.4	-0.22	64.8	-1.19
Russian Federation	(1456.2	(-0.22	(84.8	(0.25	(637.4	(-0.53	(70.9	(-0.01	(341.6	(-0.08	(77.3	(0.39	(0.2	(0.06	(0.5	(-1.84	(120.5	(0.05	(327.3	(-0.53	(277.1	(-0.39	(43.1	(-1.47
	,2608.2)	,-0.1)	,150)	,0.43)	,1363.4)	,-0.31)	,166.2)	,0.01)	,748.5)	,0.11)	,155.7)	,0.46)	,1.1)	,0.09)	,11.4)	,-0.85)	,279.2)	,0.06)	,704.4)	,-0.18)	,627.3)	,-0.06)	,89.7)	,-0.91)
	2973.9	-0.26	113.2	0.23	2003.6	-0.45	130.2	0.03	436	0.26	98.5	0.36	0.3	-0.02	7.5	-1.07	184.6	-0.01	436.9	-0.12	414.9	-0.14	22	0.21
Rwanda	(1994.2	(-0.31	(70.5	(0.18	(1224.8	(-0.52	(76.8	(0.01	(250.7	(0.21	(65.7	(0.3	(0.1	(-0.06	(1.4	(-1.43	(116.7	(-0.03	(270.2	(-0.25	(253.7	(-0.27	(11.1	(0.18
	,4221.5)	,-0.2)	,159.5)	,0.29)	,3069.1)	,-0.38)	,204.9)	,0.05)	,720.9)	,0.3)	,140.8)	,0.42)	,0.6)	,0.01)	,17.1)	,-0.7)	,274.8)	,0.01)	,636.9)	,0.01)	,611.5)	,0)	,36.2)	,0.25)
	2350.7	0.04	145.5	0.15	1210.1	0	163.4	-0.03	557.3	0.08	82.5	0.27	2.6	0.12	2.9	-1.02	186.3	0	425.8	0.03	403.5	0.03	22.2	-0.02
Saint Kitts and Nevis	(1639.3	(0	(92.7	(0.13	(734.6	(-0.04	(94.7	(-0.03	(310.9	(0.01	(55	(0.23	(1.1	(0.09	(0.2	(-1.08	(118.4	(-0.01	(268.1	(-0.08	(249.2	(-0.08	(11.9	(-0.09
	,3226.3)	,0.07)	,204.6)	,0.16)	,1906)	,0.05)	,255.7)	,-0.02)	,925)	,0.15)	,118.6)	,0.3)	,4.8)	,0.14)	,7.6)	,-0.96)	,278.5)	,0)	,623.6)	,0.14)	,598)	,0.15)	,35.6)	,0.04)
	2079.6	0.06	139.4	0.09	930.3	0.06	163.9	-0.02	566	0.07	84.9	0.25	2.6	0.13	5.1	-0.6	187.3	0.03	244.9	-0.19	223.4	-0.2	21.4	-0.14
Saint Lucia	(1443.8	(0.01	(89.3	(0.08	(570.4	(-0.02	(96	(-0.02	(316	(-0.01	(56.8	(0.22	(1.1	(0.11	(0.6	(-0.66	(118.4	(0.02	(156.8	(-0.32	(140.6	(-0.34	(11.9	(-0.19
	,2813.7)	,0.12)	,194.5)	,0.1)	,1420.7)	,0.15)	,253.5)	,-0.01)	,925.1)	,0.15)	,122)	,0.28)	,4.8)	,0.15)	,11.9)	,-0.53)	,278.6)	,0.04)	,351.4)	,-0.06)	,325.4)	,-0.05)	,34.6)	,-0.09)
	2068	0.08	137.9	0.14	941.3	0.15	162.5	-0.04	545.1	-0.02	84.1	0.27	2.7	0.21	5.1	-0.77	189.4	0.06	255.9	0.15	234.7	0.18	21.2	-0.14
Saint Vincent and the Grenadines	(1445.9	(0.04	(88.5	(0.12	(578	(0.08	(95	(-0.04	(306.7	(-0.09	(56.1	(0.26	(1.2	(0.18	(0.6	(-0.84	(119.7	(0.05	(160.9	(0.05	(142.1	(0.06	(11.8	(-0.2
	,2792.7)	,0.13)	,193.6)	,0.16)	,1435.4)	,0.22)	,254)	,-0.03)	,873.8)	,0.04)	,120.3)	,0.29)	,5)	,0.23)	,12)	,-0.69)	,283.3)	,0.07)	,370.9)	,0.26)	,346.5)	,0.3)	,34.3)	,-0.08)
	1703.4	-0.06	150.9	-0.03	657.4	-0.2	70.4	-0.02	541.4	0.08	86.4	0.1	0.8	0	9.6	-0.62	186.5	-0.02	98.8	-0.16	71.1	-0.2	27.7	-0.05
Samoa	(1216.1	(-0.08	(95.9	(-0.04	(417.6	(-0.24	(38.4	(-0.03	(308.1	(0.05	(57.3	(0.09	(0.2	(-0.02	(2.7	(-0.72	(117.7	(-0.03	(64	(-0.18	(42.3	(-0.24	(15.2	(-0.08
	,2292.3)	,-0.04)	,210.5)	,-0.01)	,975.1)	,-0.16)	,115.6)	,-0.02)	,898.6)	,0.11)	,123)	,0.11)	,1.6)	,0.03)	,18.9)	,-0.52)	,277.1)	,-0.02)	,142.8)	,-0.13)	,110.4)	,-0.16)	,43.9)	,-0.02)
	2359.8	0.04	181.9	-0.07	968.9	0.02	214.7	0.09	676.3	0.06	118.4	0.19	0.7	0.03	6.7	-0.65	192.2	0.04	125.6	-0.13	92.4	-0.17	33.2	0
San Marino	(1637	(0	(118.5	(-0.09	(578.5	(-0.04	(126.5	(0.07	(381	(-0.02	(81.6	(0.16	(0.2	(0.01	(1.8	(-1.02	(122.5	(0.03	(78.7	(-0.26	(52.3	(-0.32	(18.8	(-0.12
	,3240.3)	,0.08)	,253.5)	,-0.04)	,1538.3)	,0.08)	,334.4)	,0.11)	,1148.3)	,0.14)	,168.2)	,0.21)	,1.4)	,0.06)	,13.8)	,-0.27)	,286.6)	,0.04)	,186.7)	,0.01)	,146)	,-0.02)	,52)	,0.11)
	2131.9	0.1	125.3	0.14	1261.5	0.14	114.8	-0.02	327.2	0.04	110.2	0.23	0.3	0.01	4.9	-0.45	187.7	-0.01	104.3	0.1	75.5	-0.07	28.9	0.56
Sao Tome and Principe	(1483.4	(0.07	(81.1	(0.11	(785.1	(0.1	(66.1	(-0.03	(180.5	(-0.01	(73.7	(0.21	(0.1	(-0.02	(0.4	(-0.59	(120.7	(-0.02	(67.3	(0.03	(45	(-0.16	(15.7	(0.48
	,2893.9)	,0.14)	,178.7)	,0.17)	,1900.6)	,0.19)	,180.5)	,-0.01)	,543.2)	,0.08)	,159.8)	,0.25)	,0.6)	,0.04)	,12.6)	,-0.3)	,282.7)	,0)	,151.2)	,0.16)	,116.3)	,0.02)	,45)	,0.63)
	1996.6	0.19	139.9	0.09	949	0.31	146.6	-0.03	454.3	0.05	106.7	0.51	1.1	0.12	7.5	-1.06	191.5	0.04	60.3	-0.09	33.5	-0.25	26.7	0.13
Saudi Arabia	(1430.5	(0.16	(89.8	(0.07	(600.9	(0.28	(85.3	(-0.04	(260.8	(0	(72.5	(0.47	(0.3	(0.05	(1.1	(-1.2	(123.4	(0.02	(36	(-0.13	(17.9	(-0.28	(12.9	(0.06
	,2680.2)	,0.21)	,195.5)	,0.11)	,1430.1)	,0.35)	,229.8)	,-0.02)	,788.9)	,0.09)	,152.7)	,0.56)	,2.2)	,0.19)	,16.4)	,-0.93)	,285.5)	,0.05)	,90.3)	,-0.04)	,54.8)	,-0.23)	,44.3)	,0.21)
	2441.1	0.1	122.2	0.11	1585.9	0.11	113.5	0	324.6	0.12	104.2	0.09	0.3	-0.06	2.8	1.51	187.7	-0.03	93.7	-0.02	73	0.05	20.7	-0.28
Senegal	(1664.5	(0.06	(79.1	(0.1	(969.8	(0.07	(65.1	(-0.01	(177.3	(0.05	(69.3	(0.08	(0.1	(-0.1	(0.4	(1.34	(120.8	(-0.04	(59.9	(-0.09	(43.9	(-0.04	(10.3	(-0.3

	,3358.3)	,0.13)	,172.4)	,0.13)	,2372.1)	,0.16)	,180)	,0)	,541.3)	,0.18)	,147.3)	,0.1)	,0.6)	,-0.03)	,9)	,1.67)	,283.8)	,-0.03)	,137.7)	,0.04)	,112.7)	,0.15)	,34.5)	,-0.25)
	2097	-0.15	126.2	0.13	1019.7	-0.38	107.9	-0.02	523.3	0.1	125.5	0.36	0.7	0.03	7.9	-1.24	185.8	0	343.3	-0.17	324	-0.19	19.4	0.24
Serbia	(1474.3	(-0.19	(80.3	(0.09	(636.3	(-0.45	(62.3	(-0.02	(291	(0.03	(84.9	(0.34	(0.2	(0	(1.5	(-1.5	(119.2	(0	(218.3	(-0.3	(201.4	(-0.33	(9.3	(0.18
	,2813.6)	,-0.1)	,179.4)	,0.16)	,1534)	,-0.32)	,170.3)	,-0.01)	,845.4)	,0.17)	,177.2)	,0.37)	,1.5)	,0.05)	,17)	,-0.98)	,277.4)	,0.01)	,496.6)	,-0.03)	,473.5)	,-0.05)	,32.8)	,0.3)
	1742.5	-0.07	166.3	0.09	665.2	-0.24	81.7	-0.06	543.2	-0.01	93.1	0.29	0.6	0.11	6.8	-0.4	185.7	0	93.2	-0.18	68.4	-0.19	24.7	-0.16
Seychelles	(1248.5	(-0.12	(108.9	(0.06	(431.2	(-0.3	(46.3	(-0.07	(302.8	(-0.08	(62.1	(0.27	(0.2	(0.07	(1.6	(-0.74	(119.7	(-0.01	(58.4	(-0.27	(40	(-0.28	(12.5	(-0.24
	,2354.5)	,-0.03)	,230.5)	,0.12)	,974.9)	,-0.18)	,130.1)	,-0.06)	,913.5)	,0.06)	,132.6)	,0.31)	,1.2)	,0.15)	,14.3)	,-0.07)	,275.9)	,0.01)	,136.7)	,-0.09)	,107.3)	,-0.1)	,40.2)	,-0.07)
	2538.1	0.28	115.6	0.05	1664.6	0.38	112.4	0.02	346.4	0.12	103.5	0.14	0.3	0.02	5.7	1.77	189.7	0	99.3	0.05	78.6	0.14	20.7	-0.23
Sierra Leone	(1746.4	(0.24	(71.8	(0.02	(1013.1	(0.33	(63.5	(0.01	(193	(0.09	(69.2	(0.08	(0.1	(-0.05	(0.7	(1.5	(119.1	(-0.01	(63.2	(-0.02	(46.4	(0.04	(10.4	(-0.26
	,3548.4)	,0.31)	,165.9)	,0.09)	,2546.7)	,0.44)	,176.3)	,0.03)	,577)	,0.14)	,146)	,0.19)	,0.6)	,0.08)	,15.1)	,2.03)	,283.1)	,0.02)	,146.9)	,0.13)	,123.1)	,0.23)	,34.4)	,-0.2)
	1464.9	-0.47	152.6	0.12	468.5	-1.49	131.7	0.03	309.1	-0.06	207.9	0.48	0.7	0.14	0.2	-9.6	194.3	0.08	90.2	0.17	62.7	0.26	27.5	-0.03
Singapore	(1065.2	(-0.54	(99.8	(0.1	(294.2	(-1.68	(76.5	(0.02	(168.2	(-0.1	(142.6	(0.46	(0.2	(0.1	(0	(-10.22	(122.8	(0.07	(55	(0.1	(34.1	(0.19	(15.2	(-0.1
	,1891.2)	,-0.4)	,214.6)	,0.13)	,713.9)	,-1.3)	,205)	,0.04)	,520.2)	,-0.03)	,290.7)	,0.49)	,1.4)	,0.18)	,1.3)	,-8.99)	,289.4)	,0.09)	,135.4)	,0.24)	,103.4)	,0.33)	,44.5)	,0.03)
	2062.2	-0.08	131.5	0.22	959.2	-0.28	109.3	0.01	547.8	0.09	124.1	0.29	0.7	0.08	5.4	-1.72	184.3	0.03	289.1	0.02	263.9	0.05	25.3	-0.29
Slovakia	(1461.3	(-0.18	(82.6	(0.2	(597.2	(-0.46	(63.7	(0	(308	(0.01	(84.1	(0.27	(0.2	(0.04	(0.8	(-1.88	(117.3	(0.01	(182.2	(-0.11	(160.5	(-0.1	(13.1	(-0.34
	,2799.8)	,0.01)	,185.7)	,0.24)	,1436.2)	,-0.1)	,171.1)	,0.02)	,897)	,0.16)	,175)	,0.31)	,1.5)	,0.11)	,12.4)	,-1.55)	,274.8)	,0.04)	,426.9)	,0.15)	,398.2)	,0.19)	,40.7)	,-0.25)
	2232.1	-0.39	134.2	0.17	1138.1	-0.78	110.1	0	519.7	-0.07	138.2	0.57	0.7	0.29	4.8	-1.65	186.3	0.11	503.1	0.44	475.1	0.41	28	0.92
Slovenia	(1572.2	(-0.45	(85.9	(0.15	(730.7	(-0.87	(63.1	(-0.01	(285.4	(-0.15	(94	(0.55	(0.2	(0.25	(0.6	(-1.79	(119.4	(0.11	(325	(0.29	(303.3	(0.26	(15.1	(0.78
	,3021)	,-0.32)	,191.1)	,0.18)	,1709.9)	,-0.69)	,171.2)	,0.01)	,847.3)	,0.01)	,195.9)	,0.59)	,1.7)	,0.32)	,11.9)	,-1.51)	,277.5)	,0.12)	,714.1)	,0.58)	,680.3)	,0.56)	,45.2)	,1.06)
	1733	0.03	139	-0.15	700.3	0	69.9	-0.05	538.3	0.19	80.6	-0.08	0.8	-0.15	14.7	0.32	189.4	-0.1	103	-0.21	77.9	-0.24	25.1	-0.1
Solomon Islands	(1250.5	(0.01	(90.7	(-0.18	(456.8	(-0.02	(39.2	(-0.06	(306.8	(0.16	(53.5	(-0.1	(0.2	(-0.18	(5.1	(0.17	(120.7	(-0.1	(65.4	(-0.24	(47.4	(-0.28	(12.9	(-0.12
	,2310.4)	,0.05)	,199.2)	,-0.13)	,1045.5)	,0.03)	,112.9)	,-0.04)	,883.6)	,0.22)	,115.8)	,-0.06)	,1.6)	,-0.11)	,26.9)	,0.47)	,285.1)	,-0.09)	,149.6)	,-0.18)	,118.8)	,-0.2)	,40.2)	,-0.08)
	2835.6	0.08	93.9	-0.07	1905.7	0.1	128.8	0	418.8	0.18	88	-0.08	0.3	-0.1	17.4	0.5	182.7	-0.07	191.7	0.06	171	0.07	20.7	-0.01
Somalia	(1903	(0.05	(57.7	(-0.1	(1151.1	(0.05	(73.1	(0	(235.4	(0.12	(58.7	(-0.1	(0.1	(-0.14	(4.7	(0.31	(116.5	(-0.08	(121.5	(-0.04	(104.9	(-0.04	(10	(-0.04
	,3931.7)	,0.12)	,137.4)	,-0.04)	,2896.8)	,0.14)	,203.8)	,0.01)	,683.2)	,0.24)	,125.9)	,-0.06)	,0.6)	,-0.06)	,35.9)	,0.7)	,272.8)	,-0.06)	,282.5)	,0.15)	,256)	,0.18)	,34.3)	,0.02)
	2420.8	-0.04	123.1	0.07	1383.4	-0.1	122.3	-0.04	499.9	0.05	102.1	0.19	0.2	0.03	2.4	-0.27	187.3	-0.03	252.2	-0.25	187.1	-0.07	65.2	-0.7
South Africa	(1757.3	(-0.12	(88.5	(0.05	(928.8	(-0.22	(76.5	(-0.05	(329.5	(-0.03	(70	(0.13	(0.1	(0.01	(0.3	(-0.35	(122	(-0.04	(169	(-0.35	(116.7	(-0.14	(44.6	(-1.05
	,3152.3)	,0.04)	,160.5)	,0.09)	,1933.6)	,0.02)	,180.5)	,-0.04)	,729.8)	,0.14)	,142.2)	,0.25)	,0.5)	,0.05)	,6.6)	,-0.19)	,279.2)	,-0.02)	,347.1)	,-0.14)	,270.1)	,0.01)	,89.7)	,-0.35)
	2692.5	0.13	110.4	-0.11	1697.6	0.15	126.7	0.01	464.8	0.29	102.8	-0.03	0.3	-0.15	2	1.31	187.8	-0.07	205.4	-0.54	183	-0.61	22.4	0.05
South Sudan	(1829.4	(0.11	(69.9	(-0.15	(1042.4	(0.12	(74.5	(0	(262.6	(0.24	(68.3	(-0.04	(0.1	(-0.2	(0.5	(0.9	(121.1	(-0.08	(130.3	(-0.62	(112.6	(-0.69	(11.4	(0.01
	,3703.5)	,0.16)	,157.1)	,-0.06)	,2553.6)	,0.19)	,196.6)	,0.01)	,771.4)	,0.34)	,146.3)	,-0.02)	,0.6)	,-0.11)	,7)	,1.73)	,279.4)	,-0.06)	,305.4)	,-0.46)	,279.2)	,-0.52)	,35.5)	,0.1)
	2425.4	0.62	176.5	0	1086.3	1.12	211.3	0.09	599	0.44	132.5	0.42	1.7	0.12	28.3	-1.48	189.8	0.03	153.1	0	107.5	-0.09	45.6	0.22
Spain	(1747.9	(0.47	(125.6	(-0.01	(695.8	(0.86	(125.1	(0.07	(344.7	(0.18	(89.6	(0.38	(0.7	(0.08	(13	(-1.6	(121.1	(0.03	(99.2	(-0.16	(63.2	(-0.24	(27.4	(0
	,3246.9)	,0.77)	,227.6)	,0)	,1636.5)	,1.38)	,324.2)	,0.11)	,984.9)	,0.7)	,183.7)	,0.47)	,3.3)	,0.16)	,47.6)	,-1.36)	,282.3)	,0.04)	,226)	,0.15)	,171.6)	,0.05)	,67.5)	,0.45)
	1703	-0.15	156.7	0.18	609.8	-0.58	82.3	-0.04	563.3	0.23	96.9	0.17	0.6	-0.08	9.8	-1.36	183.7	-0.11	122.3	-1.16	99.1	-1.25	23.2	-0.71
Sri Lanka	(1228.6	(-0.2	(100.8	(0.14	(395.3	(-0.66	(46.3	(-0.05	(316.6	(0.16	(65.3	(0.14	(0.2	(-0.12	(2.7	(-1.67	(116.9	(-0.12	(78.6	(-1.35	(59.3	(-1.47	(11.7	(-0.77
	,2289.5)	,-0.09)	,219.8)	,0.21)	,895)	,-0.49)	,132.4)	,-0.04)	,931.6)	,0.29)	,138.3)	,0.2)	,1.2)	,-0.04)	,19.6)	,-1.04)	,272)	,-0.1)	,179.2)	,-0.96)	,152.8)	,-1.03)	,38.8)	,-0.64)
	2024.6	0.06	120	0.14	947.9	0.12	145.2	-0.01	489.9	0.05	92.1	0.31	1.2	0.01	35.8	-1.28	192.5	0.03	63.4	0.06	34.6	-0.3	28.8	0.54
Sudan	(1448.3	(0.03	(75.8	(0.13	(592.3	(0.08	(84.3	(-0.02	(284.5	(-0.02	(61.5	(0.3	(0.4	(-0.02	(15.7	(-1.37	(122.7	(0.02	(39	(0.05	(18.8	(-0.34	(15.1	(0.5
	,2726.3)	,0.1)	,170.2)	,0.16)	,1459.4)	,0.16)	,232)	,0)	,805.3)	,0.12)	,131.1)	,0.32)	,2.4)	,0.04)	,62.4)	,-1.19)	,287.4)	,0.03)	,93)	,0.07)	,56.9)	,-0.26)	,45.3)	,0.58)

	2473	0.18	128.8	0.04	1347.1	0.3	162.1	-0.05	564.1	0.14	77.6	-0.01	2.5	-0.14	4.4	-0.58	186.3	-0.09	199	-0.18	177.8	-0.21	21.2	0.03
Suriname	(1724	(0.13	(85.8	(0.02	(822.1	(0.23	(95.4	(-0.06	(321	(0.06	(52.1	(-0.02	(1.1	(-0.16	(0.5	(-0.7	(119.1	(-0.1	(122.7	(-0.24	(105.2	(-0.27	(11.5	(-0.01
	,3378.5)	,0.23)	,179.8)	,0.06)	,2070.6)	,0.36)	,252.4)	,-0.04)	,913.8)	,0.23)	,110.8)	,0)	,4.7)	,-0.12)	,10.6)	,-0.47)	,273.2)	,-0.08)	,295.7)	,-0.12)	,270.7)	,-0.14)	,34.9)	,0.07)
	2333.3	0.06	214.1	-0.05	951	0.07	218.2	0.07	616	0.04	129.2	0.32	0.9	0.17	9.7	-0.7	194.3	0.05	250.6	0.53	196.2	0.02	54.4	3.52
Sweden	(1682.2	(0.03	(150.1	(-0.06	(620.6	(0.01	(133	(0.05	(372.2	(-0.02	(87	(0.29	(0.3	(0.13	(2.9	(-0.94	(125.8	(0.04	(161.7	(0.45	(118.1	(-0.08	(33.9	(3.36
	,3047)	,0.09)	,286.6)	,-0.04)	,1410.6)	,0.13)	,330.2)	,0.09)	,905.9)	,0.09)	,183.7)	,0.35)	,1.7)	,0.21)	,18.9)	,-0.46)	,288.8)	,0.05)	,355.8)	,0.61)	,292.9)	,0.11)	,78.7)	,3.69)
	2549.3	-0.09	181.5	-0.02	1003.3	-0.44	212.8	0.1	837.7	0.14	117.5	0.33	0.9	0.06	6	-1.19	189.6	0.07	266.4	0.11	229.8	0.12	36.6	0.08
Switzerland	(1826.1	(-0.24	(117.7	(-0.04	(617.2	(-0.82	(126.1	(0.08	(495.3	(0.09	(78.9	(0.3	(0.3	(0.04	(1.5	(-1.25	(121.2	(0.06	(174.7	(0.06	(145.4	(0.05	(21.3	(0.03
	,3428.5)	,0.06)	,249.8)	,0)	,1507.7)	,-0.05)	,332)	,0.13)	,1290.3)	,0.19)	,167.7)	,0.35)	,1.9)	,0.09)	,12.7)	,-1.14)	,282.5)	,0.08)	,381.9)	,0.17)	,340)	,0.18)	,56.5)	,0.13)
	2070.6	0.08	123	0	929.1	0.1	147.1	-0.02	546.8	0.14	100.2	0.25	1.2	-0.06	33.6	-0.57	189.7	-0.04	84.4	0.57	43.5	0.12	40.8	1.12
Syrian Arab Republic	(1453.1	(0.04	(80.3	(-0.02	(582.1	(0.04	(85.1	(-0.03	(311.1	(0.08	(66.7	(0.21	(0.4	(-0.09	(12.6	(-0.68	(121.5	(-0.05	(53.9	(0.54	(24.6	(0.06	(23.5	(1.08
	,2832.8)	,0.12)	,171.6)	,0.02)	,1433.8)	,0.16)	,228.8)	,-0.02)	,887.1)	,0.2)	,142.5)	,0.29)	,2.3)	,-0.03)	,59.2)	,-0.46)	,283.2)	,-0.03)	,120.3)	,0.61)	,69.1)	,0.19)	,62.2)	,1.17)
	1830.2	0.09	187.8	-0.04	733.6	0.15	50.6	-0.02	535.6	0.13	131	0.15	0.7	-0.23	1.4	-3.47	189.5	-0.1	67.5	-0.07	35	0.21	32.6	-0.35
Taiwan (Province of China)	(1303.7	(0.07	(123.5	(-0.07	(469.6	(0.12	(27.5	(-0.03	(300.3	(0.11	(87.4	(0.12	(0.2	(-0.29	(0	(-3.71	(120.2	(-0.11	(42.7	(-0.14	(20	(0.09	(17.5	(-0.4
	,2422)	,0.1)	,258.6)	,-0.01)	,1083.5)	,0.17)	,82.6)	,-0.01)	,858.9)	,0.16)	,185.2)	,0.18)	,1.5)	,-0.16)	,4.5)	,-3.22)	,287.2)	,-0.09)	,95.6)	,0.01)	,55.6)	,0.34)	,50.8)	,-0.29)
	1882.6	-0.18	113.4	0	1022	-0.36	111.3	-0.01	324.1	-0.02	105.9	0.25	0.7	0.06	14.5	0.52	190.7	0.08	284.8	-0.66	253.6	-0.67	31.2	-0.56
Tajikistan	(1350.5	(-0.24	(70.3	(-0.07	(642.5	(-0.46	(63.9	(-0.02	(182.5	(-0.1	(71.4	(0.23	(0.2	(0.01	(3.5	(-0.22	(123.2	(0.07	(174.5	(-0.95	(150.9	(-0.99	(16.2	(-0.63
	,2560.8)	,-0.12)	,162.8)	,0.07)	,1549.2)	,-0.27)	,176.4)	,0)	,562.3)	,0.07)	,148.8)	,0.26)	,1.5)	,0.12)	,29.4)	,1.27)	,284.1)	,0.1)	,429.1)	,-0.37)	,394.9)	,-0.35)	,50.5)	,-0.48)
	1800.3	0.25	161.7	0.23	767	0.46	83.5	0.02	491.6	0.12	95.8	0.36	1.5	-0.05	11.2	-2.15	188	0.02	121.1	-0.64	91.6	-0.85	29.4	0.08
Thailand	(1295.3	(0.23	(103.9	(0.21	(499.7	(0.42	(46	(0.01	(277.7	(0.09	(64.5	(0.34	(0.5	(-0.07	(2	(-2.46	(120.1	(0.01	(74.8	(-0.67	(52.7	(-0.92	(14.8	(-0.03
	,2420.4)	,0.27)	,228)	,0.25)	,1126.6)	,0.51)	,133.8)	,0.03)	,824.4)	,0.15)	,137.3)	,0.38)	,2.9)	,-0.02)	,23.3)	,-1.83)	,281.7)	,0.02)	,180.5)	,-0.61)	,146.9)	,-0.79)	,48)	,0.19)
	1697	0	135.7	0.21	677.9	-0.14	79.2	0.02	521.3	0.2	82	0.18	0.6	-0.08	12.6	-2.38	187.8	-0.02	94.9	-0.26	75.5	-0.32	19.3	-0.05
Timor-Leste	(1186.3	(-0.03	(88	(0.16	(428.2	(-0.18	(44.1	(0	(290.3	(0.14	(55.7	(0.15	(0.2	(-0.11	(3.4	(-2.76	(119.9	(-0.03	(57.7	(-0.29	(43.1	(-0.35	(9.4	(-0.13
	,2264.7)	,0.03)	,191.1)	,0.26)	,996.8)	,-0.1)	,125.3)	,0.04)	,848.1)	,0.25)	,118)	,0.21)	,1.3)	,-0.05)	,24.7)	,-2)	,281.8)	,-0.01)	,139.1)	,-0.24)	,117.1)	,-0.28)	,33.2)	,0.03)
	2664.5	0.23	118.3	0.1	1780.1	0.3	114	0.02	363.8	0.21	98.8	-0.01	0.3	-0.08	4.3	1.78	184.9	-0.07	99	0.16	79	0.14	20	0.22
Togo	(1850.9	(0.22	(73.8	(0.08	(1101.6	(0.28	(64.2	(0.01	(198.4	(0.18	(65.9	(-0.06	(0.1	(-0.13	(0.6	(1.53	(118	(-0.08	(61.5	(0.09	(46.9	(0.06	(9.6	(0.2
	,3731.6)	,0.24)	,169.5)	,0.11)	,2720.3)	,0.33)	,181.5)	,0.03)	,624.2)	,0.23)	,140.7)	,0.04)	,0.6)	,-0.03)	,12.7)	,2.02)	,278.8)	,-0.06)	,146.5)	,0.22)	,122.8)	,0.21)	,33.8)	,0.24)
	1794.9	0.04	152.5	0	743.5	0.04	70	-0.04	545.3	0.12	88.4	0.09	0.8	-0.12	7.7	-1.01	186.5	-0.07	93.6	-0.26	66.4	-0.33	27.2	-0.08
Tokelau	(1287.2	(0.03	(100.6	(-0.02	(464.6	(0.02	(37.1	(-0.05	(313.6	(0.09	(57.9	(0.05	(0.2	(-0.21	(1.8	(-1.06	(120.2	(-0.08	(58.8	(-0.29	(38.2	(-0.37	(14.1	(-0.11
	,2425)	,0.05)	,215.6)	,0.02)	,1099.6)	,0.06)	,114.4)	,-0.04)	,892.6)	,0.14)	,126.3)	,0.13)	,1.7)	,-0.04)	,16.4)	,-0.95)	,276.2)	,-0.05)	,135.5)	,-0.23)	,103.5)	,-0.29)	,43.6)	,-0.06)
	1686.7	0.02	154	-0.08	626.3	0.01	71.2	-0.03	551	0.11	87.4	-0.01	0.8	-0.05	9.1	-0.15	186.9	-0.06	93.9	-0.16	66.8	-0.23	27.1	0.03
Tonga	(1218.3	(0	(100.5	(-0.09	(413.8	(-0.02	(39.1	(-0.04	(305.4	(0.07	(57.2	(-0.02	(0.3	(-0.08	(2.2	(-0.25	(119.7	(-0.07	(59.5	(-0.21	(39.4	(-0.3	(14.3	(-0.01
	,2266.2)	,0.04)	,216)	,-0.07)	,939)	,0.04)	,113.7)	,-0.03)	,916.7)	,0.14)	,125.1)	,0)	,1.7)	,-0.02)	,18.5)	,-0.04)	,279.9)	,-0.06)	,135.1)	,-0.11)	,101.6)	,-0.16)	,43.2)	,0.06)
	2166	0.02	145.9	0.53	1042.4	-0.09	162.3	-0.04	539.3	0.14	84	0.18	2.6	0.02	2.5	-2.25	187	-0.02	192.1	0.19	168.8	0.19	23.2	0.26
Trinidad and Tobago	(1536.5	(-0.04	(95.6	(0.41	(632.9	(-0.18	(95.5	(-0.04	(303	(0.05	(55.6	(0.17	(1.1	(0	(0.2	(-2.5	(118.1	(-0.03	(117.8	(0.01	(98.2	(-0.02	(12.7	(0.21
	,2907.5)	,0.09)	,203.8)	,0.65)	,1586.1)	,0)	,254.6)	,-0.03)	,891.3)	,0.23)	,118.5)	,0.19)	,4.9)	,0.04)	,6.9)	,-2.01)	,277.4)	,-0.01)	,286.1)	,0.37)	,258.2)	,0.39)	,36.2)	,0.31)
	2663.8	0.05	130.2	0.06	1514.1	0.05	148.5	-0.03	558.4	0.17	100.8	0.14	1.1	-0.08	24.6	-1.38	186.2	-0.07	64.4	0.18	32.5	-0.29	31.9	0.71
Tunisia	(1839.2	(-0.01	(83.8	(0.05	(936	(-0.04	(86.1	(-0.04	(315.4	(0.09	(67.8	(0.13	(0.4	(-0.11	(9	(-1.45	(120	(-0.07	(39.5	(0.14	(17.8	(-0.33	(16.8	(0.67
	,3679.4)	,0.1)	,184.4)	,0.07)	,2365.6)	,0.14)	,230.1)	,-0.02)	,903.5)	,0.26)	,143.8)	,0.16)	,2.2)	,-0.05)	,44.7)	,-1.3)	,277.4)	,-0.06)	,94.3)	,0.22)	,53.7)	,-0.24)	,51.7)	,0.76)
Turkey	1922.1	0.08	132.5	0.1	864.9	-0.14	151	-0.03	469.3	0.64	100.3	0.38	1.7	-0.06	15.5	-1.62	186.9	-0.02	63.1	-0.16	36.5	-0.24	26.6	-0.04
	(1342.9	(0.01	(88.6	(0.09	(528.6	(-0.21	(87.6	(-0.03	(255.7	(0.45	(66.6	(0.36	(0.7	(-0.08	(4.3	(-1.69	(118.5	(-0.03	(38.6	(-0.18	(19.4	(-0.29	(12.7	(-0.11

	,2598.4)	,0.14)	,182.9)	,0.11)	,1324.4)	,-0.08)	,235.8)	,-0.02)	,792)	,0.83)	,143.9)	,0.4)	,3.2)	,-0.04)	,30.6)	,-1.55)	,282.5)	,-0.02)	,94)	,-0.13)	,59.7)	,-0.2)	,44.5)	,0.03)
	2026	-0.1	126.4	0.29	1161.1	-0.25	111.1	0	328.2	0.12	104.9	0.27	0.7	-0.04	6.7	-2.25	186.8	0.04	292.5	-0.56	256.9	-0.57	35.6	-0.43
Turkmenistan	(1397.4	(-0.16	(80.4	(0.23	(727.1	(-0.33	(63.6	(0	(173.8	(0.05	(70	(0.26	(0.2	(-0.09	(1.3	(-2.8	(117.4	(0.03	(188	(-0.88	(160	(-0.94	(19.4	(-0.47
	,2708.6)	,-0.05)	,175.5)	,0.35)	,1702.1)	,-0.18)	,178)	,0.01)	,547.4)	,0.2)	,148.8)	,0.28)	,1.4)	,0.01)	,14.7)	,-1.69)	,280)	,0.05)	,421.5)	,-0.23)	,382.8)	,-0.21)	,56.3)	,-0.4)
	1801.2	0.03	145.1	0	751.2	0.01	70.3	-0.01	555.9	0.06	80.5	0.22	0.8	0	11.5	-0.51	186	0.01	98.4	-0.19	70.7	-0.23	27.7	-0.09
Tuvalu	(1272.3	(0.01	(95	(-0.02	(474.8	(0	(38.8	(-0.02	(311	(0.02	(53.5	(0.2	(0.3	(-0.04	(3.4	(-0.68	(118	(0	(63	(-0.23	(41.5	(-0.29	(14.3	(-0.1
	,2435.8)	,0.05)	,202.8)	,0.01)	,1140.9)	,0.03)	,112.8)	,0)	,906.1)	,0.1)	,115.6)	,0.23)	,1.6)	,0.03)	,22.5)	,-0.33)	,278.5)	,0.01)	,141.5)	,-0.15)	,109.9)	,-0.17)	,44.6)	,-0.08)
	3762.4	-0.02	111.4	0.26	2782.2	-0.1	129	0.04	447.5	0.39	100.2	0.11	0.3	-0.08	7.4	-0.51	184.5	-0.03	155.2	0.06	131.9	0.05	23.4	0.14
Uganda	(2519.9	(-0.09	(70.6	(0.24	(1735.9	(-0.2	(73.6	(0.03	(242	(0.34	(66.9	(0.08	(0.1	(-0.11	(1.4	(-0.9	(118.5	(-0.04	(99.2	(0.02	(81	(-0.01	(11.7	(0.1
	,5271.2)	,0.05)	,162.2)	,0.28)	,4229.1)	,0)	,203)	,0.05)	,747.9)	,0.45)	,144.4)	,0.13)	,0.6)	,-0.05)	,16.9)	,-0.12)	,276.8)	,-0.02)	,226.7)	,0.11)	,199.5)	,0.1)	,37.5)	,0.19)
	2452.9	-0.39	111	0.06	1455.3	-0.7	113.5	0.02	468.8	0.13	109.1	0.21	0.6	0.02	8	-0.02	186.8	0.06	334.5	-0.85	286.4	-0.72	48.1	-1.6
Ukraine	(1713.5	(-0.47	(73.2	(0.01	(914	(-0.83	(69.4	(0.01	(281.5	(0.01	(74.5	(0.19	(0.2	(-0.01	(1.2	(-0.49	(119.8	(0.05	(213.9	(-1.05	(176.8	(-0.94	(27.7	(-1.87
	,3342.8)	,-0.31)	,153.4)	,0.11)	,2157.6)	,-0.57)	,173.1)	,0.03)	,711.4)	,0.26)	,153.4)	,0.23)	,1.2)	,0.05)	,17.7)	,0.45)	,283.2)	,0.06)	,475.8)	,-0.64)	,418.5)	,-0.51)	,74.5)	,-1.32)
	1875.1	-0.12	148.1	-0.13	839.5	-0.22	143.2	-0.05	412.8	-0.32	116.2	0.63	0.4	0.25	11.6	0.69	203.3	0.2	99.5	0.38	46.3	-0.24	53.3	1
United Arab Emirates	(1329.4	(-0.16	(96.1	(-0.15	(522.4	(-0.28	(81.4	(-0.06	(232	(-0.37	(76.3	(0.57	(0.1	(0.19	(2.8	(0.58	(132.2	(0.17	(63.4	(0.21	(25	(-0.34	(30.9	(0.74
	,2465)	,-0.08)	,208)	,-0.1)	,1283.4)	,-0.16)	,226.2)	,-0.04)	,675.1)	,-0.26)	,165.2)	,0.68)	,1.1)	,0.3)	,23.5)	,0.79)	,301.7)	,0.22)	,144.8)	,0.55)	,76.4)	,-0.14)	,81.5)	,1.26)
	2278.2	0.21	149.8	-0.13	997.4	0.01	237.3	0.04	596.8	0.78	95.4	0.61	0.3	0.13	9.1	-1.46	192.1	0.04	229.6	1.71	173.2	2.02	56.5	0.88
United Kingdom	(1637.8	(0.16	(109.1	(-0.23	(651.2	(-0.06	(149.8	(0.03	(396.2	(0.63	(65.5	(0.56	(0.1	(0.12	(3.1	(-1.57	(125.2	(0.04	(155	(1.61	(111.5	(1.87	(39.3	(0.76
	,2969.4)	,0.25)	,191)	,-0.03)	,1410.8)	,0.09)	,348.3)	,0.05)	,856.9)	,0.92)	,132.6)	,0.67)	,0.7)	,0.15)	,17)	,-1.35)	,285.1)	,0.05)	,313.8)	,1.82)	,251.2)	,2.17)	,75.4)	,1)
	2749.3	0.03	114.7	0.22	1777.6	-0.04	129.6	0.02	429.2	0.21	104.7	0.19	0.3	0	4.5	-0.97	188.7	0.03	228.3	0.22	196.3	0.15	32	0.7
United Republic of Tanzania	(1869.4	(0	(72.8	(0.18	(1080.5	(-0.06	(73.9	(0.02	(242	(0.17	(68.9	(0.16	(0.1	(-0.05	(0.5	(-1.2	(120.3	(0.01	(145.6	(0.13	(119.5	(0.05	(17.9	(0.66
	,3766.7)	,0.05)	,163.2)	,0.25)	,2658.5)	,-0.01)	,204.4)	,0.03)	,723.6)	,0.25)	,149.1)	,0.22)	,0.6)	,0.05)	,12.3)	,-0.74)	,279.8)	,0.04)	,340.6)	,0.31)	,299.9)	,0.24)	,48.8)	,0.75)
	1961.1	0.09	195.4	0.08	650.5	0.15	118.8	-0.06	650.5	0.1	138.9	0.08	0.8	-0.02	8.9	-1.62	197.2	-0.04	314.5	0.26	150.9	0.28	163.6	-0.03
United States of America	(1465.2	(0.05	(141.4	(0.03	(439.7	(0.09	(79	(-0.06	(437.3	(0.04	(96.4	(0.07	(0.4	(-0.05	(2.8	(-2.1	(129.8	(-0.05	(218.7	(0.14	(94.9	(0.15	(111.5	(-0.09
	,2508.4)	,0.13)	,252.3)	,0.13)	,903.5)	,0.21)	,167.4)	,-0.05)	,928.7)	,0.17)	,192.7)	,0.09)	,1.6)	,0)	,17.2)	,-1.13)	,288.1)	,-0.04)	,417)	,0.38)	,216.2)	,0.42)	,217.9)	,0.02)
	2093.8	-0.08	152.4	-0.04	941.4	0.03	163.7	-0.06	558.1	-0.31	87.2	0.23	2.6	-0.01	2	-0.75	186.3	0	325.5	2.07	300.2	0.71	25.4	4.25
United States Virgin Islands	(1463.1	(-0.18	(98.1	(-0.08	(562.1	(-0.07	(94.8	(-0.06	(319.5	(-0.56	(57.4	(0.19	(1.1	(-0.17	(0.1	(-0.79	(117.6	(0	(206.6	(1.84	(187.5	(0.48	(14	(4.04
	,2882.2)	,0.02)	,213.3)	,-0.01)	,1412.3)	,0.13)	,257.6)	,-0.05)	,940.5)	,-0.07)	,123.8)	,0.26)	,4.7)	,0.15)	,5.9)	,-0.71)	,278.7)	,0.01)	,493.5)	,2.31)	,462.7)	,0.95)	,40.3)	,4.47)
	1899.9	0.13	160.4	0.03	664.3	0.28	156.5	-0.05	593.1	0.11	129.5	0.15	0.6	-0.01	8.6	-1.25	187.1	-0.02	159.4	0.27	125.3	0.42	34.1	-0.25
Uruguay	(1351.3	(0.09	(104.3	(0	(416.6	(0.18	(92	(-0.06	(334.4	(0.04	(86.4	(0.14	(0.1	(-0.05	(2.4	(-1.39	(119.1	(-0.02	(105.6	(0.09	(80	(0.2	(18.9	(-0.31
	,2514)	,0.17)	,226.1)	,0.05)	,1035.7)	,0.37)	,244.7)	,-0.04)	,968.2)	,0.18)	,183)	,0.16)	,1.2)	,0.04)	,17.2)	,-1.1)	,278.3)	,-0.01)	,221.1)	,0.45)	,181.3)	,0.65)	,53.1)	,-0.2)
	1971.1	-0.2	117.7	0.18	1151.5	-0.35	110.1	-0.02	288.6	-0.05	106.8	0.27	0.7	0.06	8.7	-1.17	186.9	0.04	207.9	-2.21	173.7	-2.53	34.2	-0.06
Uzbekistan	(1379.9	(-0.25	(74.9	(0.14	(719.9	(-0.44	(62.4	(-0.03	(163.2	(-0.12	(72	(0.26	(0.2	(0.02	(1.7	(-1.54	(119.3	(0.03	(128	(-2.7	(99.3	(-3.08	(18.4	(-0.12
	,2646.8)	,-0.14)	,168.8)	,0.22)	,1702.7)	,-0.27)	,173.1)	,-0.01)	,486.6)	,0.02)	,150.5)	,0.28)	,1.4)	,0.11)	,18.8)	,-0.8)	,276.4)	,0.05)	,310.9)	,-1.73)	,273.1)	,-1.98)	,54.3)	,0.01)
	1744.1	0.02	142.5	-0.11	699.9	-0.03	70.5	-0.03	550.6	0.18	80.4	-0.08	0.8	-0.15	10.6	0.2	188.8	-0.06	97	-0.17	71.7	-0.22	25.3	-0.02
Vanuatu	(1257.3	(0	(90.6	(-0.12	(445.1	(-0.06	(38.1	(-0.04	(312.5	(0.14	(53.8	(-0.09	(0.2	(-0.19	(2.5	(0.14	(120.2	(-0.07	(62	(-0.19	(43.2	(-0.25	(13.6	(-0.03
	,2295.8)	,0.04)	,201)	,-0.09)	,1021.4)	,0)	,113.5)	,-0.02)	,914.7)	,0.22)	,115)	,-0.08)	,1.6)	,-0.12)	,21.9)	,0.26)	,281.4)	,-0.06)	,139.5)	,-0.14)	,109.2)	,-0.18)	,39.9)	,0)
	2108.8	0.01	143.1	0.01	1045.4	-0.08	150.3	-0.01	484.8	0.19	92.5	0.18	2	-0.05	4.3	-0.41	186.3	-0.02	164.2	-1.29	146.4	-1.4	17.9	-0.27
Venezuela (Bolivarian Republic of)	(1460	(-0.02	(91.7	(-0.02	(628	(-0.12	(88	(-0.02	(269.9	(0.14	(61.9	(0.16	(0.8	(-0.07	(0.4	(-0.74	(119.7	(-0.03	(98.2	(-1.45	(83.5	(-1.58	(9.1	(-0.32
	,2891.3)	,0.04)	,202.3)	,0.04)	,1600)	,-0.03)	,232.9)	,0)	,791.3)	,0.24)	,131.3)	,0.21)	,3.9)	,-0.03)	,10.6)	,-0.08)	,277.6)	,-0.02)	,250.2)	,-1.14)	,230.8)	,-1.23)	,29.5)	,-0.22)

Vietnam	1462.7 (1067.7 ,1960.1)	0.01 (-0.04 ,0.07)	166.1 (109 ,231.6)	0.29 (0.27 ,0.31)	604.1 (386.8 ,905.4)	-0.04 (-0.12 ,0.03)	84 (46.9 ,134.5)	0.01 (0 ,0.02)	320.9 (181.1 ,548.3)	0.04 (-0.1 ,0.17)	91.6 (62.6 ,130.3)	0.35 (0.34 ,0.37)	0.6 (0.2 ,1.3)	0 (-0.03 ,0.03)	9.3 (2.9 ,18.6)	-3.06 (-3.49 ,-2.62)	186.1 (119 ,277.8)	0.01 (0 ,0.01)	124.4 (76.3 ,184.4)	0.49 (0.44 ,0.55)	98.4 (56.2 ,153.4)	0.61 (0.54 ,0.68)	25.9 (13.1 ,42.3)	0.07 (0.02 ,0.13)
Yemen	2124.2 (1483.2 ,2870.9)	0.07 (0.05 ,0.09)	114.9 (71.6 ,165)	0.01 (-0.03 ,0.05)	1062 (648.9 ,1639.1)	0.16 (0.15 ,0.18)	145.8 (84.5 ,224.6)	-0.01 (-0.02 ,-0.01)	486.4 (274.5 ,797.8)	-0.05 (-0.09 ,-0.02)	85.1 (56.4 ,120.4)	0.22 (0.18 ,0.26)	0.8 (0.3 ,1.6)	-0.05 (-0.08 ,-0.02)	41.2 (18.6 ,71.5)	-0.45 (-0.66 ,-0.25)	187.8 (120.8 ,279.8)	0.03 (0.01 ,0.04)	62.1 (38.8 ,91.3)	0.16 (0.15 ,0.18)	33.7 (18.5 ,54.8)	-0.2 (-0.23 ,-0.17)	28.4 (14.7 ,46)	0.66 (0.63 ,0.69)
Zambia	2543.9 (1753.5 ,3536.3)	0.15 (0.09 ,0.22)	115.2 (73.3 ,163.4)	0.24 (0.19 ,0.29)	1558.2 (966 ,2372.9)	0.13 (0.05 ,0.21)	127.6 (73.7 ,199.6)	0 (-0.01 ,0.01)	452.9 (250.5 ,756)	0.42 (0.36 ,0.48)	100.9 (68.3 ,145.7)	0.06 (0 ,0.11)	0.3 (0.1 ,0.6)	-0.07 (-0.11 ,-0.03)	3 (0.4 ,9.1)	-0.16 (-0.63 ,0.32)	185.8 (118.8 ,276.6)	-0.09 (-0.1 ,-0.08)	231.3 (149.3 ,335.2)	-0.1 (-0.16 ,-0.03)	204.2 (124.9 ,305.8)	-0.11 (-0.18 ,-0.04)	27.1 (14.7 ,42.8)	0.04 (0.02 ,0.07)
Zimbabwe	2154.4 (1516.3 ,2949.4)	0.1 (0.08 ,0.13)	110.4 (69.7 ,159.3)	-0.17 (-0.21 ,-0.14)	1247.8 (805.9 ,1898.7)	0.2 (0.17 ,0.23)	119.6 (69.2 ,187.8)	-0.03 (-0.04 ,-0.02)	392.9 (219.6 ,658.6)	0.13 (0.08 ,0.19)	93.7 (62.1 ,134.2)	-0.4 (-0.44 ,-0.36)	0.3 (0.1 ,0.6)	-0.29 (-0.32 ,-0.27)	6.8 (1.5 ,15.2)	1.75 (1.37 ,2.14)	182.9 (119.4 ,272.2)	-0.1 (-0.11 ,-0.1)	188.6 (123.6 ,269.9)	-0.54 (-0.62 ,-0.46)	125.4 (72.9 ,198.3)	-0.77 (-0.87 ,-0.66)	63.2 (39.9 ,90.4)	-0.05 (-0.09 ,0)

Note: YLDS-years lived with disability; EAPC-estimated annual percentage changes ; 95% CI-95% confidence intervals(EAPC); 95% UI-95% uncertainty interval

eTable 9. GATHER checklist of information that should be included in reports of global health estimates

Item	Checklist item	Reported On Page
**Objectives and funding**		
1	Define the indicator(s), populations (including age, sex, and geographic entities), and time period(s) for which estimates were made.	Page 1 (Abstract);<br>Page 4-5 (Methods)
2	List the funding sources for the work.	Page 14 (Funding)
**Data inputs**		
**For all data inputs from multiple sources that are synthesised as part of the study:**		
3	Describe how the data were identified and how the data were accessed.	Page 4-5 (Methods);<br>Page 1 (Supplement 1)
4	Specify the inclusion and exclusion criteria. Identify all ad-hoc exclusions.	Page 4-5 (Methods);<br>Page 1 (Supplement 1)
5	Provide information about all included data sources and their main characteristics. For each data source used, report reference information or contact name/institution, population represented, data collection method, year(s) of data collection, sex and age range, diagnostic criteria or measurement method, and sample size, as relevant.	Page 4-5 (Methods);<br>Page 3, 6-7 (Supplement 1)
6	Identify and describe any categories of input data that have potentially important biases (eg, based on characteristics listed in item 5).	Page 4-5 (Methods);<br>Page 3, 6-7 (Supplement 1)
**For data inputs that contribute to the analysis but were not synthesised as part of the study:**		
7	Describe and give sources for any other data inputs.	Page 1-2 (Supplement 1)
**For all data inputs:**		

8	Provide all data inputs in a file format from which data can be efficiently extracted (eg, a spreadsheet rather than a PDF), including all relevant meta-data listed in item 5. For any data inputs that cannot be shared because of ethical or legal reasons, such as third-party ownership, provide a contact name or the name of the institution that retains the right to the data.	Page 15 (Data sharing)
**Data analysis**		
9	Provide a conceptual overview of the data analysis method. A diagram may be helpful.	Page 5-6 (Methods)
10	Provide a detailed description of all steps of the analysis, including mathematical formulae. This description should cover, as relevant, data cleaning, data pre-processing, data adjustments and weighting of data sources, and mathematical or statistical model(s).	Page 5-6 (Methods)
11	Describe how candidate models were evaluated and how the final model(s) were selected.	Page 5-6 (Methods)
12	Provide the results of an evaluation of model performance, if done, as well as the results of any relevant sensitivity analysis.	Not applicable
13	Describe methods of calculating uncertainty of the estimates. State which sources of uncertainty were, and were not, accounted for in the uncertainty analysis.	Page 5-6 (Methods)
14	State how analytical or statistical source code used to generate estimates can be accessed.	Page 15 (Data sharing)
**Results and discussion**		

15	Provide published estimates in a file format from which data can be efficiently extracted.	Page 19-22 (Table 1)
16	Report a quantitative measure of the uncertainty of the estimates (eg, uncertainty intervals).	Page 6-10 (Results);<br>Page 19-22 (Table 1);<br>Page 8-64 (Supplement 1)
17	Interpret results in light of existing evidence. If updating a previous set of estimates, describe the reasons for changes in estimates.	Not applicable
18	Discuss limitations of the estimates. Include a discussion of any modelling assumptions or data limitations that affect interpretation of the estimates.	Page 13 (Discussion)

This checklist should be used in conjunction with the GATHER statement and Explanation and Elaboration document, found on gather-statement.org
